# Palladium-Catalyzed *Ortho* C–H Arylation of Unprotected Anilines: Chemo- and Regioselectivity Enabled by the Cooperating Ligand [2,2′-Bipyridin]-6(1*H*)-one

**DOI:** 10.1021/acscatal.2c05206

**Published:** 2022-11-11

**Authors:** Cintya Pinilla, Vanesa Salamanca, Agustí Lledós, Ana C. Albéniz

**Affiliations:** † IU CINQUIMA/Química Inorgánica, Universidad de Valladolid, 47071 Valladolid, Spain; ‡ Departament de Química, Universitat Autònoma de Barcelona, 08193 Barcelona, Spain

**Keywords:** C−H activation, unprotected anilines, direct arylation, palladium, metal−ligand cooperation, pyridones

## Abstract

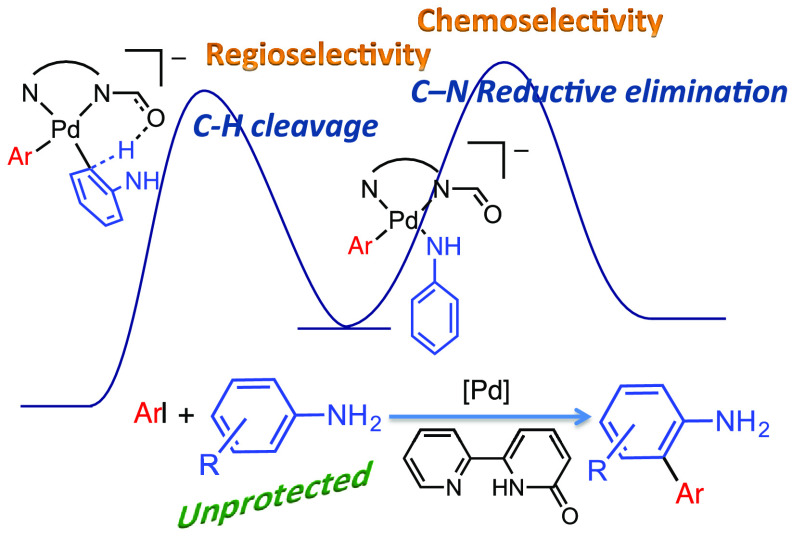

Metal-catalyzed C–H functionalizations on the aryl ring of anilines usually need cumbersome N-protection–deprotection strategies to ensure chemoselectivity. We describe here the Pd-catalyzed direct C–H arylation of unprotected anilines with no competition of the N-arylation product. The ligand [2,2′-bipyridin]-6(1*H*)-one drives the chemoselectivity by kinetic differentiation in the product-forming step, while playing a cooperating role in the C–H cleavage step. The latter is favored in an anionic intermediate where the NH moiety is deprotonated, driving the regioselectivity of the reaction toward *ortho* substitution.

Anilines are attractive substrates for C–H functionalization. Many biologically relevant compounds have the aniline motif in their structure; thus, there is a great deal of interest in developing efficient derivatization methods of the parent aniline that can be used in the synthesis of more complex molecules. The functionalization by direct transformation of the C–H bonds of the aryl ring of aniline into C–C bonds allows the synthesis of useful derivatives in a lower number of steps, taking into account that there is no need to prepare the intermediate reagents required for conventional coupling reactions. Most metal-catalyzed processes of this type use N-protected anilines as substrates.^1^ Tertiary anilines, anilides, or anilines bearing N-bound directing groups such as 2-pyridyl have been functionalized in a number of ways. For example, the alkenylation of protected anilines using the Fujiwara–Moritani or oxidative Heck reaction of arenes has been reported,^2,3^ as well as arylation (Scheme 1a),^4−7^ alkylation,^8^ alkynylation,^9^ and acylation reactions.^10^ The presence of the protecting group directs the *ortho* selectivity observed in most cases, via chelate-assisted C–H activation. Also, the careful design of the protecting group allows the selective synthesis of other regioisomers.^4e,11^

**Scheme 1:**
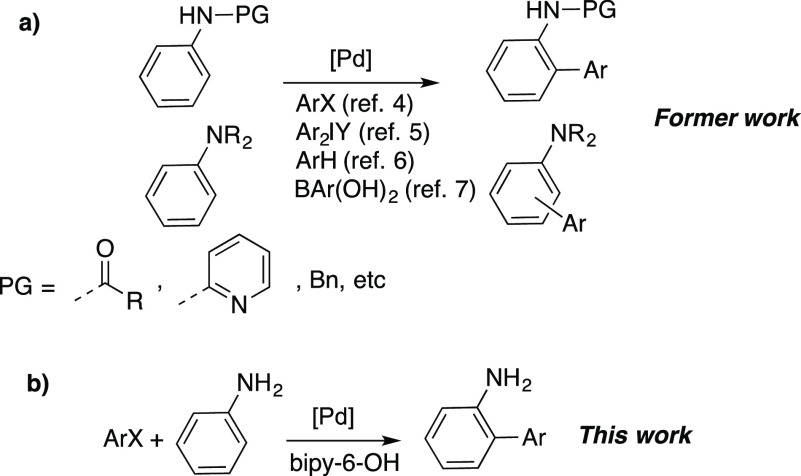
C–H Arylation of Protected Secondary and Tertiary Anilines (a) and Unprotected Anilines (b)

The use of unprotected primary anilines in C–H activation reactions is rare. Fernández-Ibáñez reported the *para*-selective palladium-catalyzed alkenylation of anilines, and a few examples of the functionalization of *ortho*-disubstituted primary anilines were included.^3^*Ortho*-substituted aryl anilines have been used as substrates in Pd-catalyzed C–H functionalization, but in these cases the −NH_2_ group directs the functionalization to the aryl substituent rather than the aniline ring.^12^ To our knowledge, no examples of palladium-catalyzed direct arylation of unprotected anilines have been reported. This is not surprising, since the combination of an aryl halide and ArNH_2_ could easily produce the Buchwald–Hartwig amination product, leading to N–H functionalization and the corresponding secondary aniline. Therefore, N-protection is the common practice. It would be very interesting to develop a chemoselective catalytic system that could functionalize the aryl ring with no interference of the amino group, so that the additional protection–deprotection steps could be avoided.

A few methods for the arylation of primary anilines have been reported, based on radical reactions of aryl diazo derivatives or arylhydrazines, both being rather hazardous reagents.^13^*Ortho* arylation of anilines has been achieved via the *in situ* generation of benzyne intermediates.^14^ Daugulis et al. described such a reaction using ArCl as a benzyne precursor in the presence of a strong lithium base, which was applied to a large number of anilines.^15^

We describe here a palladium catalytic system that brings about the selective *ortho* arylation of unprotected anilines (Scheme 1, b). The use of the ligand [2,2′-bipyridin]-6(1*H*)-one (bipy-6-OH) is crucial. It is responsible for the activity of the catalyst by playing a cooperating role in the C–H cleavage.^16,17^ It is also important in determining the selectivity of the process by favoring the C–C vs the C–N coupling (chemoselectivity) and also the *ortho* regioselectivity.

The well-defined complex [Pd(bipy-6-OH)Br(C_6_F_5_)] (**1**) was tested in the reaction of aniline with *p*-CF_3_C_6_H_4_I, an aryl halide that allows the easy monitoring of the reaction by ^19^F NMR. Following our previous work,^18^ we used pinacolone as the solvent and a moderate excess of aniline (10-fold). The reaction goes to completion in 24 h, and good yields of the *ortho*-arylated aniline were obtained. Similar results can be achieved in a much shorter time (6 h) using DMA (entries 1 and 2, Table 1); therefore, this solvent was selected for our experiments. The reaction is equally effective when an equimolar mixture of palladium acetate and bipy-6-OH was used as the precatalyst (cf. entries 2 and 3, Table 1). The presence of the cooperating ligand is necessary, and negligible conversion was observed when no ligand was added, when the pyridone moiety was not present in the ligand, or when it was in a position far from the metal so that it was not able to play a cooperating role (entries 4–6, Table 1). In contrast, the ligand phen-2-OH is also effective, although the reaction is slower. The amount of aniline reactant can be reduced to almost the stoichiometric amount at the expense of a moderate reduction of the yield and a much longer reaction time (entry 8, Table 1).

**Table 1:** Arylation of Aniline with *p*-CF_3_C_6_H_4_I Using Different Catalysts According to eq 1^a^

		crude yield, % (conversn, %)	
entry	[Pd]	6 h^b^	24 h^b^
1^c^	**1**	46 (51)	92 (100)
2	**1**	83 (100)	
3	[Pd(OAc)_2_] + bipy-6-OH	86 (100)	
4	[Pd(OAc)_2_]	0 (3)	0 (9)
5	**2**	0 (4)	1 (10)
6	**3**	0 (0)	0 (6)
7	**4**	31 (40)	91 (100)
8^d^	**1**	13 (20)	74 (100)

a Reaction conditions unless specified otherwise: *p*-CF_3_C_6_H_4_I (0.34 mmol), aniline (3.4 mmol), [Pd] (5 mol %), Cs_2_CO_3_ (0.68 mmol), DMA (2.7 mL); 130 °C.

b Crude yields determined by ^19^F NMR of the reaction mixture. Mixture of regioisomers *o*:*m*:*p* = 25:1:1. The reduction of the aryl iodide (ArH) and homocoupling (Ar–Ar) are the observed byproducts.

c Pinacolone as solvent.

d Aniline (0.37 mmol).

The *ortho*-arylated product was the major one and only 5% of the Buchwald-Hartwig amination product (N-arylation) was detected in the crude mixture (Figure S21 in the Supporting Information). In fact, the amination product was obtained cleanly when the same base (Cs_2_CO_3_) and solvent (DMF) similar to those in eq 1 were used, but a different Pd catalyst: a mixture of a Pd(0) precursor and XPhos (eq 2). Thus, the Pd-bipy-6-OH catalyst system can be used in combination with other catalysts for the orthogonal functionalization of aniline by C–C and C–N coupling.
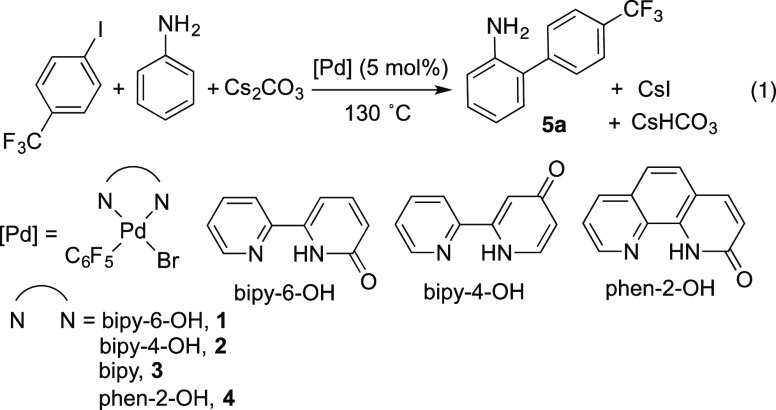
1
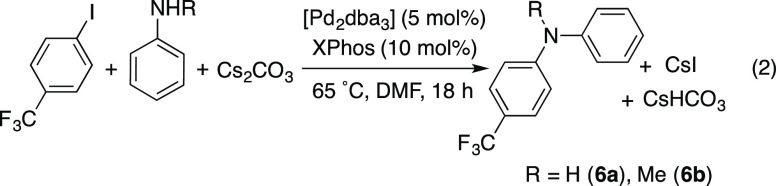
2

The selective *ortho* arylation can be extended to primary anilines with different substitution patterns in the aromatic ring (*ortho*-, *meta*- and *para*-substituted). Electron-withdrawing and electron-donating groups are tolerated in the aniline and in the aryl halide (Scheme 2). Only monoarylation was observed in all cases, and the *ortho*-arylated anilines were obtained in good to moderate yields, with the only exception being the tertiary dimethylaniline (**5n**). Some of the derivatives shown can be interesting precursors of biologically active compounds as, for example, in the synthesis of biphenylbenzamide microbiocidal agents (i.e., **5a**–**c**,**f**,**g**),^19^ carbazole alkaloids,^20^ and dyes (**5k**).^21^ Again, in the few cases that it was observed, the C–N coupling product only accounts for about 2–5% of the crude yield (see the Supporting Information). Note that when *o*-phenylaniline is used as substrate only the functionalization of the aniline ring occurs and the aryl substituent remains unaltered, in contrast to other reactions in the literature that use 2-anilino as a directing group.^12^

**Scheme 2:**
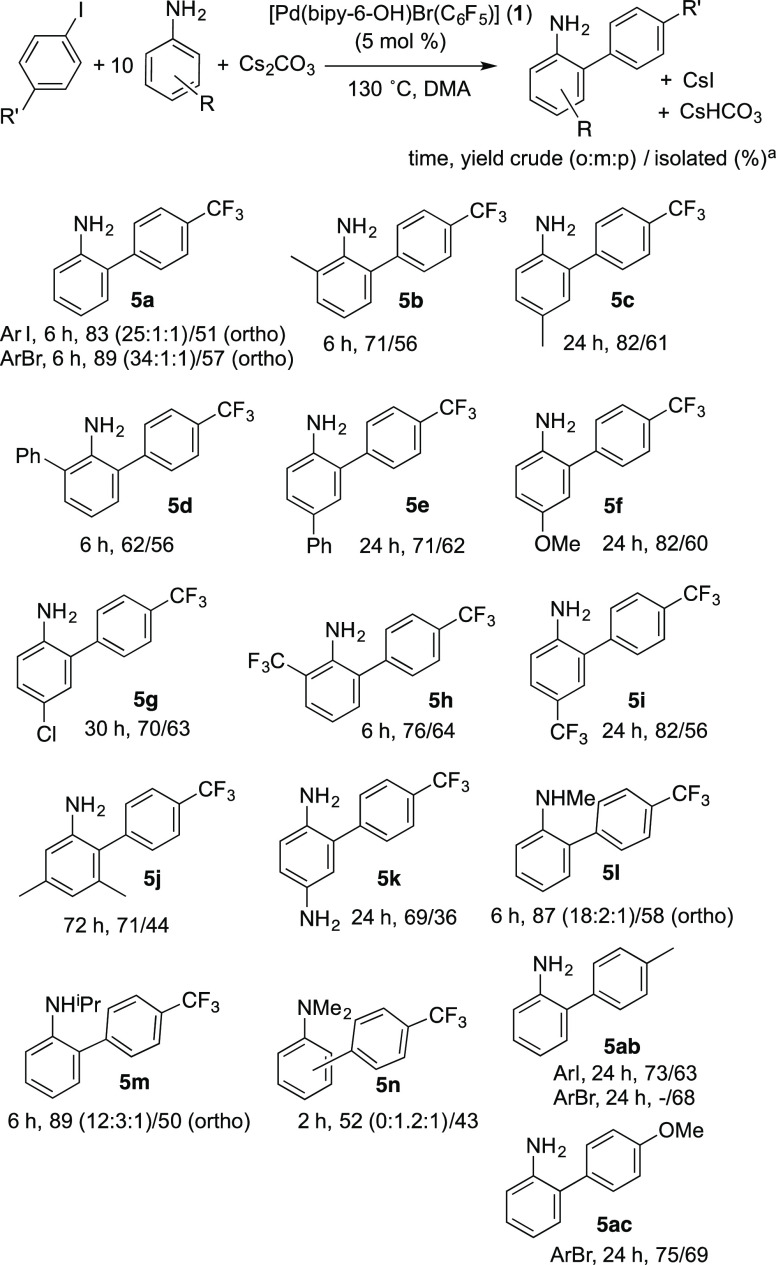
Arylation of Anilines with Complex **1** Reaction conditions given in Table 1.

Scheme 2 also shows the arylation products of several secondary and tertiary anilines. We observed that the regioselectivity is eroded as the N-substitution increases. However, whereas the *ortho* isomer is still the major isomer for secondary anilines, the arylation of *N*,*N*-dimethylaniline only affords a mixture of the *meta* and *para* isomers.

Mechanistic experiments were carried out to gather information on the catalytic cycle and the origin of the selectivity observed. The reaction shown in eq 3 was used as a model. Complex **1** is transformed under catalytic conditions (DMA, excess of aniline) into the amino derivative **7**, which was independently synthesized and characterized (eq 4; see molecular structure in Figure S10 in the Supporting Information). The analogous complex [Pd(bipy-6-O)(*p*-CF_3_C_6_H_4_)(PhNH_2_)] (**8**) was also prepared, and it is catalytically competent for the reaction in eq 3 (90% yield in 6 h). It also decomposes under catalytic conditions to the *ortho*-arylated aniline (eq 5); thus, the presence of **8** is plausible in the catalytic reaction.
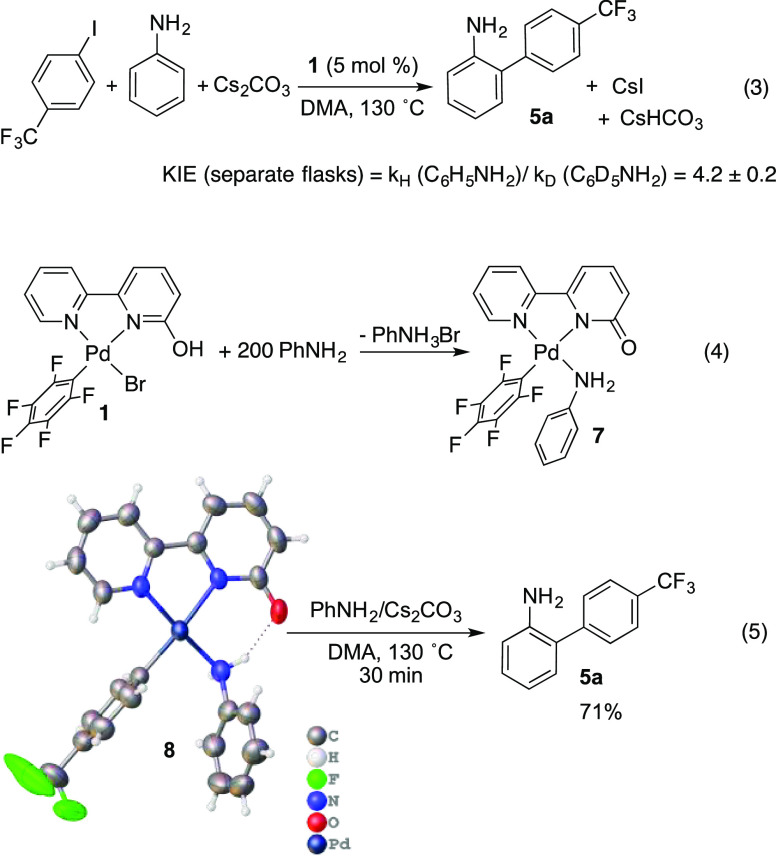
3

Kinetic experiments show that the rate of the reaction exhibits a first-order dependence on the catalyst (complex **1**) and is independent of the concentration of the aryl halide. The reaction rate in also insensitive to the concentration of aniline in the excess range used for this reactant in the catalysis (about 10-fold). As can be seen in eq 4, under these conditions the coordination equilibrium is completely shifted to the aniline-coordinated species (Figure S1 in the Supporting Information). A large kinetic isotope effect was found (KIE = 4.2 ± 0.4), pointing to the C–H activation as the turnover-limiting step. The reaction in eq 3 was also carried out in the presence of D_2_O. Since the H/D exchange in the protonated ligand is facile (Figure S4 in the Supporting Information), a reversible C–H activation should lead to the incorporation of deuterium in the final substituted aniline. No deuterium incorporation was observed, supporting an irreversible C–H cleavage.

The reaction mechanism has been investigated by computational methods. A thorough exploration of several routes, with the locations of intermediates and transition states, was carried out using the M06 functional with basis set BS1 and including solvation in the optimizations through the SMD implicit solvent method. However, to obtain accurate energies, additional single-point calculations were performed on all optimized structures employing the domain-based local pair natural orbital coupled cluster approach (DLPNO-CCSD(T)) and an extended basis set (def2-TZVP) (see computational details in the Supporting Information). This method can be considered the state of the art for providing energies of systems of this size, and it has proved to be very effective in obtaining accurate reaction thermodynamics and barrier heights,^22^ including palladium-catalyzed cross-coupling reactions.^23^ All of the Gibbs energies collected in the text have been obtained, adding to the DLPNO-CCSD(T)/def2-TZVp electronic energies thermal and entropic corrections as well as solvation energies (Δ*G*(solv)) obtained at the M06/BS1 level.

We found that the choice of model is crucial to reproduce the basic features of the reaction: C–C coupling vs C–N chemoselectivity, *ortho* regioselectivity, and a turnover-limiting C–H cleavage. The simplest model, consisting of just the palladium, the (bipy-6-OH) ligand, the aryl group, and aniline, fails to account for the experimental results (see Figure S89 in the Supporting Information). To improve it, we enlarged the model, adding to the computational model other species present in the reaction medium, as carbonate and cesium ions. We found the smallest model able to reproduce the prevalence of C–C coupling over C–N coupling must involve, in addition to the cesium carbonate and the continuum representation of the solvent, several explicit DMA solvent molecules (model 4 in Figure S90; see the Supporting Information for inconsistent results with other models). This has been the model employed in all of the calculations.

Figure 1 shows a complete profile for the reaction yielding the *ortho*-arylated product. The rearrangement of the aniline from a N- to a C-bound mode transforms complex **8** into **c1**_***ortho***_. At this point, the deprotonation of the aniline is facile to give **c1NH**_***ortho***_, which undergoes C–H cleavage (via **TS-c1NH**_***ortho***_**-c2NH**_***ortho***_) with a lower energy barrier than that from **c1**_***ortho***_ (**TS-c1**_***ortho***_**-c2**_***ortho***_; Gibbs energy barriers of 12.1 vs 16 kcal mol^–1^, respectively). Therefore, an anionic route on an amido-type intermediate is preferred. A series of proton transfer steps occur on biaryl intermediate **c2NH**_***ortho***_ with the involvement of the pair HCO_3_^–^/CO_3_^2–^, which eventually leads to **c2b**_***ortho***_. In this way the *ortho* CH proton ends up in the carbonate, with a notable stabilization of the system. From **c2b**_***ortho***_ a reductive elimination follows through transition state **TS-c2b**_***ortho***_**-c3**, leading to the arylation product and the Pd(0) intermediate **c4**. In the presence of aniline oxidative addition occurs, leading to **c1**_***ortho***_ that closes the cycle. The conversion of **c1**_***ortho***_ into **8** has a lower energy barrier than the C–H cleavage, and therefore **8** is the plausible resting state of the reaction, outside the catalytic cycle. The equilibrium between **8** and **c1**_***ortho***_ controls the actual concentration of palladium in the catalytic cycle and leads to an energetic span of 29.9 kcal mol^–1^ for the reaction, consistent with the reaction conditions needed. The computed catalytic cycle is represented in Scheme 3.^24^ A microkinetic simulation of this pathway is also consistent with the conversions observed experimentally (see the Supporting Information for details).

**Figure 1:**
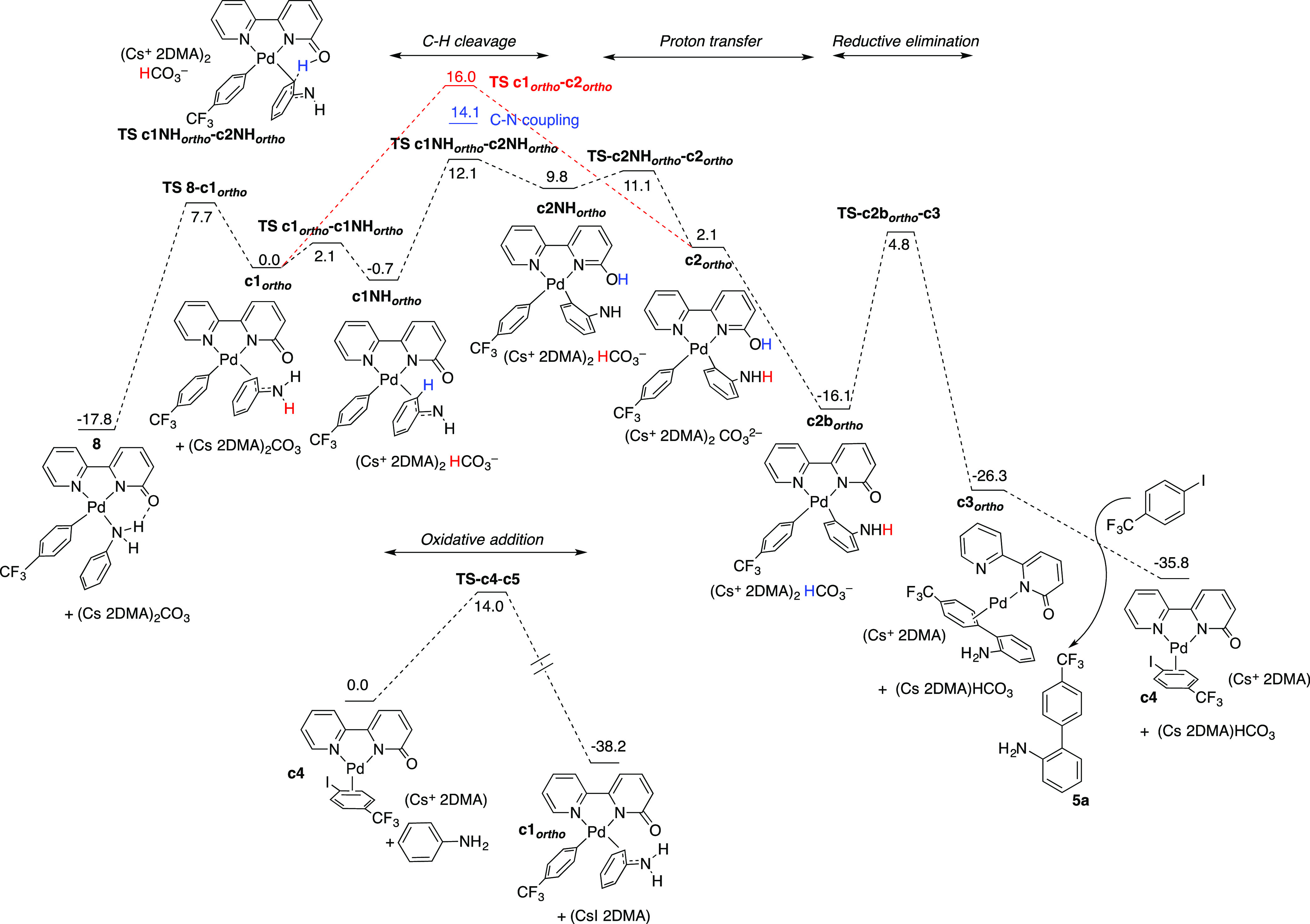
Gibbs energy profile for the Pd-catalyzed arylation of aniline in the *ortho* position, assisted by the ligand bipy-6-O. Energies are given in kcal mol^–1^.

**Scheme 3:**
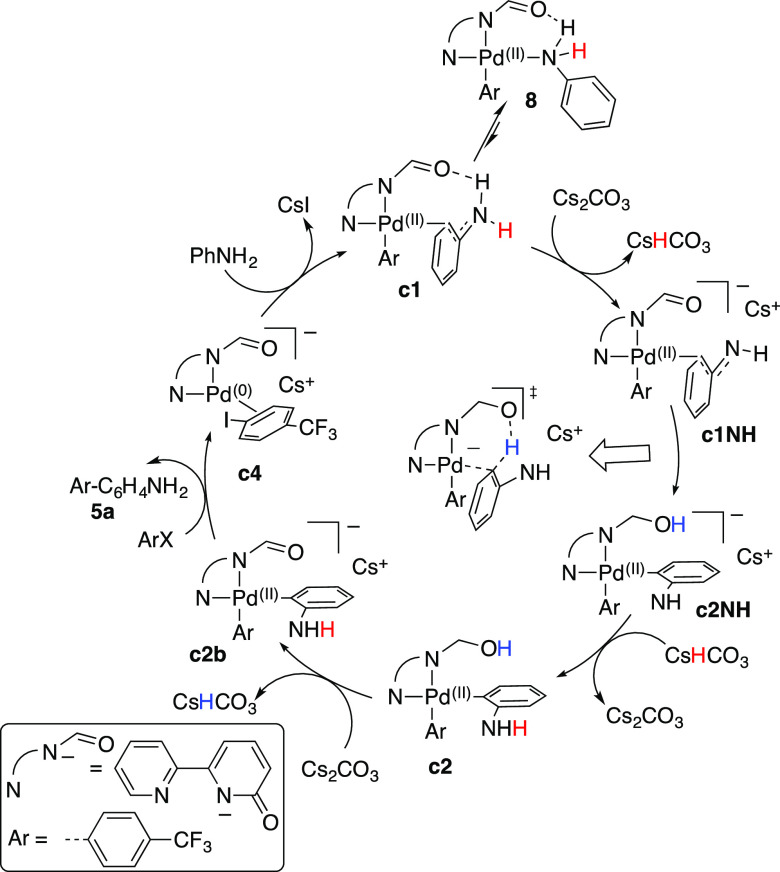
Plausible Catalytic Cycle for the *ortho* Arylation of Aniline

The energy barriers for the other two regioisomers were also calculated. Both the neutral (implying NH_2_ in the aniline) and the anionic (with NH) pathways were computed. The C–H cleavage via the neutral pathway (**c1** to **c2**) is preferred to the anionic pathway (**c1NH** to **c2NH**) for the *meta* CH activation, with Gibbs energy barriers of 19.0 and. 25.7 kcal mol^–1^, respectively, whereas the anionic route is slightly preferred for the *para* CH activation (19.4 vs 19.7 kcal mol^–1^ Gibbs energy barriers; see Figure S92 in the Supporting Information). The lowest energy pathway for each regioisomer is less favored than the ortho arylation by 6.9 kcal mol^–1^ (*meta*) and 7.3 kcal mol^–1^ (*para*). Therefore, the facile deprotonation of the aniline by the carbonate anion, forming an amido type intermediate in these reactions, is important to drive the regioselectivity toward *ortho* arylation. This is possible for primary and secondary anilines but not for the tertiary *N*,*N*-dimethylaniline where the *ortho* isomer was not observed.^25^

As commented below, the C–N coupling route to give the Buchwald–Hartwig amination product was also calculated. The deprotonation of the aniline in complex **8**, is facile and the amido version of complex **8** is found 0.6 kcal mol^–1^ below the neutral form, pointing out the existence of and amino–amido equilibrium in the presence of carbonate in the reaction medium (Figure S93 in the Supporting Information). The aryl-amido reductive elimination barrier is 14.1 kcal mol^–1^, as shown in Figure 1. This value is 2 kcal mol^–1^ higher than the barrier for the C–H *ortho* cleavage in the aniline (12.1 kcal mol^–1^, Figure 1), which makes the *ortho* C–H functionalization preferred, in good agreement with the experimental chemoselectivity. In contrast to bulky phosphine ligands, the ligand bipy-6-OH does not favor the reductive elimination step, which, eventually, is an advantage for chemoselectivity (cf. eqs 1 and 2). Using this enlarged model, the barrier for the CH *ortho* activation is found to be 2.0 kcal mol^–1^ below that of the C–N coupling,

In conclusion, we have shown that unprotected anilines can be selectively arylated in the *ortho* position using the Pd/bipy-6-OH catalyst system. The cooperating role of bipy-6-OH in the C–H cleavage step along with a high discriminating barrier for reductive elimination eliminates the competition of the C–N coupling product (amination).

## Supporting Information Available

(PDF). . The Supporting Information is available free of charge at https://pubs.acs.org/doi/10.1021/acscatal.2c05206.

Experimental details, characterization, kinetic and X-ray structure determination data, and selected spectra (PDF)
Computational details, extended description of the computational results, and Cartesian coordinates and absolute energies of all the optimized structures (PDF)
rystallographic data for complex **7** (CIF)
Crystallographic data for complex **8** (CIF)

## Supplementary Material

cs2c05206_si_002.pdf

cs2c05206_si_003.cif

cs2c05206_si_004.cif

## Abbreviations

CMD: concerted metalation–deprotonation
