# Supplementary material for: Palladium-Catalyzed Ortho C–H Arylation of Unprotected Anilines: Chemo- and Regioselectivity Enabled by the Cooperating Ligand [2,2′-Bipyridin]-6(1H)-one
Source: ACS Catal. 2022 Nov 11;12(23):14527–32. doi: 10.1021/acscatal.2c05206 (PMC9724229; doi:10.1021/acscatal.2c05206)
Supplement: Supplementary file 1 — cs2c05206_si_001.pdf [file cs2c05206_si_001.pdf]

# Supporting information

# Palladium-Catalyzed *Ortho* C–H Arylation of Unprotected Anilines: Chemo- and Regioselectivity Enabled by the Cooperating Ligand [2,2′-bipyridin]-6(1*H*)-one

Cintya Pinilla,[a] Vanesa Salamanca,[a] Agustí Lledós[b]* and Ana C Albéniz[a]*

[a] *IU CINQUIMA/Química Inorgánica, Universidad de Valladolid, 47071 Valladolid, Spain. E-mail: albeniz@uva.es*

[b] *Departament de Química, Universitat Autònoma de Barcelona, Barcelona 08193, Spain. Email: agusti.lledos@uab.cat*

**PART 1**

1. Experimental details.
    1.1. General considerations
    1.2. Synthesis of Palladium complexes
    1.3. Catalytic reactions
        1.3.1. General procedure for the direct arylation of anilines
        1.3.2. Amination of 4-iodobenzotrifluoride with anilines
    1.4. Mechanistic experiments.
        1.4.1. Behavior of complex **1** with aniline
        1.4.2. Thermal decomposition of complex **8** under catalytic conditions
        1.4.3. H-D exchange in complexes **1** and **8** with deuterium oxide
        1.4.4. Determination of equilibrium constants for the coordination of anilines and comparison with the regioselectivity observed
        1.4.5. Probing the intermediacy of Pd(IV) species.
    1.5. Kinetic data
        1.5.1. Determination of the KIE
        1.5.2. Kinetic experiments for the direct arylation of aniline
        1.5.3.
2. Data for X-Ray structure determinations

3. Selected spectra

References

## 1. Experimental details

### 1.1- General considerations.

$^{1}H$, $^{13}C\{^{1}H\}$ and $^{19}F$ NMR spectra were recorded on Agilent MR-500, Agilent MR-400 or Bruker AV-400 spectrometers at the Laboratorio de Técnicas Instrumentales (LTI) of the UVa. Chemical shifts (in δ units, ppm) were referenced to $SiMe_4$ ($^{1}H$ and $^{13}C$) and $CFCl_3$ ($^{19}F$). The spectral data were recorded at 298 K unless otherwise noted. Homonuclear ($^{1}H$-COSY and $^{1}H$-NOESY) and heteronuclear ($^{1}H$-$^{13}C$ HSQC, $^{1}H$-$^{13}C$ HMBC, $^{19}F$-$^{13}C$ HSQC and $^{19}F$-$^{13}C$ HMBC) experiments were used to help with the signal assignments. The GC-MS analyses were performed in a Thermo-Scientific Focus DSQ II GC/MS apparatus. The intensities are reported as percentages relative to the base peak after the corresponding *m/z* value. HRMS analyses were carried out on a Bruker Maxis Impact mass spectrometer at the Laboratorio de Técnicas Instrumentales (LTI) of the UVa. Elemental analyses were carried out in a Carlo Erba 1108 microanalyzer (at the Vigo University, Spain). Infrared spectra were recorded (in the range 4000-200 $cm^{-1}$) on a Perkin-Elmer FT-IR Spectrum Frontier with an ATR diamond accessory.

Solvents were dried using a solvent purification system SPS PS-MD-5 (ether, hexane and $CH_2Cl_2$) or distilled from appropriate drying agents under nitrogen prior to use and stored over 3 Å or 4 Å molecular sieves (toluene, DMA, acetone).

4-Iodobenzotrifluoride, 4-iodotoluene, 4-bromoanisole, cesium carbonate, tetramethylethylenediamine (TMEDA), palladium acetate and all the anilines are commercially available and were purchased from Sigma-Aldrich, Acros Organics, Alfa Aesar or Fluorochem. Commercial reagents were used as received unless otherwise noted. The anilines were distilled or recrystallized and kept under a $N_2$ atmosphere before use.

[Pd(bipy-6-OH)Br($C_6F_5$)] (**1**),[1] [[Pd(bipy-4-OH)Br($C_6F_5$)] (**2**),[1] [Pd(bipy)Br($C_6F_5$)] (**3**),[1,2] [PdBr($C_6F_5$)(phen-2-OH)] (**4**),[3] $(NBu_4)_2[Pd_2(\mu\text{-}Br)_2(C_6F_5)_4]$,[2] $[Pd_2(dba)_3].CHCl_3$[4], [Pd($C_6H_4$-*p*-$CF_3$)I(TMEDA)][5] and [2,2'-bipyridin]-6(1*H*)-one (bipy-6-OH),[1,6] were prepared according to the procedures in the literature.

## 1.2- Synthesis of Palladium complexes.

**[Pd(bipy-6-O)($C_6F_5$)($PhNH_2$)] (7).** [Pd(bipy-6-OH)Br($C_6F_5$)] (95.8 mg, 0.182 mmol) was dissolved in dichloromethane (25 mL). Aniline (50 μL, 0.547 mmol), $Cs_2CO_3$ (60 mg, 0.182 mmol) and $AgBF_4$ (35.5 mg, 0.182 mmol) were added while stirring. The reaction mixture was stirred for 20 h at room temperature. The yellow solution was filtered through kieselgur and the solvent was removed. Cold EtOH was added (15 mL) and the yellow solid was filtered, washed with cold EtOH and air-dried. Yield: 61 mg (62%).

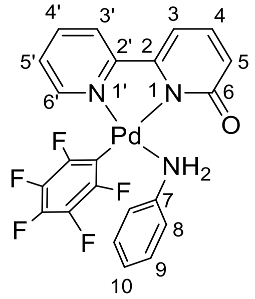


$^1$H NMR (500.13 MHz, δ, $CDCl_3$): 8.65 (br, 2H, $H^{NH2}$), 7.87 (m, 2H, $H^{3'}$, $H^{4'}$), 7.64 (d, J = 6.5 Hz, 1H, $H^{6'}$), 7.45 (dd, J = 7.2, 8.7 Hz, 1H, $H^4$), 7.09 (m, 3H, $H^{5'}$, $H^9$), 7.02 (t, J = 6.2 Hz, 1H, $H^{10}$), 6.94 (d, J = 7.1 Hz, 1H, $H^3$), 6.84 (d, J = 7.5 Hz, 2H, $H^8$), 6.66 (d, J = 8.9 Hz, 1H, $H^5$). $^{13}C\{^1H\}$ NMR (125.78 MHz, δ, $CDCl_3$): 174.1 ($C^6$), 160.7 ($C^{2'}$), 151.4 ($C^{6'}$, $C^2$), 147.5 ($C^{Fortho}$)*, 142.5 ($C^7$), 138.9 ($C^{4'}$), 137.9 ($C^{Fpara}$ *, $C^4$), 136.2 ($C^{Fmeta}$)*, 129 ($C^9$), 124.5 ($C^{10}$, $C^{5'}$),122.9 ($C^5$), 121.6 ($C^{3'}$), 120.8 ($C^8$), 106.7 ($C^3$). $^{19}$F NMR (470.168 MHz, δ, $CDCl_3$): -119.9 (m, 2F, $F_{ortho}$), 158.39 (t, J = 19.1 Hz, 1F, $F_{para}$), 161.27 (m, 2F, $F_{meta}$). Anal. Calculated for $C_{22}H_{14}F_5N_3OPd$ C, 49.13 %; N, 7.81 %; H, 2.62 %; found: C, 49.41 %; N, 7.68 %; H, 2.80 %.

*The chemical shifts of the $C_6F_5$ carbon resonances were determined by HSQC $^{13}$C-$^{19}$F.

**[Pd(bipy-6-O)($C_6H_4$-*p*-$CF_3$)($PhNH_2$)] (8).** Bipy-6-OH (24.1 mg, 0.14 mmol) was dissolved in acetone (25 mL) and $Cs_2CO_3$ (46.0 mg, 0.14 mmol) was added. The suspension was stirred at room temperature for 10 min. Then, aniline (39 μL, 0.42 mmol) and [Pd($C_6H_4$-*p*-$CF_3$)I(TMEDA)] (69 mg, 0.14 mmol) were added. The mixture was stirred at room temperature for 2 h. The yellow suspension was filtered through kieselgur and the solvent was removed. Cold $Et_2O$ (5 mL) was added and the yellow solid was filtered, washed with cold hexane (2 x 2 mL) and air-dried. Yield: 60 mg (83%).

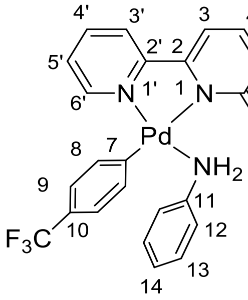


$^{1}H$ NMR (500.13 MHz, δ, $CDCl_3$): 8.29 (br, 2H, $H^{NH2}$), 7.87 (d, J = 8.1 Hz, 1H, $H^{3'}$), 7.79 (td, J = 8.0, 1.5 Hz, 1H, $H^{4'}$), 7.59 (d, J = 5.5 Hz, 1H, $H^{6'}$), 7.43 (dd, J = 8.7, 6.9 Hz, 1H, $H^{4}$), 7.33 (m, J = 7.5 Hz, 2H, $H^{8}$), 7.21(m, J = 7.5 Hz, 2H, $H^{9}$), 7.09 (t, J = 8.0 Hz, 2H, $H^{13}$), 7.04 (t, J = 6.7 Hz, 1H, $H^{5'}$), 6.99 (t, J = 7.2 Hz, 1H, $H^{14}$), 6.93 (d, J = 6.7 Hz, 1H, $H^{3}$), 6.78 (d, J = 7.6 Hz, 2H, $H^{12}$), 6.64 (d, J = 8.9, 1H, $H^{5}$). $^{13}C\{^{1}H\}$ NMR (125.78 MHz, δ, $CDCl_3$): 174.1 ($C^{6}$), 164.2 ($C^{7}$), 160.2 ($C^{2'}$), 151.1 ($C^{6'}$), 150.6 ($C^{2}$), 142.2 ($C^{11}$), 138.2 ($C^{4'}$), 137.9 ($C^{4}$), 134.7 ($C^{8}$), 128.8 ($C^{13}$), 126.2 (q, $^{2}J_{C-F}$ = 32 Hz, $C^{10}$), 125.9 (q, $^{1}J_{C-F}$ = 178 Hz, $CF_3$), 124.3 ($C^{5'}$), 123.9 ($C^{14}$), 123.2 (q, $^{3}J_{C-F}$ = 3.5 Hz, $C^{9}$), 121.7 ($C^{5}$), 121.5 ($C^{3'}$, $C^{12}$), 106.3 ($C^{3}$). $^{19}F$ NMR (470.168 MHz, δ, $CDCl_3$): -61.91 (s, $CF_3$). Anal. Calculated for $C_{22}H_{18}F_3N_3OPd$: C, 53.55 %; N, 8.15 %; H, 3.52 %; found: C, 53.50 %; N, 8.08 %; H, 3.70 %.

**[Pd(bipy-6-O)($C_6F_5$)(PhNHMe)] (9).** [Pd(bipy-6-OH)Br($C_6F_5$)] (106 mg, 0.202 mmol) was dissolved in dichloromethane (25 mL). N-methylaniline (65.5 μL, 0.606 mmol), $Cs_2CO_3$ (65.8 mg, 0.202 mmol) and $AgBF_4$ (39.3 mg, 0.202 mmol) were added to the solution while stirring. The reaction mixture was stirred for 20 h at room temperature. The yellow solution was filtered through kieselgur and the solvent was removed. Cold EtOH (15 mL) was added and the yellow solid was filtered, washed with cold EtOH (5 mL) and air-dried. Yield: 65 mg (58%). The solid is a mixture of complex **9** and a minor isomer (5%) as shown by $^{1}H$ and $^{19}F$ NMR. Only complex **9** was completely characterized.

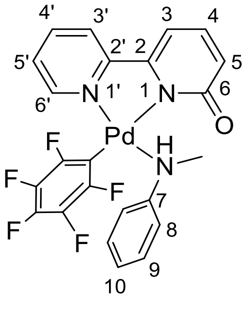

$^1H$ NMR (500.13 MHz, δ, $CDCl_3$): 13.81 (br, H, $H^{NH}$), 7.84 (m, 2H, $H^{4'}$, $H^{3'}$), 7.46 (dd, J = 8.7, 7.1 Hz, 1H, $H^4$), 7.43 (d, J = 5.7 Hz, 1H, $H^{6'}$), 7.17 (t, J = 6.9 Hz, 2H, $H^9$), 7.09 (t, J = 7.2 Hz, 1H, $H^{10}$), 7.04 (t, J = 6.6 Hz, 1H, $H^{5'}$), 6.99 (d, J = 8.3 Hz, 2H, $H^8$), 6.95 (dd, J = 7.2, 1 Hz, 1H, $H^3$), 6.68 (dd, J = 8.7, 1 Hz, 1H, $H^5$), 2.9 (d, J = 5.3 Hz, 3H, $H^{Me}$). $^{13}C\{^1H\}$ NMR (125.78 MHz, δ, $CDCl_3$): 174.2 ($C^6$), 160.4 ($C^{2'}$), 151.4 ($C^2$), 151.3 ($C^{6'}$), 148.5 ($C^7$), 147.7 ($C^{Fortho}$)*, 146.9 ($C^{Fortho}$)*, 138.8 ($C^{4'}$), 138.2 ($C^{Fpara}$)*, 138 ($C^4$), 136.2 ($C^{Fmeta}$)*, 135.9 ($C^{Fmeta}$)*, 128.8 ($C^9$), 124.6 ($C^{10}$), 124.6 ($C^{5'}$), 122.7 ($C^5$), 121.8 ($C^{3'}$), 119.6 ($C^8$), 106.9 ($C^3$), 37.7 ($C^{Me}$). $^{19}F$ NMR (470.168 MHz, δ, $CDCl_3$): -120.01 (m, 1F, $F_{ortho}$), -120.2 (m, 1F, $F_{ortho}$), -158.64 (t, J = 20.1 Hz, 1F, $F_{para}$), -161 (m, 1F, $F_{meta}$), -161.86 (m, 1F, $F_{meta}$). Anal. Calculated for $C_{23}H_{16}F_5N_3OPd$: C, 50.06 %; N, 7.62 %; H, 2.92 %; found: C, 49.77 %; N, 7.22 %; H, 2.82 %.

*The chemical shifts of the $C_6F_5$ carbon resonances were determined by HSQC $^{13}C$-$^{19}F$.

## 1.3- Catalytic reactions

**1.3.1. General procedure for the direct arylation of anilines.**

[Pd(bipy-6-OH)Br($C_6F_5$)] (**1**) (9.0 mg, 0.017 mmol) and cesium carbonate (222 mg, 0.68 mmol) were introduced in a Schlenk flask under a nitrogen atmosphere. Then, 4-iodobenzotrifluoride (51 µL, 0.34 mmol), the corresponding aniline (3.4 mmol) and dry DMA (2.7 mL) were added to the flask. The reaction mixture was stirred at 130 ºC and checked by $^{19}$F NMR of the crude mixture after 2 h, 6 h or 24 h depending on the aniline. When total conversion was observed, the solvent was evaporated in vacuo and the organic product was extracted with a mixture of *n*-hexane (3 mL) and ethyl acetate (0.3 mL). The extract was filtered through kieselgur and evaporated to dryness. The ratio of isomers was checked again by $^{19}$F NMR and GC-MS. The product was purified by column chromatography using silica gel and a mixture of *n*-hexane:EtOAc = 9:1 as eluent.

The reaction with aryl halides other than 4-iodobenzotrifluoride were carried out in the same way. Those products that could not be completely purified by column chromatography were subjected to preparative TLC (*n*-hexane:EtOAc = 9:1). The yields and characterization data for the products obtained are collected below. For known compounds the spectral data conforms to those in the literature (references are given).

**Aniline** as arene. ArX = ***p*-$CF_3C_6H_4I$ (5a)**. The product was obtained as a yellow oil, mixture of three isomers in a ratio o:m:p = 25:1:1. Yield: 0.061 g (76 %). The major isomer (*ortho*) was separated by column chromatography (0.041 g, 51 % yield). The characterization of 4’-(trifluoromethyl)-[1,1’-biphenyl]-2-amine, 4’-(trifluoromethyl)-[1,1’-biphenyl]-4-amine and 4’-(trifluoromethyl)-[1,1’-biphenyl]-3-amine has been reported before.[7]

***N*-methylaniline** as arene. ArX = ***p*-$CF_3C_6H_4I$ (5l)** The product was obtained as a yellow oil, mixture of three isomers in the ratio o:m:p = 18:2:1. Yield: 0.068 g (79 %). The major isomer (*ortho*) was separated by column chromatography (0.05 g, 58 % yield).

<u>*N*-Methyl-4’-(trifluoromethyl)[1,1’-biphenyl]-2-amine:</u>[8] $^1$H NMR (500.13 MHz, δ, $CDCl_3$): 7.70 (m, 2H, $H^2$, $H^6$), 7.56 (m, 2H, $H^3$, $H^5$), 7.31 (ddd, J = 8.3, 7.4, 1.6 Hz, 1H,

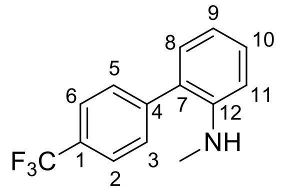

$H^{10}$), 7.08 (dd, J = 7.4, 1.5 Hz, 1H, $H^{8}$), 6.80 (td, J = 7.4, 1.6 Hz, 1H, $H^{9}$), 6.73 (d, J = 8.3 Hz, 1H, $H^{11}$), 3.86 (br, 1H, $H^{NH}$), 2.81 (s, 3H, Me). $^{13}C\{^{1}H\}$ NMR (125.78 MHz, δ, $CDCl_3$): 145.9 ($C^{12}$), 143.3 ($C^{4}$), 129.9 ($C^{8}$), 129.8 ($C^{3}$, $C^{5}$), 129.5 ($C^{10}$), 129.3 (q, $^{2}J_{C-F}$ = 33.6 Hz, $C^{1}$), 126.0 ($C^{7}$), 125.8 (q, $^{3}J_{C-F}$ = 3.8 Hz, $C^{2}$, $C^{6}$), 124.2 (q, $^{1}J_{C-F}$ = 272 Hz, $CF_3$), 117.1 ($C^{9}$), 110.2 ($C^{11}$), 30.8 (Me). $^{19}F$ NMR (470.168 MHz, δ, $CDCl_3$): -62.51 (s, $CF_3$). MS (EI, 70 eV): m/z (%) 251 (50) [$M^{+}$], 235 (10), 202 (7), 181 (35), 152 (10).

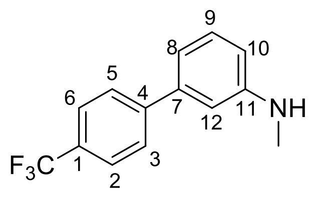

<u>*N*-Methyl-4'-(trifluoromethyl)[1,1'-biphenyl]-3-amine:[9]</u> $^{1}H$ NMR (500.13 MHz, δ, $CDCl_3$): 7.67 (br, 4H, $H^{2}$, $H^{3}$, $H^{5}$, $H^{6}$), 7.28 (t, J = 7.7 Hz, 1H, $H^{9}$), 6.93 (d, J = 7.5 Hz, 1H, $H^{8}$), 6.79 (br, 1H, $H^{12}$), 6.66 (d, J = 7.9 Hz, 1H, $H^{10}$), 3.87 (br, 1H, $H^{NH}$), 2.90 (s, 3H, Me). $^{13}C\{^{1}H\}$ NMR (125.78 MHz, δ, $CDCl_3$): 129.9 ($C^{9}$), 127.5 ($C^{3}$, $C^{5}$), 125.6 ($C^{2}$, $C^{6}$), 116.4 ($C^{8}$), 112.5 ($C^{10}$), 110.9 ($C^{12}$), 30.4 (Me). $^{19}F$ NMR (470.168 MHz, δ, $CDCl_3$): -62.37 (s, $CF_3$). MS (EI, 70 eV): m/z (%) 251 (100) [$M^{+}$], 201 (15), 152 (20).

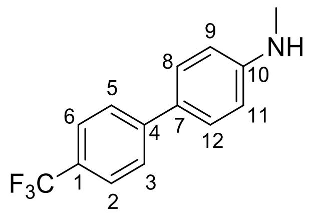

<u>*N*-Methyl-4'-(trifluoromethyl)[1,1'-biphenyl]-4-amine:[10]</u> $^{1}H$ NMR (500.13 MHz, δ, $CDCl_3$): 7.63 (br, 4H, $H^{2}$, $H^{3}$, $H^{5}$, $H^{6}$), 7.47 (d, J = 8.6 Hz, 2H, $H^{8}$, $H^{12}$), 6.70 (d, J = 8.8 Hz, 2H, $H^{9}$, $H^{11}$), 3.87 (br, 1H, $H^{NH}$), 2.90 (s, 3H, Me). $^{13}C\{^{1}H\}$ NMR (125.78 MHz, δ, $CDCl_3$): 149.3 ($C^{10}$), 144.6 ($C^{4}$), 128.1 ($C^{8}$, $C^{12}$), 126.2 ($C^{3}$, $C^{5}$), 125.6 ($C^{2}$, $C^{6}$), 123.1 ($CF_3$), 112.9 ($C^{9}$, $C^{11}$), 30.8 (Me). $^{19}F$ NMR (470.168 MHz, δ, $CDCl_3$): -62.22 (s, $CF_3$). MS (EI, 70 eV): m/z (%) 251(100) [$M^{+}$], 235 (10), 181(40).

***N*-isopropylaniline** as arene. ArX = ***p*-$CF_3C_6H_4I$** (**5m**). The product was obtained as a yellow oil, mixture of three isomers in the ratio o:m:p = 12:3:1. Yield: 0.062 g (65 %). The major product (*ortho*) was separated by column chromatography (0.048 g, 50 % yield).

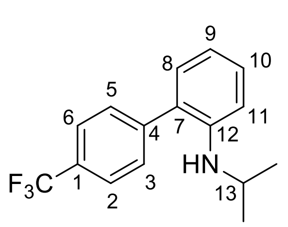

<u>*N*-Isopropyl-4'-(trifluoromethyl)[1,1'-biphenyl]-2-amine:</u> $^{1}H$ NMR (500.13 MHz, δ, $CDCl_3$): 7.70 (m, 2H, $H^{2}$, $H^{6}$), 7.54 (m, 2H, $H^{3}$, $H^{5}$), 7.26 (t, J = 7.39 Hz, 1H, $H^{10}$), 7.05 (d, J = 7.34 Hz, 1H, $H^{8}$), 6.75 (m, 3H, $H^{9}$, $H^{11}$), 3.63 (br, 2H, $H^{NH}$, $H^{13}$), 1.15 (d, J = 5.89 Hz, 6H, Me). $^{13}C\{^{1}H\}$ NMR (125.78 MHz, δ, $CDCl_3$): 144.1 ($C^{12}$), 143.5 ($C^{4}$),

130.4 ($C^8$), 129.7 ($C^3$, $C^5$), 129.4 ($C^{10}$), 129.2 (q, $^2J_{C\text{-}F}$ = 34 Hz, $C^1$), 126.0 ($C^7$), 125.8 (q, $^3J_{C\text{-}F}$ = 4 Hz, $C^2$, $C^6$), 124.2 (q, $^1J_{C\text{-}F}$ = 273 Hz, $CF_3$), 116.7 ($C^9$), 111.4 ($C^{11}$), 44.2 ($C^{13}$), 22.9 (Me). $^{19}$F NMR (470.168 MHz, δ, $CDCl_3$): -62.49 (s, $CF_3$). IR (neat) $cm^{-1}$: 3422, 2968, 1504, 1320, 1119. HRMS (ESI-TOF): Calcd. for $C_{16}H_{17}F_3N$ $(M+H)^+$ 280.1308, found 280.1313. Rf ($SiO_2$, *n*-hexane:EtOAc = 8:2) = 0.72.

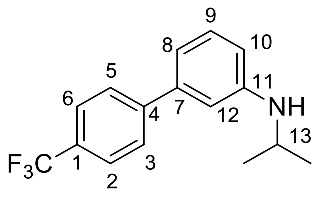


*N*-Isopropyl-4'-(trifluoromethyl)[1,1'-biphenyl]-3-amine: $^1$H NMR (500.13 MHz, δ, $CDCl_3$): 7.66 (m, 4H, $H^2$, $H^3$, $H^5$, $H^6$), 7.25 (t, J = 8.0 Hz, 1H, $H^9$), 6.89 (d, J = 7.9 Hz, 1H, $H^8$), 6.77 (s, 1H, $H^{12}$), 6.63 (d, J = 7.9 Hz, 1H, $H^{10}$), 3.68 (br, 2H, $H^{NH}$, $H^{13}$), 1.25 (d, J = 6.1 Hz, 6H, Me). $^{19}$F NMR (470.168 MHz, δ, $CDCl_3$): -62.38 (s, $CF_3$). MS (EI, 70 eV): m/z (%) 279 (40) [$M^+$], 264 (100), 248 (20).

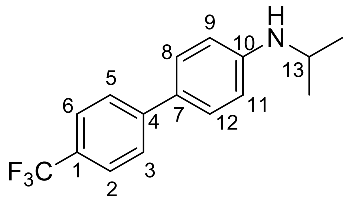


*N*-Isopropyl-4'-(trifluoromethyl)[1,1'-biphenyl]-4-amine: $^1$H NMR (500.13 MHz, δ, $CDCl_3$): 7.62 (m, 4H, $H^2$, $H^3$, $H^5$, $H^6$), 7.44 (m, J = 8.5 Hz, 2H, $H^8$, $H^{12}$), 6.6 (m, J = 8.4 Hz, 2H, $H^9$, $H^{11}$), 3.68 (br, 2H, $H^{NH}$, $H^{13}$), 1.25 (d, J = 6.0 Hz, 6H, Me). $^{13}C\{^1H\}$ NMR (125.78 MHz, δ, $CDCl_3$): 147.4 ($C^{10}$), 144.7 ($C^4$), 128.1 ($C^8$, $C^{12}$), 128.0 ($C^7$), 126.1 ($C^3$, $C^5$), 125.8 ($C^1$), 125.6 ($C^2$, $C^6$), 123.2 ($CF_3$)*, 113.5 ($C^9$, $C^{11}$), 44.7 ($C^{13}$), 22.8 (Me). $^{19}$F NMR (470.168 MHz, δ, $CDCl_3$): -62.23 (s, $CF_3$). MS (EI, 70 eV): m/z (%) 279 (40) [$M^+$], 264 (100), 248 (20). *The chemical shift was determined by HSQC $^{13}$C-$^{19}$F.

***N*, *N*-dimethylaniline** as arene. ArX = ***p*-$CF_3C_6H_4I$** (**5n**). The product was obtained as a yellow oil, mixture of two isomers in the ratio m:p = 1.2:1. Yield: 0.039 g (43%). The products could not be completely separated by column chromatography.

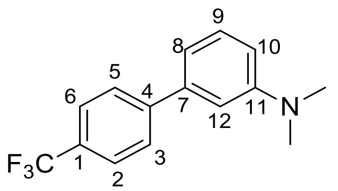


*N*, *N*-Dimethyl-4'-(trifluoromethyl)[1,1'-biphenyl]-3-amine:[11] $^1$H NMR (500.13 MHz, δ, $CDCl_3$): 7.68 (m, 4H, $H^2$, $H^3$, $H^5$, $H^6$), 7.33 (t, J = 7.9 Hz, 1H, $H^9$), 6.93 (d, J = 7.3 Hz, 1H, $H^8$), 6.89 (m, 1H, $H^{12}$), 6.79 (d, J = 9.1 Hz, 1H, $H^{10}$), 3.02 (s, 6H, Me). $^{13}C\{^1H\}$ NMR (125.78 MHz, δ, $CDCl_3$): 150.9 ($C^{11}$), 145.9 ($C^4$), 140.8 ($C^7$), 129.5 ($C^9$), 129.1 (q, $^2J_{C\text{-}F}$ = 31 Hz, $C^1$), 127.5 ($C^3$, $C^5$), 125.5 (q, $^3J_{C\text{-}F}$ = 3.8 Hz, $C^2$, $C^6$), 124.3 (q, $^1J_{C\text{-}F}$ = 271 Hz, $CF_3$), 115.8 ($C^8$), 112.3 ($C^{10}$), 111.1 ($C^{12}$), 40.1 (Me). $^{19}$F NMR (470.168 MHz, δ, $CDCl_3$): -62.37 (s, $CF_3$). MS (EI, 70 eV): m/z (%) 264 (100) [$M^+$], 221 (10), 201 (20), 152 (25).

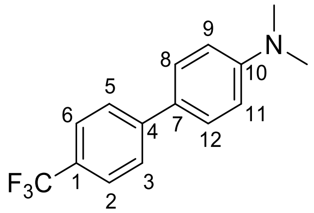


<u>*N, N*-Dimethyl-4'-(trifluoromethyl)[1,1'-biphenyl]-4-amine:</u>[11] $^1$H NMR (500.13 MHz, δ, $CDCl_3$): 7.63 (m, 4H, $H^2$, $H^3$, $H^5$, $H^6$), 7.56 (d, J = 8.7 Hz, 2H, $H^8$, $H^{12}$), 7.28 (d, J = 8.9 Hz, $H^9$, $H^{11}$), 2.84 (s, 6H, Me). $^{13}C\{^1H\}$ NMR (125.78 MHz, δ, $CDCl_3$): 150.5 ($C^{10}$), 144.6 ($C^4$), 140.8 ($C^7$), 128 (q, J = 32.9 Hz, $C^1$), 127.9 ($C^8$, $C^{12}$), 126.2 ($C^3$, $C^5$), 125.5 (q, J = 2.7 Hz, $C^2$, $C^6$), 124.4 ($CF_3$)*, 112.8 ($C^9$, $C^{11}$), 40.4 (Me). $^{19}$F NMR (470.168 MHz, δ, $CDCl_3$): -62.23 (s, $CF_3$). MS (EI, 70 eV): m/z (%) 265 (20) [$M^+$], 251 (50), 201 (15), 152 (15). *The chemical shift was determined by HSQC $^{13}C$-$^{19}F$.

**2-Toluidine** as arene. ArX = ***p*-$CF_3C_6H_4I$** (**5b**). The product was obtained as a yellow oil. Yield: 0.048 g (56 %). Rf ($SiO_2$, *n*-hexane:EtOAc = 8:2) = 0.42.

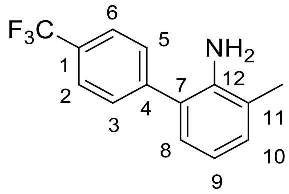


<u>3-Methyl-4'-(trifluoromethyl)-[1,1'-biphenyl]-2-amine:</u> $^1$H NMR (500.13 MHz, δ, $CDCl_3$): 7.71 (d, J = 8.3 Hz, 2H, $H^2$, $H^6$), 7.59 (d, J = 8.3 Hz, 2H, $H^3$, $H^5$), 7.10 (d, J = 7.4 Hz, 1H, $H^{10}$), 6.98 (d, J = 7.5 Hz, 1H, $H^8$), 6.78 (t, J = 7.7 Hz, 1H, $H^9$), 3.68 (br, 2H, $H^{NH2}$), 2.23 (s, 3H, Me). $^{13}C\{^1H\}$ NMR (125.78 MHz, δ, $CDCl_3$): 143.7 ($C^4$), 141.3 ($C^{12}$), 130.4 ($C^{10}$), 129.6 ($C^3$, $C^5$), 129.3 (q, $^2J_{C\text{-}F}$ = 32 Hz, $C^1$), 128.2 ($C^8$), 125.7 (q, $^3J_{C\text{-}F}$ = 3.8 Hz, $C^2$, $C^6$, $C^7$), 124.0 (q, $^1J_{C\text{-}F}$ = 273 Hz, $CF_3$), 122.8 ($C^{11}$), 118.4 ($C^9$), 17.9 (Me). $^{19}$F NMR (470.168 MHz, δ, $CDCl_3$): -62.52 (s, $CF_3$). IR (neat) $cm^{-1}$: 3388, 2922, 1609, 1464, 1322, 1118. HRMS (ESI-TOF): Calcd. for $C_{14}H_{13}F_3N$ $[M+H]^+$ 252.0995, found 252.1001. The regiochemistry of the product was unequivocally determined by the observation of a positive NOE effect $H^{NH2}$-$H^{3,5}$ in a NOESY NMR experiment.

**4-Toluidine** as arene. ArX = ***p*-$CF_3C_6H_4I$** (**5c**). The product is obtained as a yellow oil. Yield: 0.052 g (61 %).

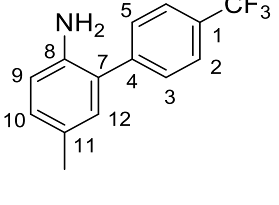


<u>5-Methyl-4'-(trifluoromethyl)-[1,1'-biphenyl]-2-amine:</u>[12] $^1$H NMR (500.13 MHz, δ, $CDCl_3$): 7.70 (d, J = 8.3 Hz, 2H, $H^2$, $H^6$), 7.60 (d, J = 8.3 Hz, 2H, $H^3$, $H^5$), 7.02 (d, J = 8.1 Hz, 1H, $H^{10}$), 6.94 (s, 1H, $H^{12}$), 6.72 (d, J = 8.1 Hz, 1H, $H^9$), 3.61 (br, 2H, $H^{NH2}$), 2.29 (s, 3H, Me). $^{13}C\{^1H\}$ NMR (125.78 MHz, δ, $CDCl_3$): 143.5 ($C^4$), 140.8 ($C^8$), 130.8 ($C^{12}$), 129.8 ($C^{10}$), 129.4 ($C^3$, $C^5$), 129.2 (q, $^2J_{C\text{-}F}$ = 32 Hz, $C^1$), 128.2 ($C^{11}$), 126.1 ($C^7$), 125.7 (q, $^3J_{C\text{-}F}$ = 3.7 Hz, $C^2$, $C^6$), 124.3 (q, $^1J_{C\text{-}F}$ =

272 Hz, $CF_3$), 116.1 ($C^9$), 20.4 (Me). $^{19}$F NMR (470.168 MHz, δ, $CDCl_3$): -62.6 (s, $CF_3$). MS (EI, 70 eV): m/z (%) 251 (100) [$M^+$], 180 (20), 106 (20). The regiochemistry of the product was unequivocally determined by the observation of a positive NOE effect between $H^{12}$-$H^{Me}$, $H^{10}$-$H^{Me}$ and $H^{NH2}$-$H^{3,5}$ in a NOESY NMR experiment.

**2-Phenylaniline** as arene. ArX = ***p*-$CF_3C_6H_4I$ (5d)**. The product is obtained as a yellow solid. m.p.: 73.5-74.8 ºC. Yield: 0.060 g (56 %). Rf ($SiO_2$, *n*-hexane:EtOAc = 8:2) = 0.56.

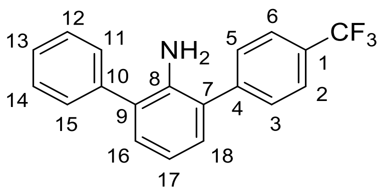


3-Phenyl-4'-(trifluoromethyl)-[1,1'-biphenyl]-2-amine: $^1$H NMR (500.13 MHz, δ, $CDCl_3$): 7.71 (d, J = 8.1 Hz, 2H, $H^2$, $H^6$), 7.64 (d, J = 8.1 Hz, 2H, $H^3$, $H^5$), 7.48 (m, 4H, $H^{11}$, $H^{12}$, $H^{14}$, $H^{15}$), 7.37 (tt, J = 6.6, 1.5 Hz, 1H, $H^{13}$), 7.15 (dd, J = 7.7, 1.5 Hz, 1H, $H^{16}$), 7.10 (dd, J = 7.7, 1.5 Hz, 1H, $H^{18}$), 6.90 (t, J = 7.7 Hz, 1H, $H^{17}$), 3.81 (br, 2H, $H^{NH2}$). $^{13}C\{^1H\}$ NMR (125.78 MHz, δ, $CDCl_3$): 143.6 ($C^4$), 140.6 ($C^8$), 139.4 ($C^{10}$), 130.4 ($C^{16}$), 129.7 (q, $^2J_{C\text{-}F}$ = 31 Hz, $C^1$, $C^3$, $C^5$, $C^{18}$), 129.3 ($C^{11}$, $C^{15}$), 128.9 ($C^{12}$, $C^{14}$), 128.3 ($C^9$), 127.4 ($C^{13}$), 126.4 ($C^7$), 125.8 (q, $^3J_{C\text{-}F}$ = 3.5 Hz, $C^2$, $C^6$), 124.1 (q, $^1J_{C\text{-}F}$ = 274 Hz, $CF_3$), 118.4 ($C^{17}$). $^{19}$F NMR (470.168 MHz, δ, $CDCl_3$): -62.52 (s, $CF_3$). IR (neat) $cm^{-1}$: 3463, 3370, 3065, 1601, 1435, 1318, 1106. HRMS (ESI-TOF): Calcd. for $C_{19}H_{15}F_3N$ $[M+H]^+$ 314.1151, found 314.1142. The regiochemistry of the product was unequivocally determined by the observation of a positive NOE effect $H^{NH2}$-$H^{11,15}$, $H^{NH2}$-$H^{3,5}$ in a NOESY NMR experiment.

**4-Phenylaniline** as arene. ArX = ***p*-$CF_3C_6H_4I$ (5e)**. The product is obtained as a yellow oil. Yield: 0.066g (62 %). Rf ($SiO_2$, *n*-hexane:EtOAc = 8:2) = 0.38.

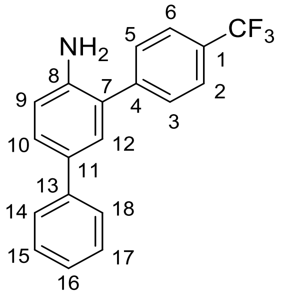


5-Phenyl-4'-(trifluoromethyl)-[1,1'-biphenyl]-2-amine: $^1$H NMR (500.13 MHz, δ, $CDCl_3$): 7.74 (d, J = 7.9 Hz, 2H, $H^2$, $H^6$), 7.66 (d, J = 7.9 Hz, 2H, $H^3$, $H^5$), 7.57 (d, J = 7.2 Hz, 2H, $H^{14}$, $H^{18}$), 7.47 (dd, J = 8.3, 2.3 Hz, 1H, $H^{10}$), 7.40 (t, J = 7.2 Hz, 2H, $H^{15}$, $H^{17}$), 7.38 (d, J = 2.3 Hz, 1H, $H^{12}$), 7.29 (t, J = 7.2 Hz, 1H, $H^{16}$), 6.86 (d, J = 8.3 Hz, 1H, $H^9$), 3.97 (br, 2H, $H^{NH2}$). $^{13}C\{^1H\}$ NMR (125.78 MHz, δ, $CDCl_3$): 143.2 ($C^4$), 142.7 ($C^8$), 140.7 ($C^{13}$), 132.0 ($C^{11}$), 129.5 ($C^3$, $C^5$), 129.4 (q, $^2J_{C\text{-}F}$ = 32 Hz, $C^1$), 129.0 ($C^{12}$), 128.7 ($C^{15}$, $C^{17}$), 127.9 ($C^{10}$), 126.5 ($C^{16}$), 126.4 ($C^7$, $C^{14}$, $C^{18}$), 125.8 (q, $^3J_{C\text{-}F}$ = 3.6 Hz, $C^2$, $C^6$),

124.1 (q, $^1J_{C-F}$ = 274 Hz, $CF_3$), 116.4 ($C^9$). $^{19}F$ NMR (470.168 MHz, δ, $CDCl_3$): -62.56 (s, $CF_3$). IR (neat) $cm^{-1}$: 3485, 3390, 2922, 1615, 1483, 1323, 1158. HRMS (ESI-TOF): Calcd. for $C_{19}H_{15}F_3N$ $[M+H]^+$ 314.1151, found 314.1157. The regiochemistry of the product was unequivocally determined by the observation of a positive NOE effect $H^{NH2}$-$H^9$, $H^{NH2}$-$H^{3,5}$ in a NOESY NMR experiment.

**4-Methoxyaniline** as arene. ArX = ***p*-CF₃C₆H₄I (5f).** The product is obtained as a yellow oil. It was completely characterized. Yield: 0.055 g (60 %). Rf ($SiO_2$, *n*-hexane:EtOAc = 8:2) = 0.27.

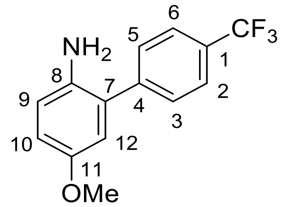


5-Methoxy-4'-(trifluoromethyl)-[1,1'-biphenyl]-2-amine: $^1H$ NMR (500.13 MHz, δ, $CDCl_3$): 7.68 (d, J = 8.0 Hz, 2H, $H^2$, $H^6$), 7.61 (d, J = 8.0 Hz, 2H, $H^3$, $H^5$), 6.82 (d, J = 1.5 Hz, 2H, $H^9$, $H^{10}$), 6.73 (d, J = 1.5 Hz, 1H, $H^{12}$), 4.43 (br, 2H, $H^{NH2}$), 3.78 (s, 3H, $H^{OMe}$). $^{13}C\{^1H\}$ NMR (125.78 MHz, δ, $CDCl_3$): 153.6 ($C^{11}$), 142.9 $C^4$), 135.2 ($C^8$), 129.6 (q, $^2J_{C-F}$ = 33 Hz, $C^1$), 129.5 ($C^3$, $C^5$), 128.2 ($C^7$), 125.7 (q, $^3J_{C-F}$ = 3.6 Hz, $C^2$, $C^6$), 124.1 (q, $^1J_{C-F}$ = 274 Hz, $CF_3$), 118.1 ($C^9$), 115.7 ($C^{12}$), 115.1 ($C^{10}$), 55.8 (OMe). $^{19}F$ NMR (470.168 MHz, δ, $CDCl_3$): -62.55 (s, $CF_3$). IR (neat) $cm^{-1}$: 3362, 2937, 1615, 1497, 1321, 1117. HRMS (ESI-TOF): Calcd. for $C_{14}H_{13}F_3NO$ $[M+H]^+$ 268.0944, found 268.0949. The regiochemistry of the product was unequivocally determined by the observation of a positive NOE effect $H^{Ome}$-$H^{10}$, $H^{Ome}$-$H^{12}$, $H^{3,5}$-$H^{12}$ in a NOESY NMR experiment.

**4-Chloroaniline** as arene. ArX = ***p*-CF₃C₆H₄I** (**5g**). The product is obtained as a yellow oil. Yield: 0.058 g (63 %). Rf ($SiO_2$, *n*-hexane:EtOAc = 8:2) = 0.33.

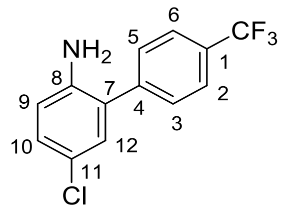


5-Chloro-4'-(trifluoromethyl)-[1,1'-biphenyl]-2-amine: $^1H$ NMR (500.13 MHz, δ, $CDCl_3$): 7.71 (m, J = 8.0 Hz, 2H, $H^2$, $H^6$), 7.55 (m, J = 8.0 Hz, 2H, $H^3$, $H^5$), 7.15 (dd, J = 8.4, 2.5 Hz, 1H, $H^{10}$), 7.09 (d, J = 2.5 Hz, 1H, $H^{12}$), 6.70 (d, J = 8.5 Hz, 1H, $H^9$), 3.73 (br, 2H, $H^{NH2}$). $^{13}C\{^1H\}$ NMR (125.78 MHz, δ, $CDCl_3$): 142 ($C^8$, $C^4$), 129.9 (q, $^2J_{C-F}$ = 32 Hz, $C^1$), 129.8 ($C^{12}$), 129.3 ($C^3$, $C^5$), 128.9 ($C^{10}$), 127.2 ($C^7$), 125.9 (q, $^3J_{C-F}$ = 3.7 Hz, $C^2$, $C^6$), 124.0 (q, $^1J_{C-F}$ = 270 Hz, $CF_3$), 123.4 ($C^{11}$), 116.9 ($C^9$). $^{19}F$ NMR (470.168 MHz, δ, $CDCl_3$): -62.62 (s, $CF_3$). IR (neat) $cm^{-1}$: 3474, 3383, 2926, 1617, 1486, 1321, 1106. HRMS (ESI-TOF): Calcd. for $C_{13}H_{10}ClF_3N$ $[M+H]^+$ 272.0448, found 272.0452. The regiochemistry of the product was

unequivocally determined by the observation of a positive NOE effect $H^{NH2}$-$H^9$, $H^{NH2}$-$H^{3,5}$ in a NOESY NMR experiment.

**2-Trifluoromethylaniline** as arene. ArX = ***p*-CF$_3$C$_6$H$_4$I** (**5h**). The product is obtained as a yellow oil. Yield: 0.066 g (64 %). $R_f$ ($SiO_2$, *n*-hexane:EtOAc = 8:2) = 0.63.

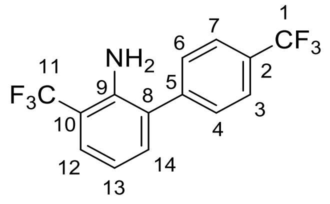


<u>3-Trifluoromethyl-4'-(trifluoromethyl)-[1,1'-biphenyl]-2-amine:</u> $^1$H NMR (500.13 MHz, δ, $CDCl_3$): 7.74 (d, J = 8.3 Hz, 2H, $H^3$, $H^7$), 7.56 (d, J = 8.3 Hz, 2H, $H^4$, $H^6$), 7.49 (d, J = 7.9 Hz, 1H, $H^{12}$), 7.29 (d, J = 7.9 Hz, 1H, $H^{14}$), 6.86 (t, J = 7.9 Hz, 1H, $H^{13}$), 4.22 (br, 1H, $H^{NH2}$). $^{13}C\{^1H\}$ NMR (125.78 MHz, δ, $CDCl_3$): 141.9 ($C^5$), 141.6 ($C^9$), 133.8 ($C^{14}$), 130.2 (q, $^2J_{C\text{-}F}$ = 32 Hz, $C^2$), 129.7 ($C^4$, $C^6$), 127.7 ($C^8$), 126.6 (q, $^3J_{C\text{-}F}$ = 5.2 Hz, $C^{12}$), 126.1 (q, $^3J_{C\text{-}F}$ = 3.8 Hz, $C^3$, $C^7$), 124.7 (q, $^1J_{C\text{-}F}$ = 273 Hz, $C^{11}$), 123.9 (q, $^1J_{C\text{-}F}$ = 272 Hz, $C^1$), 117.3 ($C^{13}$), 114.2 (q, $^2J$ = 30 Hz, $C^{10}$). $^{19}$F NMR (470.168 MHz, δ, $CDCl_3$): -62.67 (s, 3F, $F^1$), -62.92 (s, 3F, $F^{11}$). IR (neat) $cm^{-1}$: 3517, 3423, 2927, 1619, 1461, 1321, 1067. HRMS (ESI-TOF): Calcd. for $C_{14}H_8F_6N$ $[M-H]^-$ 304.0566, found 304.0560. The regiochemistry of the product was unequivocally determined by the observation of a positive NOE effect $H^{NH2}$-$H^{4,6}$ in a NOESY NMR experiment.

**4-Trifluoromethylaniline** as arene. ArX = ***p*-CF$_3$C$_6$H$_4$I** (**5i**). The product is obtained as a yellow oil. $R_f$ ($SiO_2$, *n*-hexane:EtOAc = 8:2) = 0.36. Yield: 0.058 g (56 %).

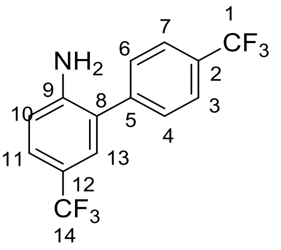


<u>5-Trifluoromethyl-4'-(trifluoromethyl)-[1,1'-biphenyl]-2-amine:</u> $^1$H NMR (500.13 MHz, δ, $CDCl_3$): 7.73 (d, J = 8.1 Hz, 2H, $H^3$, $H^7$), 7.57 (d, J = 8.1 Hz, 2H, $H^4$, $H^6$), 7.41 (dd, J = 8.6, 2.0 Hz, 1H, $H^{11}$), 7.35 (d, J = 2.0 Hz, 1H, $H^{13}$), 6.80 (d, J = 8.6 Hz, 1H, $H^{10}$), 4.10 (br, 2H, $H^{NH2}$). $^{13}C\{^1H\}$ NMR (125.78 MHz, δ, $CDCl_3$): 146.4 ($C^9$), 141.8 ($C^5$), 130.1 (q, $^2J_{C\text{-}F}$ = 32 Hz, $C^2$), 129.4 ($C^4$, $C^6$), 127.5 (q, $^3J_{C\text{-}F}$ = 3.7 Hz, $C^{13}$), 126.3 (q, $^3J_{C\text{-}F}$ = 3.7 Hz, $C^{11}$), 126.0 (q, $^3J_{C\text{-}F}$ = 3.1 Hz, $C^3$, $C^7$), 125.3 ($C^8$), 124.6 (q, $^1J_{C\text{-}F}$ = 271 Hz, $C^{14}$), 124.1 (q, $^1J_{C\text{-}F}$ = 273 Hz, $C^1$), 120.7 (q, $^2J_{C\text{-}F}$ = 34 Hz, $C^{12}$), 115.3 ($C^{10}$). $^{19}$F NMR (470.168 MHz, δ, $CDCl_3$): -61.30 (s, 3F, $F^{14}$), -62.67 (s, 3F, $F^1$). IR (neat) $cm^{-1}$: 3500, 3410, 2929, 1625, 1320, 1103. HRMS (ESI-TOF): Calcd. for $C_{14}H_{10}F_6N$ $[M+H]^+$ 306.0712, found 306.0712. The regiochemistry of the product was unequivocally determined by the observation of a positive NOE effect $H^{NH2}$-$H^{4,6}$, $H^{NH2}$-$H^{10}$ in a NOESY NMR experiment.

**3,5-Dimethylaniline** as arene. ArX = ***p*-CF$_3$C$_6$H$_4$I** (**5j**). The product is obtained as a yellow oil. R$_f$ (SiO$_2$, *n*-hexane:EtOAc = 8:2) = 0.5. Yield: 0.040 g (44 %).

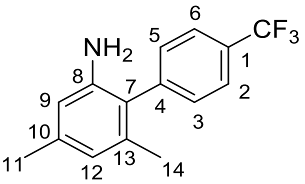


4,6-Dimethyl-4'-(trifluoromethyl)-[1,1'-biphenyl]-2-amine: $^1$H NMR (500.13 MHz, δ, CDCl$_3$): 7.72 (d, 2H, J = 8.4 Hz, H$^2$, H$^6$), 7.38 (d, 2H, J = 8.4 Hz, H$^3$, H$^5$), 6.55 (s, 1H, H$^{12}$), 6.48 (s, 1H, H$^9$), 3.35 (br, 2H, H$^{NH2}$), 2.27 (s, 3H, H$^{11}$), 1.96 (s, 3H, H$^{14}$). $^{13}$C{$^1$H} NMR (125.78 MHz, δ, CDCl$_3$): 143.6 (C$^8$), 142.3 (C$^4$), 138.4 (C$^{10}$), 136.5 (C$^{13}$), 130.6 (C$^3$, C$^5$), 129.2 (C$^1$)*, 126 (q, J = 3.8 Hz, C$^2$, C$^6$), 124.2 (CF$_3$)*, 123.6 (C$^7$), 121.2 (C$^{12}$), 113.6 (C$^9$), 21.2 (C$^{11}$), 20.4 (C$^{14}$). $^{19}$F NMR (470.168 MHz, δ, CDCl$_3$): -62.54 (s, CF$_3$). IR (neat) cm$^{-1}$: 3465, 3367, 2922, 1614, 1449, 1319, 1116. HRMS (ESI-TOF): Calcd. for C$_{15}$H$_{15}$F$_3$N [M+H]$^+$ 266.1151, found 266.1158. The regiochemistry of the product was unequivocally determined by the observation of a positive NOE effect H$^{NH2}$-H$^9$, H$^{11}$-H$^9$, H$^{11}$-H$^{12}$, H$^{14}$-H$^{12}$, H$^{3,5}$-H$^{14}$, H$^{3,5}$-H$^{NH2}$ in a NOESY NMR experiment. *The chemical shifts were determined by HSQC $^{13}$C-$^{19}$F.

**1,4-Diaminobenzene** as arene. ArX = ***p*-CF$_3$C$_6$H$_4$I** (**5k**). The product is obtained as a brown oil. R$_f$ (SiO$_2$, *n*-hexane:EtOAc = 8:2) = 0.26. Yield = 0.031 g (36%).

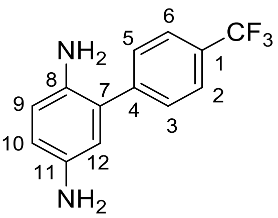


4′-(Trifluoromethyl)[1,1′-biphenyl]-2,5-diamine: $^1$H NMR (500.13 MHz, δ, CDCl$_3$): 7.67 (d, J = 8.4 Hz, 2H, H$^2$, H$^6$), 7.55 (d, J = 8.4 Hz, 2H, H$^3$, H$^5$), 6.76 (dd, J = 8.8, 2.4 Hz, 1H, H$^9$), 6.68 (m, 2H, H$^{10}$, H$^{12}$), 4.49 (br, 4H, H$^{NH2}$). $^{13}$C{$^1$H} NMR (125.78 MHz, δ, CDCl$_3$): 142.8 (C$^4$), 136.9 (C$^{11}$), 135.0 (C$^8$), 129.4 (C$^3$, C$^5$, C$^1$)*, 127.5 (C$^7$), 125.7 (C$^2$, C$^6$), 124.1 (CF$_3$)*, 119.0 (C$^{10}$), 118.4 (C$^9$), 117.5 (C$^{12}$). $^{19}$F NMR (470.168 MHz, δ, CDCl$_3$): -62.53 (s, CF$_3$). HRMS (ESI-TOF): Calcd. for C$_{13}$H$_{12}$F$_3$N$_2$ [M+H]$^+$ 253.0947, found 253.0951. *The chemical shifts were determined by HSQC $^{13}$C-$^{19}$F and HMBC $^{13}$C-$^{19}$F.

**Aniline** as arene. ArX = ***p*-CH$_3$C$_6$H$_4$I** (**5ab**). The product is obtained as a colorless oil. Yield: 0.039 g (63 %).

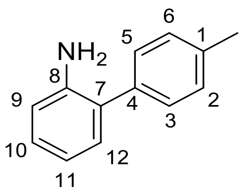


4'-Methyl-[1,1'-biphenyl]-2-amine:[13] $^1$H NMR (500.13 MHz, δ, $CDCl_3$): 7.35 (d, J = 8.3 Hz, 2H, $H^3$, $H^5$), 7.26 (d, J = 8.3 Hz, 2H, $H^2$, $H^6$), 7.13 (m, 2H, $H^{10}$, $H^{12}$), 6.83 (td, J = 7.5, 1.1 Hz, 1H, $H^{11}$), 6.76 (dd, J = 7.9, 1.1 Hz, 1H, $H^9$), 3.75 (br, 2H, $H^{NH2}$), 2.39 (s, 3H, Me). $^{13}C\{^1H\}$ NMR (125.78 MHz, δ, $CDCl_3$): 142.9 ($C^8$), 136.9 ($C^1$), 136.4 ($C^4$), 130.5 ($C^{12}$), 129.5 ($C^2$, $C^6$), 128.9 ($C^3$, $C^5$), 128.3 ($C^{10}$), 128.0 ($C^7$), 119.0 ($C^{11}$), 115.9 ($C^9$), 21.2 (Me). MS (EI, 70 eV): m/z (%) 183 (100) [$M^+$], 167 (45) 152 (15). The regiochemistry of the product was unequivocally determined by the observation of a positive NOE effect $H^{3,5}$-$H^{12}$, $H^{10,12}$-$H^{11}$, $H^{10}$-$H^9$ $H^{NH2}$-$H^9$ in a NOESY NMR experiment.

**Aniline** as arene. ArX = ***p*-(OMe)$C_6H_4$Br** (**5ac**). The product is obtained as a white solid. Yield: 0.047 g (69 %).

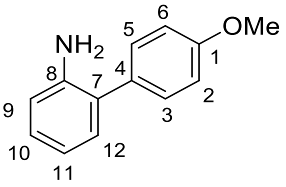


4'-Methoxy-[1,1'-biphenyl]-2-amine:[14] $^1$H NMR (500.13 MHz, δ, $CDCl_3$): 7.37 (m, 2H, $H^3$, $H^5$), 7.14 (ddd, J = 7.9, 7.4, 1.6 Hz, 1H, $H^{10}$), 7.11 (dd, J = 7.4, 1.5 Hz, 1H, $H^{12}$), 6.97 (m, 2H, $H^2$, $H^6$), 6.82 (td, J = 7.4, 1.3 Hz, 1H, $H^{11}$), 6.77 (dd, J = 7.6, 1.1 Hz, 1H, $H^9$), 3.92 (br, 2H, $H^{NH2}$), 3.85 (s, 3H, OMe). $^{13}C\{^1H\}$ NMR (125.78 MHz, δ, $CDCl_3$): 158.8 ($C^1$), 143.3 ($C^8$), 131.7 ($C^4$), 130.5 ($C^{12}$), 130.2 ($C^3$, $C^5$), 128.2 ($C^{10}$), 127.6 ($C^7$), 118.8 ($C^{11}$), 115.7 ($C^9$), 114.2 ($C^2$, $C^6$), 55.3 (OMe). MS (EI, 70 eV): m/z (%) 199 (100) [$M^+$], 184 (45), 168 (30).

### 1.3.2 Amination of 4-iodobenzotrifluoride with anilines.

The procedure used follows the conditions reported in the literature with small variations.[15]

$Pd_2dba_3.CHCl_3$ (25.8 mg, 0.025mmol), Xphos (23.8 mg, 0.050 mmol) and cesium carbonate (231.3 mg, 0.71 mmol) were introduced in a Schlenk flask in a nitrogen atmosphere. Then, the corresponding aniline (0.5 mmol) and DMF (0.25 mL) were added. The reaction mixture was stirred for 10 min at room temperature. Then 4-iodobenzotrifluoride (75 μL, 0.5 mmol) was added. The mixture was stirred at 65 ºC for 18 h and checked by $^{19}$F NMR of the crude mixture. The crude was purified by column chromatography using silica gel and a mixture of *n*-hexane:EtOAc = 1:1.

<u>*N*-Phenyl-4-(trifluoromethyl)benzenamine (**6a**).</u>[16] The product was obtained as a brown oil. Yield: 0.072 g (61 %).

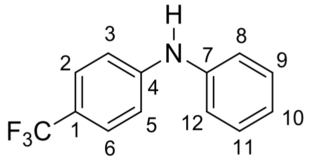


$^1$H NMR (500.13 MHz, δ, $CDCl_3$): 7.45 (d, J = 8.6 Hz, 2H, $H^2$, $H^6$), 7.33 (t, J = 7.4 Hz, 2H, $H^9$, $H^{10}$), 7.15 (d, J = 7.4 Hz, 2H, $H^8$, $H^{12}$), 7.05 (m, 3H, $H^3$, $H^5$, $H^{10}$), 5.91 (br, 1H, $H^{NH}$). $^{13}$C{$^1$H} NMR (125.78 MHz, δ, $CDCl_3$): 146.9 ($C^4$), 141.2 ($C^7$), 129.6 ($C^9$, $C^{11}$), 126.7 (q, $^3J_{C\text{-}F}$ = 4 Hz, $C^2$, $C^6$), 124.6 (q, $^1J_{C\text{-}F}$ = 270 Hz, $CF_3$), 122.9 ($C^{10}$), 121.7 (q, $^2J_{C\text{-}F}$ = 31 Hz, $C^1$), 120.1 ($C^8$, $C^{12}$), 115.3 ($C^3$, $C^5$). $^{19}$F NMR (470.168 MHz, δ, $CDCl_3$): -61.48 (s, $CF_3$). MS (EI, 70 eV): m/z (%) 237 (100) [$M^+$], 167 (40).

<u>*N*-Methyl-N-phenyl-4-(trifluoromethyl)benzenamine (**6b**).</u>[17] The product was obtained as a brown oil. Yield: 0.052 g (41 %).

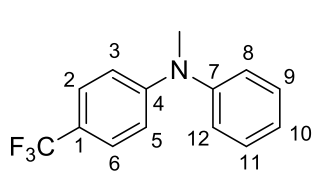


$^1$H NMR (500.13 MHz, δ, $CDCl_3$): 7.41 (m, 4H, $H^2$, $H^6$, $H^8$, $H^{12}$), 7.20 (m, 3H, $H^9$, $H^{10}$, $H^{11}$), 6.85 (d, J = 8.9 Hz, 2H, $H^3$, $H^5$), 3.35 (s, 3H, Me). $^{13}$C{$^1$H} NMR (125.78 MHz, δ, $CDCl_3$): 151.5 ($C^4$), 147.7 ($C^7$), 129.7 ($C^8$, $C^{12}$), 126.2 (q, $^3J_{C\text{-}F}$ = 3.5 Hz, $C^2$, $C^6$), 125.2 ($C^9$, $C^{11}$), 124.9 ($C^{10}$), 124.8 (q, $^1J_{C\text{-}F}$ = 264 Hz, $CF_3$), 119.8 (q, $^2J_{C\text{-}F}$ = 27 Hz, $C^1$), 114.7 ($C^3$, $C^5$), 40.2 (Me). $^{19}$F NMR (470.168 MHz, δ, $CDCl_3$): -61.26 (s, $CF_3$). MS (EI, 70 eV): m/z (%) 251 (100) [$M^+$], 232 (10), 167 (20).

## 1.4- Mechanistic experiments.

### 1.4.1. Behavior of complex 1 with aniline.

Complex **1** (5.3 mg, 0.01 mmol) was added into an NMR tube along with a sealed glass capillary filled with DMSO-$d_6$ as NMR lock signal. Then, 0.6 mL of dry DMA were added. The corresponding aniline was added to the tube and the Pd:aniline molar ratio is specified in the spectra. The species formed in solution at room temperature were examined by $^{19}$F NMR.

The spectroscopic data of the identified species are given below.

**1**: Mixture of isomers A and B.[3] Isomer A: $^{19}$F NMR (470.168 MHz, δ, DMA/DMSO-$d^6$ capillary): -119.29 (m, 2F, $F_{ortho}$), -166.52 (t, J = 19.1 Hz, 1F, $F_{para}$), -168.36 (m, 2F, $F_{meta}$). Isomer B: $^{19}$F NMR (470.168 MHz, δ, DMA/DMSO-$d^6$ capillary): -119.54 (m, 2F, $F_{ortho}$), -161.70 (t, J = 21.8 Hz, 1F, $F_{para}$), -164.42 (m, 2F, $F_{meta}$).

**7**: $^{19}$F NMR (470.168 MHz, δ, DMA/DMSO-$d^6$ capillary): -118.50 (m, 2F, $F_{ortho}$), -161.83 (t, J = 20.1 Hz, 1F, $F_{para}$), -164.02 (m, 2F, $F_{meta}$).

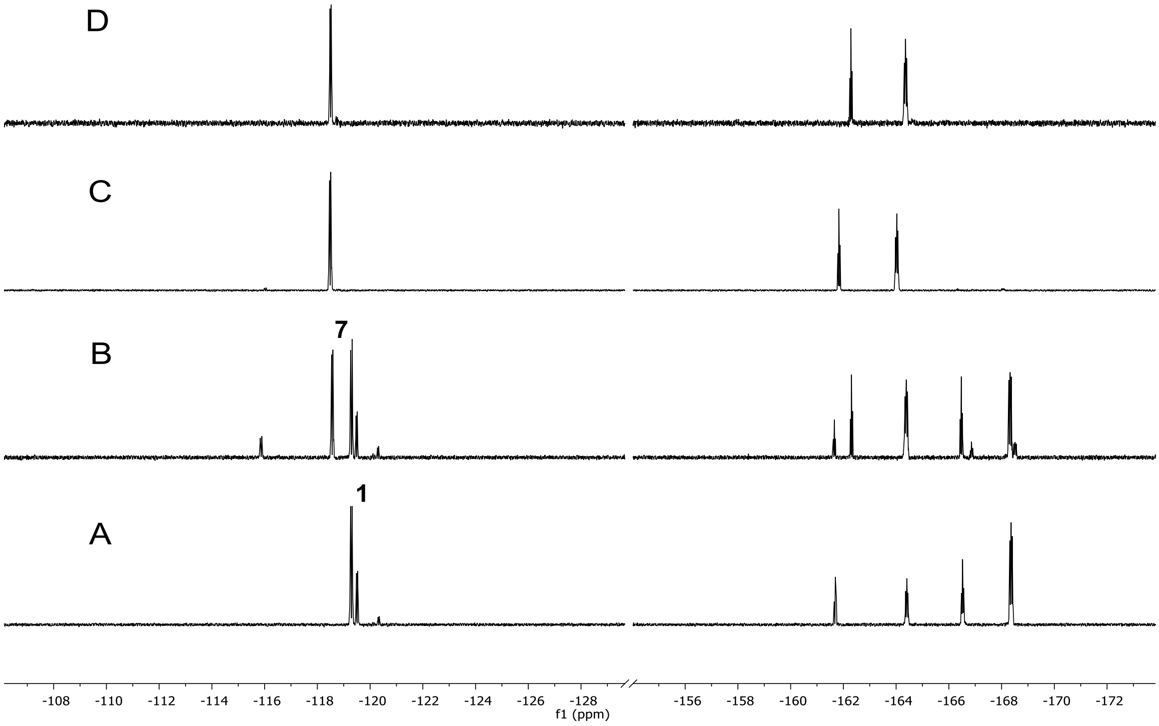


**Figure S1.** $^{19}$F NMR spectra (470.168 MHz): A) Complex **1** in DMA (mixture of isomers). B) Sample A after adding 10 equivalents of aniline. C) Sample A after adding 200 equivalents of aniline. D) Complex **7** in DMA.

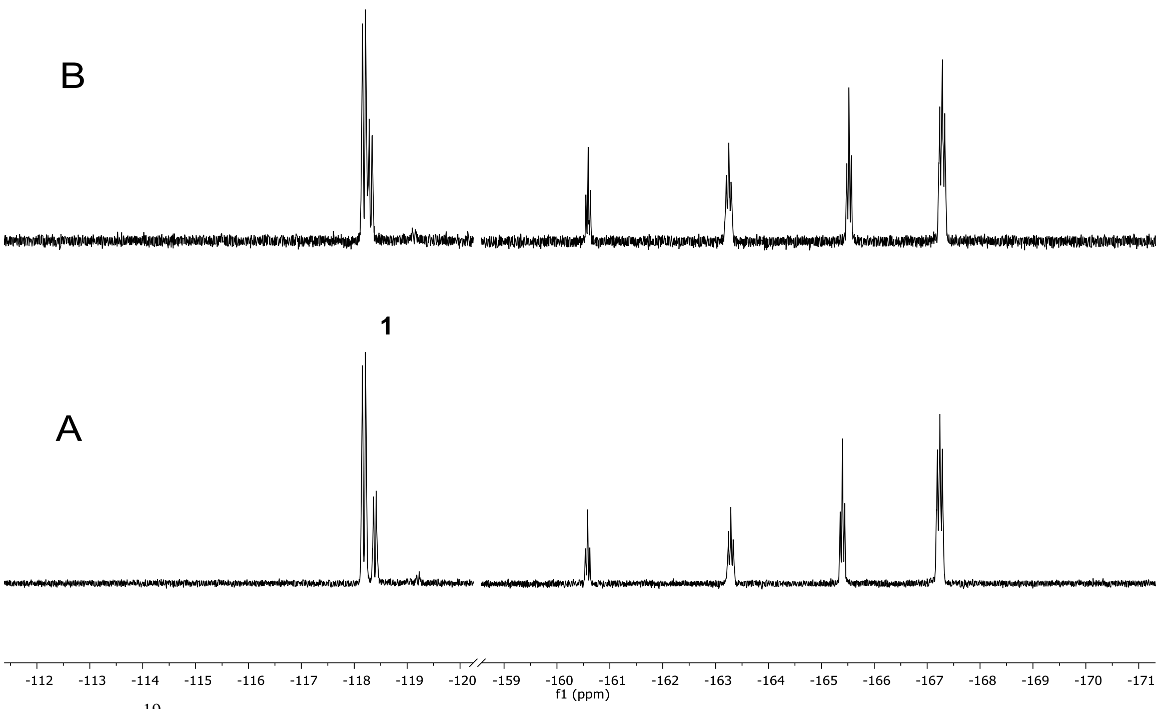


**Figure S2.** $^{19}$F NMR spectra (470.168 MHz): A) Complex **1** in DMA (mixture of isomers). B) Sample A after adding 200 equivalents of *N*, *N*-dimethylaniline.

### 1.4.2. Thermal decomposition of complex 8 under catalytic conditions.

Complex **8** (5.2 mg, 0.01 mmol) and $Cs_2CO_3$ (3.3 mg, 0.01 mmol) were added into an NMR tube along with a sealed glass capillary filled with DMSO-$d_6$ as NMR lock signal. Then, 0.6 mL of DMA and aniline (183 μL, 2 mmol) were added. The species formed in solution at room temperature were examined by $^{19}$F NMR. Then, the mixture was heated at 130 °C for the specified time.

**8**: $^{19}$F NMR (470.168 MHz, δ, DMA/DMSO-$d^6$ capillary): -61.58 (s, $CF_3$).

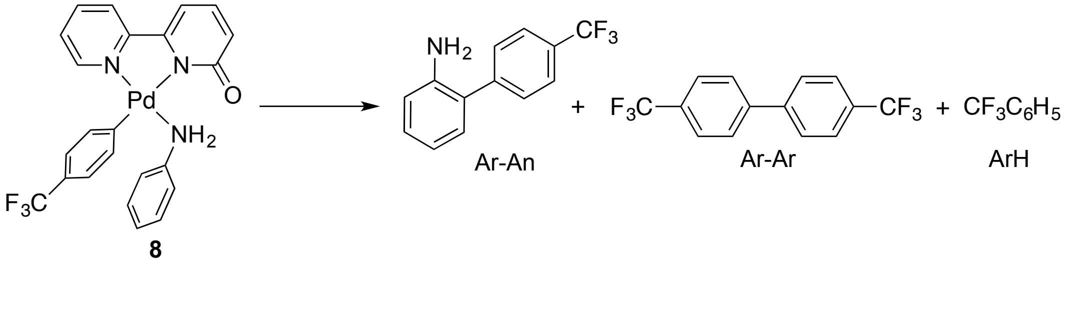

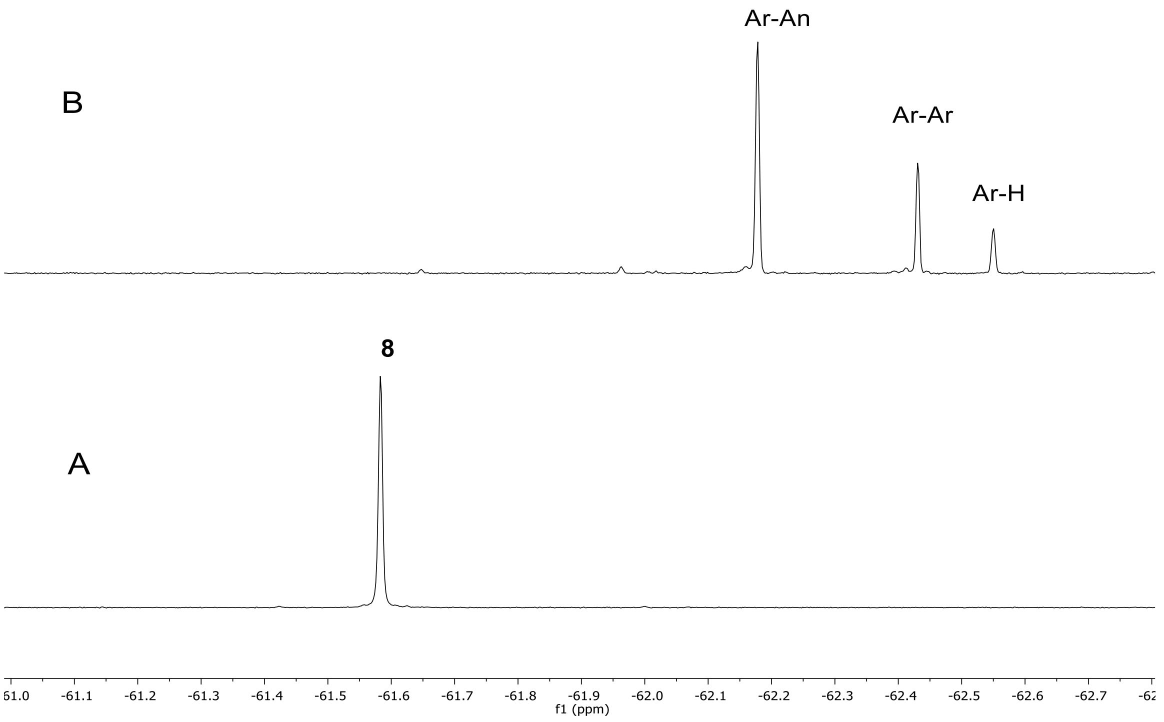


**Figure S3.** $^{19}$F NMR spectra (470.168 MHz): A) Complex **8** in DMA with 200 equivalents of $PhNH_2$ and 1 equivalent of $Cs_2CO_3$. B) Sample A after heating at 130 °C for 30 min (mol ratio ArAn : Ar-Ar : ArH = 5 : 1 : 1).

### 1.4.3. H-D exchange in complexes 1 and 8 with deuterium oxide.

The palladium complex (0.01 mmol) was added into an NMR tube along with a sealed glass capillary filled with DMSO-$d_6$ as NMR lock signal. Then, 0.6 mL of dry DMA and deuterium oxide (1 μL) were added to the tube.

The H-D exchange was observed at room temperature by $^1$H NMR for the resonances specified below.

**1**: Mixture of isomers A and B. Isomer A: $^1$H NMR (500.13 MHz, δ, DMA/DMSO-$d^6$ capillary): 13.40 (br, 1H, OH). Isomer B: $^1$H NMR (500.13 MHz, δ, DMA/DMSO-$d^6$ capillary): 10.9 (br, 1H, OH).

**8**: $^1$H NMR (500.13 MHz, δ, DMA/DMSO-$d^6$ capillary): 9.38 (br, 2H, $NH_2$).

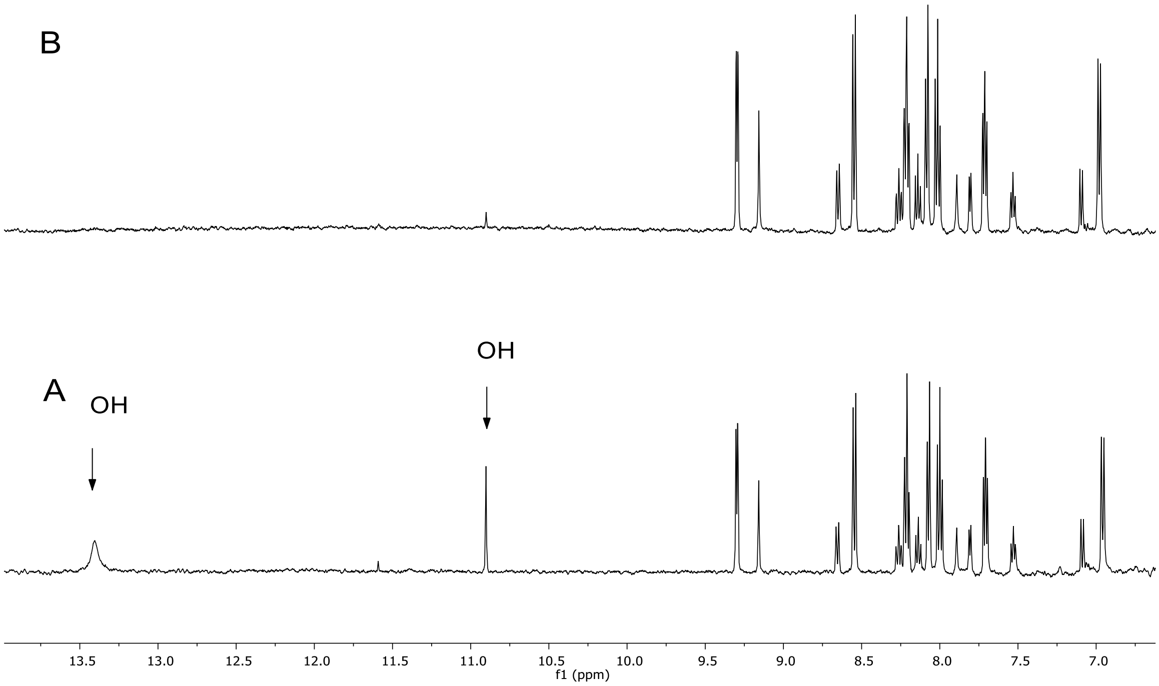


**Figure S4.** $^{1}H$ NMR spectra (500.13 MHz): A) Complex **1** in DMA. B) Sample A after adding $D_2O$.

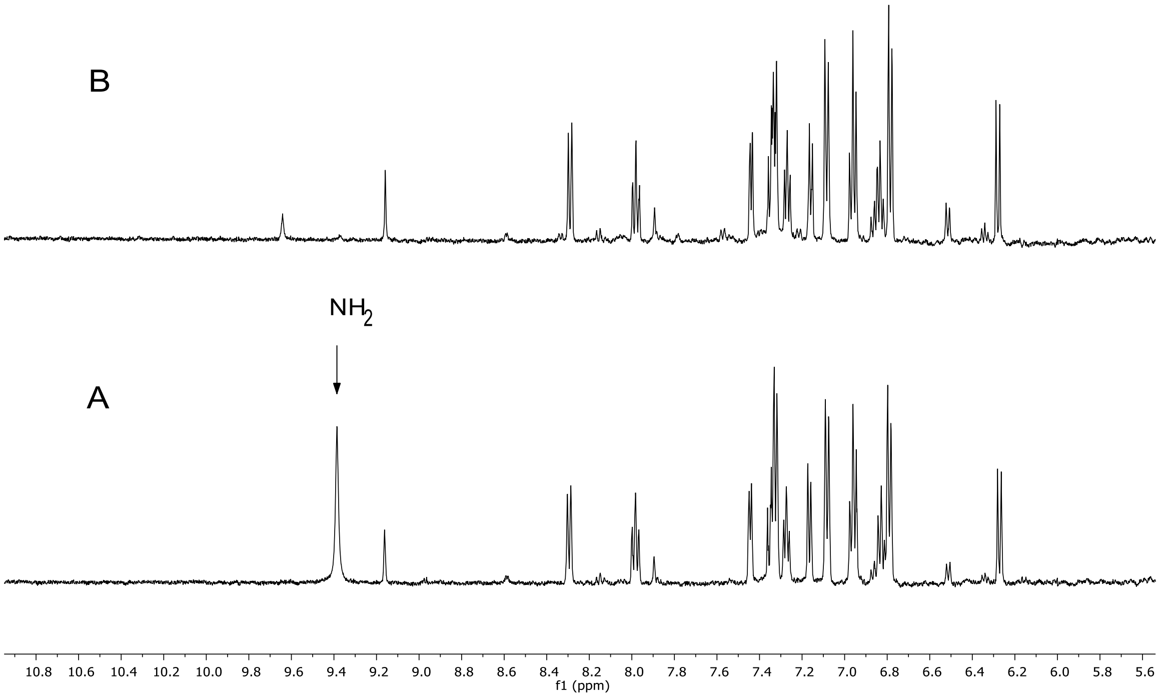


**Figure S5**. $^{1}H$ NMR spectra (500.13 MHz): A) Complex **8** in DMA. B) Sample A after adding $D_2O$.

### 1.4.4. Determination of equilibrium constants for the coordination of anilines and comparison with the regioselectivity observed.

The coordination ability of the N-substituted anilines and aniline was determined by measuring the equilibrium constants of their coordination to the model palladium complex $(NBu_4)[Pd_2(\mu\text{-}Br)_2(C_6F_5)_4]$ (Eq. S1), taking advantage of the distinct patterns of complexes **I** and **II** in $^{19}$F NMR (Figure S6).

Samples of 400 μL of a 4.72 $10^{-3}$ M solution of $(NBu_4)_2[Pd_2(\mu\text{-}Br)_2(C_6F_5)_4]$ (**I**) in dichloromethane were placed in 1 mL volumetric flasks. A different known quantity of the chosen aniline was added to each sample and the volumetric flasks were filled with $CH_2Cl_2$. 0.6 mL of the final solution were introduced into a NMR tube along with a sealed glass capillary filled with acetone-$d_6$ as NMR lock signal. The species formed in the solution at room temperature were examined by $^{19}$F NMR. The equilibrium concentrations of complexes **I** and **II** (Eq. S1) were determined by integration of $^{19}$F NMR signals. The values of $K_{eq}$ and errors were determined as an average of three measurements.

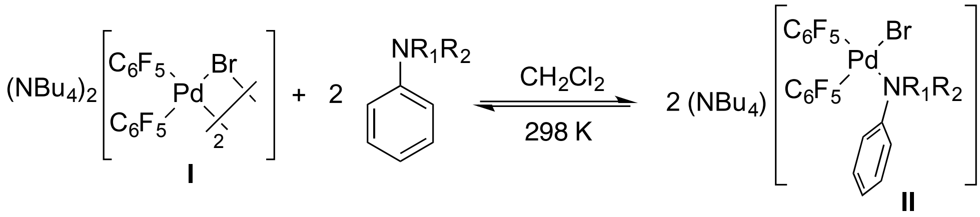


(S1)

$$K_{eq} = \frac{[II]^2}{[I]\,[Aniline]^2}$$

**Table S1.** Equilibrium constants ($K_{eq}$) for the coordination of different anilines.

| Entry | $R_1$, $R_2$ | $K_{eq}$ (L mol$^{-1}$) |
|---|---|---|
| 1 | H | 118 ± 4 |
| 2 | H, Me | 1.2 ± 0.4 |
| 3 | H, $^{i}$Pr | 0.027 ± 0.016 |
| 4 | Me | 1.7x10$^{-5}$ ± 0.6x10$^{-5}$ |

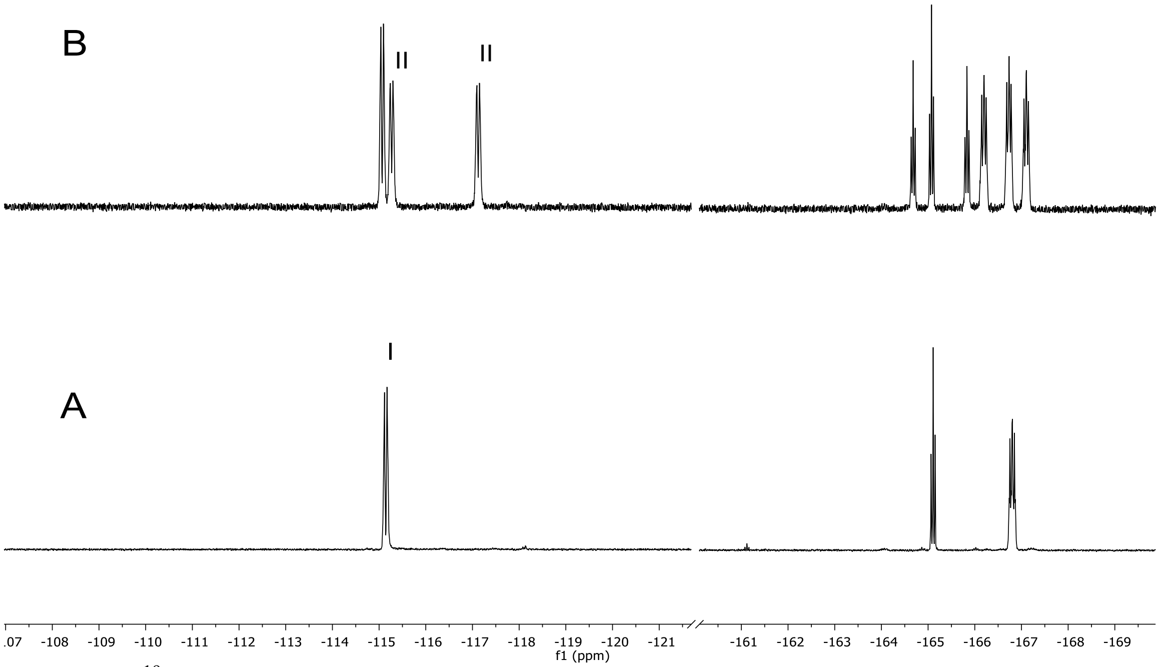


**Figure S6.** $^{19}$F NMR spectra (470.168 MHz): A) Complex **I** in dichloromethane. B) Sample A after adding 10 equivalents of aniline.

The $K_{eq}$ values show a clear decrease in coordination ability of the anilines as the bulkiness of the N-substitution increases, $PhNMe_2$ being the least coordinating one. A plot of log ($K_{eq}$) vs log (regioselectivity) was created, taken the selectivity as the percentage of *ortho* isomer observed in the arylation reaction. Since the *ortho* isomer was not detected for $PhNMe_2$, a maximun of 1 % corresponding to the NMR integration uncertainty was assumed.

Although a linear decrease was observed for aniline and the secondary anilines, no correlation was observed for all the anilines tested (Figure S7).

**Table S2.** Coordination equilibrium constants ($K_{eq}$) and selectivity for different anilines.

| **Entry** | **$R_1$, $R_2$** | **$K_{eq}$ (L mol$^{-1}$)** | **Ratio o:m:p** | **Regioselectivity (% *ortho*)** |
|---|---|---|---|---|
| 1 | H | 118 ± 4 | 25:1:1 | 93 |
| 2 | H, Me | 1.2 ± 0.4 | 18:2:1 | 86 |
| 3 | H, $^{i}$Pr | 0.027 ± 0.016 | 12:3:1 | 75 |
| 4 | Me | $1.7x10^{-5} \pm 0.6x10^{-5}$ | 0:1.2:1 | 1 |

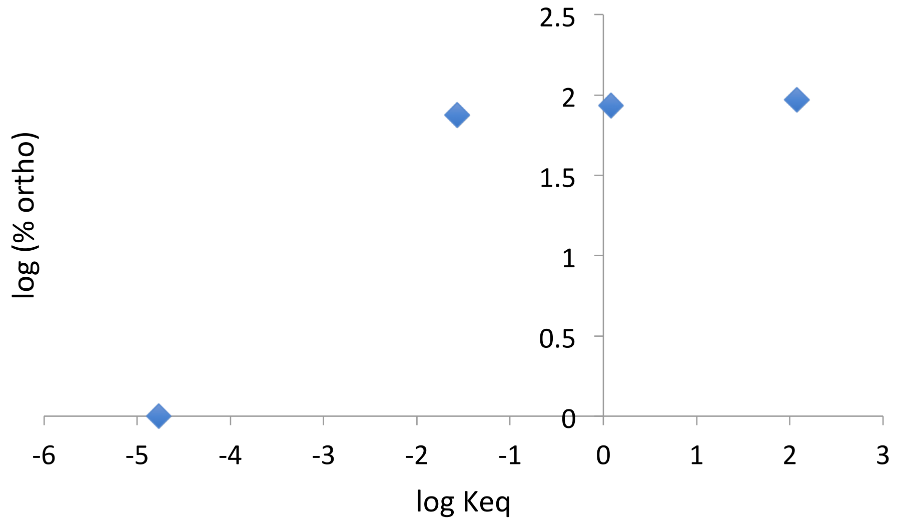


**Figure S7.** Plot of log($K_{eq}$) (coordination equilibirum) vs log(regioselectivity) in the arylation of different anilines. Data from Table S2.

### 1.4.5. Probing the intermediacy of Pd(IV) species.

We checked the possibility of a formation of a Pd(IV) intermediate in our reaction using two model Pd(II) aryl complexes.

a) [Pd(bipy-6-O)($C_6F_5$)($PhNH_2$)] (**7**). Complex **7** (0.01 mmol) was placed into an NMR tube along with a sealed glass capillary filled with DMSO-$d_6$ as NMR lock signal. Then, 0.6 mL of dry DMA and *p*-$CF_3C_6H_4I$ (0.02 mmol) were added. The mixture was heated at 130 °C for 30 min. The species formed were examined by $^{19}F$ NMR. We did not observed any C-C cross coupling product (*p*-$CF_3C_6H_4$–$C_6F_5$) and only a small amount of $C_6F_5H$ (7 %) from decomposition of **7** was found.

b) [Pd($\kappa^2$-N,C-$NH_2CH_2C_6H_4$)(bipy6-O)]. This complex was prepared by reaction of [Pd($\mu$-OAc)($\kappa^2$-N,C-$NH_2CH_2C_6H_4$)]$_2$ synthesized as reported in the literature,[18] with the stoichiometric amount of bipy-6-OH (Scheme S1).

$^1H$ NMR (500.13 MHz, δ, $CDCl_3$): 8.91 (d, J = 5.4 Hz, 1H, $H^{3'}$), 7.91 (m, 2H, $H^{5'}$, $H^{6'}$), 7.40 (m, 2H, $H^{4'}$, $H^4$), 7.22 (d, J = 6.5 Hz, 1H, $H^{11}$), 7.09 (m, 3H, $H^8$, $H^9$, $H^{10}$), 6.96 (d, J = 6.5 Hz, 1H, $H^3$), 6.64 (d, J = 8.6 Hz, 1H, $H^5$), 5.62 (br, 2H, $H^{NH2}$), 4.28 (t, J = 5.6 Hz, 2H, $H^{13}$).

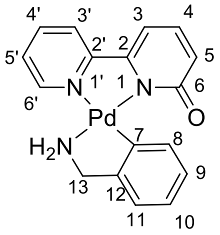

The same procedure described above (a) was used for the reaction of this complex with *p*-$CF_3C_6H_4I$. No reaction was observed (Scheme S1).

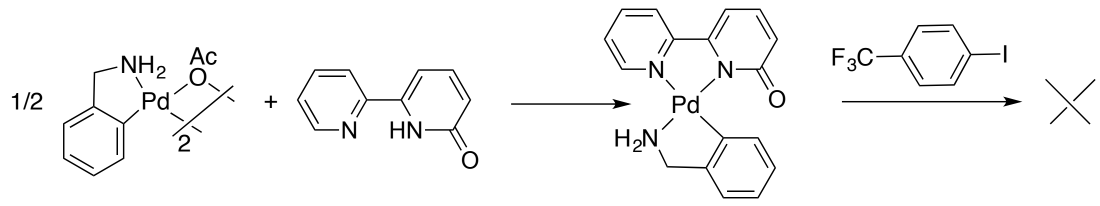


Scheme S1

## 1.5- Kinetic data.

### 1.5.1 Determination of the KIE.

Two Schlenk flasks equipped with a septum cap and a Teflon stirring bar were charged with [Pd(bipy-6-OH)Br($C_6F_5$)] (**1**) (9 mg, 0.017 mmol) and cesium carbonate (222 mg, 0.68 mmol) in a nitrogen atmosphere. 4-iodobenzotrifluoride (51 µL, 0.34 mmol) was added to each flask. Then, aniline (3.4 mmol, 310 µL) was added to one flask and aniline-2,3,4,5,6-$d_5$ to the other. Finally, DMA (2.7 mL) was added to each flasks ($[ArI]_0$ = 0.12 M). The Schlenk flasks were heated at 130 °C with constant stirring. At the indicated time, an aliquot was taken and analyzed by $^{19}F$ NMR adding 0.5 mL of $CDCl_3$ as NMR lock signal. The concentration of the product was determined by integration of the distinct trifluoromethyl signals of reagents and products. The ratio of initial rate constants for both experiments ($k_H/k_D$) gives the reported KIE value (see below).

**Table S3.** Time and product concentration data for the KIE determining experiments.[a]

| **Aniline** | | **Anilne-$d_5$** | |
|---|---|---|---|
| **Time (min)** | **[Product] (M)** | **Time (min)** | **[Product] (M)** |
| 15 | 0.0036 | 30 | 0.0024 |
| 30 | 0.0085 | 60 | 0.0036 |
| 60 | 0.0182 | 90 | 0.0049 |
| 75 | 0.0219 | 120 | 0.0061 |
| 90 | 0.0255 | 150 | 0.0085 |
| 105 | 0.0291 | 180 | 0.0109 |
| | | 210 | 0.0134 |
| | | 270 | 0.0182 |

[a]Initial concentrations: $[ArI]_0$ = 0.12 M; $[PhNH_2]_0$ = 1.2 M

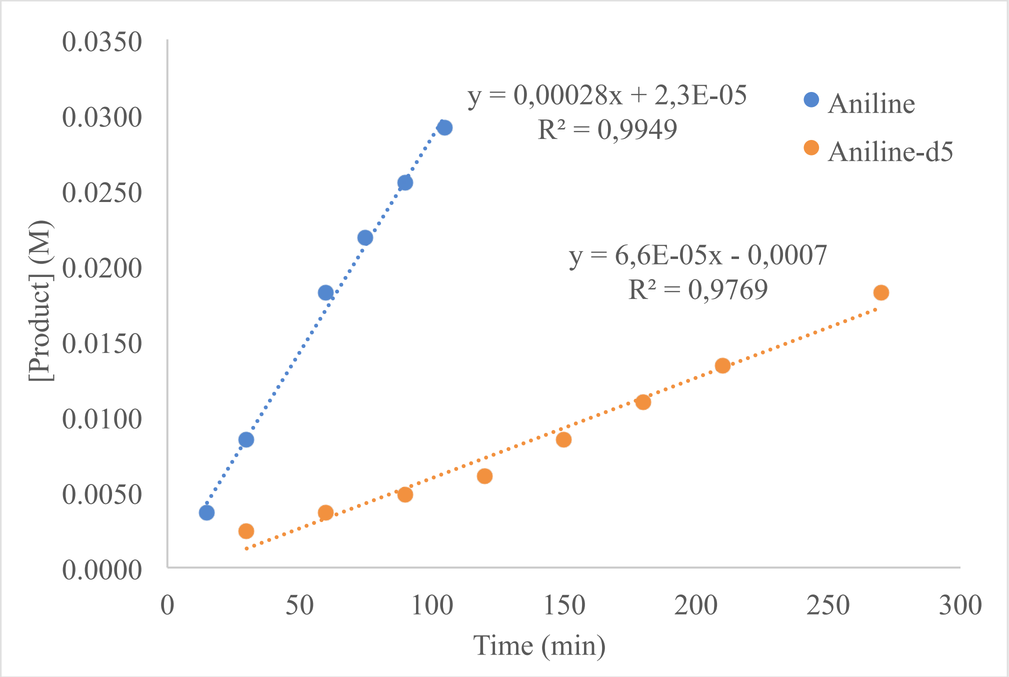


**Figure S8.** Concentration-time plots of the direct arylation of aniline and aniline-$d_5$.

The ratio of initial reaction rate constants gives the KIE value:
$k_H = 2.8 \pm 0.1 \times 10^{-4}$; $k_D = 6.6 \pm 0.4 \times 10^{-5}$ . KIE = $k_H/k_D = 4.2 \pm 0.4$

### 1.5.2 Kinetic experiments of the direct arylation of aniline.

A Schlenk flask equipped with a septum cap and a Teflon stirring bar were charged, in a nitrogen atmosphere, with [Pd(bipy-6-OH)Br($C_6F_5$)] (**1**) and cesium carbonate. Then, 4-iodobenzotrifluoride, aniline and DMA (2.7 mL) were added. The Schlenk flask was heated at 130 °C with constant stirring. At the indicated time, an aliquot was taken and analyzed by $^{19}$F NMR adding 0.5 mL of $CDCl_3$ as NMR lock signal. The concentration of the product was determined by integration of the distinct trifluoromethyl signals of reagents and products.

The variable time normalization analysis (VTNA) reported by Burés,[19] was used to determine the order on the reactants for the catalytic reaction. Four experiments were performed each time varying one of the reagent's initial concentration (Table S4). The resulting plots are represented in Figure S9.

**Table S4.** Initial concentration values for the kinetic experiments.

| Experiment | [Cat] (M) | [ArI] (M) | [Aniline] (M) |
|---|---|---|---|
| 1 | 0.006 | 0.1214 | 1.2143 |
| 2 | 0.003 | 0.1214 | 1.2143 |
| 3 | 0.006 | 0.2429 | 1.2143 |
| 4 | 0.006 | 0.1214 | 1.7586 |

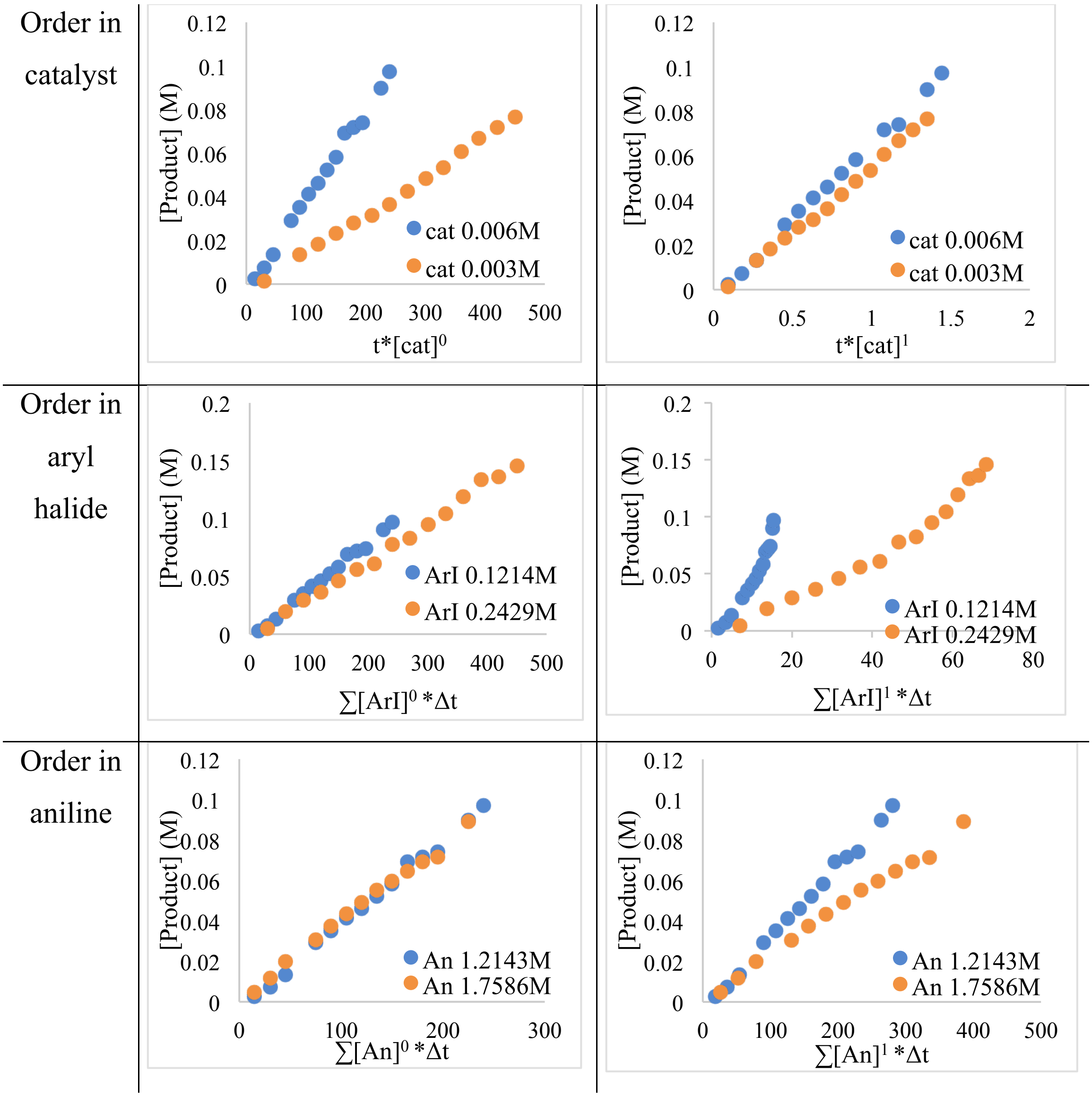


**Figure S9.** Plots derived from the variable time normalization analysis (VTNA). Overlay of plots from two different experiments gives the order in the reagent whose initial concentration is changed (power value in abscissa axis).

**Table S5**. Kinetic data (product concentration at different times) for experiments 1-4 (see Table S4).

| Experiment | Time (min) | [Product] (M) |
|---|---|---|
| [Cat] = 0.006 M<br>[ArI] = 0.1214 M<br>[An] = 1.2143 M | 15 | 0.0024 |
| | 30 | 0.0073 |
| | 45 | 0.0134 |
| | 75 | 0.0291 |
| | 90 | 0.0352 |
| | 105 | 0.0413 |
| | 120 | 0.0461 |
| | 135 | 0.0522 |
| | 150 | 0.0583 |
| | 165 | 0.0692 |
| | 180 | 0.0716 |
| | 195 | 0.0741 |
| | 225 | 0.0899 |
| | 240 | 0.0971 |
| [Cat] = 0.003 M<br>[ArI] = 0.1214 M<br>[An] = 1.2143 M | 30 | 0.0012 |
| | 60 | 0.0012 |
| | 90 | 0.0134 |
| | 120 | 0.0182 |
| | 150 | 0.0231 |
| | 180 | 0.0279 |
| | 210 | 0.0316 |
| | 240 | 0.0364 |
| | 270 | 0.0425 |
| | 300 | 0.0486 |
| | 330 | 0.0534 |
| | 360 | 0.0607 |
| | 390 | 0.0668 |
| | 420 | 0.0716 |
| | 450 | 0.0765 |
| [Cat] = 0.006 M<br>[ArI] = 0.2429 M<br>[An] = 1.2143 M | 30 | 0.0049 |
| | 60 | 0.0194 |
| | 90 | 0.0291 |
| | 120 | 0.0364 |
| | 150 | 0.0461 |
| | 180 | 0.0559 |
| | 210 | 0.0607 |
| | 240 | 0.0777 |
| | 270 | 0.0826 |
| | 300 | 0.0947 |
| | 330 | 0.1044 |
| | 360 | 0.1190 |
| | 390 | 0.1336 |

| | | |
|---|---|---|
| | 420 | 0.1360 |
| | 450 | 0.1457 |
| [Cat] = 0.006 M<br>[ArI] = 0.1214 M<br>[An] = 1.7586 M | 15 | 0.0047 |
| | 30 | 0.0117 |
| | 45 | 0.0199 |
| | 75 | 0.0305 |
| | 90 | 0.0375 |
| | 105 | 0.0434 |
| | 120 | 0.0492 |
| | 135 | 0.0551 |
| | 150 | 0.0598 |
| | 165 | 0.0645 |
| | 180 | 0.0692 |
| | 195 | 0.0715 |
| | 225 | 0.0891 |

## *2. Data for X-Ray structure determinations*

Crystals suitable for X-ray analyses were obtained by slow evaporation of the solvent of a solution of complex [Pd(bipy-6-O)($C_6F_5$)($PhNH_2$)] (**7**) in $CH_2Cl_2$ at room temperature or a solution of complex [Pd(bipy-6-O)($C_6H_4$-*p*-$CF_3$)(κ-N-aniline)] (**8**) in $CH_2Cl_2$ at -20 °C. In each case, the crystal was attached to a glass fiber and transferred to an Agilent Supernova diffractometer with an Atlas CCD area detector. Data collection was performed with Mo Kα radiation (0.71073 Å) at 298 K. Data integration and empirical absorption correction were carried out using the CrysAlisPro program package.[20] The structures were solved by direct methods and refined by full-matrix least squares against $F^2$ with SHELX,[21] using Olex2.[22] The non-hydrogen atoms were refined anisotropically and hydrogen atoms were constrained to ideal geometries and refined with fixed isotropic displacement parameters. Refinement proceeded smoothly to give the residuals shown in Table S6. Complex **8** crystallized with a water molecule, hydrogen-bonded to the pyridone oxygen (Figure S11). Both crystal structures have deposited in the CCDC database (CCDC-2165844 and CCDC-2165845).

**Table S6.** Crystal data and structure refinement for complexes **7** and **8**.

| | **7** | **8** |
|---|---|---|
| Empirical formula | $C_{22}H_{14}N_3OF_5Pd$ | $C_{23}H_{20}N_3O_2F_3Pd$ |
| Formula weight | 537.76 | 515.80 |
| Temperature/K | 298 | 298 |
| Crystal system | triclinic | triclinic |
| Space group | P-1 | P-1 |
| a/Å | 7.6417(8) | 7.0887(4) |
| b/Å | 10.5945(9) | 11.1373(7) |
| c/Å | 12.9337(13) | 14.2923(8) |
| α/° | 94.254(8) | 86.703(5) |
| β/° | 90.812(9) | 83.477(5) |
| γ/° | 103.361(8) | 82.420(5) |
| Volume/Å$^3$ | 1015.43(17) | 1110.23(11) |
| Z | 2 | 2 |
| $\rho_{calc}$g/cm$^3$ | 1.759 | 1.543 |
| μ/mm$^{-1}$ | 0.979 | 0.879 |
| F(000) | 532.0 | 516.0 |
| Crystal size/mm$^3$ | 0.435 × 0.247 × 0.063 | 0.282 × 0.197 × 0.154 |

| | | |
|---|---|---|
| Radiation | MoKα (λ = 0.71073) | MoKα (λ = 0.71073) |
| 2Θ range for data collection/° | 6.692 to 58.998 | 6.694 to 58.93 |
| Index ranges | -10 ≤ h ≤ 9, -10 ≤ k ≤ 14, -17 ≤ l ≤ 17 | -7 ≤ h ≤ 9, -11 ≤ k ≤ 14, -19 ≤ l ≤ 18 |
| Reflections collected | 7073 | 8624 |
| Independent reflections | 4621 [$R_{int}$ = 0.0304, $R_{sigma}$ = 0.0690] | 5189 [$R_{int}$ = 0.0287, $R_{sigma}$ = 0.0579] |
| Data/restraints/parameters | 4621/0/289 | 5189/0/292 |
| Goodness-of-fit on $F^2$ | 1.053 | 1.063 |
| Final R indexes [I>=2σ (I)] | $R_1$ = 0.0454, $wR_2$ = 0.0750 | $R_1$ = 0.0463, $wR_2$ = 0.0956 |
| Final R indexes [all data] | $R_1$ = 0.0753, $wR_2$ = 0.0899 | $R_1$ = 0.0698, $wR_2$ = 0.1133 |
| Largest diff. peak/hole / e $Å^{-3}$ | 0.76/-0.59 | 0.76/-0.68 |

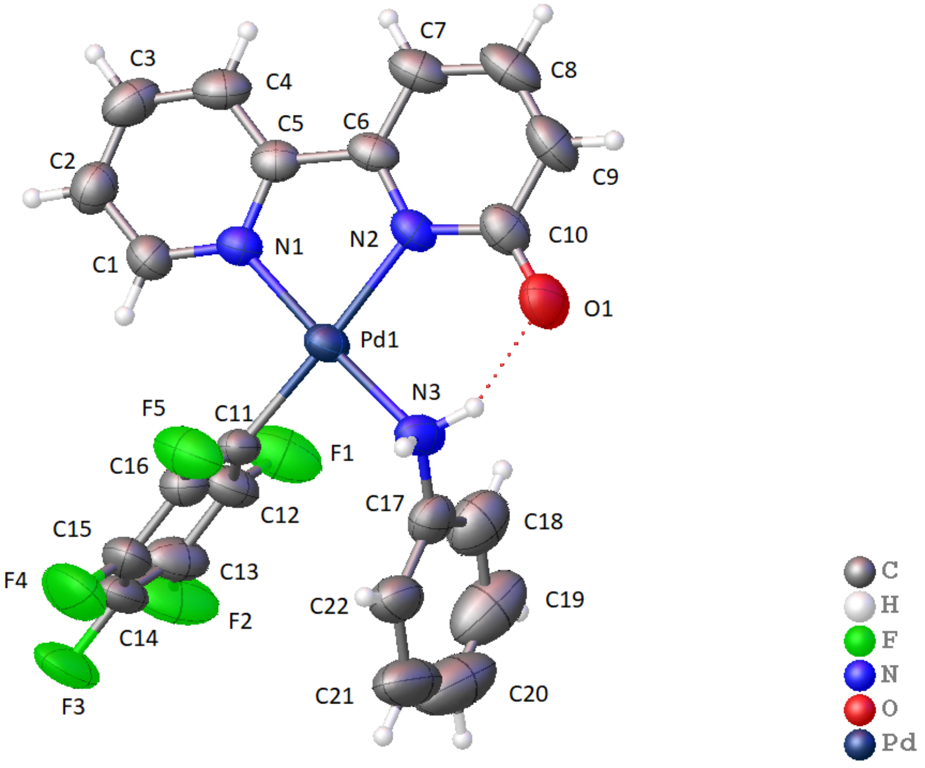


**Figure S10.** X-ray molecular structure of [Pd(bipy-6-O)($C_6F_5$)($PhNH_2$)] (**7**). ORTEP plot (40% probability ellipsoids) is shown.

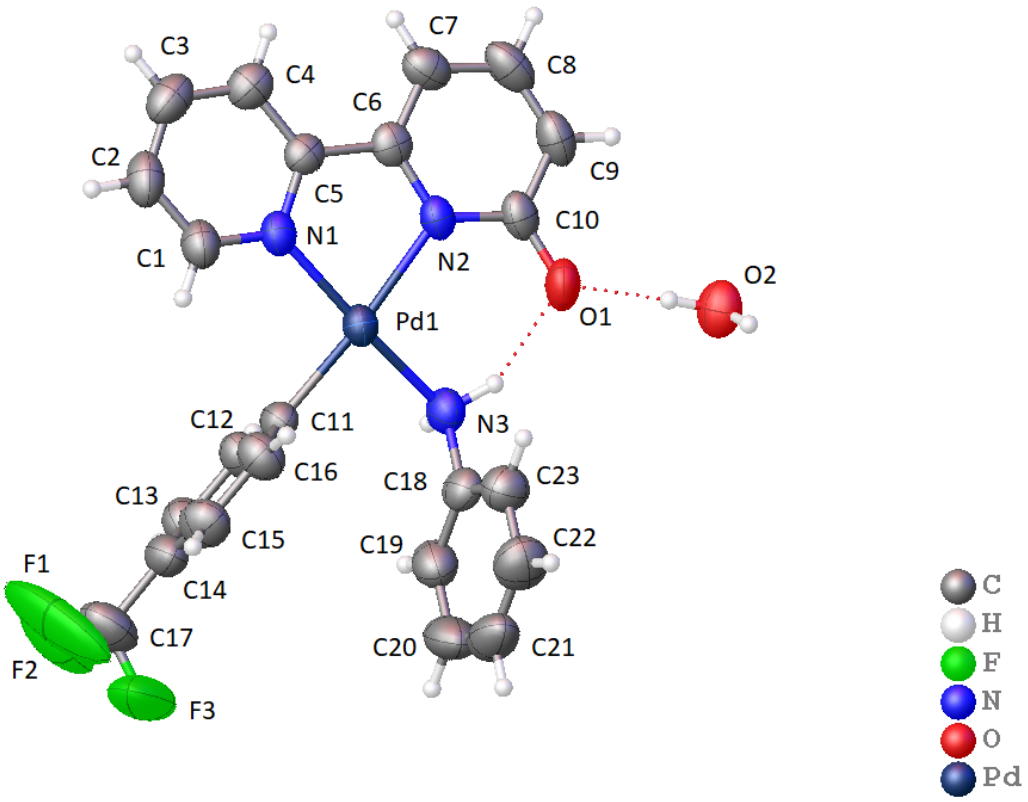


**Figure S11.** X-ray molecular structure of [Pd(Bipy-6-O)($C_6H_4$-*p*-$CF_3$)($PhNH_2$)] (**8**). ORTEP plot (40% probability ellipsoids) is shown.

## 3. *Selected Spectra*

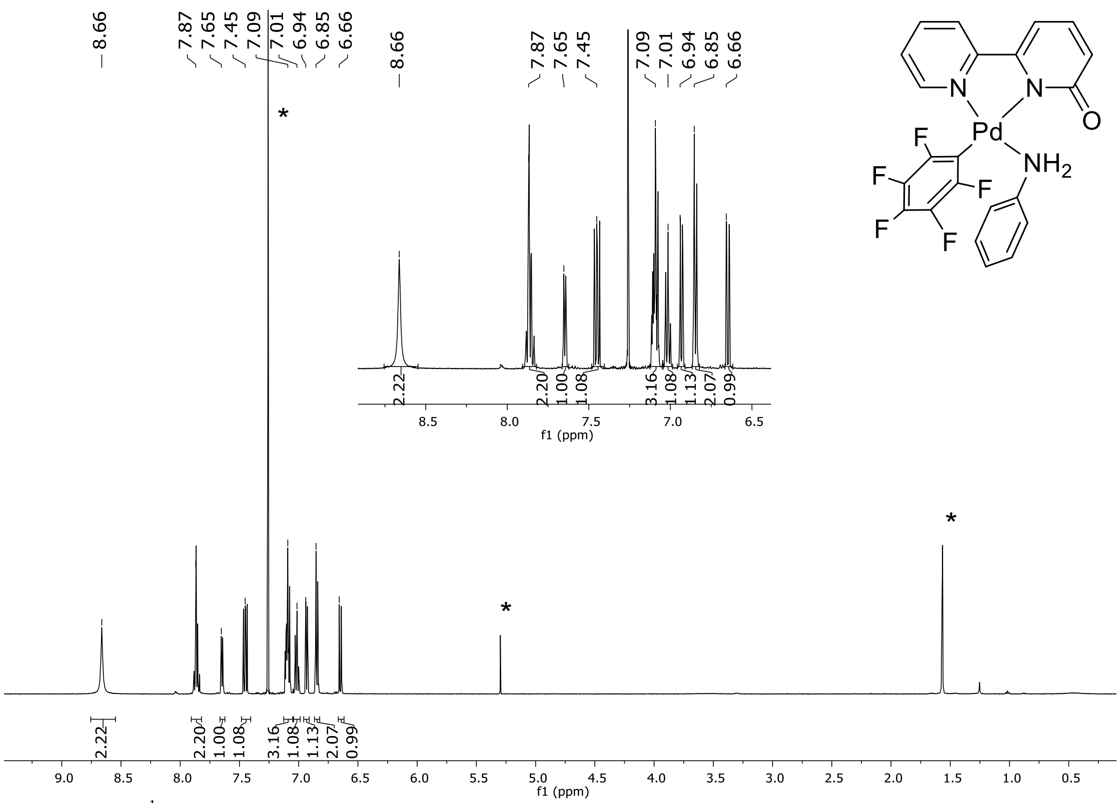

**Figure S12.** $^{1}H$ NMR (500.13 MHz, $CDCl_3$) of [Pd(bipy-6-O)($C_6F_5$)($PhNH_2$)] (**7**) at 298 K. (*) Signal corresponding to solvent (chloroform, $H_2O$ and dichloromethane).

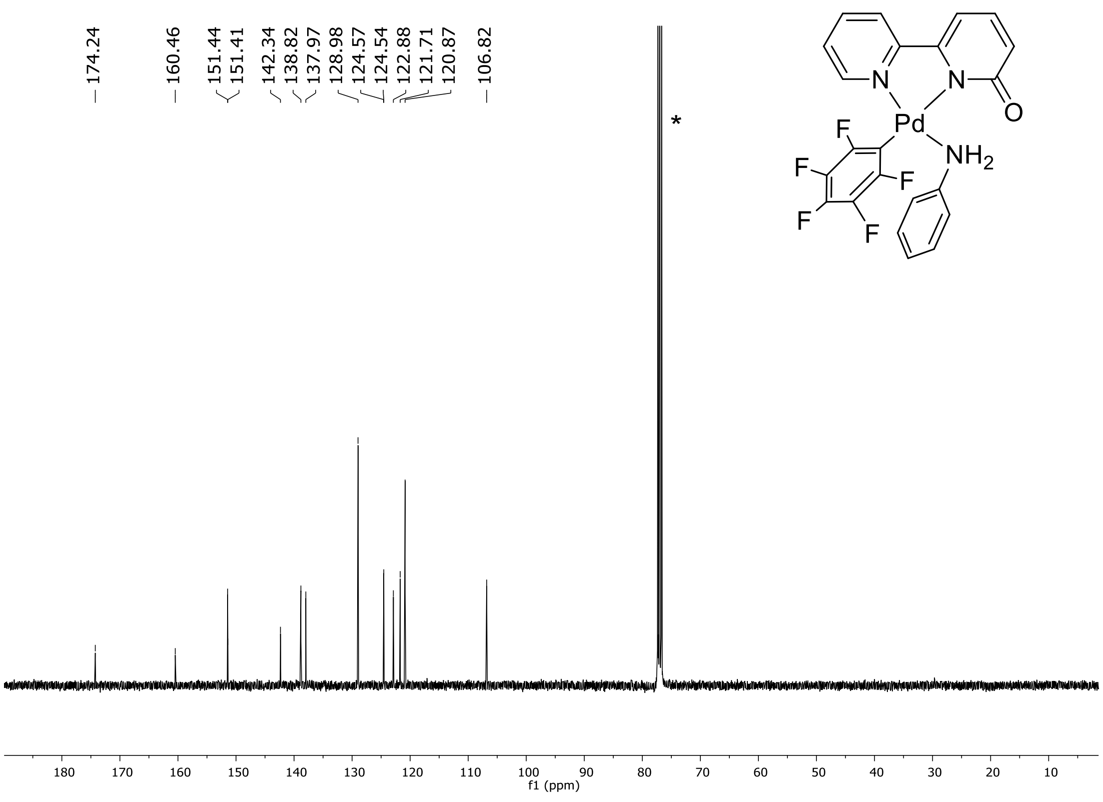

**Figure S13.** $^{13}C$ NMR (125.87 MHz, $CDCl_3$) of [Pd(bipy-6-O)($C_6F_5$)($PhNH_2$)] (**7**) at 298 K. (*) Signal corresponding to solvent (chloroform).

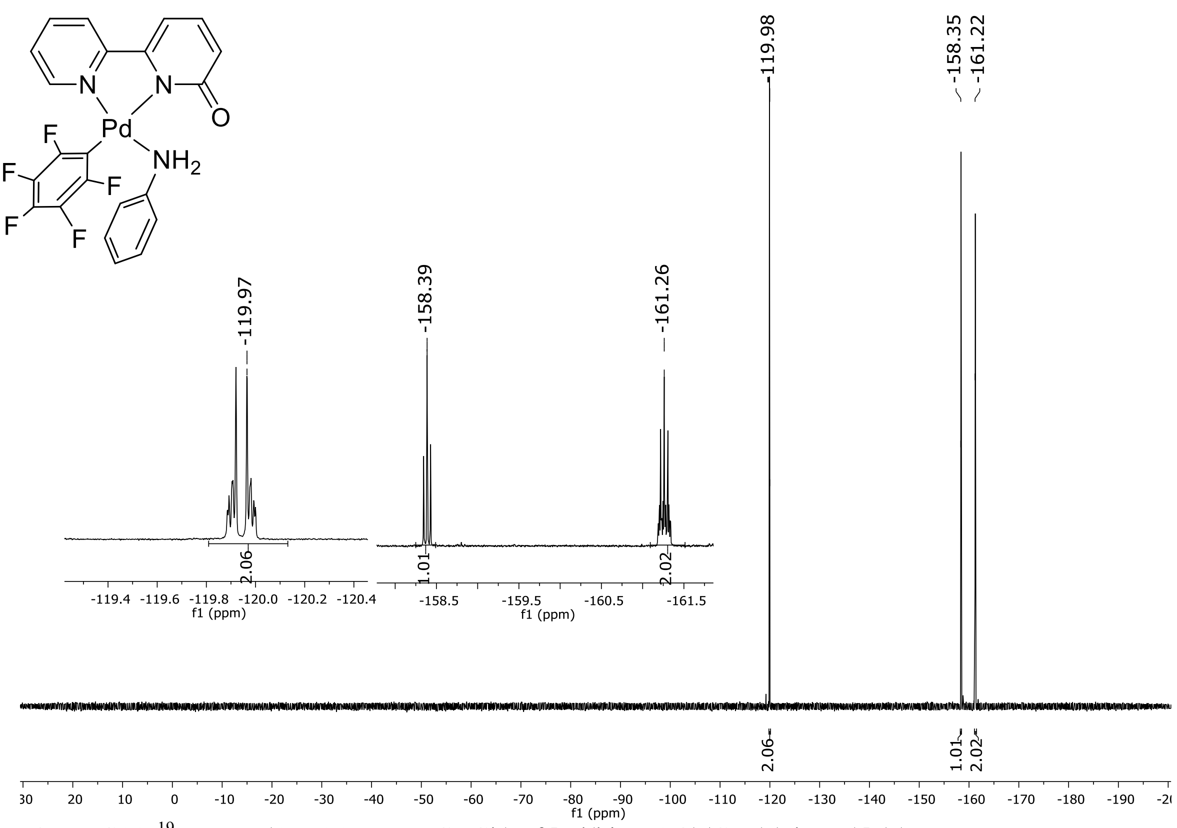

**Figure S14.** $^{19}F$ NMR (470.168 MHz, $CDCl_3$) of [Pd(bipy-6-O)($C_6F_5$)($PhNH_2$)] (**7**) at 298 K.

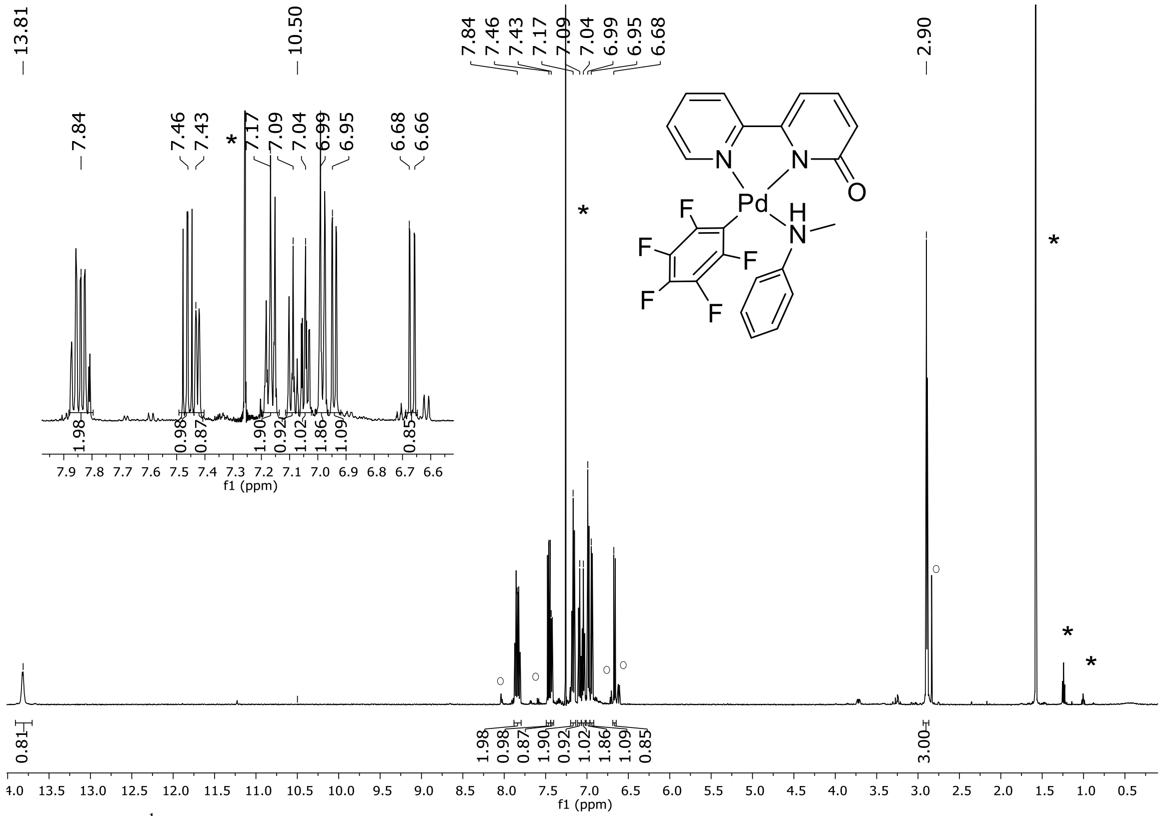

**Figure S15.** $^{1}H$ NMR (500.13 MHz, $CDCl_3$) of [Pd(bipy-6-O)($C_6F_5$)(PhNHMe)] (**9**) at 298 K. (*)Signal corresponding to solvent (chloroform, hexane and $H_2O$). (○) Signal corresponding to a minor isomer.

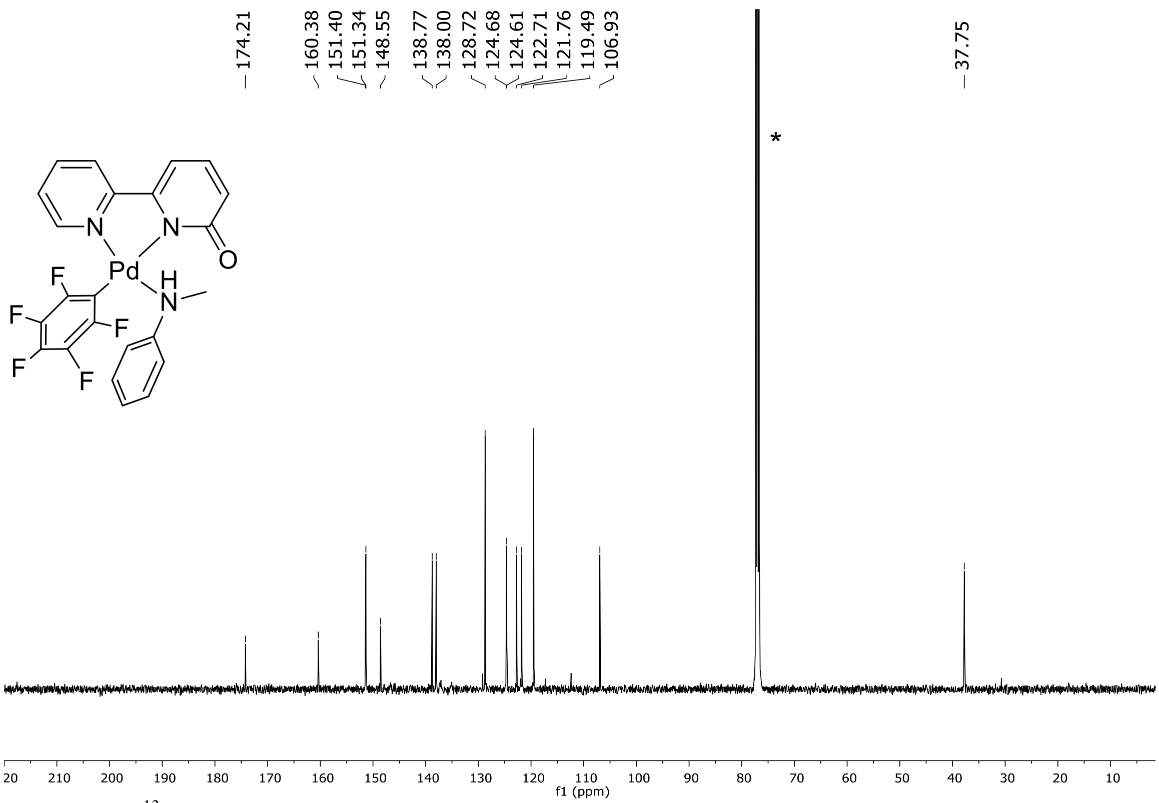


**Figure S16.** $^{13}$C NMR (125.87 MHz, $CDCl_3$) of [Pd(bipy-6-O)($C_6F_5$)(PhNHMe)] (**9**) at 298 K. (*) Signal corresponding to solvent (chloroform).

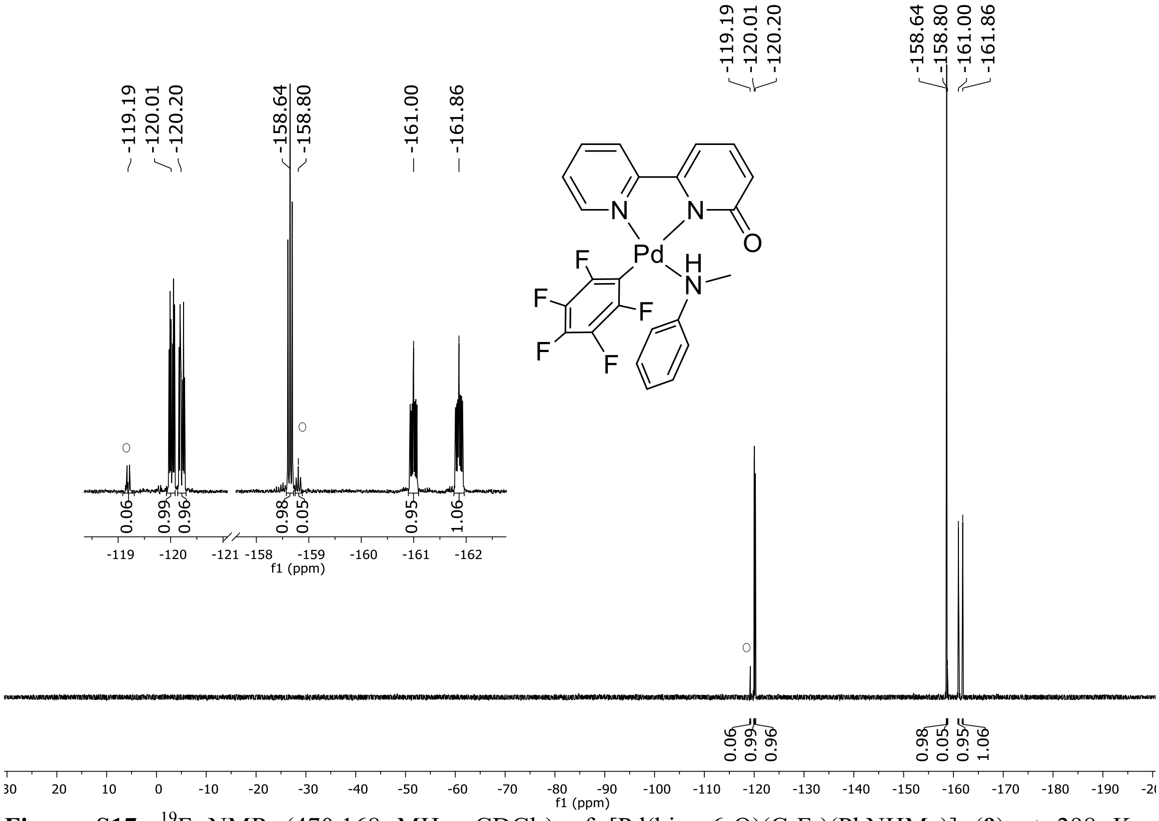


**Figure S17.** $^{19}$F NMR (470.168 MHz, $CDCl_3$) of [Pd(bipy-6-O)($C_6F_5$)(PhNHMe)] (**9**) at 298 K. (○) Signals corresponding to a minor isomer.

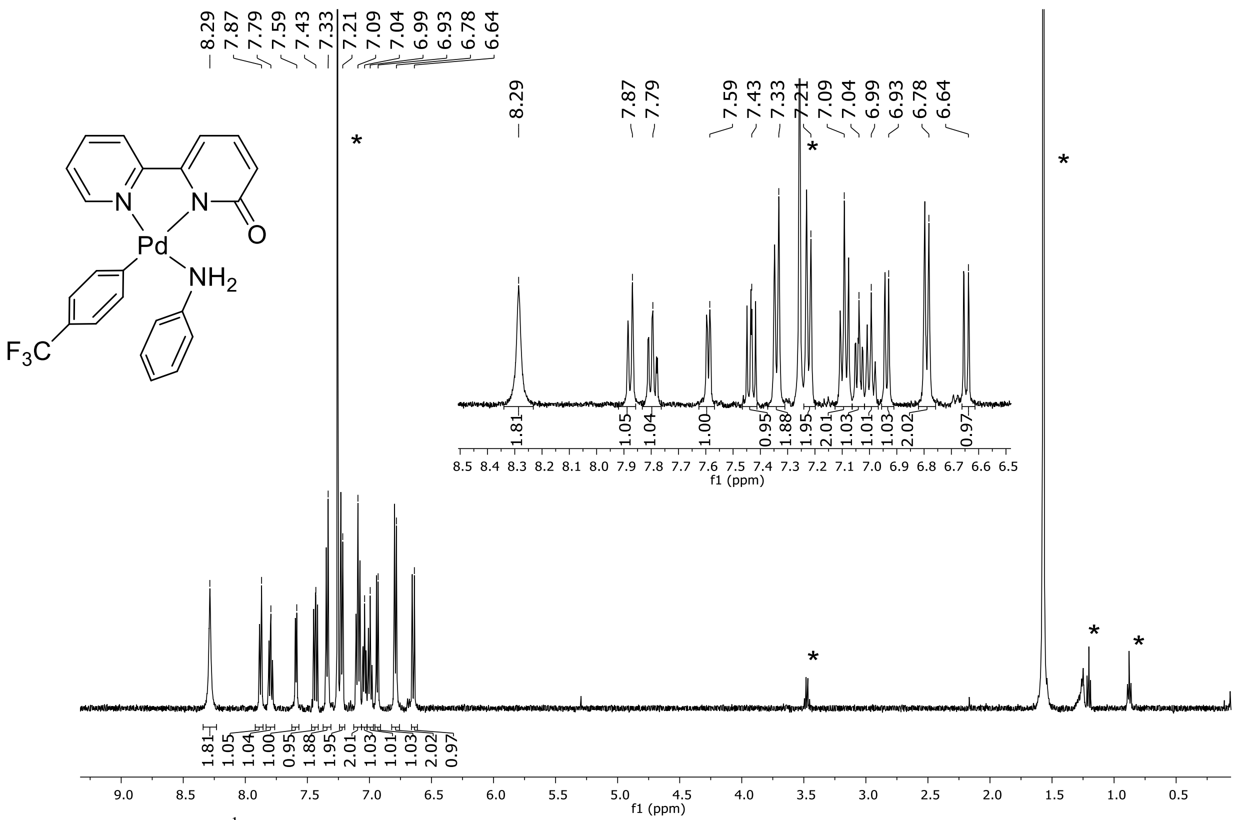


**Figure S18.** $^{1}$H NMR (500.13 MHz, CDCl$_3$) of [Pd(bipy-6-O)(C$_6$H$_4$-p-CF$_3$)(PhNH$_2$)] (**8**) at 298 K. (*) Signal corresponding to solvent (chloroform, Et$_2$O, hexane and H$_2$O).

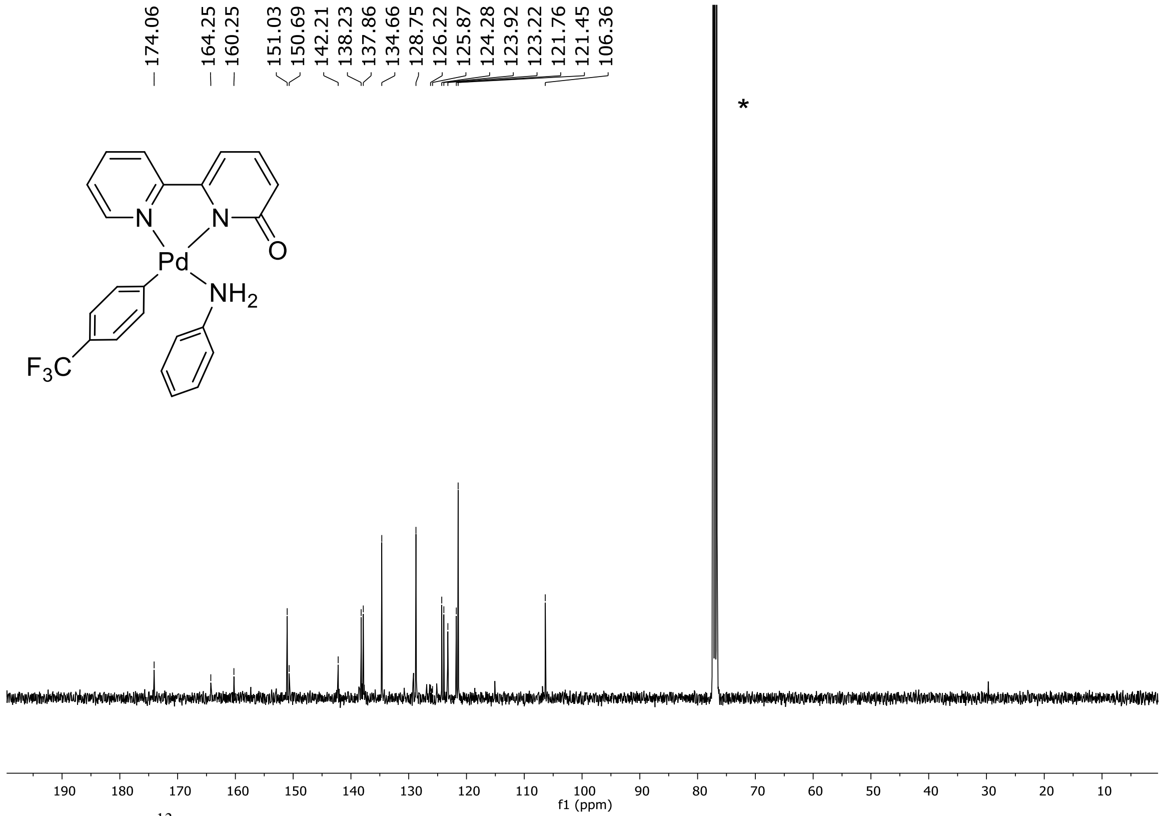


**Figure S19.** $^{13}$C NMR (125.87 MHz, CDCl$_3$) of [Pd(bipy-6-O)(C$_6$H$_4$-p-CF$_3$)(PhNH$_2$)] (**8**) at 298 K. (*) Signal corresponding to solvent (chloroform).

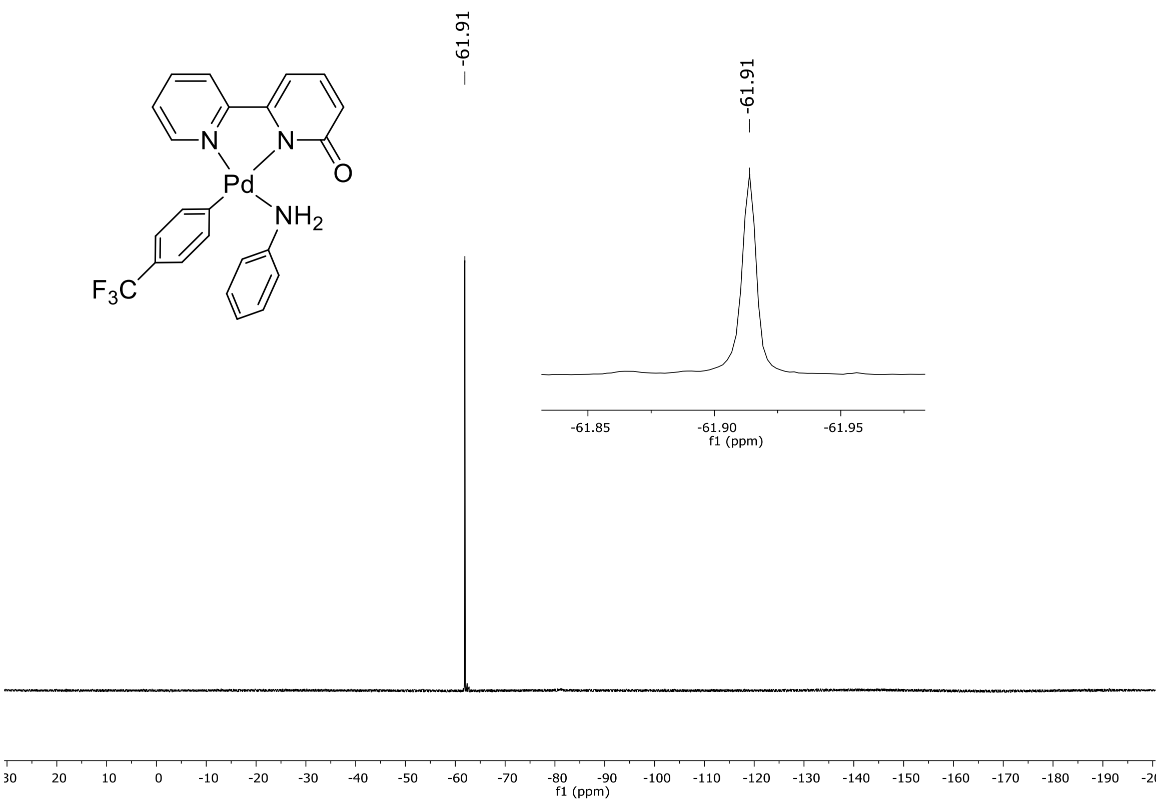


**Figure S20.** $^{19}$F NMR (470.168 MHz, $CDCl_3$) of [Pd(bipy-6-O)($C_6H_4$-p-$CF_3$)($PhNH_2$)] (**8**) at 298 K.

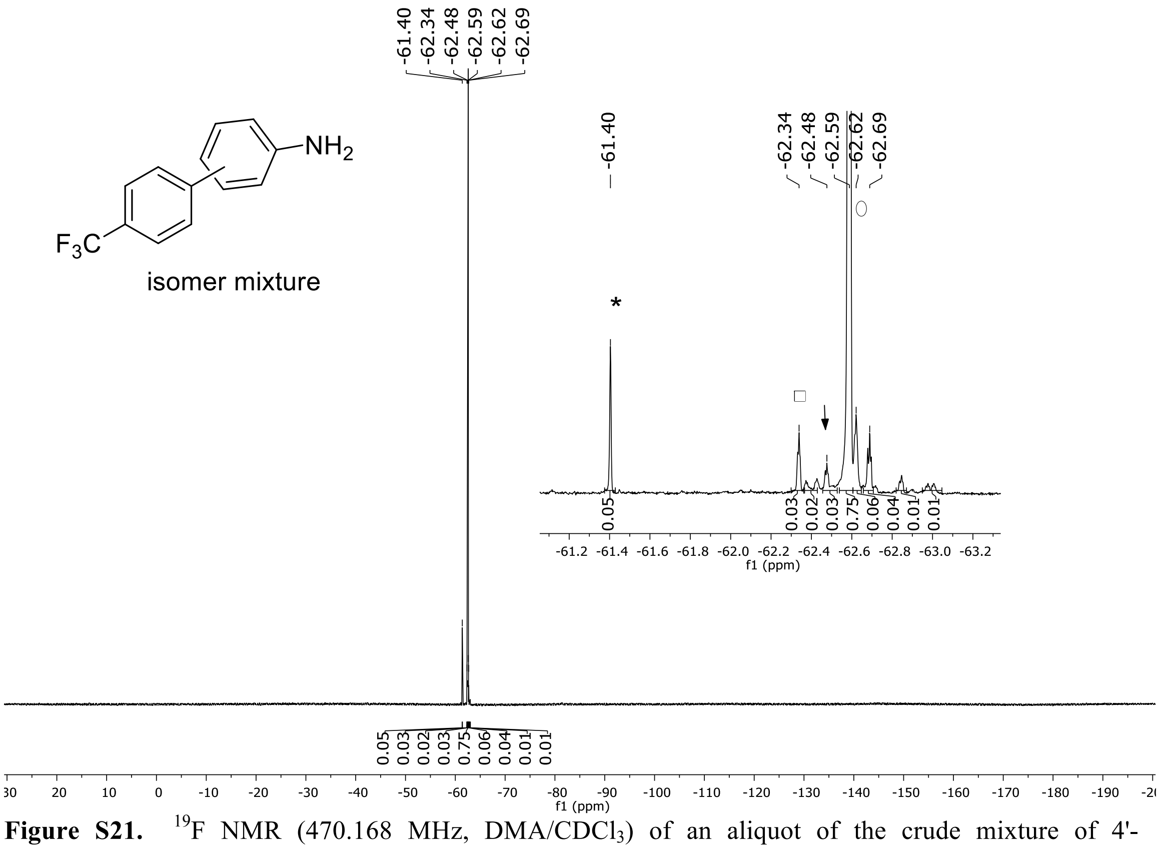


**Figure S21.** $^{19}$F NMR (470.168 MHz, DMA/$CDCl_3$) of an aliquot of the crude mixture of 4'-(trifluoromethyl)-[1,1'-biphenyl]-2-amine (○), 4'-(trifluoromethyl)-[1,1'-biphenyl]-3-amine (↓) and 4'-(trifluoromethyl)-[1,1'-biphenyl]-4-amine (□) (isomer ratio o:m:p = 25:1:1). Small amounts of the C-N coupling product (5 %, *), reduction and aryl homocoupling products are also observed.

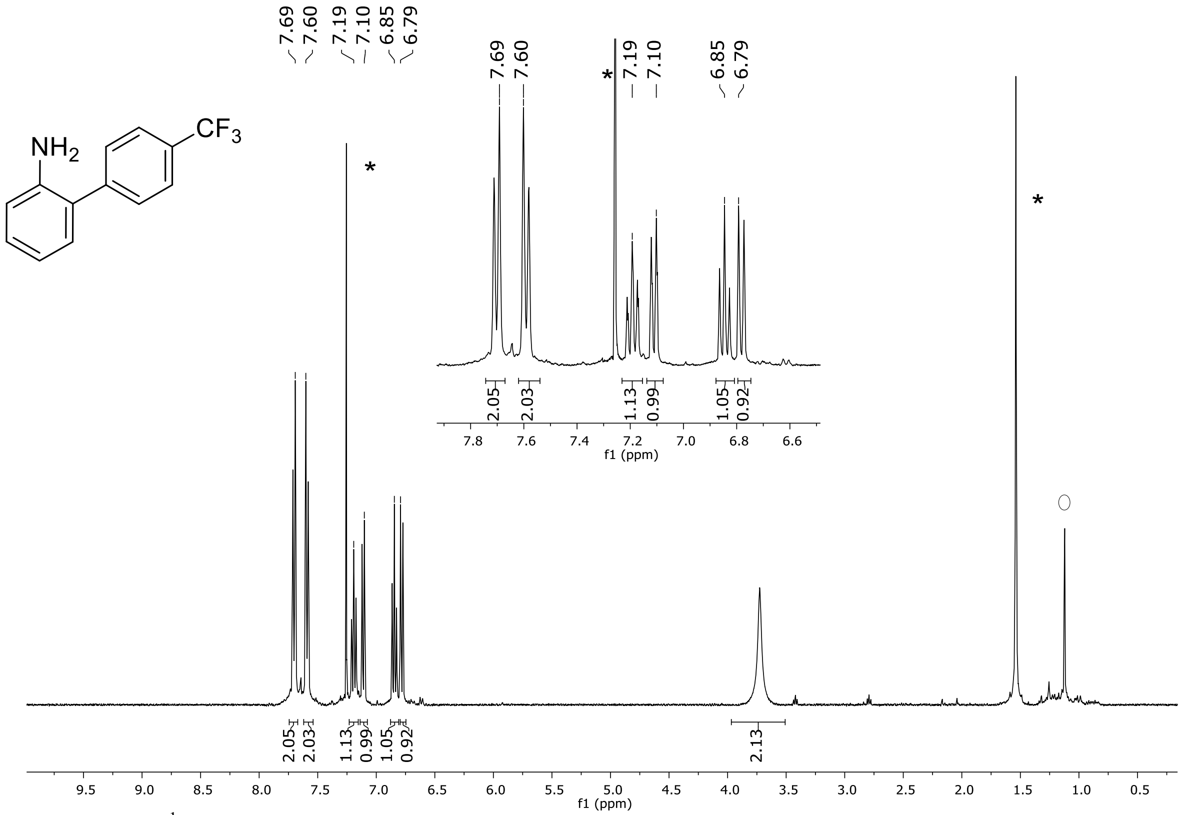


**Figure S22.** $^{1}$H NMR (500.13 MHz, $CDCl_3$) of 4'-(trifluoromethyl)-[1,1'-biphenyl]-2-amine (**5a**) at 298 K. (*) Signal corresponding to solvent (chloroform and $H_2O$). (○) Residual high MW paraffin.

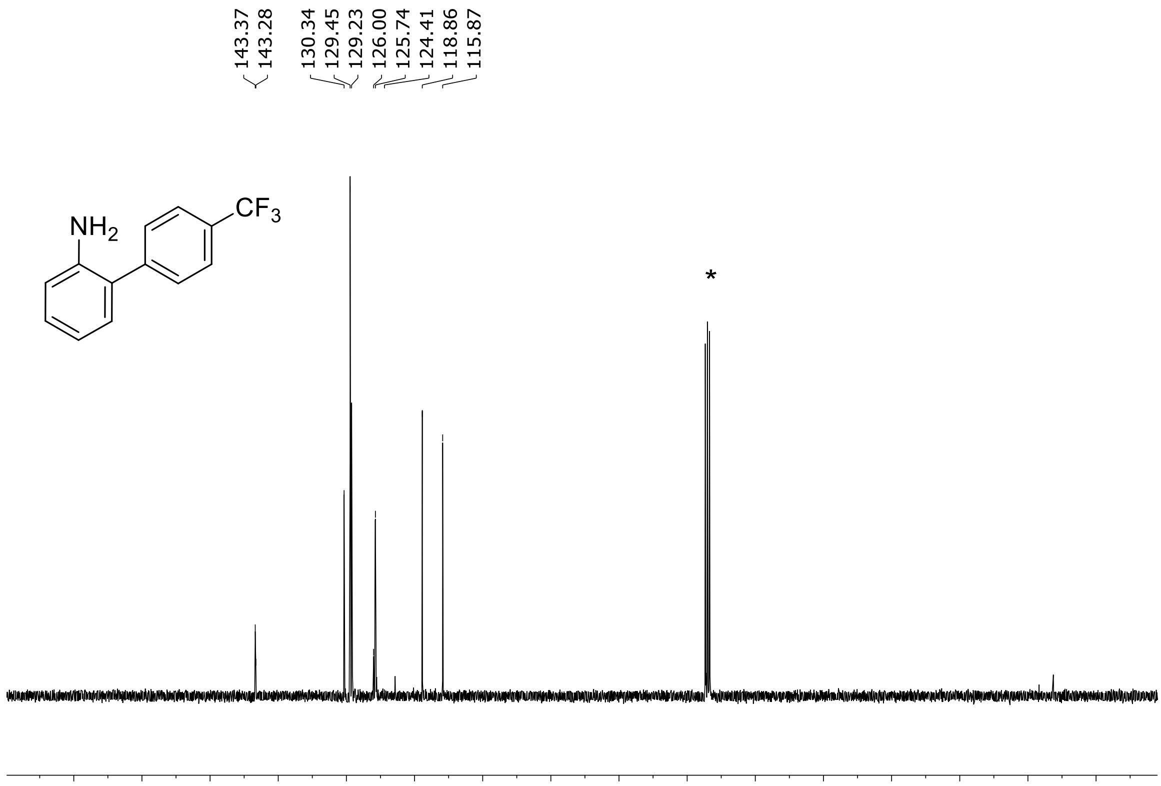


**Figure S23.** $^{13}$C NMR (125.87 MHz, $CDCl_3$) of 4'-(trifluoromethyl)-[1,1'-biphenyl]-2-amine (**5a**) at 298 K. (*) Signal corresponding to solvent (chloroform).

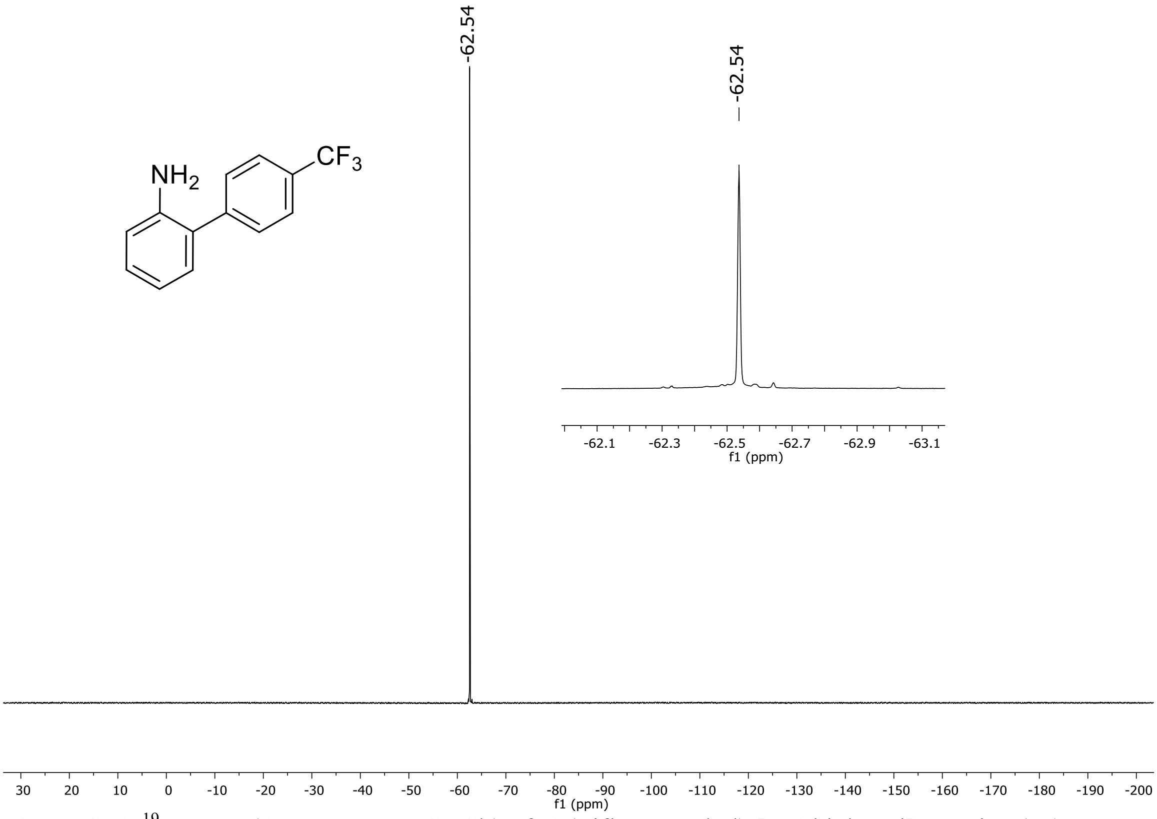


**Figure S24.** $^{19}$F NMR (470.168 MHz, $CDCl_3$) of 4'-(trifluoromethyl)-[1,1'-biphenyl]-2-amine (**5a**) at 298 K.

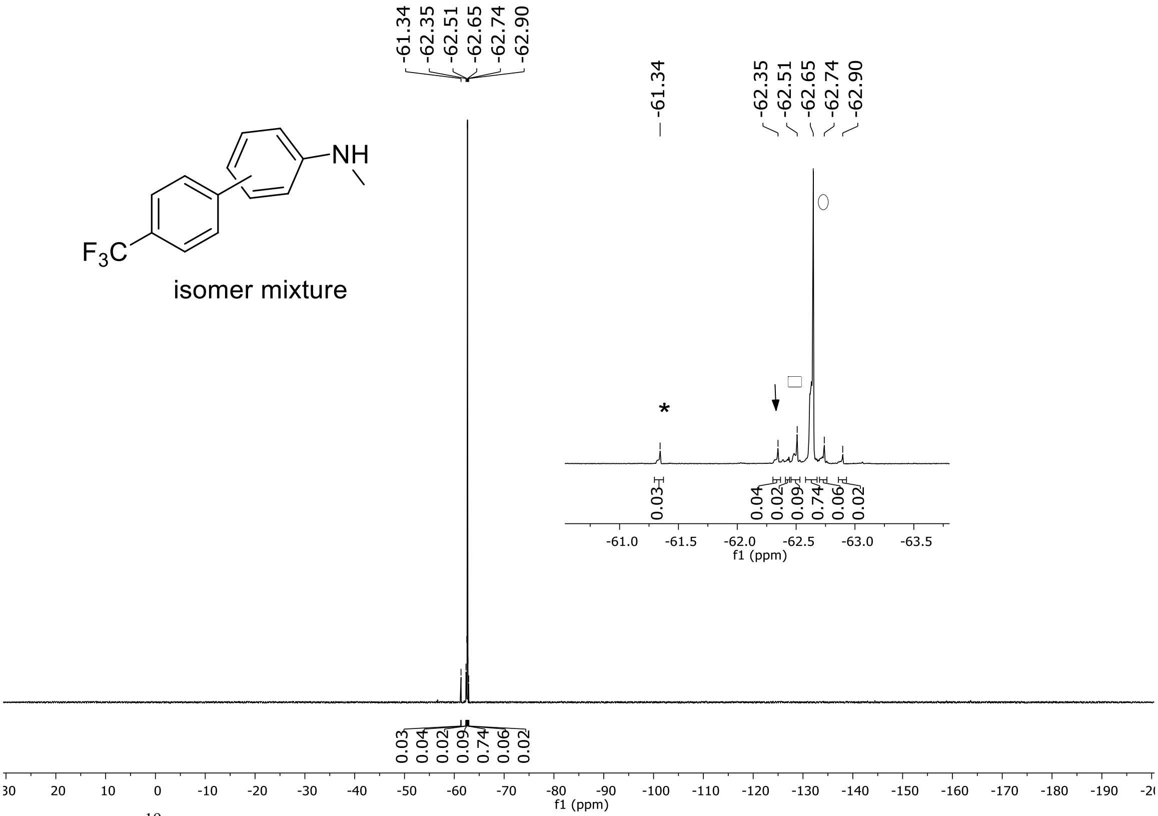


**Figure S25.** $^{19}$F NMR (470.168 MHz, DMA/$CDCl_3$) of an aliquot of the crude mixture of N-methyl-4'-(trifluoromethyl)[1,1'-biphenyl]-2-amine (○), N-methyl-4'-(trifluoromethyl)[1,1'-biphenyl]-3-amine (□) and N-methyl-4'-(trifluoromethyl)[1,1'-biphenyl]-4-amine (↓) (isomer ratio o:m:p = 18:2:1). Small amounts of the C-N coupling product (*), reduction and aryl homocoupling products are also observed.

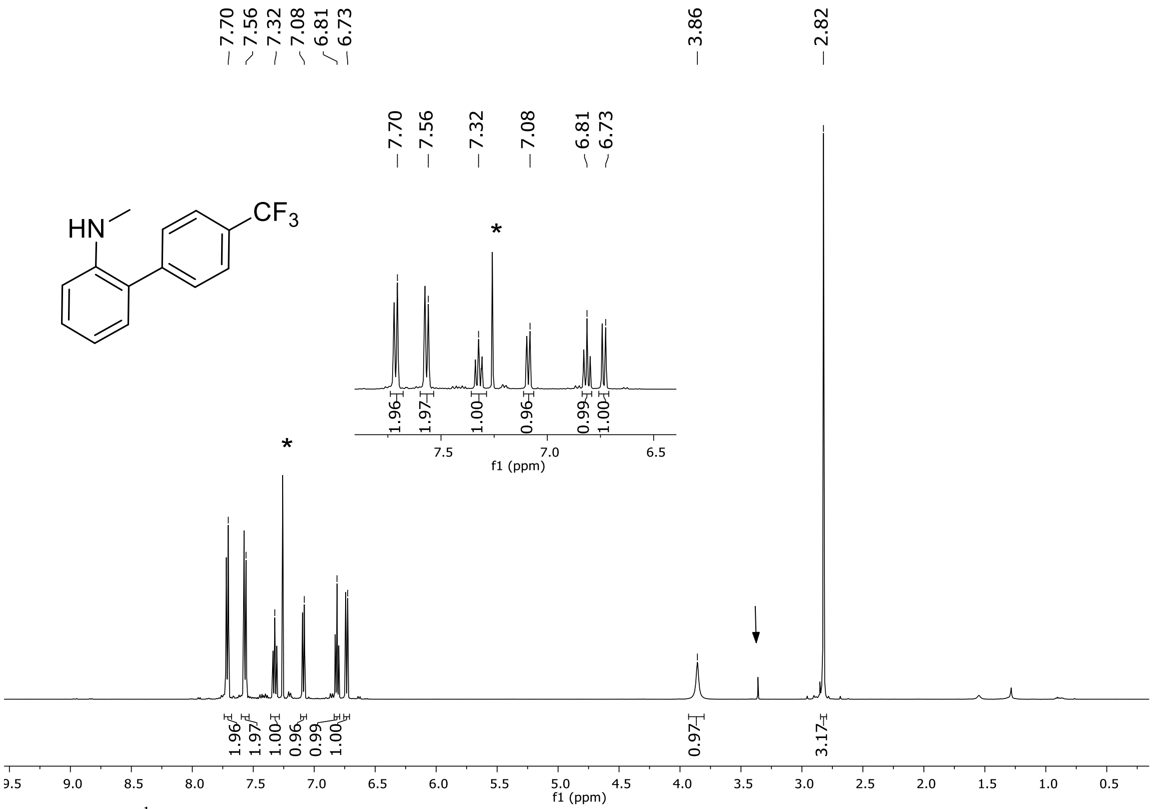


**Figure S26.** $^{1}H$ NMR (500.13 MHz, $CDCl_3$) of N-methyl-4'-(trifluoromethyl)[1,1'-biphenyl]-2-amine (**5l**) at 298 K. (*) Signal corresponding to solvent (chloroform). (↓) Signal corresponding to C-N coupling product.

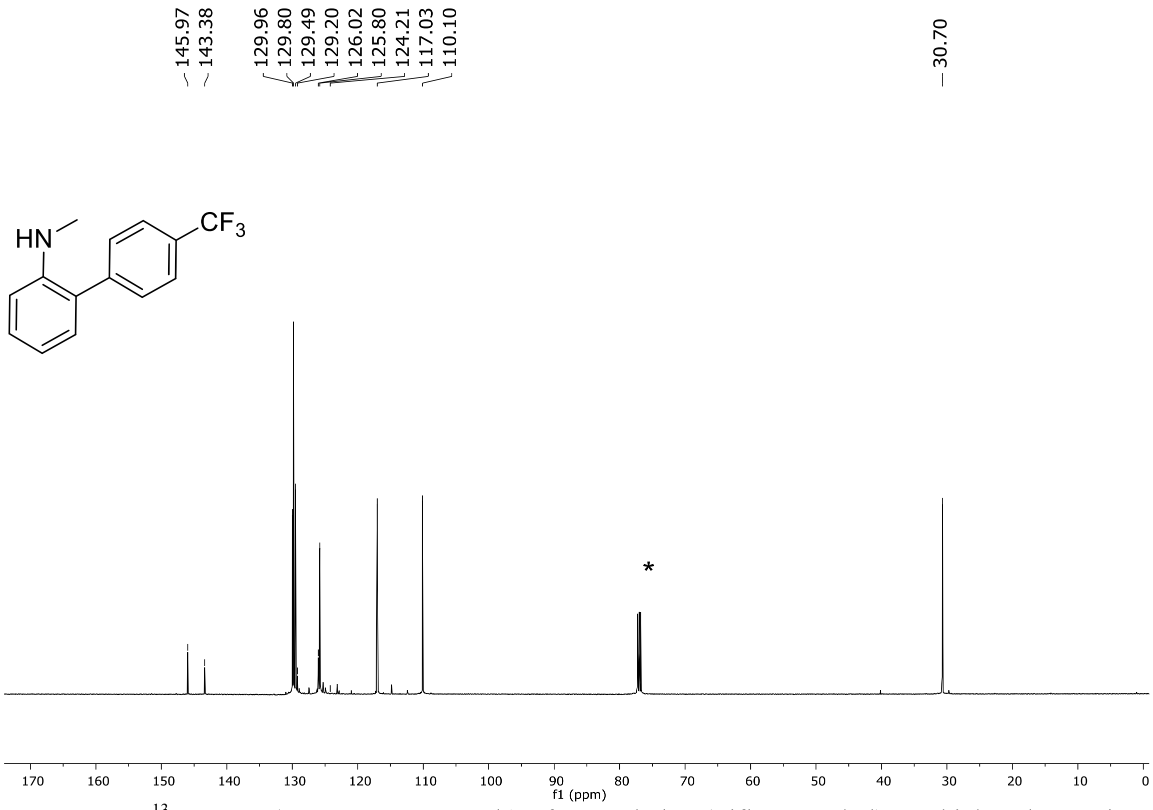


**Figure S27.** $^{13}C$ NMR (125.87 MHz, $CDCl_3$) of N-methyl-4'-(trifluoromethyl)[1,1'-biphenyl]-2-amine (**5l**) at 298 K. (*) Signal corresponding to solvent (chloroform).

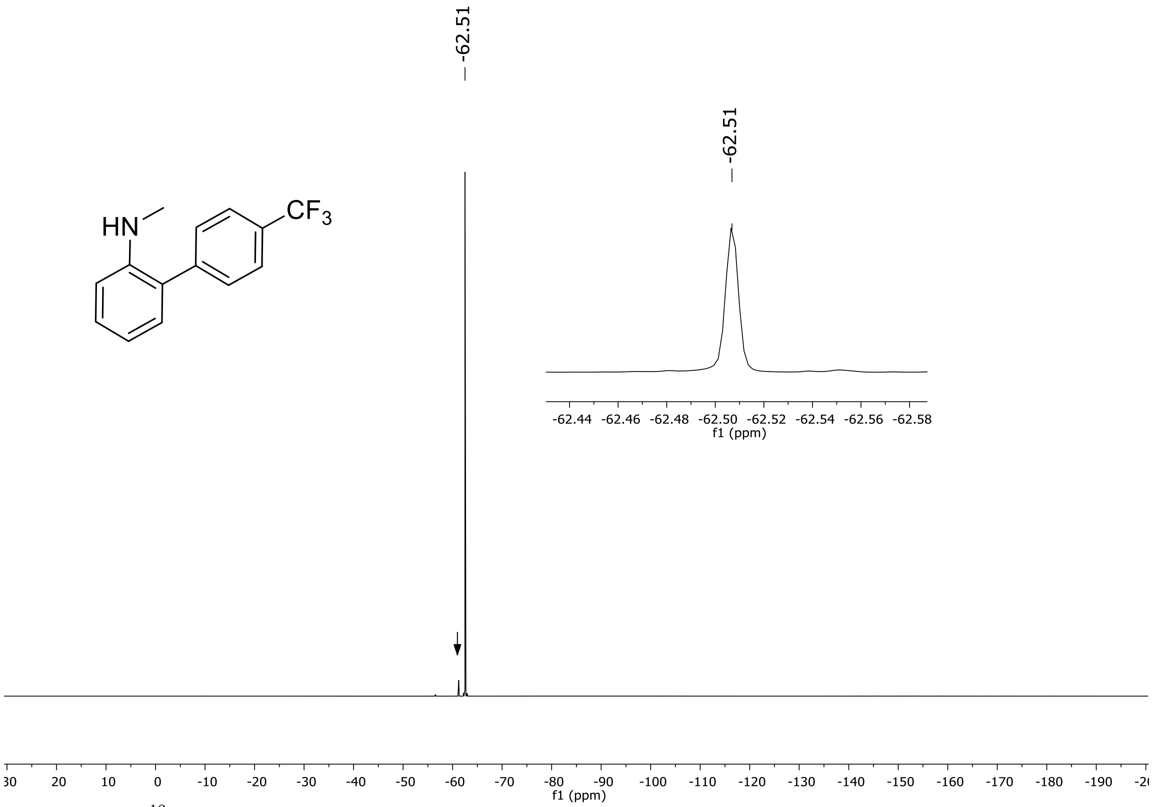


**Figure S28.** $^{19}F$ NMR (470.168 MHz, $CDCl_3$) of N-methyl-4'-(trifluoromethyl)[1,1'-biphenyl]-2-amine (**5l**) at 298 K. (↓) Signal corresponding to C-N coupling product.

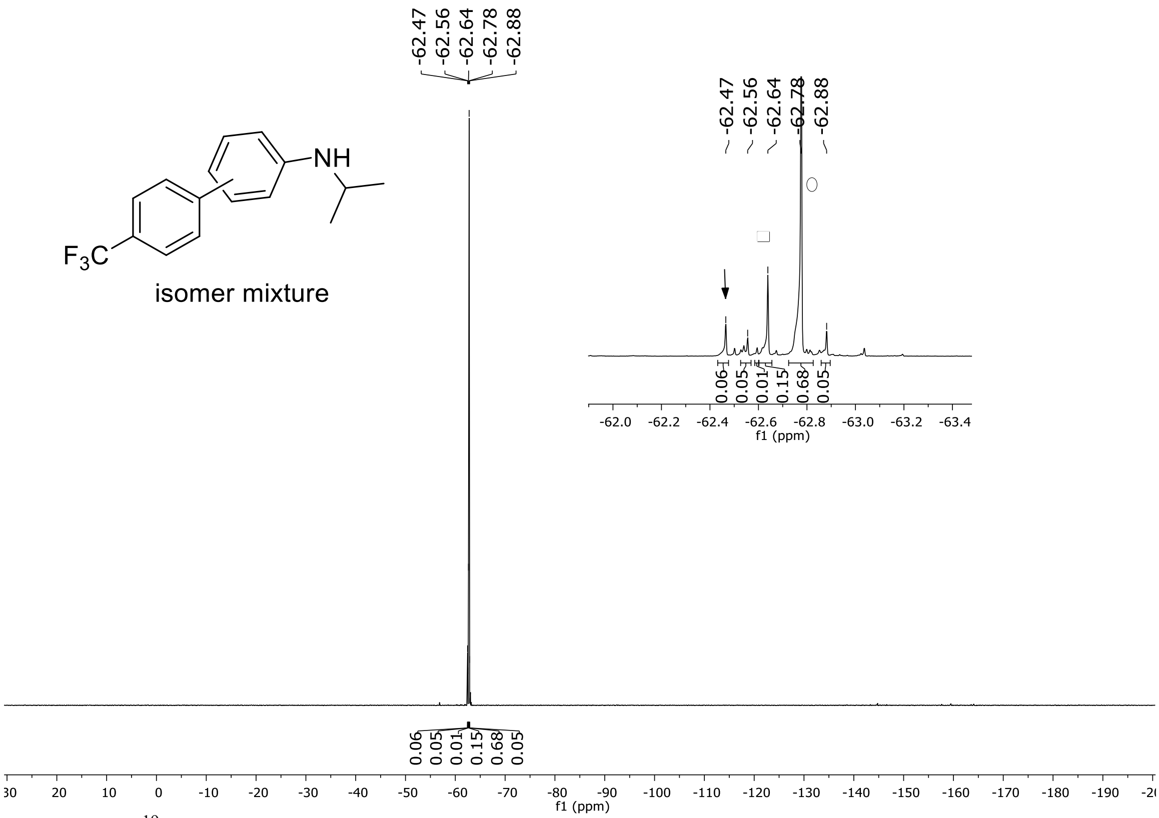


**Figure S29.** $^{19}F$ NMR (470.168 MHz, DMA/$CDCl_3$) of an aliquot of the crude mixture of N-isopropyl-4'-(trifluoromethyl)[1,1'-biphenyl]-2-amine (○), N-isopropyl-4'-(trifluoromethyl)[1,1'-biphenyl]-3-amine (□), N-isopropyl-4'-(trifluoromethyl)[1,1'-biphenyl]-4-amine (↓) (isomer ratio o:m:p = 12:3:1). The aryl reduction product and other minor products are observed.

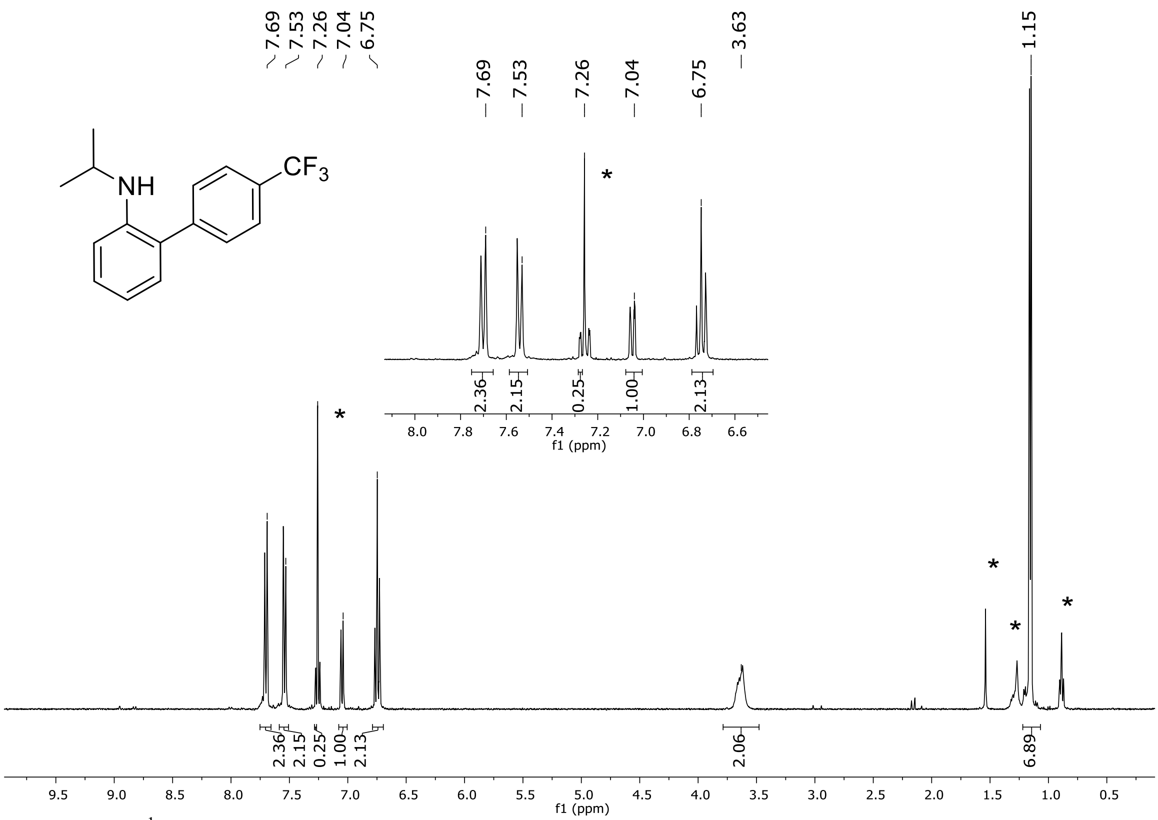


**Figure S30.** $^{1}H$ NMR (500.13 MHz, $CDCl_3$) of N-isopropyl-4'-(trifluoromethyl)[1,1'-biphenyl]-2-amine (**5m**) at 298 K. (*) Signals corresponding to solvent (chloroform, $H_2O$ and hexane).

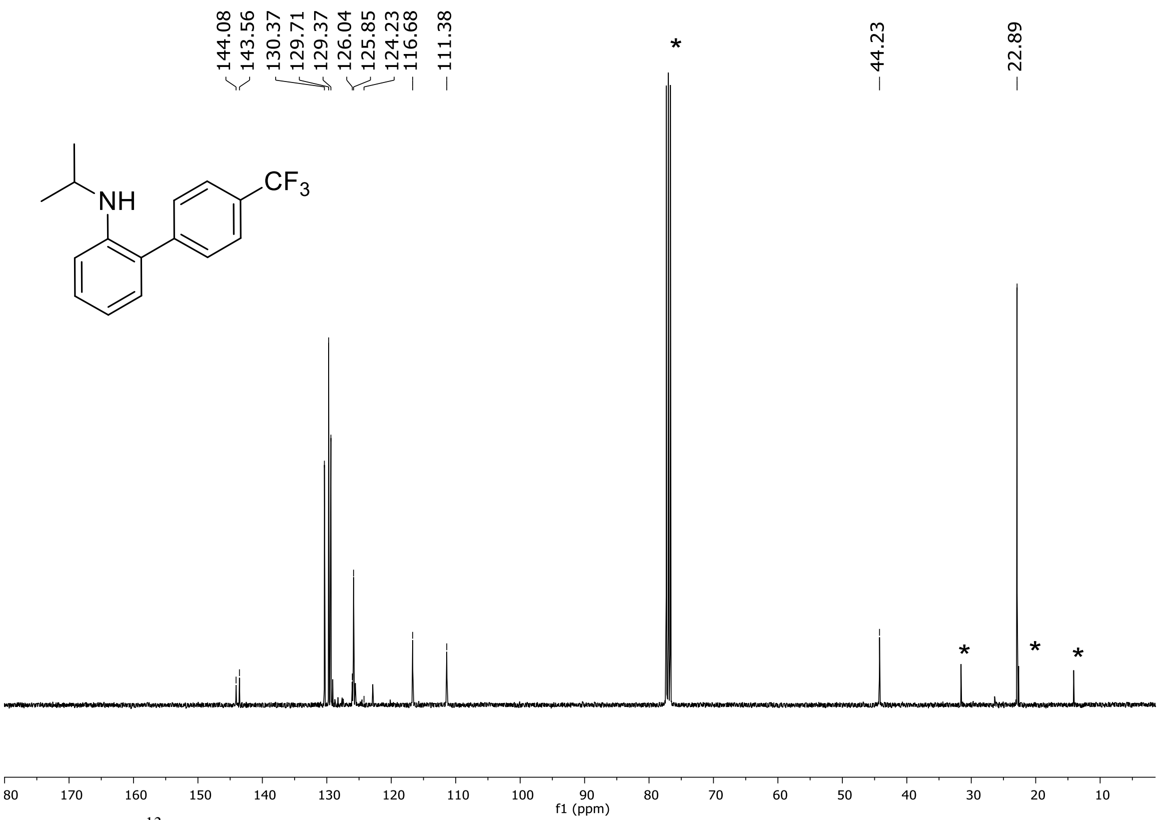


**Figure S31.** $^{13}C$ NMR (125.87 MHz, $CDCl_3$) of N-isopropyl-4'-(trifluoromethyl)[1,1'-biphenyl]-2-amine (**5m**) at 298 K. (*) Signals corresponding to solvent (chloroform and hexane).

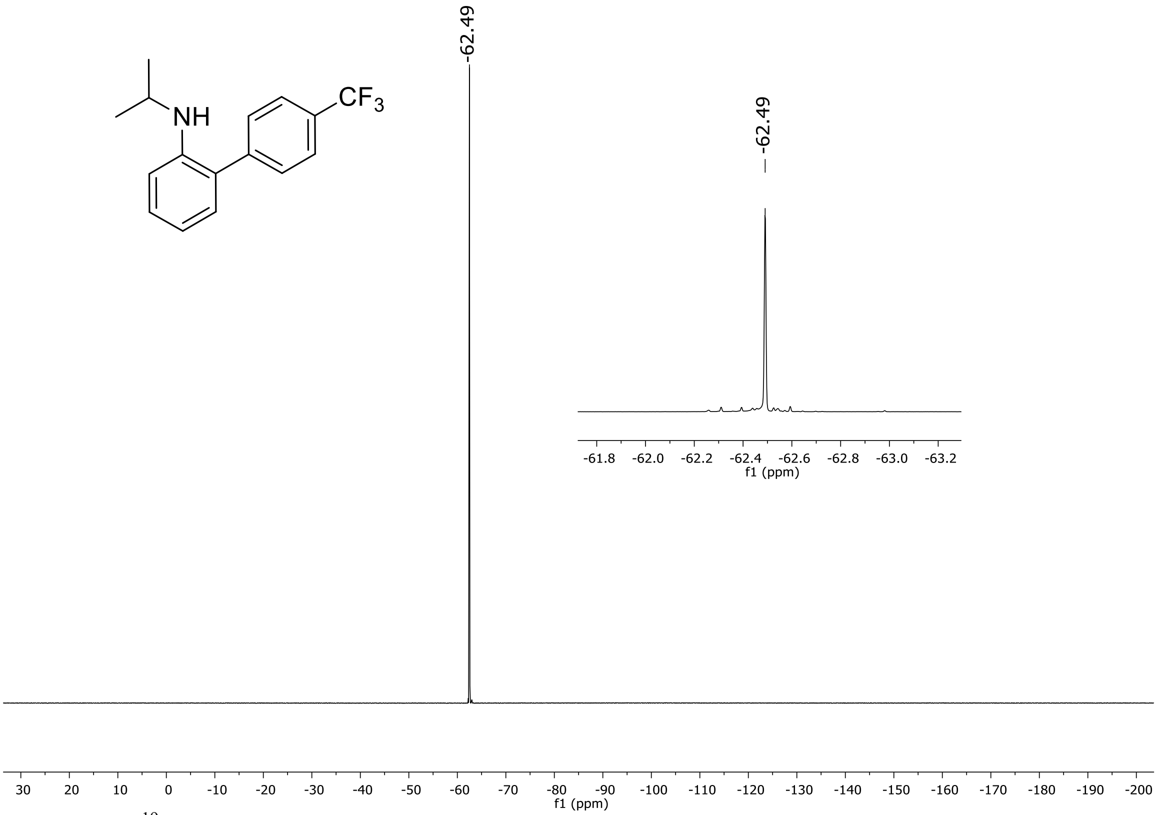


**Figure S32**. $^{19}$F NMR (470.168 MHz, $CDCl_3$) of N-isopropyl-4'-(trifluoromethyl)[1,1'-biphenyl]-2-amine (**5m**) at 298 K.

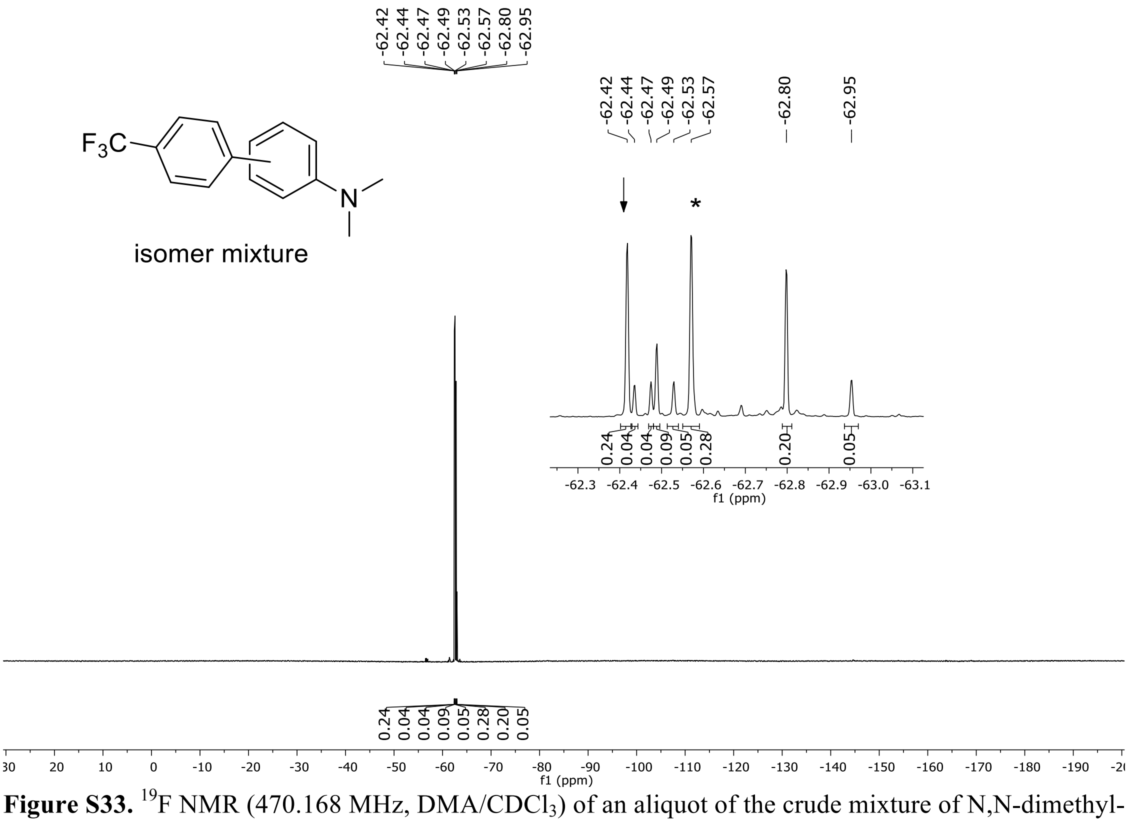


**Figure S33.** $^{19}$F NMR (470.168 MHz, DMA/$CDCl_3$) of an aliquot of the crude mixture of N,N-dimethyl-4'-(trifluoromethyl)[1,1'-biphenyl]-3-amine (*) and N,N-dimethyl-4'-(trifluoromethyl)[1,1'-biphenyl]-4-amine (↓) (isomer ratio m:p = 1.2:1). The aryl iodide, aryl reduction product and other minor products are observed.

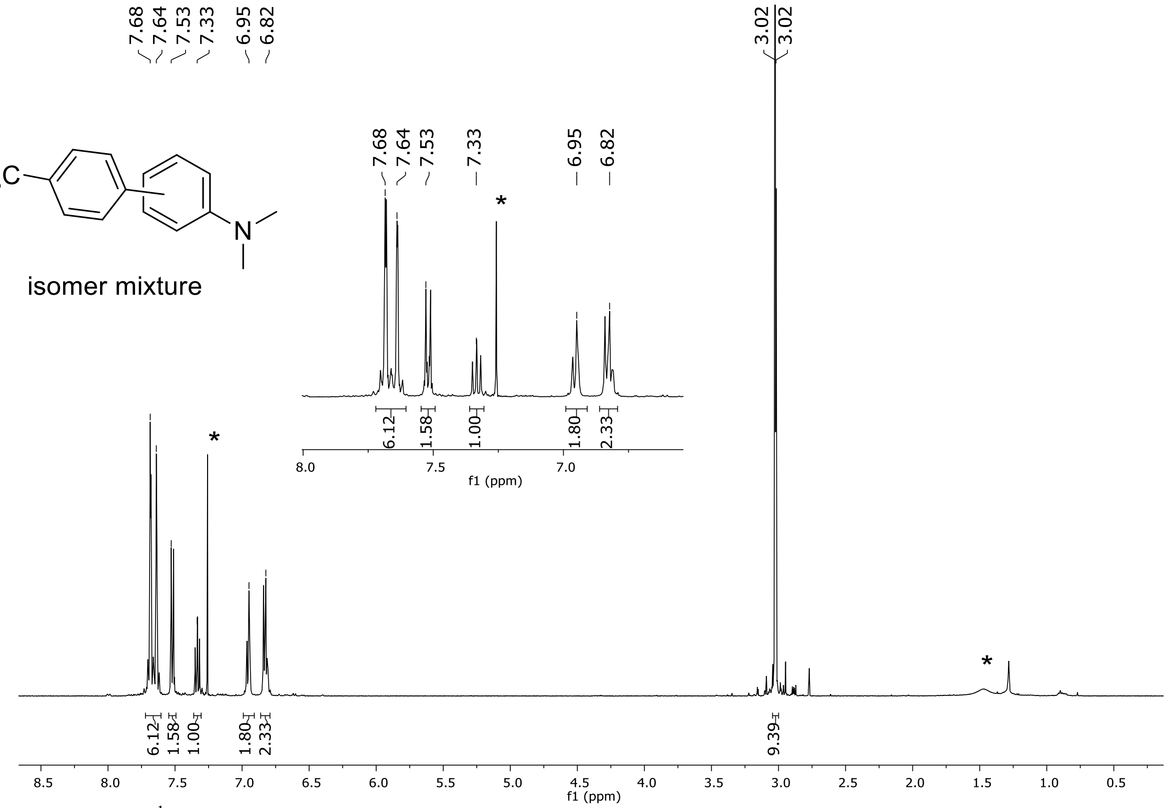


**Figure S34**. $^{1}$H NMR (500.13 MHz, $CDCl_3$) of N,N-dimethyl-4'-(trifluoromethyl)[1,1'-biphenyl]-3-amine and N,N-dimethyl-4'-(trifluoromethyl)[1,1'-biphenyl]-4-amine (**5n**) at 348 K. Isomers in the ratio m:p = 1.2:1. (*) Signal corresponding to solvent (chloroform and $H_2O$).

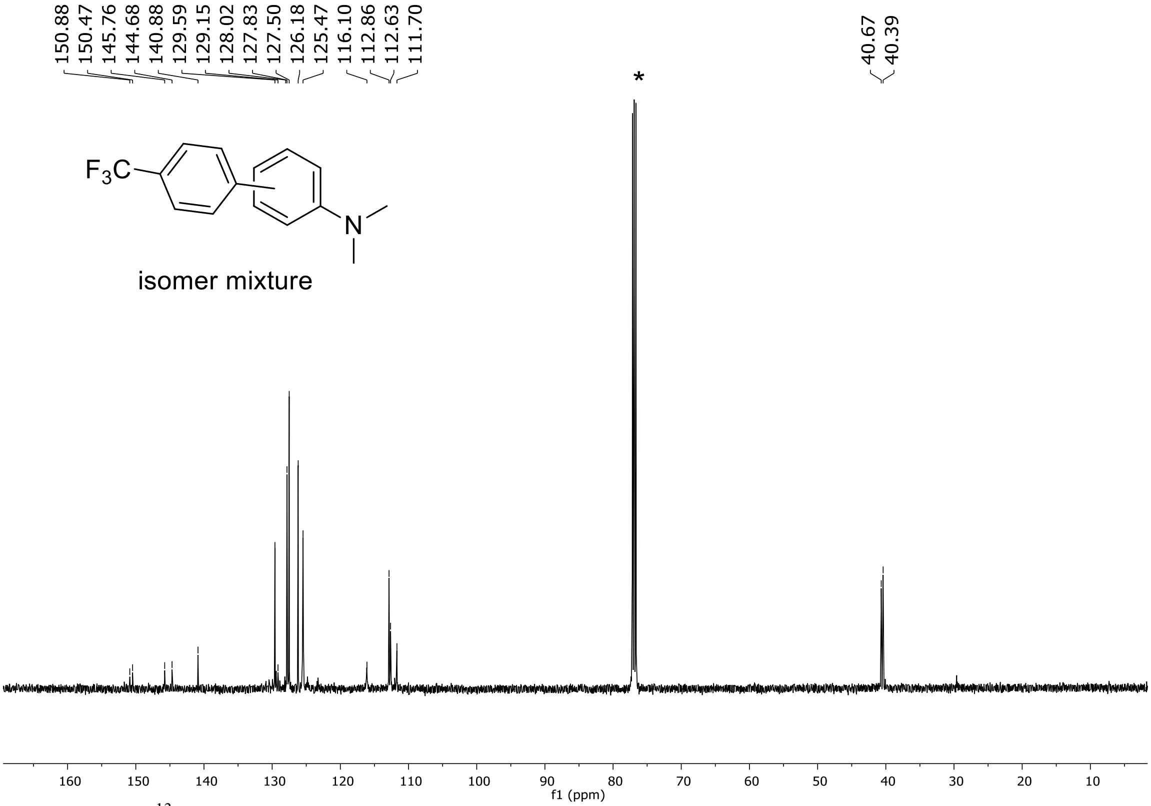


**Figure S35.** $^{13}$C NMR (125.87 MHz, $CDCl_3$) of N,N-dimethyl-4'-(trifluoromethyl)[1,1'-biphenyl]-3-amine and N,N-dimethyl-4'-(trifluoromethyl)[1,1'-biphenyl]-4-amine (**5n**) at 348 K. Isomers in the ratio m:p = 1.2:1. (*) Signal corresponding to solvent (chloroform).

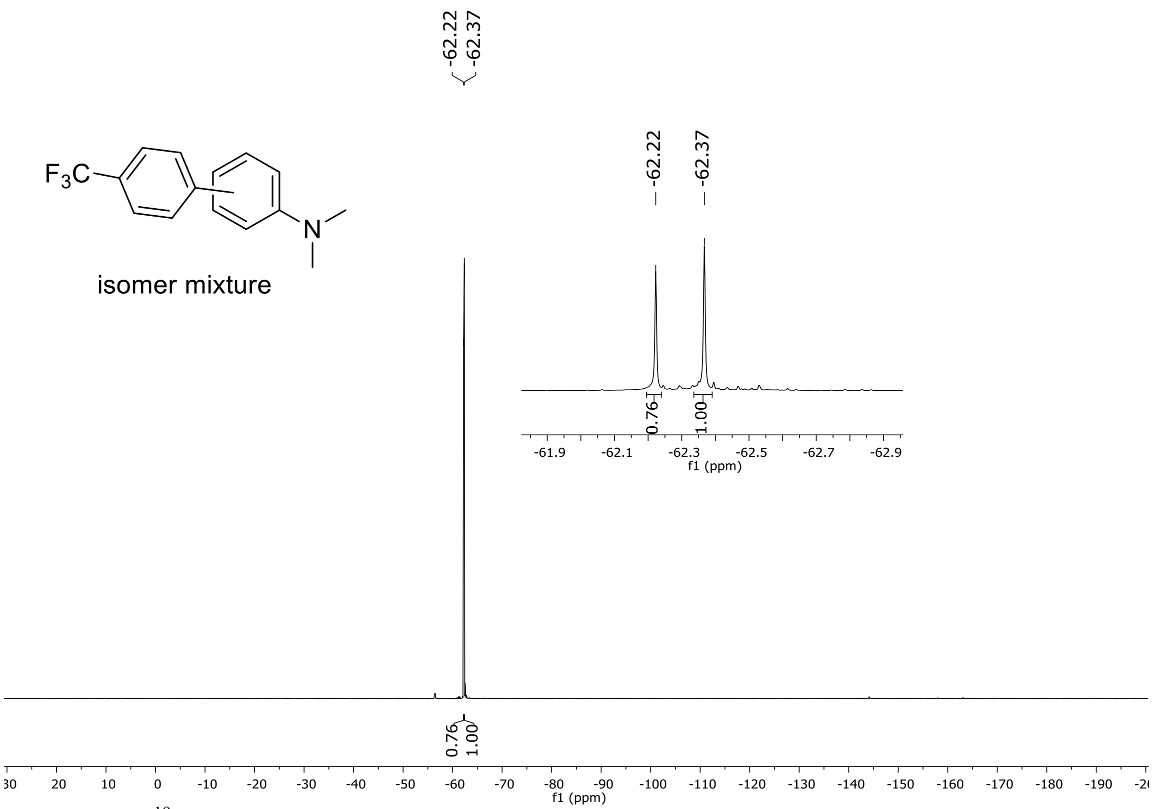


**Figure S36.** $^{19}$F NMR (470.168 MHz, $CDCl_3$) of N,N-dimethyl-4'-(trifluoromethyl)[1,1'-biphenyl]-3-amine and N,N-dimethyl-4'-(trifluoromethyl)[1,1'-biphenyl]-4-amine (**5n**) at 348 K. Isomers in the ratio m:p = 1.2:1.

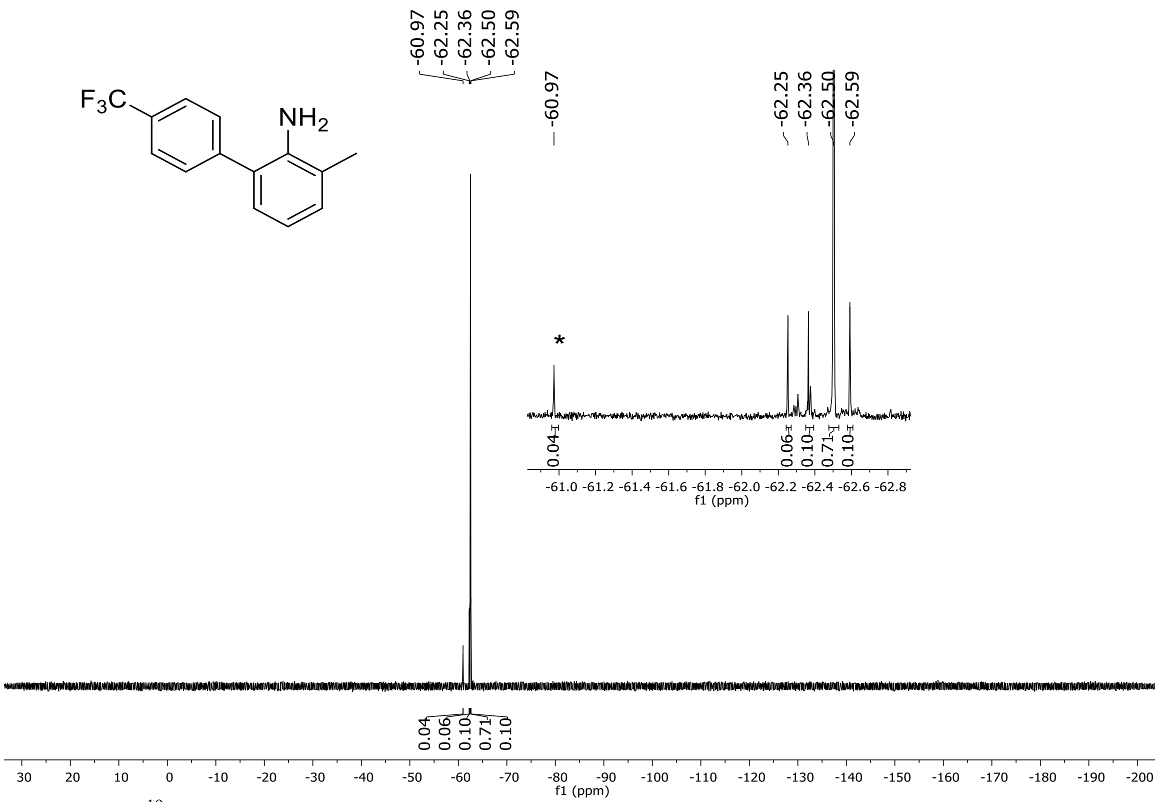


**Figure S37.** $^{19}$F NMR (470.168 MHz, DMA/$CDCl_3$) of an aliquot of the crude mixture of 3-methyl-4'-(trifluoromethyl)-[1,1'-biphenyl]-2-amine. Small amounts of the C-N coupling product (*), aryl homocoupling and other unidentified products are also observed.

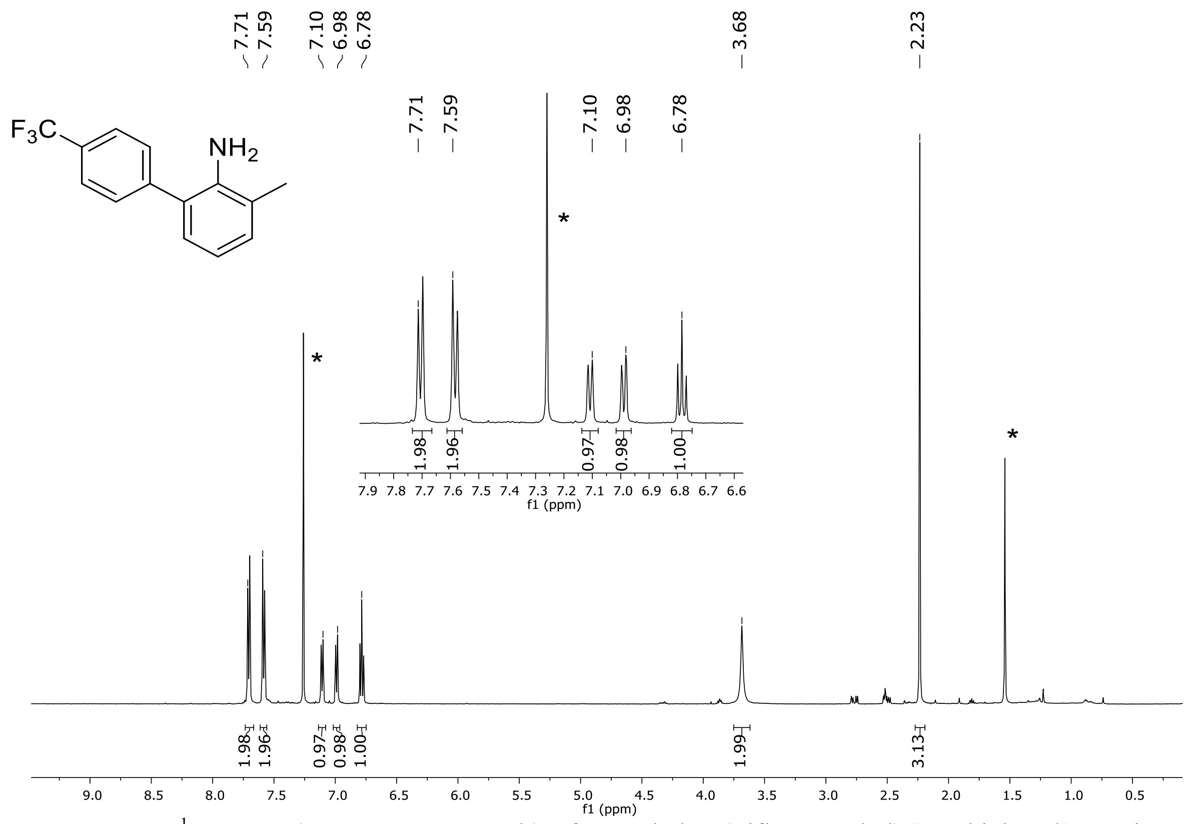


**Figure S38.** $^{1}H$ NMR (500.13 MHz, $CDCl_3$) of 3-methyl-4'-(trifluoromethyl)-[1,1'-biphenyl]-2-amine (**5b**) at 298 K. (*) Signals corresponding to the solvent (chloroform and $H_2O$).

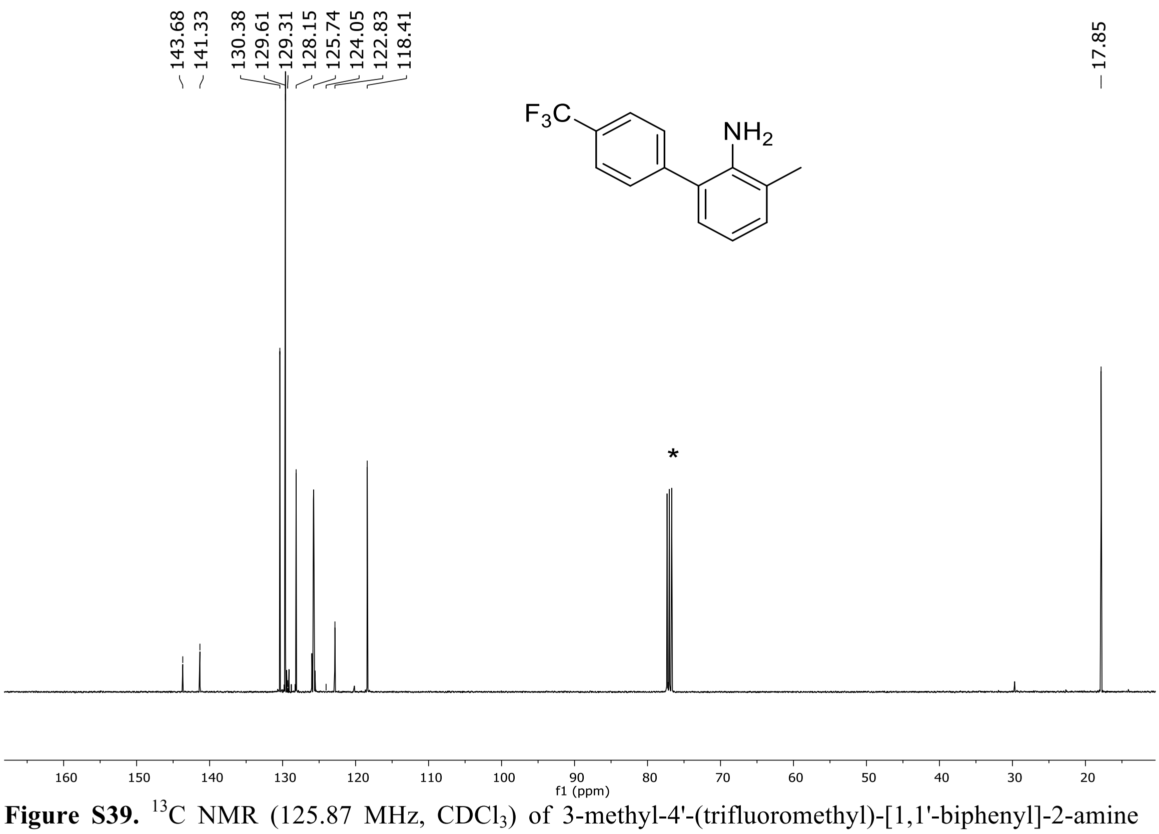


**Figure S39.** $^{13}C$ NMR (125.87 MHz, $CDCl_3$) of 3-methyl-4'-(trifluoromethyl)-[1,1'-biphenyl]-2-amine (**5b**) at 298 K. (*) Signal corresponding to the solvent (chloroform).

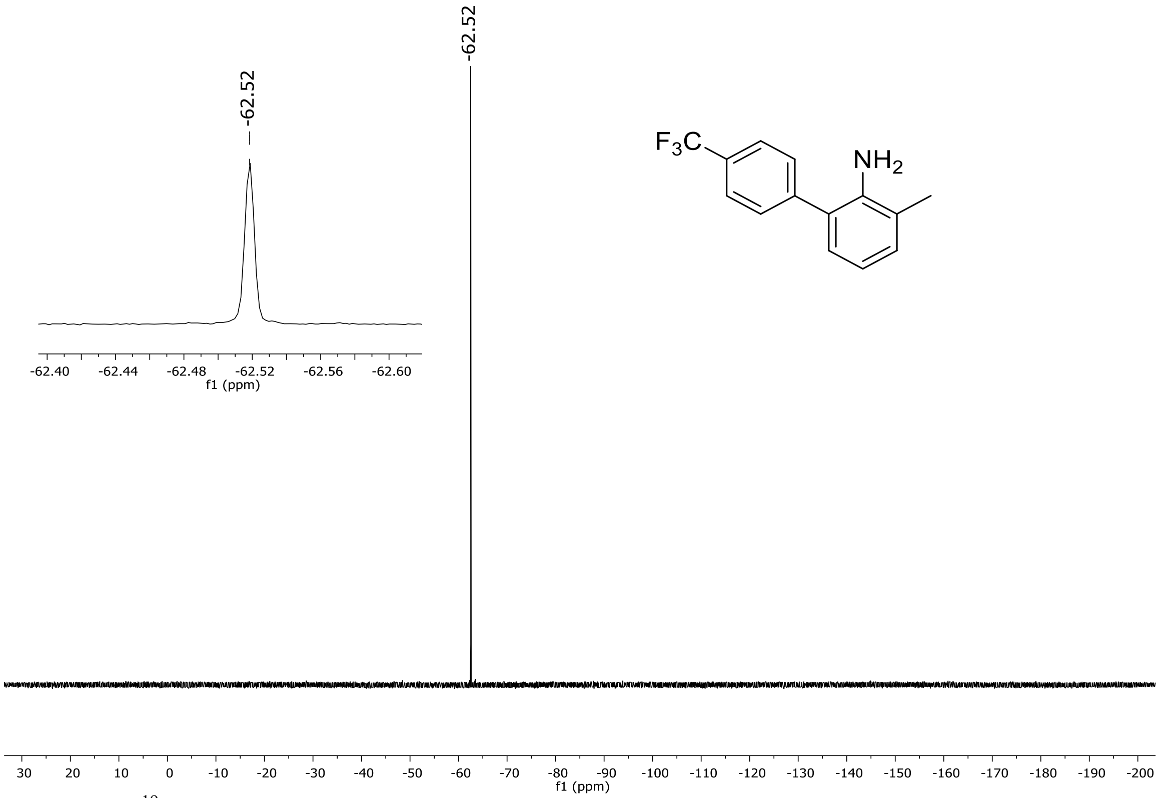


**Figure S40.** $^{19}$F NMR (470.168 MHz, $CDCl_3$) of 3-methyl-4'-(trifluoromethyl)-[1,1'-biphenyl]-2-amine (**5b**) at 298 K.

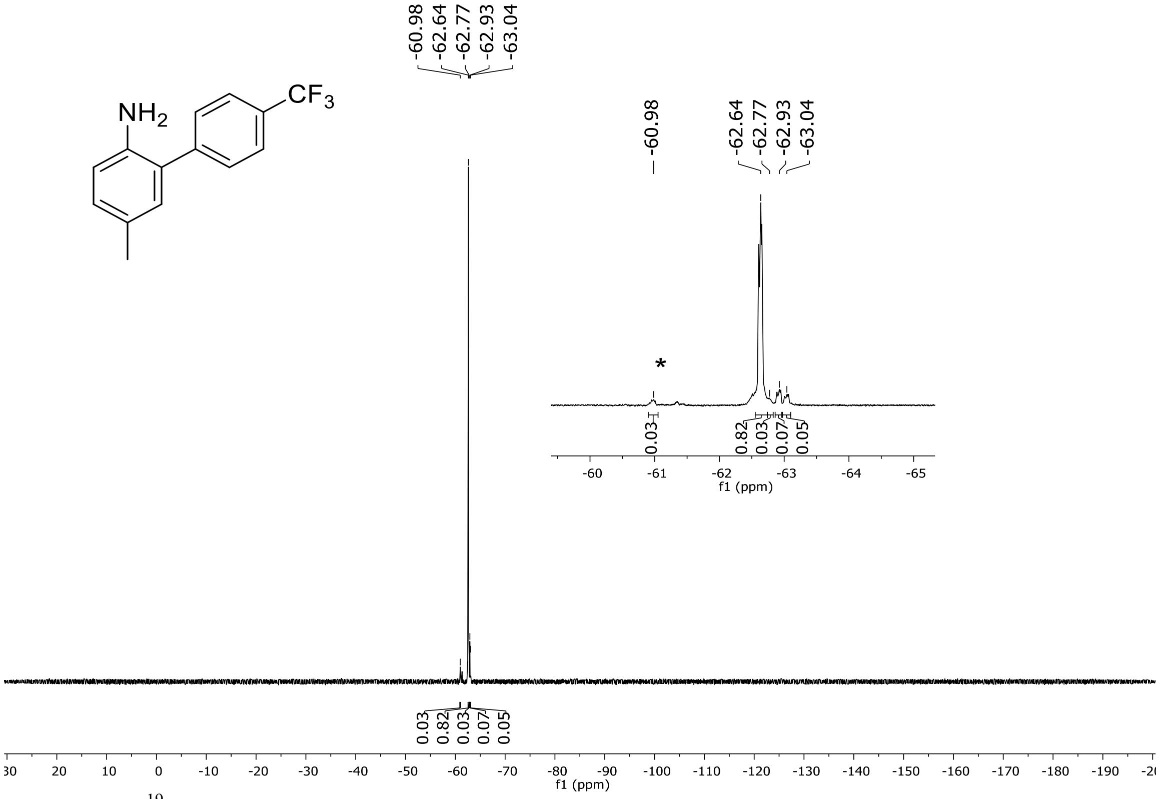


**Figure S41.** $^{19}$F NMR (470.168 MHz, DMA/$CDCl_3$) of an aliquot of the crude mixture of 5-methyl-4'-(trifluoromethyl)-[1,1'-biphenyl]-2-amine. Small amounts of the C-N coupling product (*), aryl iodide, aryl homocoupling and aryl reduction product are observed.

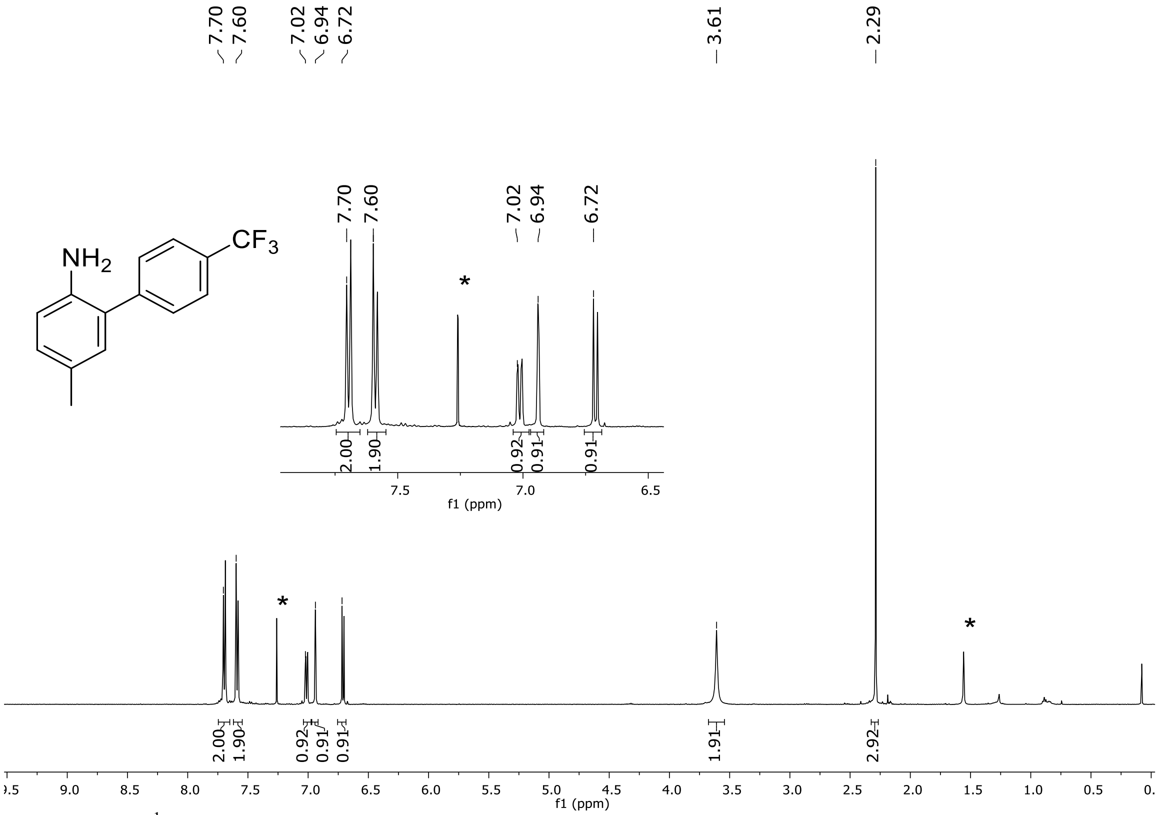


**Figure S42.** $^{1}H$ NMR (500.13 MHz, $CDCl_3$) of 5-methyl-4'-(trifluoromethyl)-[1,1'-biphenyl]-2-amine (**5c**) at 298 K. (*) Signals corresponding to the solvent (chloroform and $H_2O$).

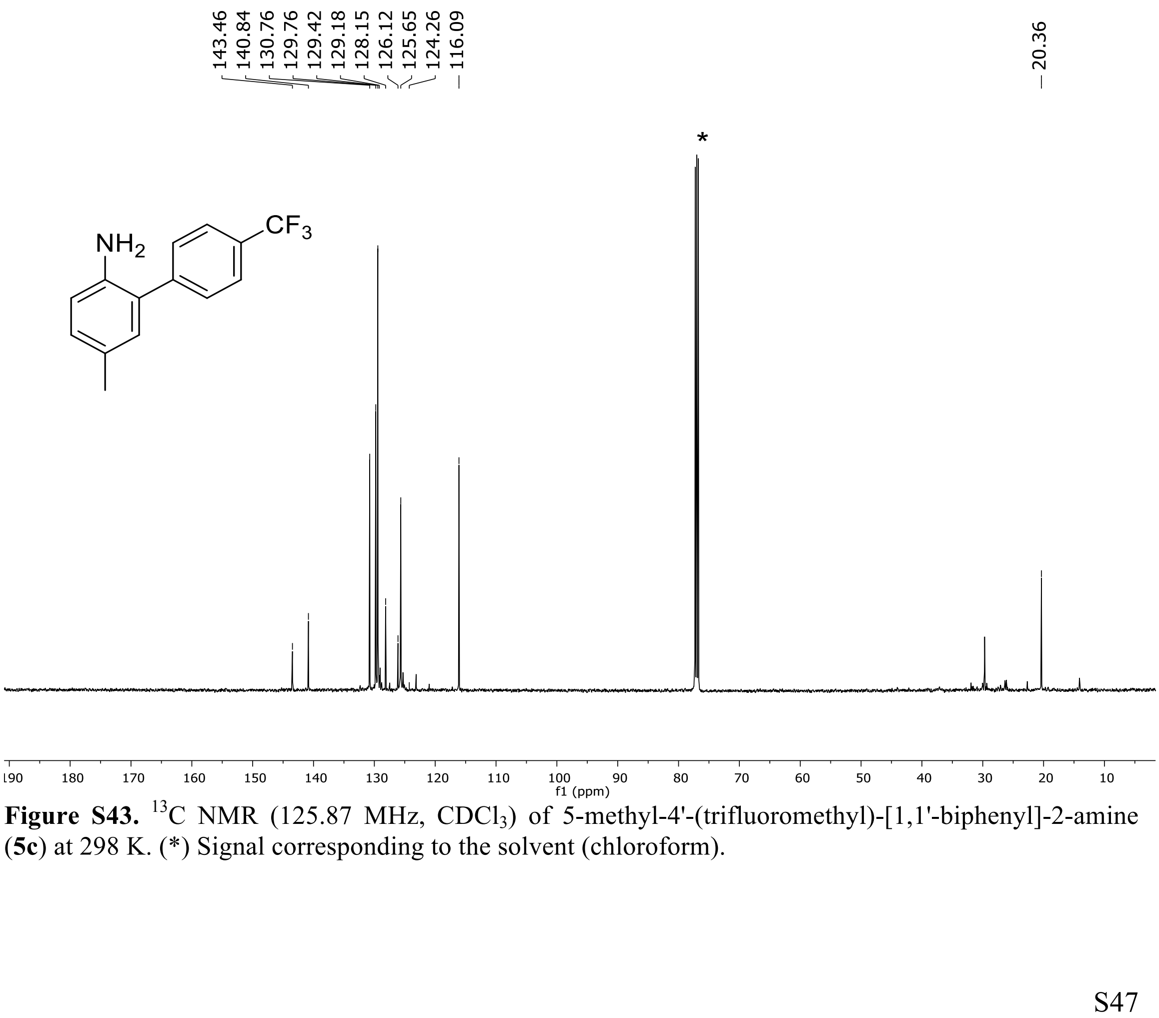


**Figure S43.** $^{13}C$ NMR (125.87 MHz, $CDCl_3$) of 5-methyl-4'-(trifluoromethyl)-[1,1'-biphenyl]-2-amine (**5c**) at 298 K. (*) Signal corresponding to the solvent (chloroform).

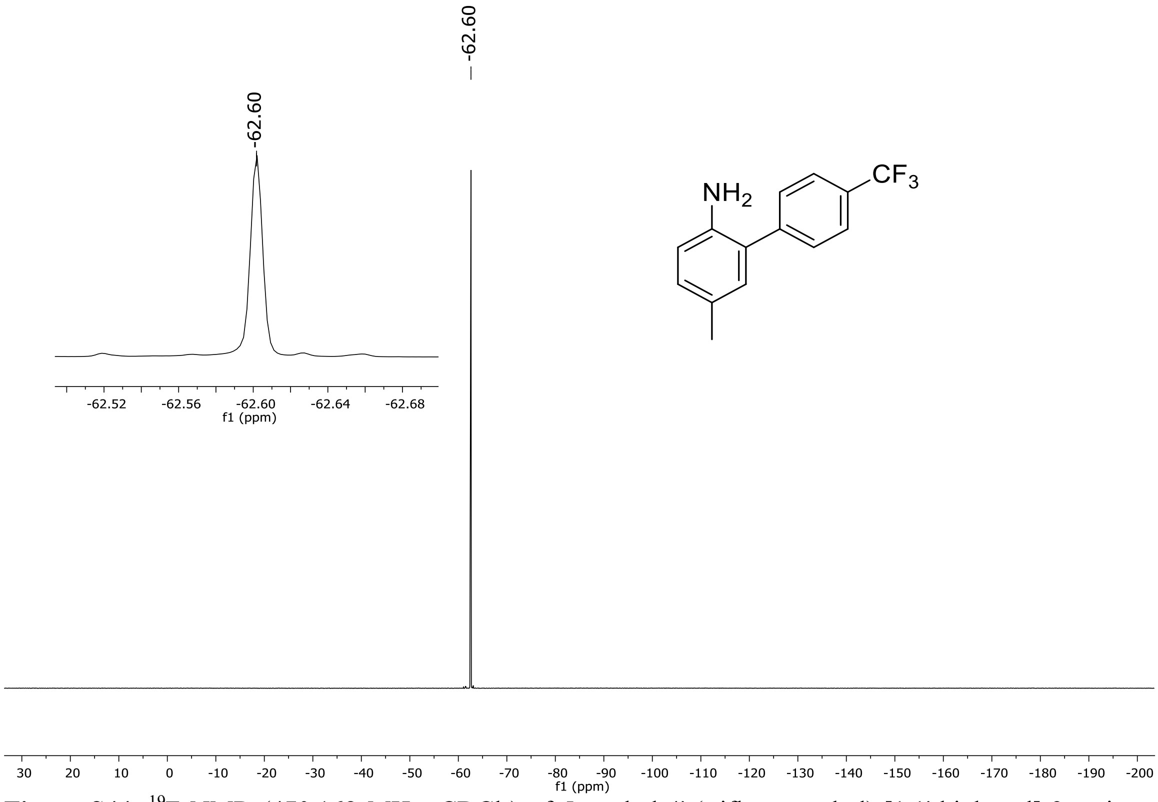


**Figure S44**. $^{19}$F NMR (470.168 MHz, $CDCl_3$) of 5-methyl-4'-(trifluoromethyl)-[1,1'-biphenyl]-2-amine (**5c**) at 298 K.

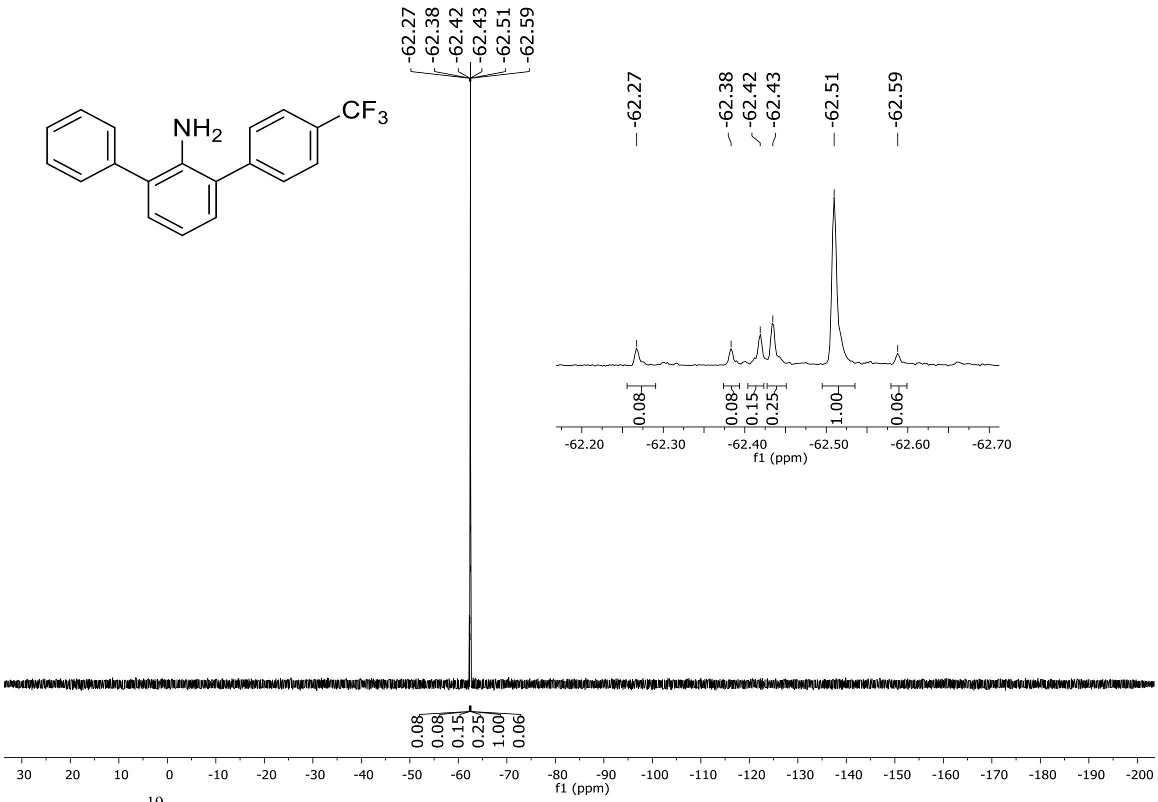


**Figure S45.** $^{19}$F NMR (470.168 MHz, DMA/$CDCl_3$) of an aliquot of the crude mixture of 3-phenyl-4'-(trifluoromethyl)-[1,1'-biphenyl]-2-amine. The aryl homocoupling product and other minor unidentified products are observed.

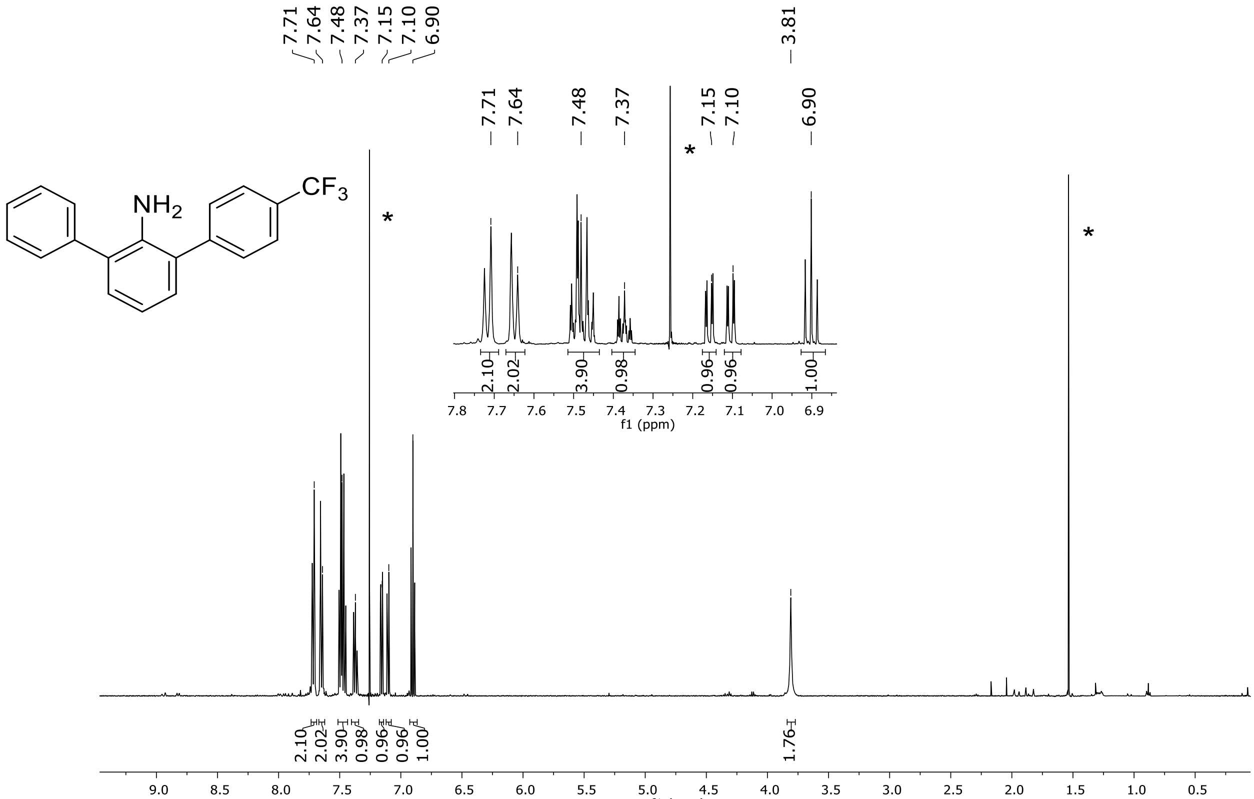

**Figure S46**. $^{1}$H NMR (500.13 MHz, $CDCl_3$) of 3-phenyl-4'-(trifluoromethyl)-[1,1'-biphenyl]-2-amine (**5d**) at 298 K.(*) Signals corresponding to the solvent (chloroform and $H_2O$).

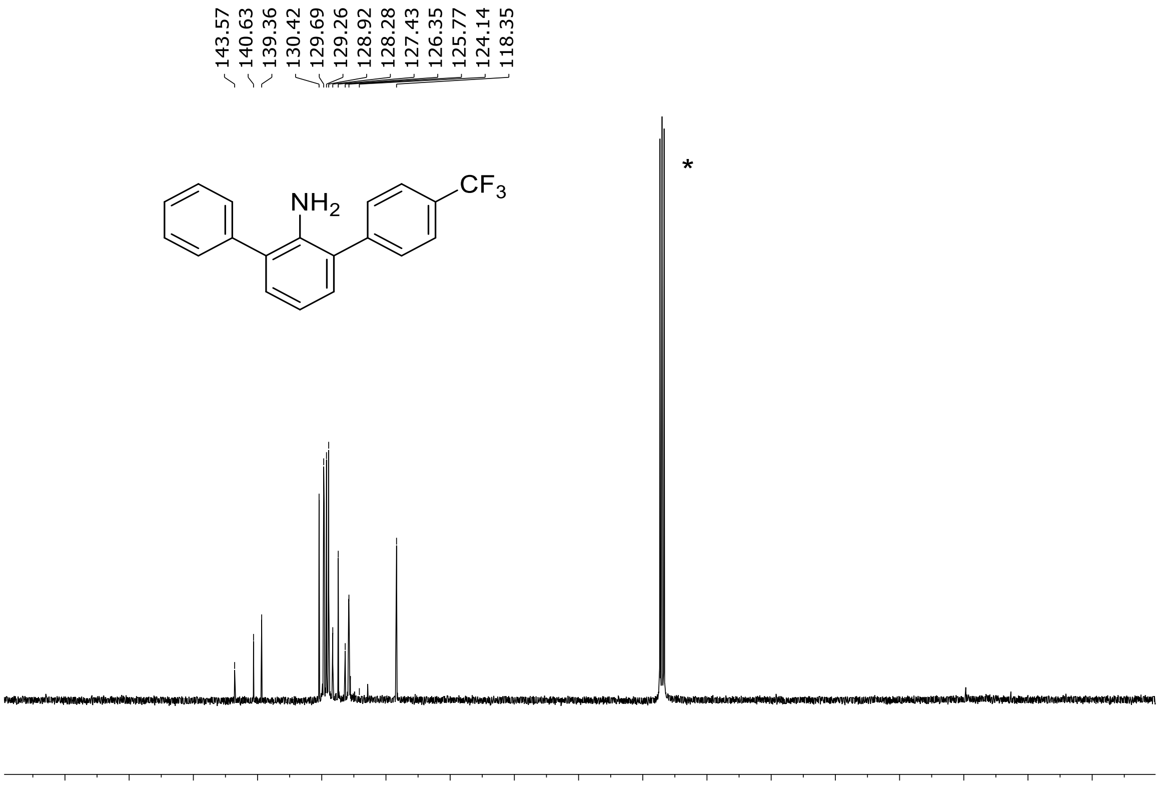

**Figure S47.** $^{13}$C NMR (125.87 MHz, $CDCl_3$) of 3-phenyl-4'-(trifluoromethyl)-[1,1'-biphenyl]-2-amine (**5d**) at 298 K. (*) Signal corresponding to the solvent (chloroform).

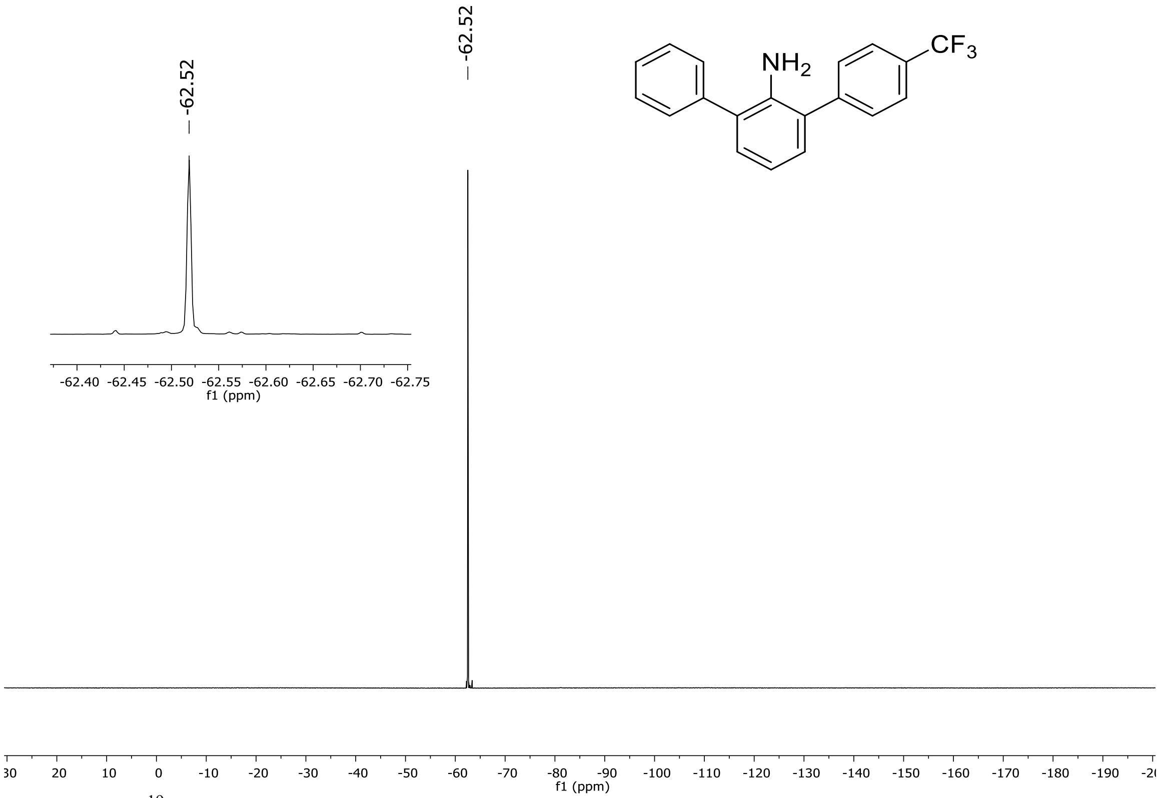


**Figure S48.** $^{19}$F NMR (470.168 MHz, $CDCl_3$) of 3-phenyl-4'-(trifluoromethyl)-[1,1'-biphenyl]-2-amine (**5d**) at 298 K.

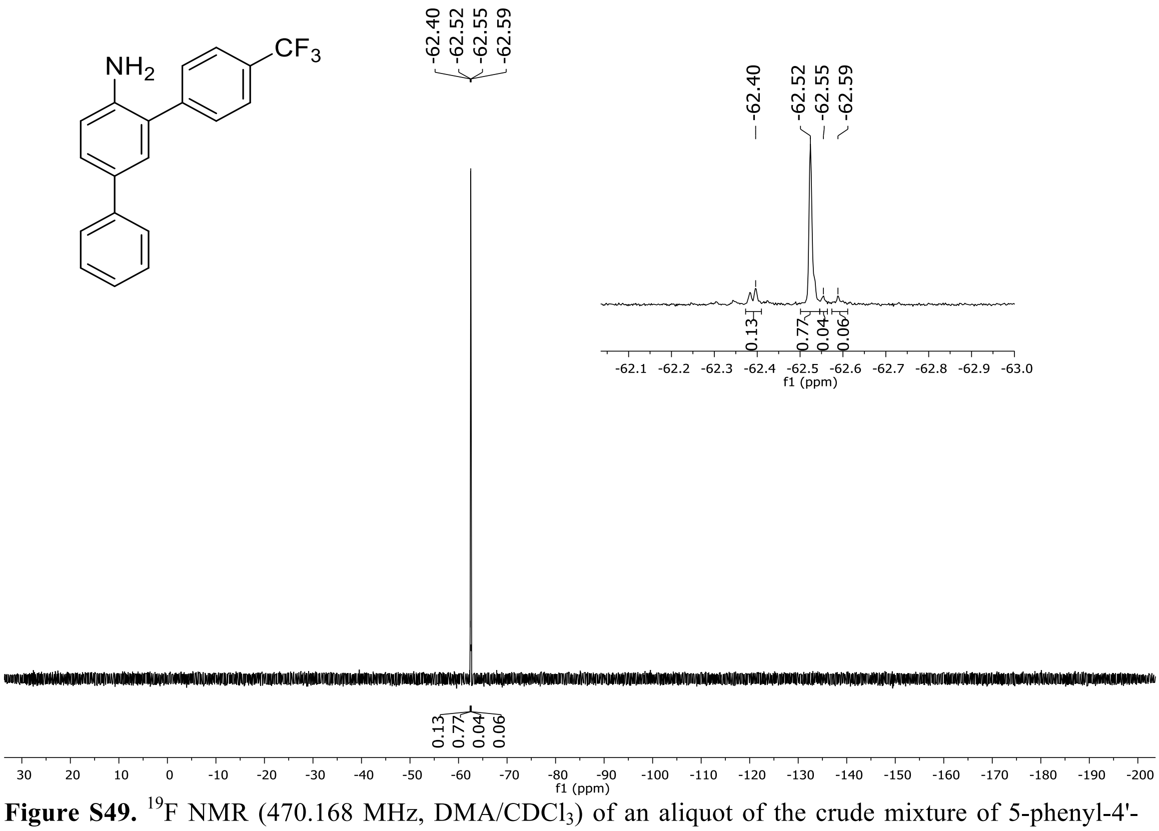


**Figure S49.** $^{19}$F NMR (470.168 MHz, DMA/$CDCl_3$) of an aliquot of the crude mixture of 5-phenyl-4'-(trifluoromethyl)-[1,1'-biphenyl]-2-amine. Aryl homocoupling product and other minor products are observed.

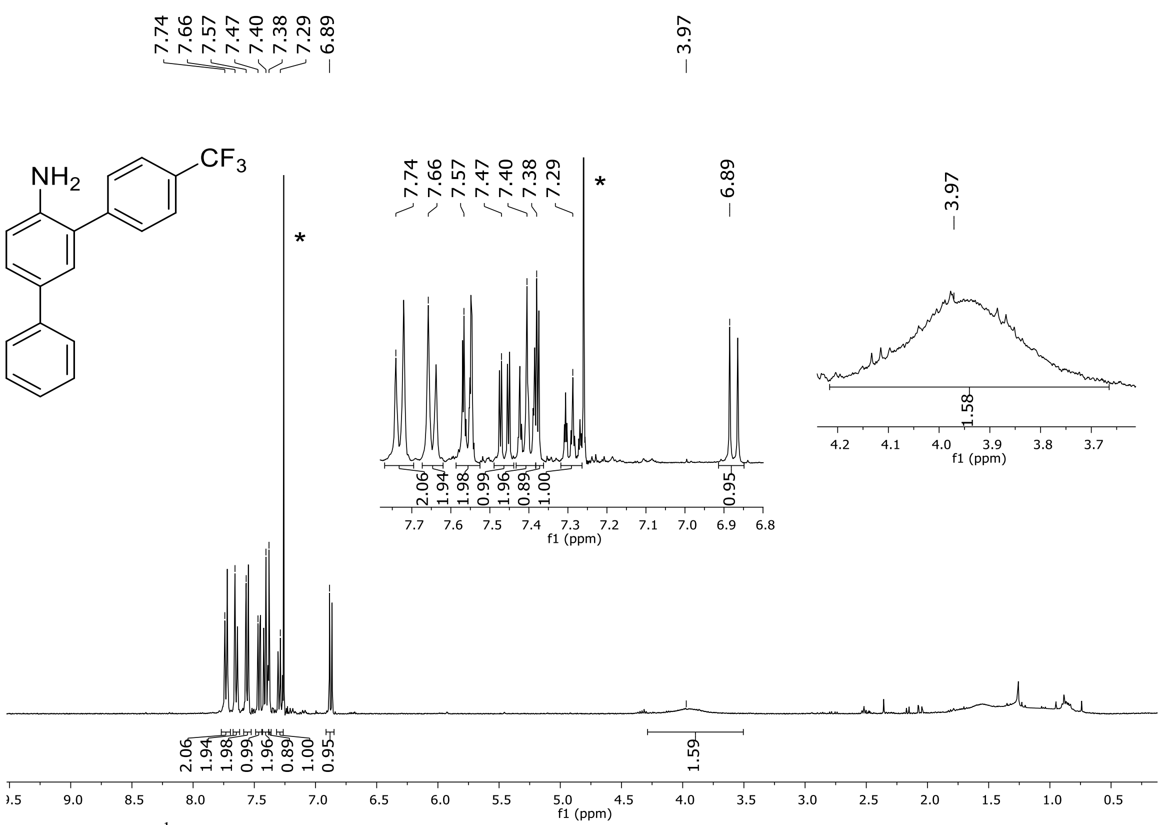


**Figure S50.** $^{1}H$ NMR (500.13 MHz, $CDCl_3$) of 5-phenyl-4'-(trifluoromethyl)-[1,1'-biphenyl]-2-amine (**5e**) at 298 K. (*) Signal corresponding to the solvent (chloroform).

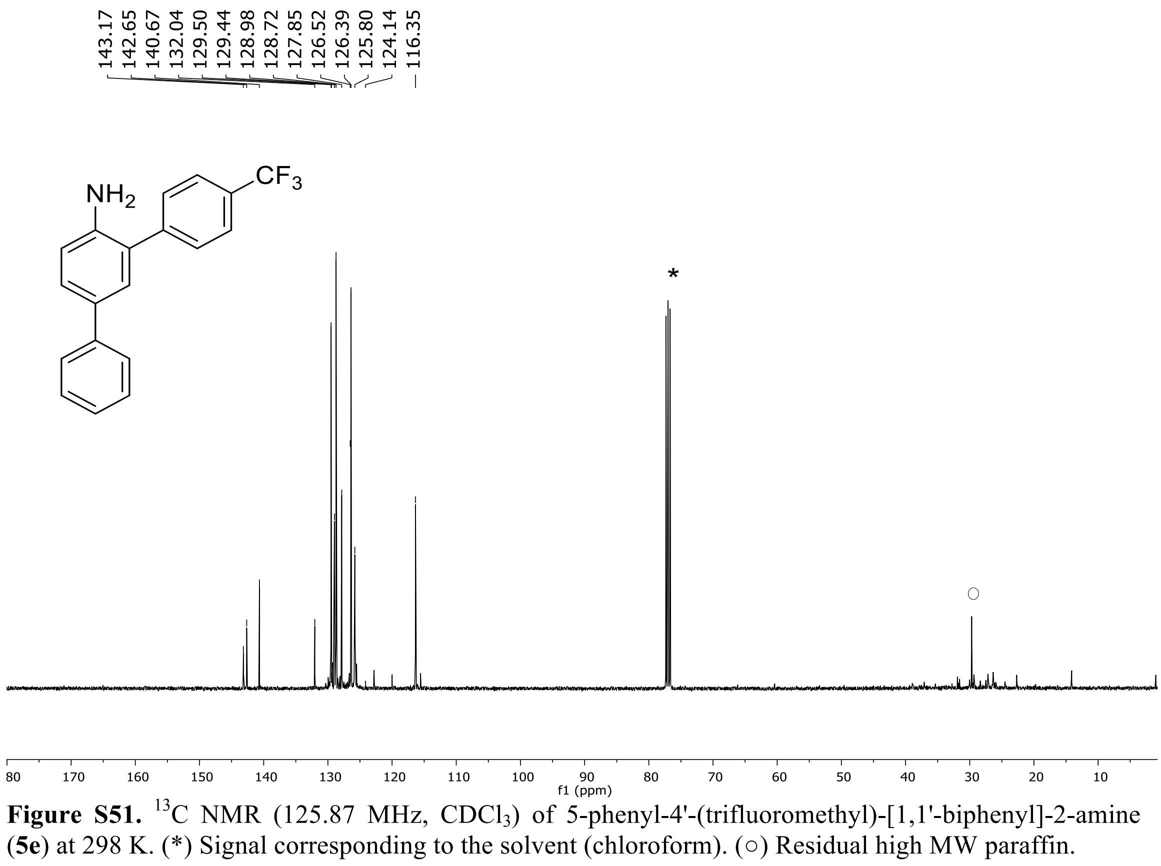


**Figure S51.** $^{13}C$ NMR (125.87 MHz, $CDCl_3$) of 5-phenyl-4'-(trifluoromethyl)-[1,1'-biphenyl]-2-amine (**5e**) at 298 K. (*) Signal corresponding to the solvent (chloroform). (○) Residual high MW paraffin.

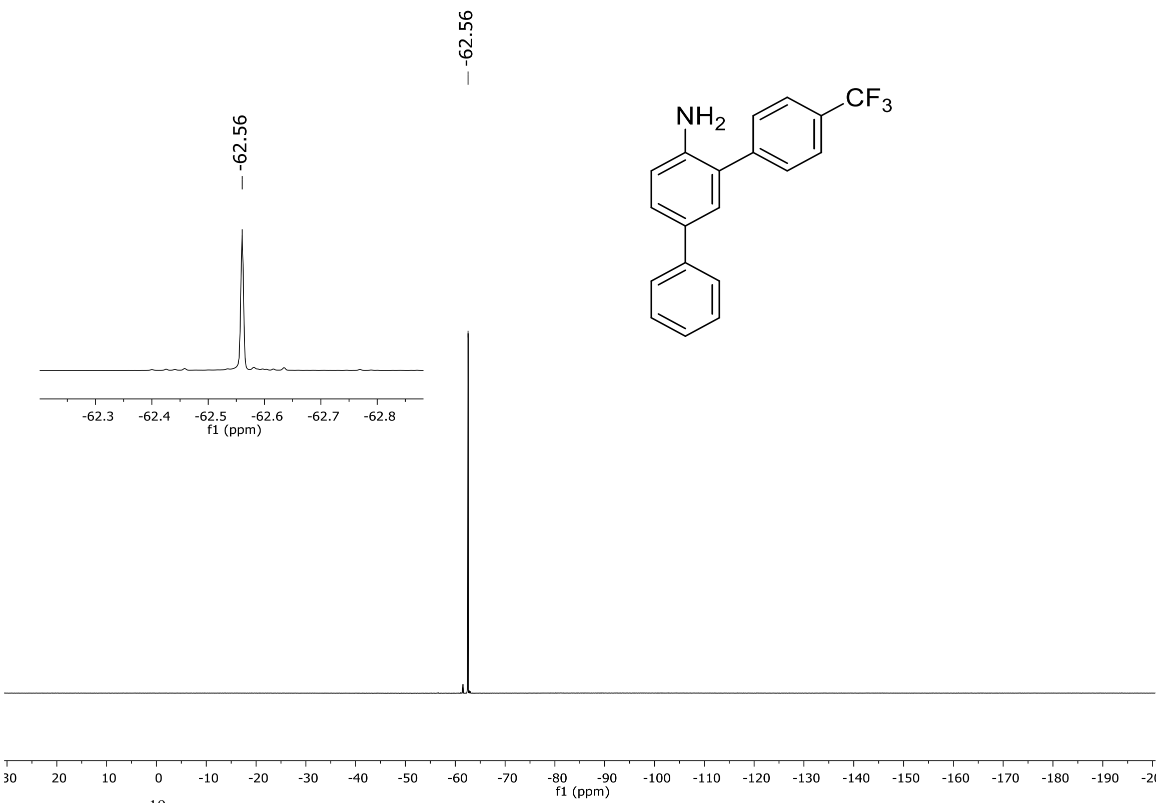


**Figure S52.** $^{19}$F NMR (470.168 MHz, $CDCl_3$) of 5-phenyl-4'-(trifluoromethyl)-[1,1'-biphenyl]-2-amine (**5e**) at 298 K.

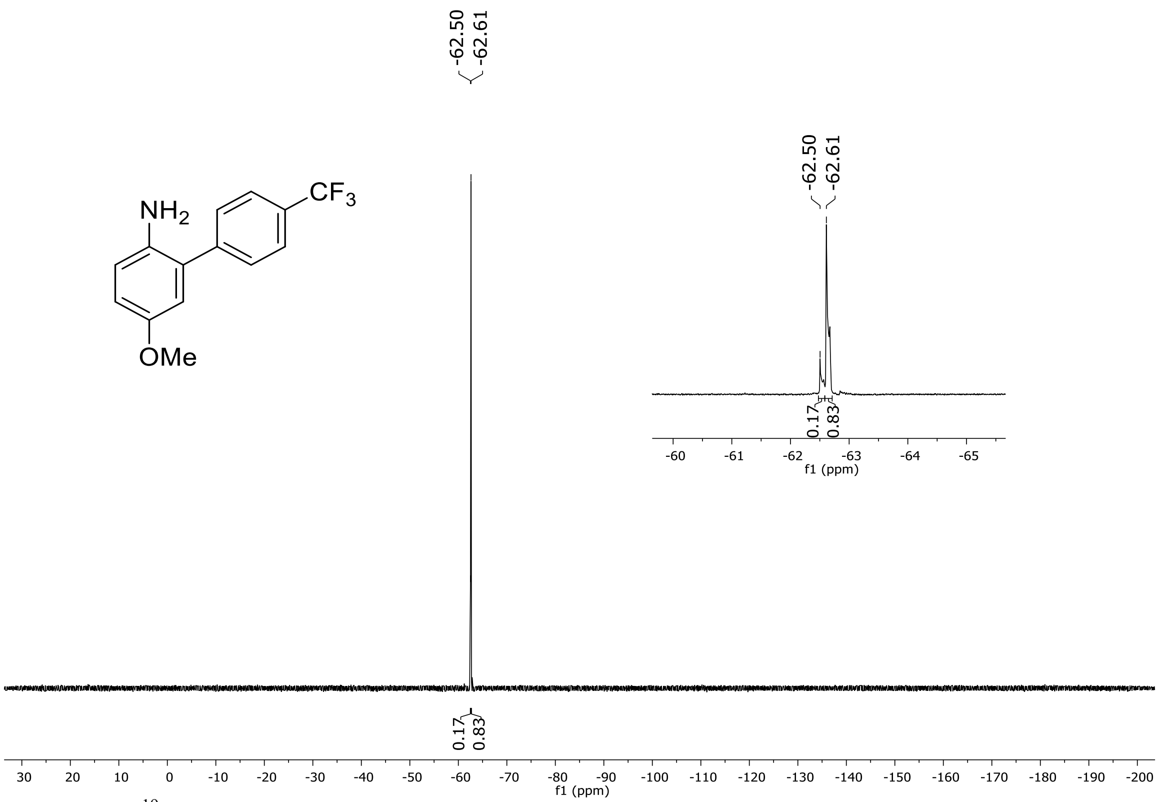


**Figure S53.** $^{19}$F NMR (470.168 MHz, DMA/$CDCl_3$) of an aliquot of the crude mixture of 5-methoxy-4'-(trifluoromethyl)-[1,1'-biphenyl]-2-amine. The aryl homocoupling product is observed.

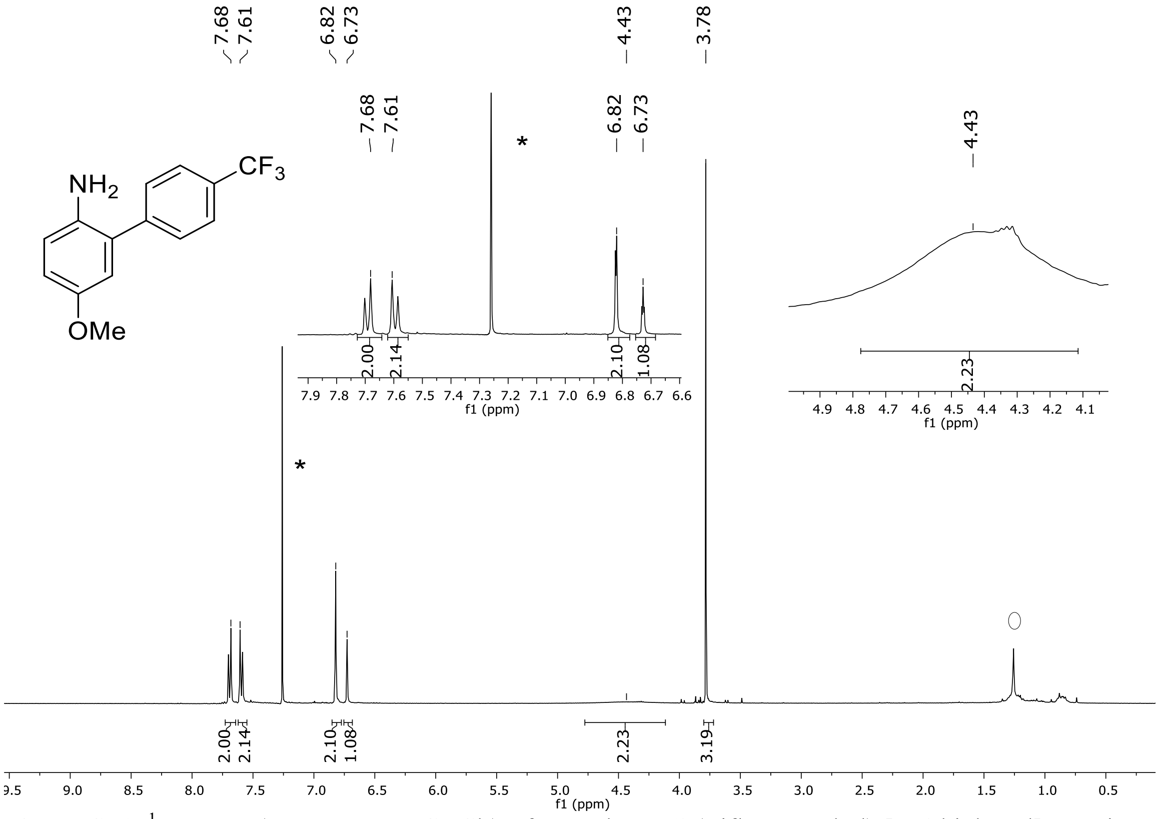

**Figure S54.** $^{1}H$ NMR (500.13 MHz, $CDCl_3$) of 5-methoxy-4'-(trifluoromethyl)-[1,1'-biphenyl]-2-amine (**5f**) at 298 K. (*) Signal corresponding to the solvent (chloroform). (○) Residual high MW paraffin.

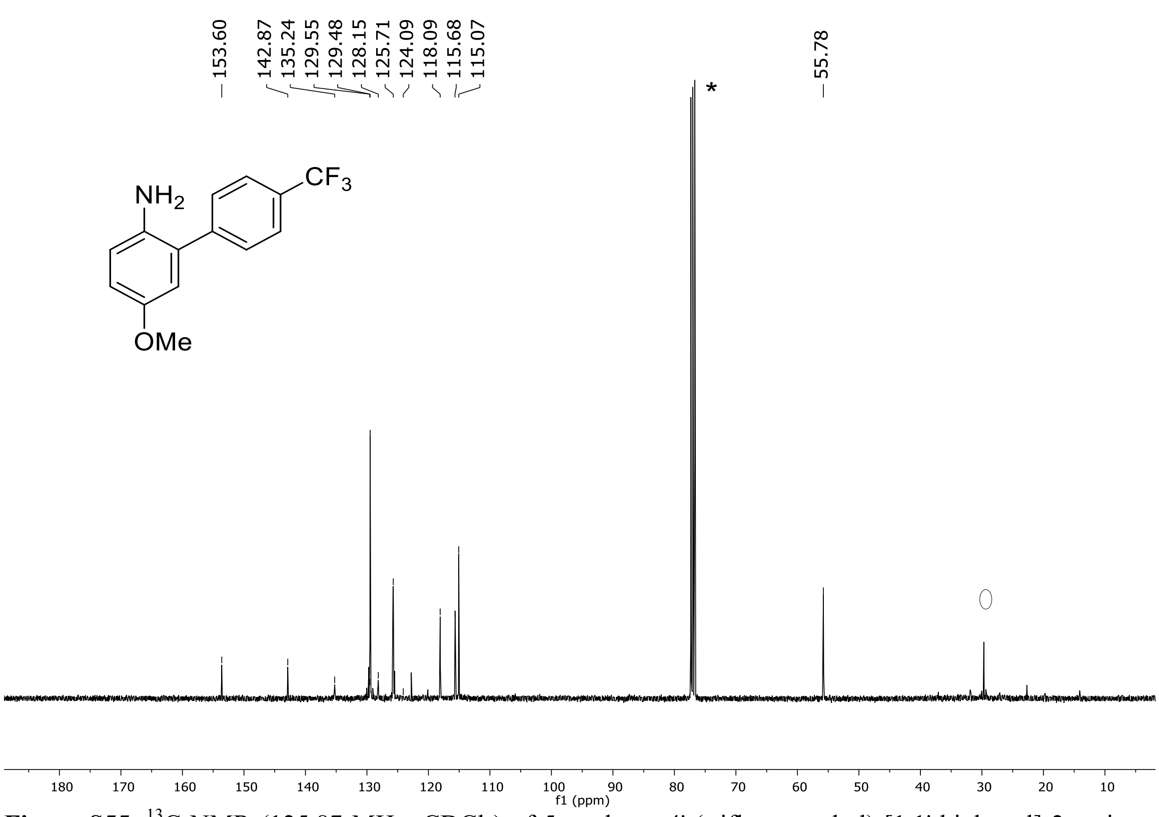

**Figure S55.** $^{13}C$ NMR (125.87 MHz, $CDCl_3$) of 5-methoxy-4'-(trifluoromethyl)-[1,1'-biphenyl]-2-amine (**5f**) at 298 K. (*) Signal corresponding to the solvent (chloroform). (○)Residual high MW paraffin.

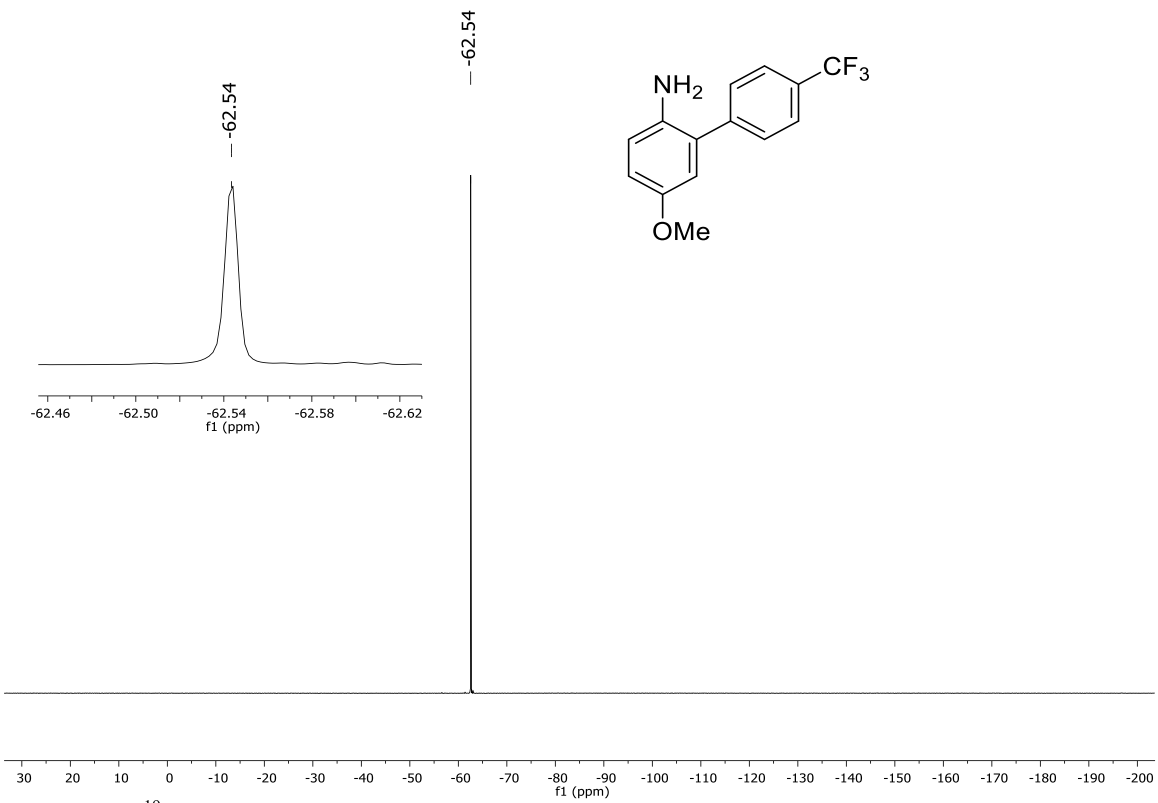

**Figure S56.** $^{19}$F NMR (470.168 MHz, $CDCl_3$) of 5-methoxy-4'-(trifluoromethyl)-[1,1'-biphenyl]-2-amine (**5f**) at 298 K.

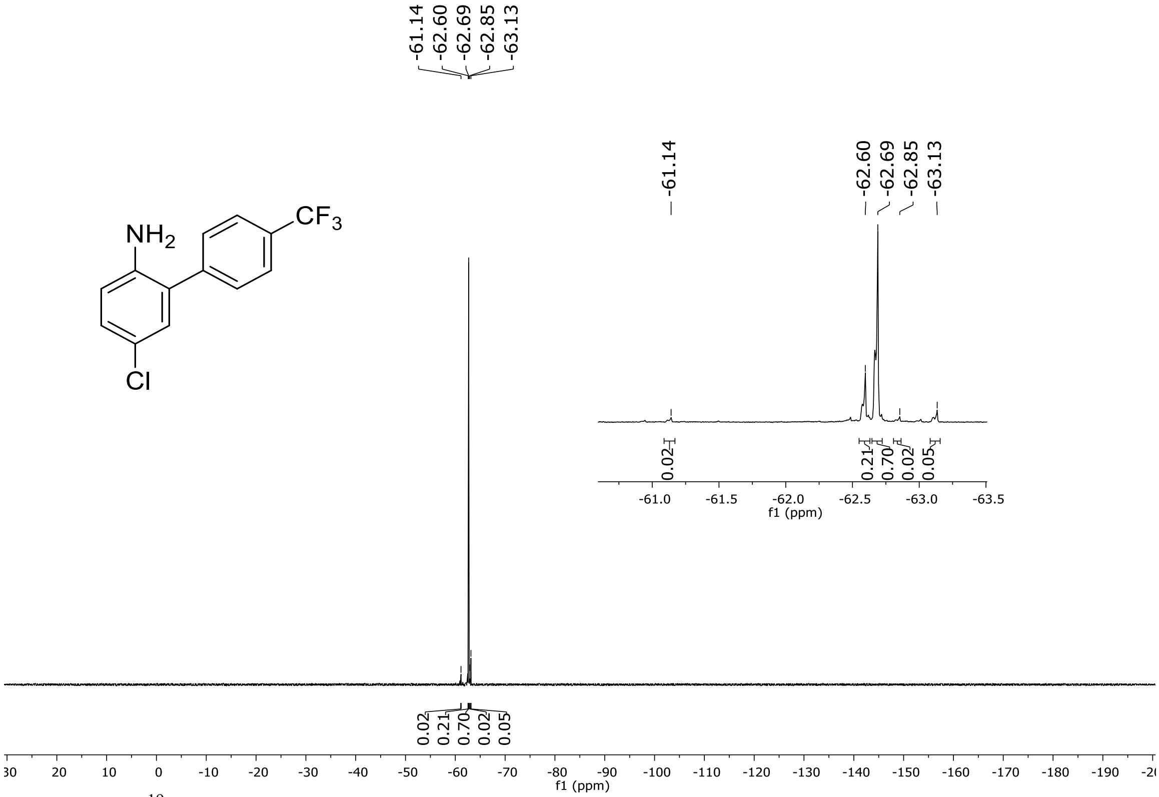

**Figure S57.** $^{19}$F NMR (470.168 MHz, DMA/$CDCl_3$) of an aliquot of the crude mixture of 5-chloro-4'-(trifluoromethyl)-[1,1'-biphenyl]-2-amine. The aryl homocoupling product and small amounts of other minor products are observed.

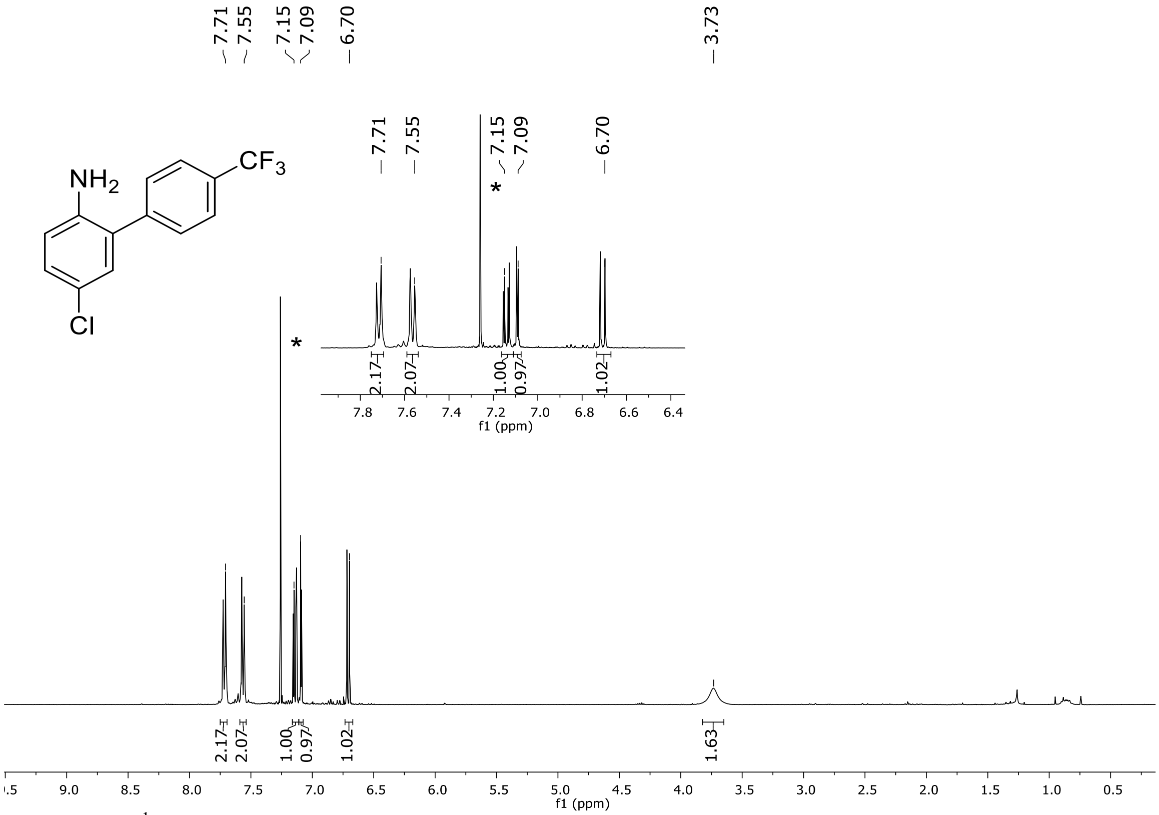


**Figure S58.** $^{1}H$ NMR (500.13 MHz, $CDCl_3$) of 5-chloro-4'-(trifluoromethyl)-[1,1'-biphenyl]-2-amine (**5g**) at 298 K. (*) Signal corresponding to the solvent (chloroform).

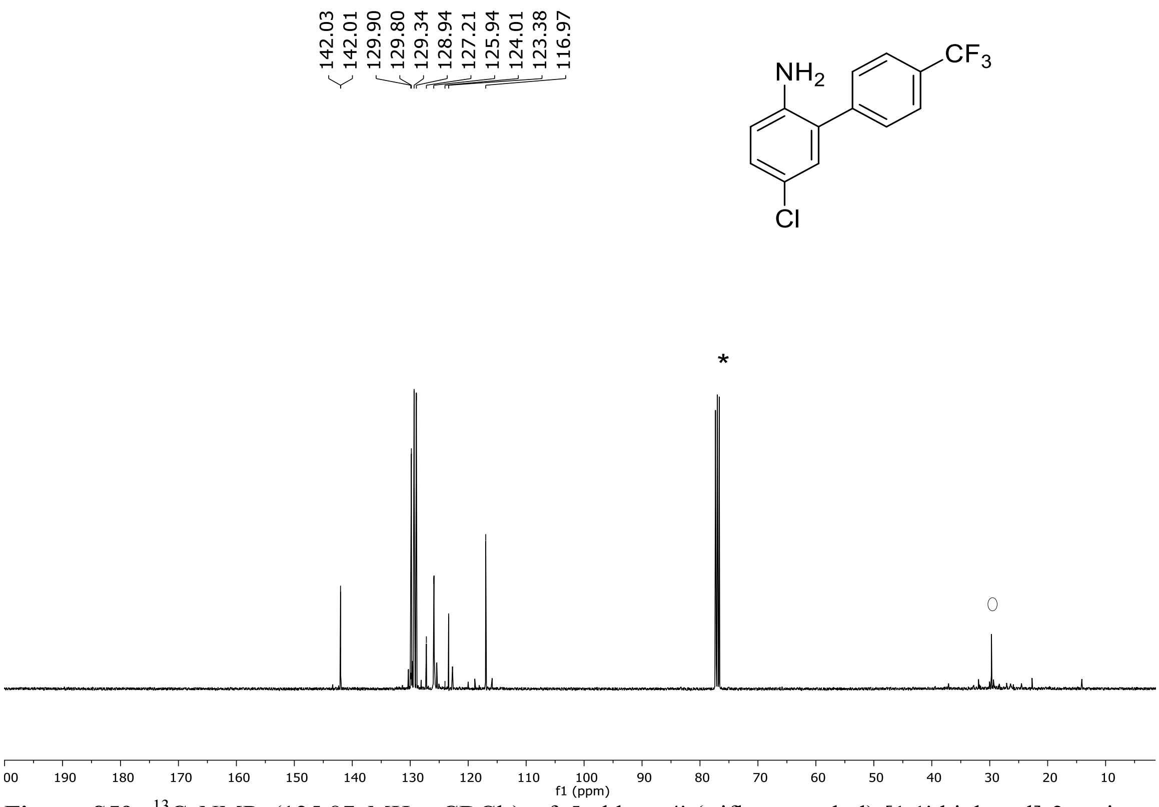


**Figure S59.** $^{13}C$ NMR (125.87 MHz, $CDCl_3$) of 5-chloro-4'-(trifluoromethyl)-[1,1'-biphenyl]-2-amine (**5g**) at 298 K. (*) Signal corresponding to the solvent (chloroform). (○) Residual high MW paraffin.

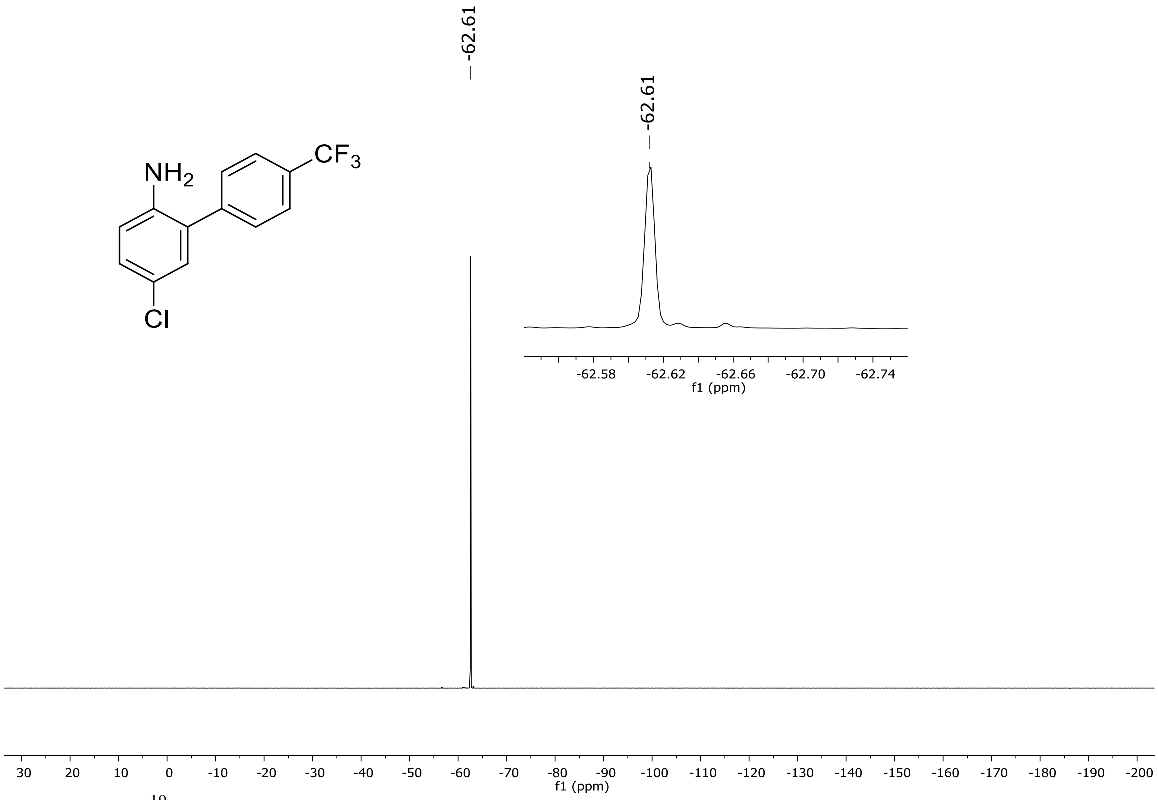


**Figure S60.** $^{19}$F NMR (470.168 MHz, $CDCl_3$) of 5-chloro-4'-(trifluoromethyl)-[1,1'-biphenyl]-2-amine (**5g**) at 298 K.

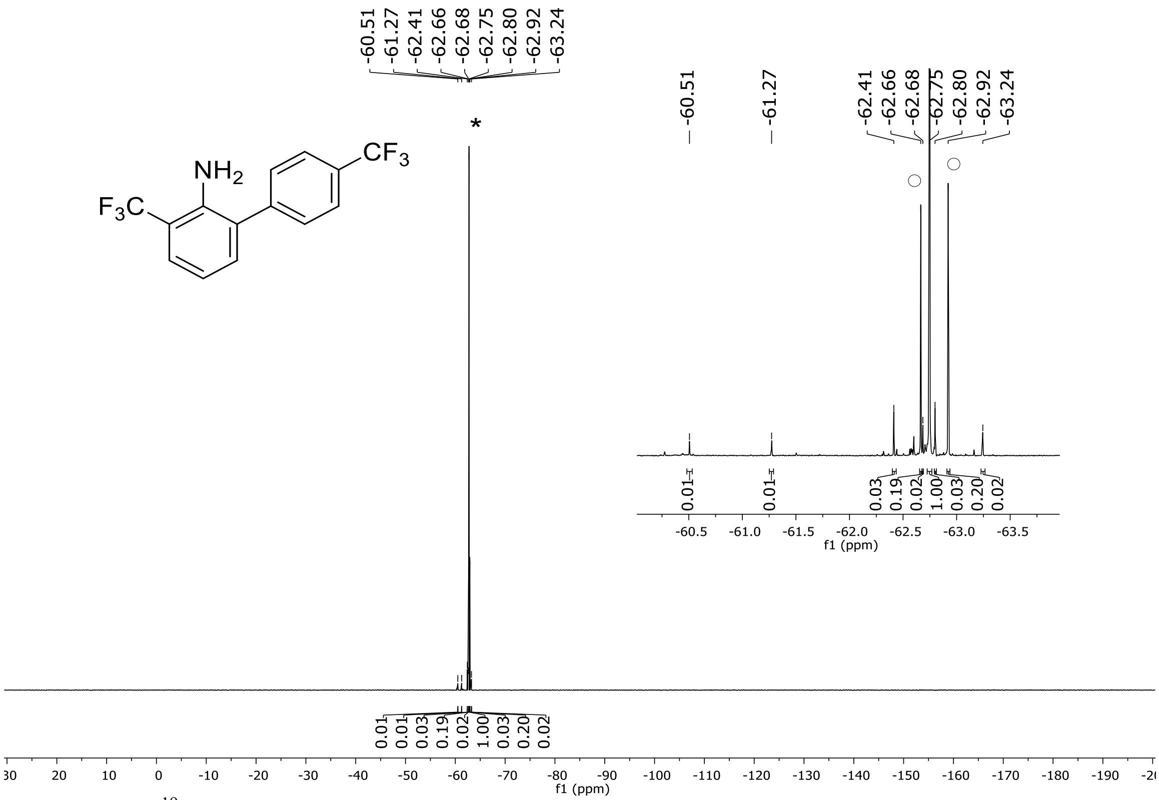


**Figure S61.** $^{19}$F NMR (470.168 MHz, DMA/$CDCl_3$) of an aliquot of the crude mixture of 3-trifluoromethyl-4'-(trifluoromethyl)-[1,1'-biphenyl]-2-amine (○). (*) Signal corresponding to the reactant arene in excess. Minor unidentified products were detected.

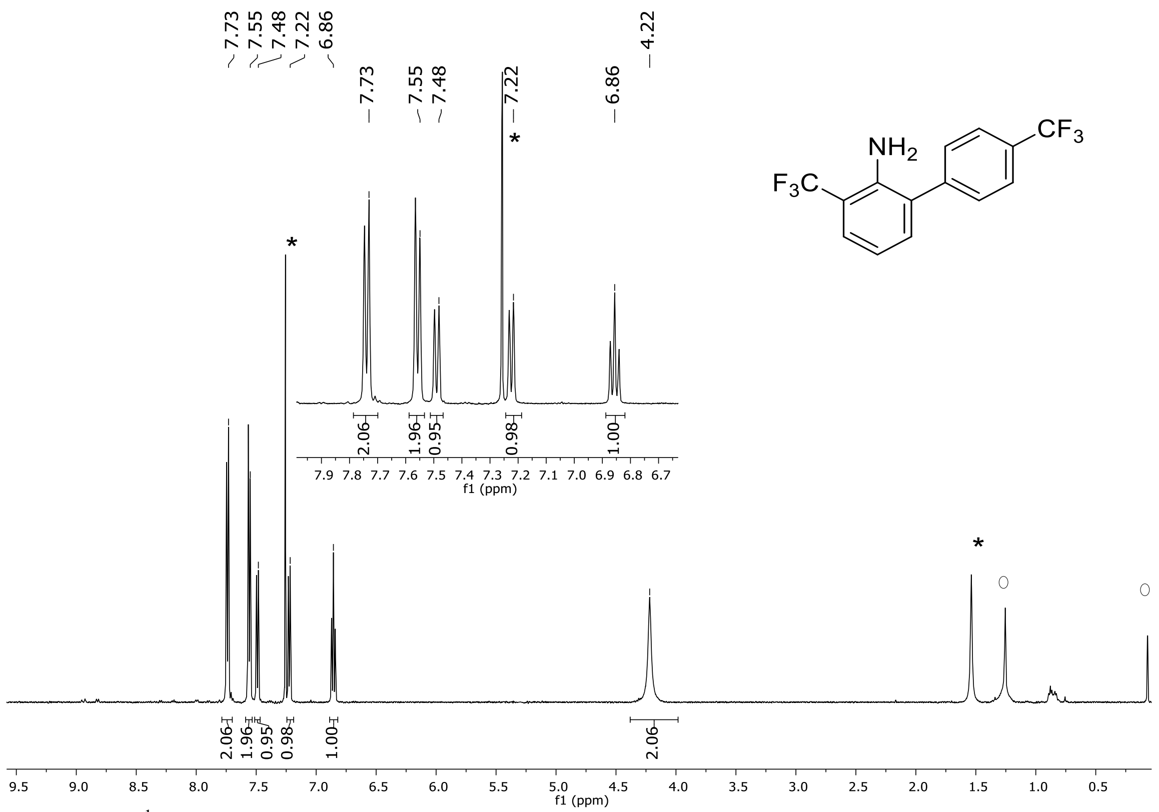


**Figure S62.** $^{1}$H NMR (500.13 MHz, $CDCl_3$) of 3-trifluoromethyl-4'-(trifluoromethyl)-[1,1'-biphenyl]-2-amine (**5h**) at 298 K. (*) Signal corresponding to the solvent (chloroform and $H_2O$). (○) Residual high MW paraffin.

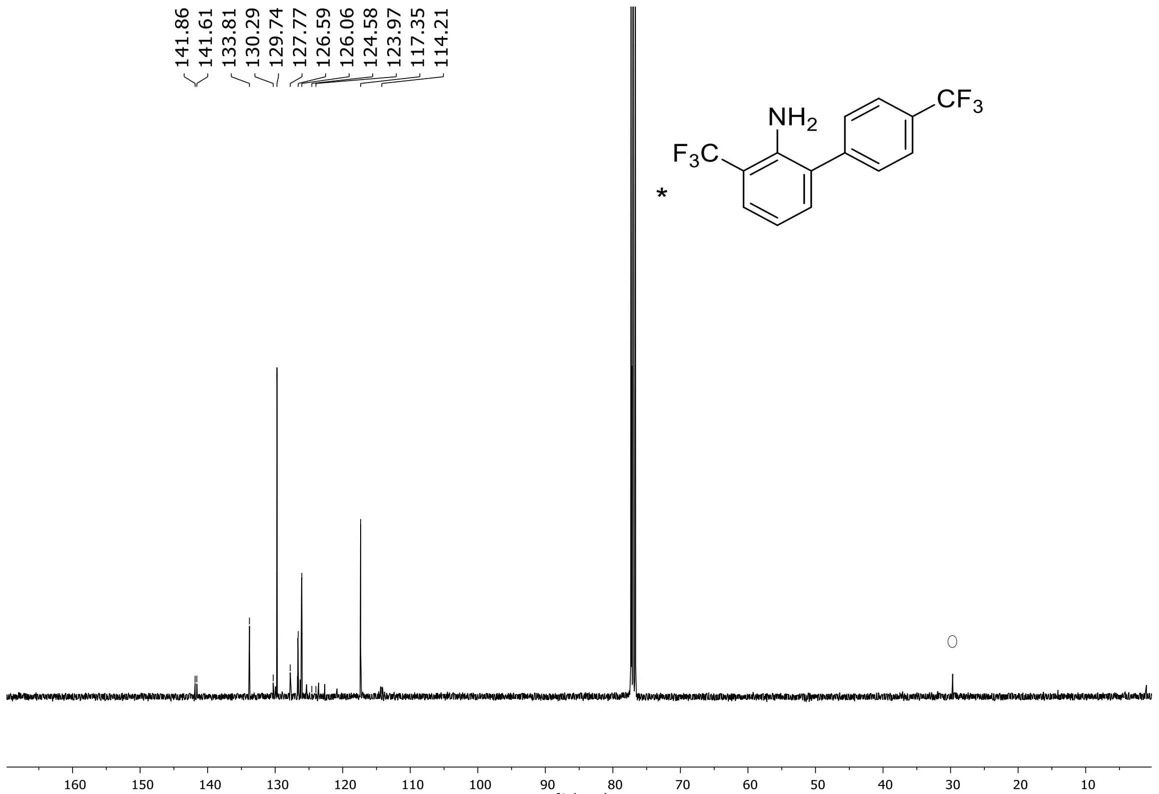


**Figure S63.** $^{13}$C NMR (125.87 MHz, $CDCl_3$) of 3-trifluoromethyl-4'-(trifluoromethyl)-[1,1'-biphenyl]-2-amine (**5h**) at 298 K. (*) Signal corresponding to the solvent (chloroform). (○) Residual high MW paraffin.

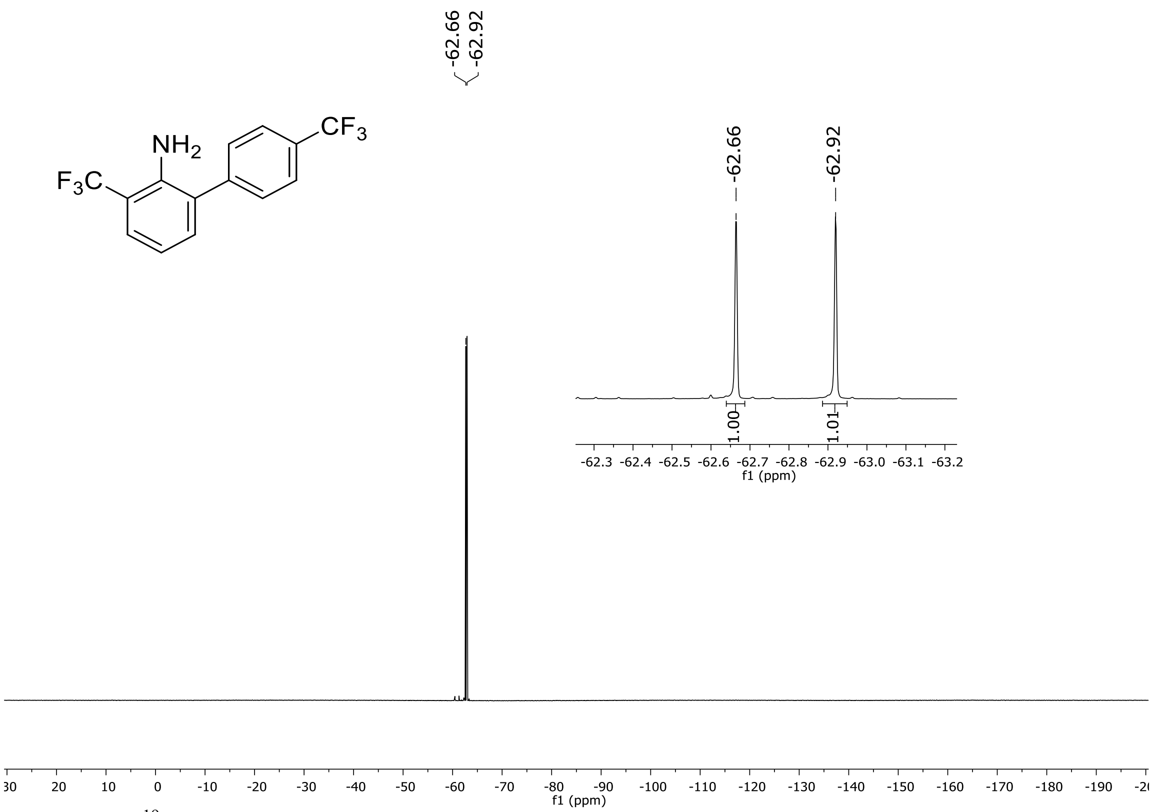


**Figure S64.** $^{19}F$ NMR (470.168 MHz, $CDCl_3$) of 3-trifluoromethyl-4'-(trifluoromethyl)-[1,1'-biphenyl]-2-amine (**5h**) at 298 K.

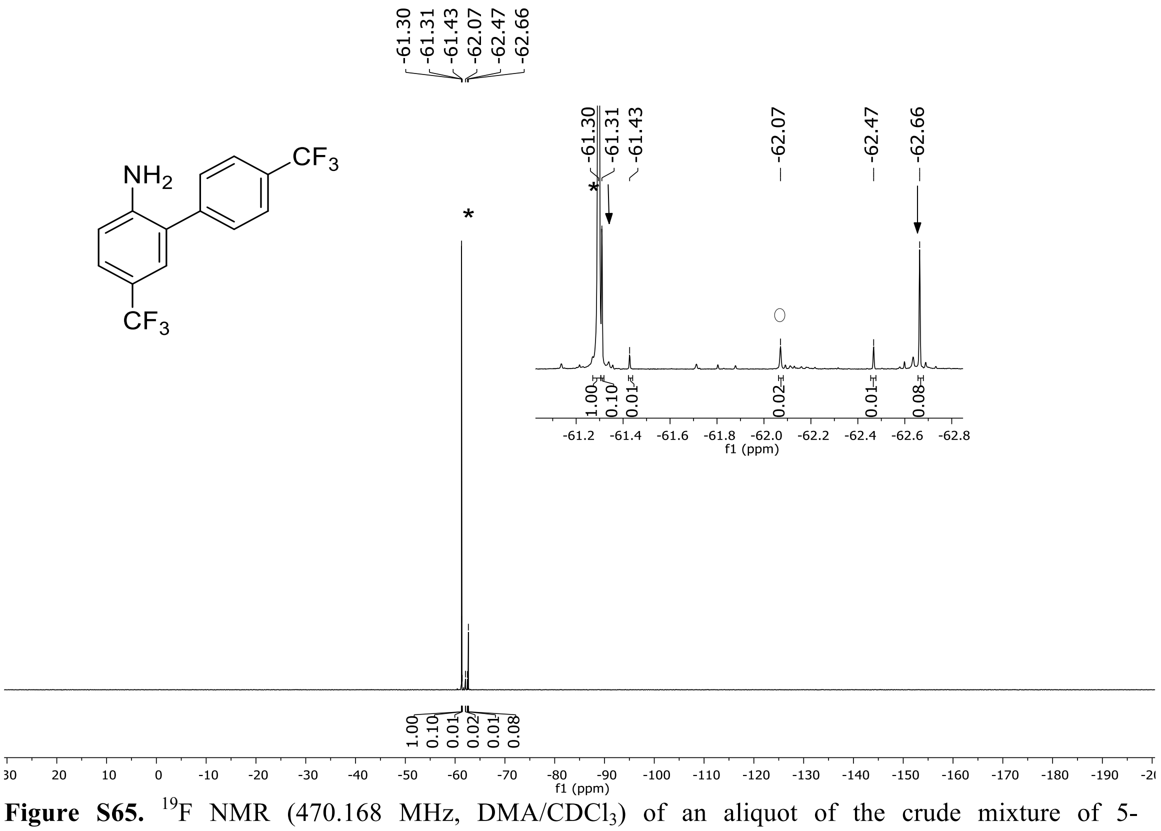


**Figure S65.** $^{19}F$ NMR (470.168 MHz, DMA/$CDCl_3$) of an aliquot of the crude mixture of 5-trifluoromethyl-4'-(trifluoromethyl)-[1,1'-biphenyl]-2-amine (↓). (*) Signal corresponding to the reactant arene in excess. (○) Signal corresponding to the C-N coupling product. A minor unidentified compound was also detected

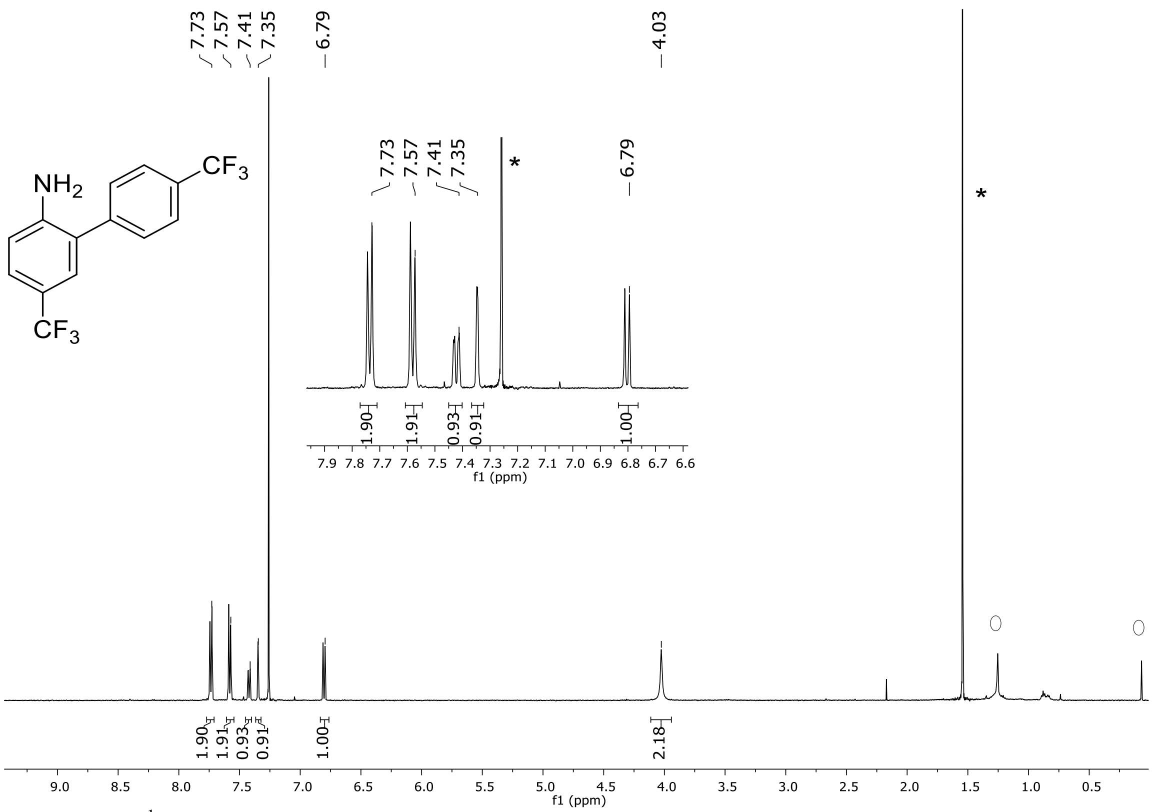


**Figure S66.** $^{1}H$ NMR (500.13 MHz, $CDCl_3$) of 5-trifluoromethyl-4'-(trifluoromethyl)-[1,1'-biphenyl]-2-amine (**5i**) at 298 K. (*) Signal corresponding to the solvent (chloroform an $H_2O$). (○) Residual high MW paraffin.

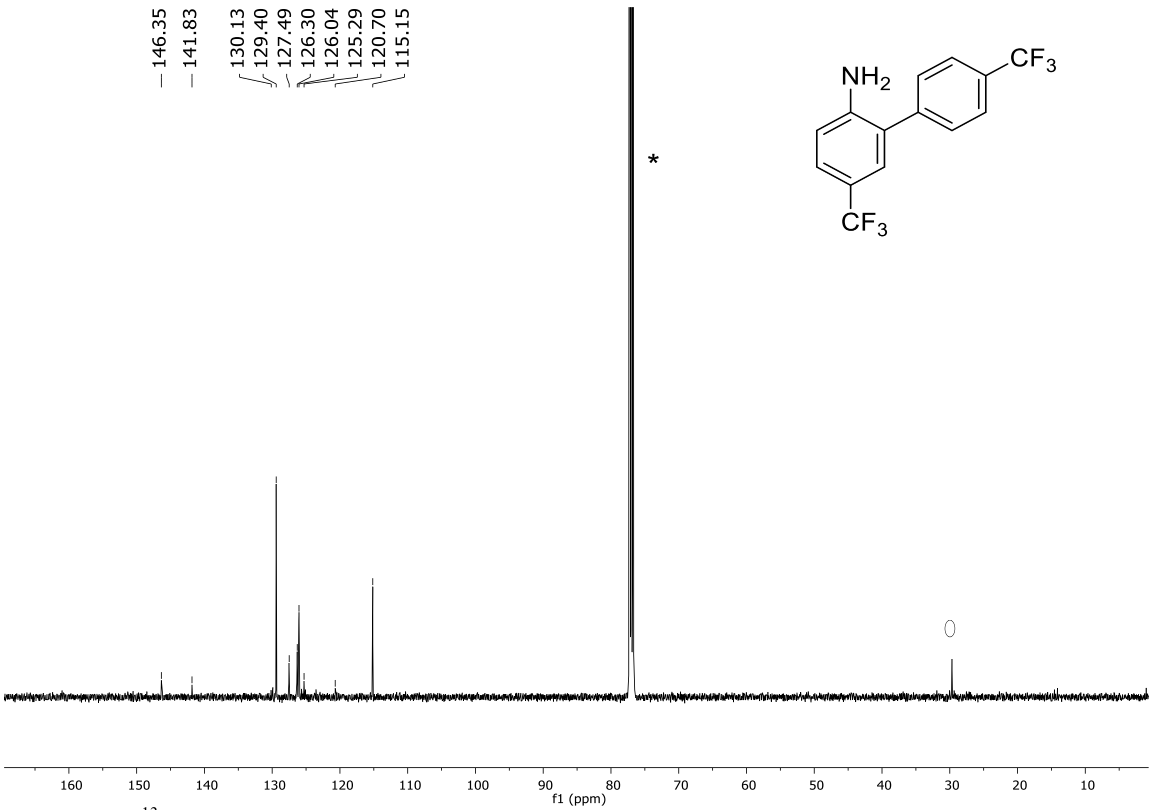


**Figure S67.** $^{13}C$ NMR (125.87 MHz, $CDCl_3$) of 5-trifluoromethyl-4'-(trifluoromethyl)-[1,1'-biphenyl]-2-amine (**5i**) at 298 K. (*) Signal corresponding to the solvent (chloroform). (○) Residual high MW paraffin.

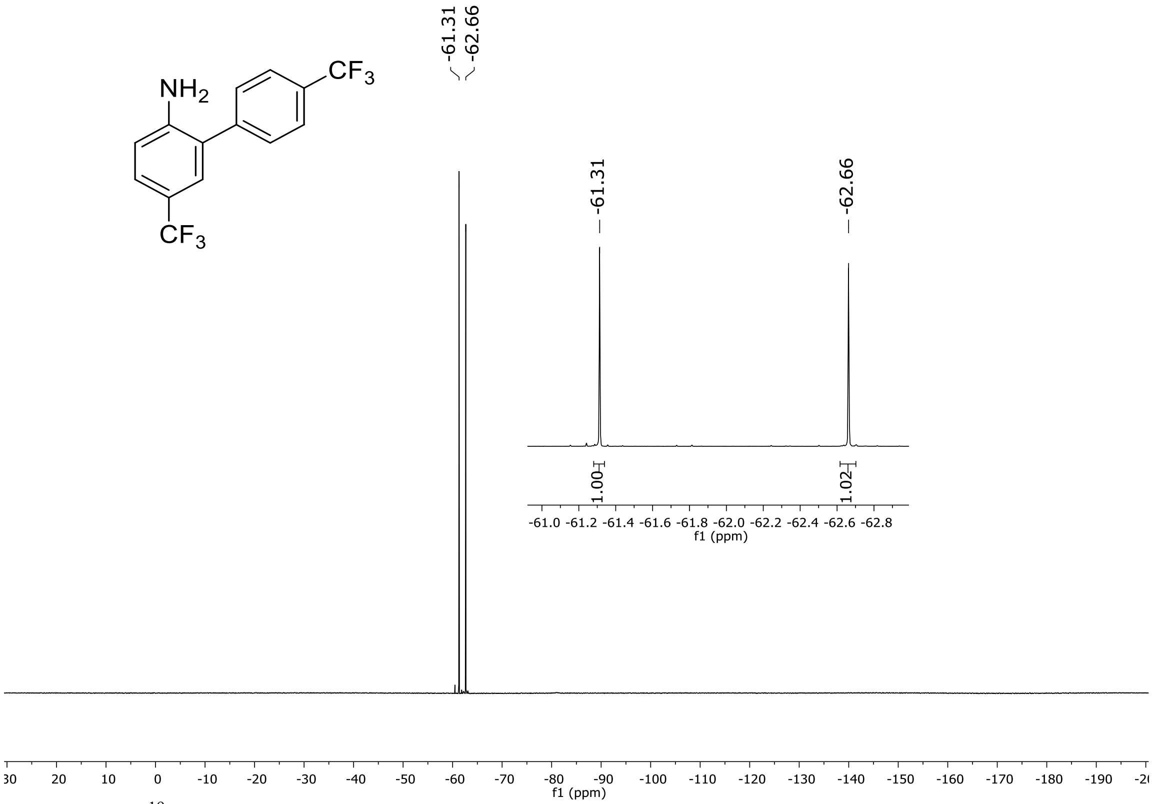


**Figure S68.** $^{19}$F NMR (470.168 MHz, $CDCl_3$) of 5-trifluoromethyl-4'-(trifluoromethyl)-[1,1'-biphenyl]-2-amine (**5i**) at 298 K.

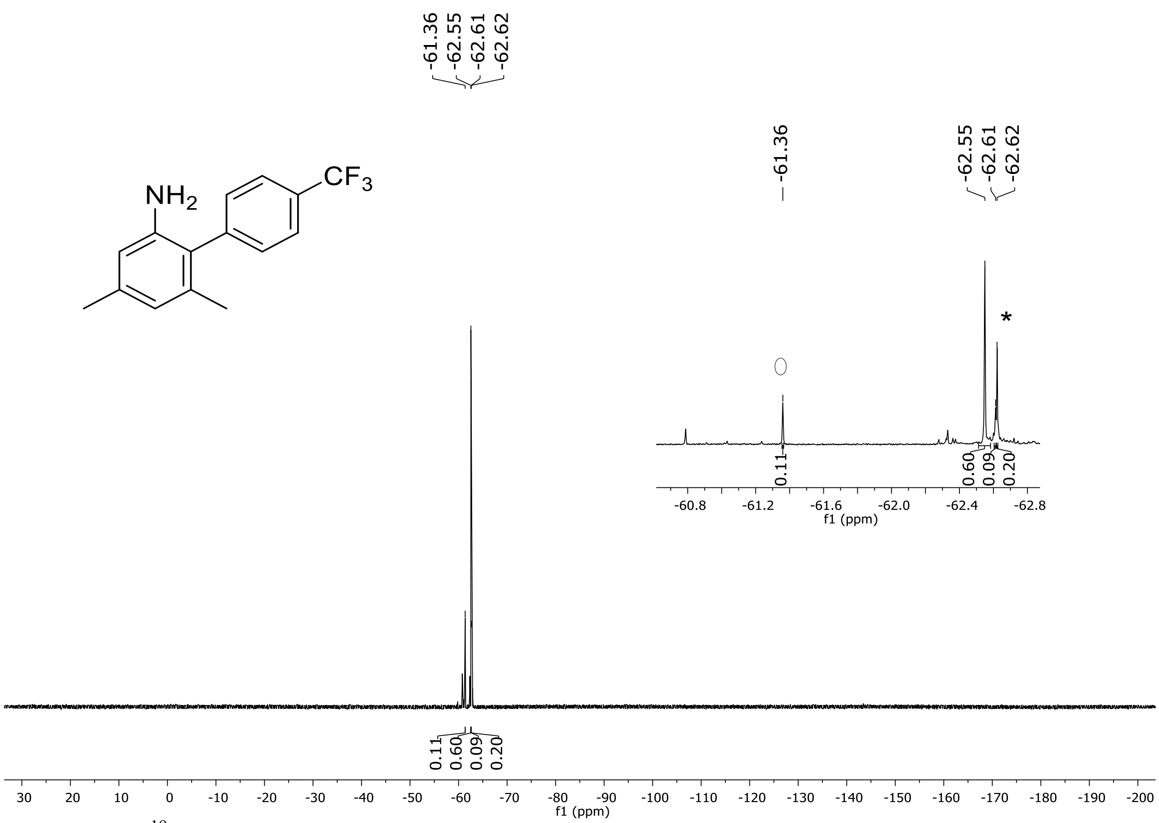


**Figure S69.** $^{19}$F NMR (470.168 MHz, $CDCl_3$) of an aliquot of the crude mixture of 4,6-dimethyl-4'-(trifluoromethyl)[1,1'-biphenyl]-2-amine. (*) Signals corresponding to the aryl homocoupling, (○) C-N coupling product. A minor unidentified compound was also observed.

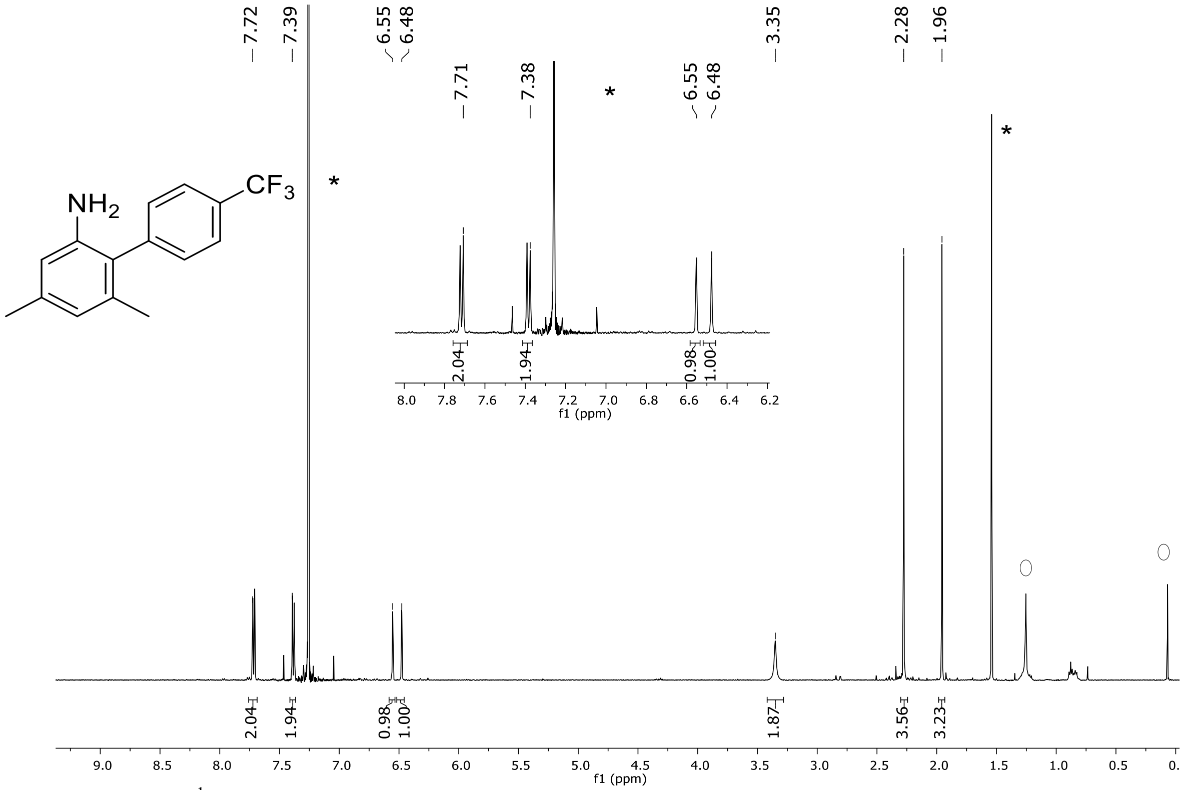


**Figure S70.** $^{1}H$ NMR (500.13 MHz, $CDCl_3$) of 4,6-dimethyl-4'-(trifluoromethyl)[1,1'-biphenyl]-2-amine (**5j**) at 298 K. (*) Signal corresponding to the solvent (chloroform and $H_2O$). (○) Residual high MW paraffin.

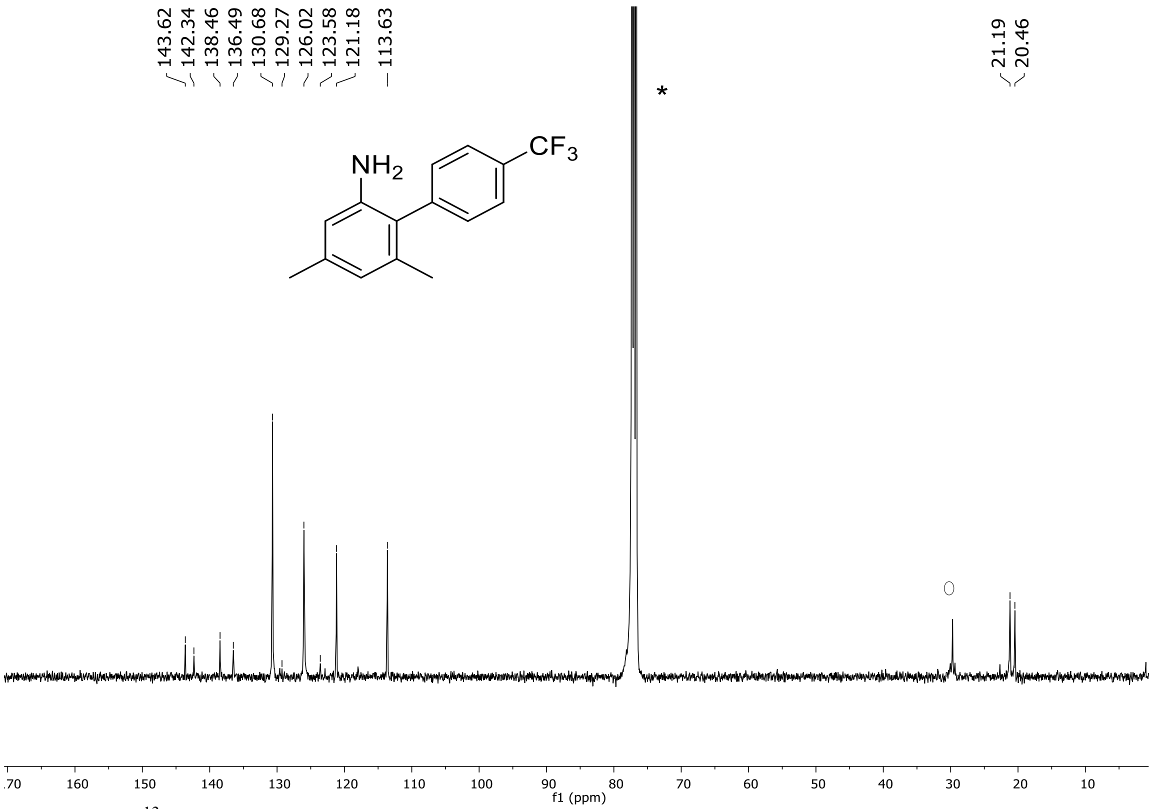


**Figure S71.** $^{13}C$ NMR (125.87 MHz, $CDCl_3$) of 4,6-dimethyl-4'-(trifluoromethyl)[1,1'-biphenyl]-2-amine (**5j**) at 298 K. (*) Signal corresponding to the solvent (chloroform). (○) Residual high MW paraffin.

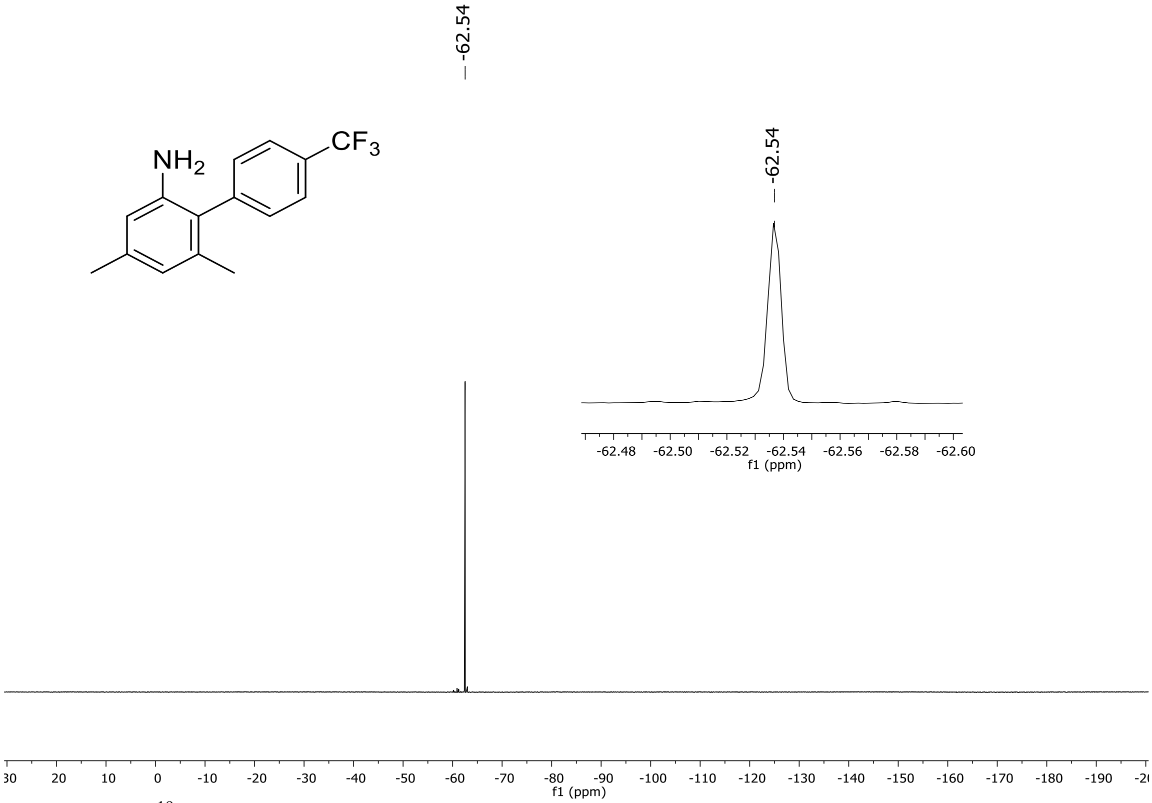


**Figure S72.** $^{19}$F NMR (470.168 MHz, $CDCl_3$) of 4,6-dimethyl-4'-(trifluoromethyl)[1,1'-biphenyl]-2-amine (**5j**) at 298 K.

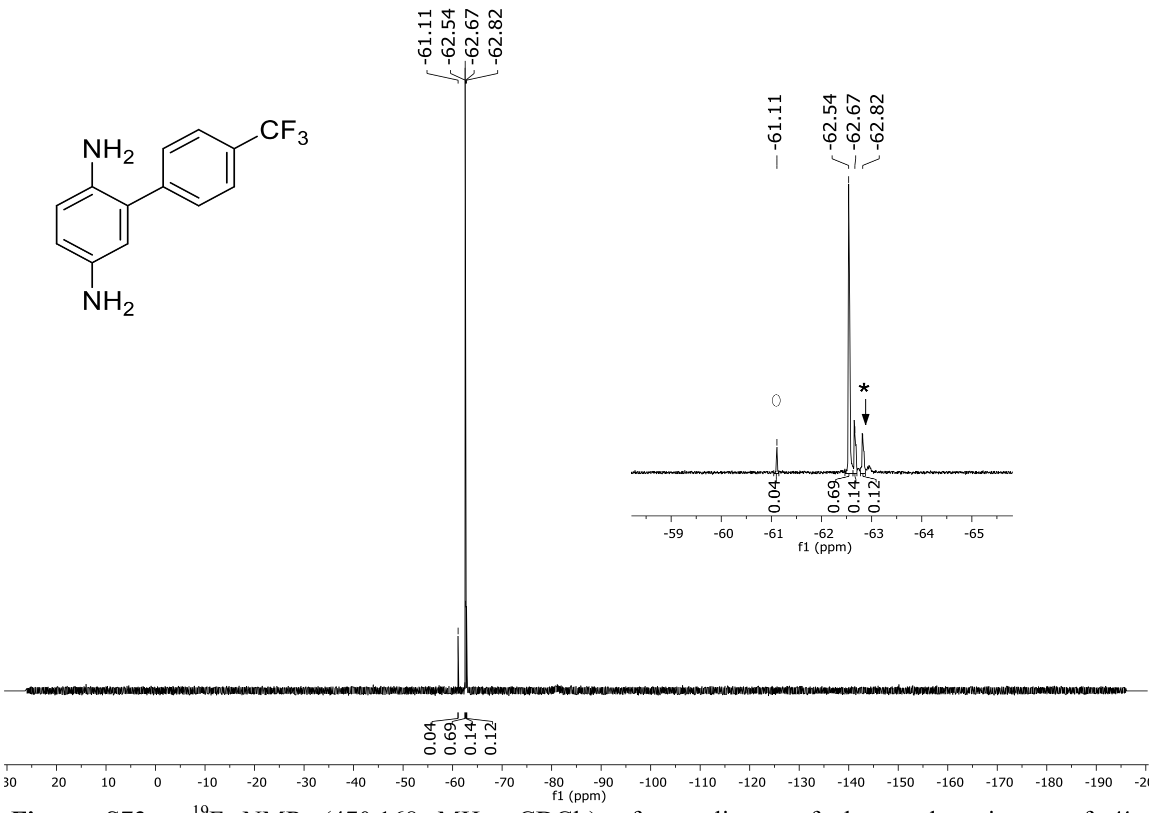


**Figure S73.** $^{19}$F NMR (470.168 MHz, $CDCl_3$) of an aliquot of the crude mixture of 4′-(trifluoromethyl)[1,1′-biphenyl]-2,5-diamine. (↓) Signals corresponding to aryl reduction. (*) Signals corresponding to the aryl homocoupling, (○) C-N coupling product.

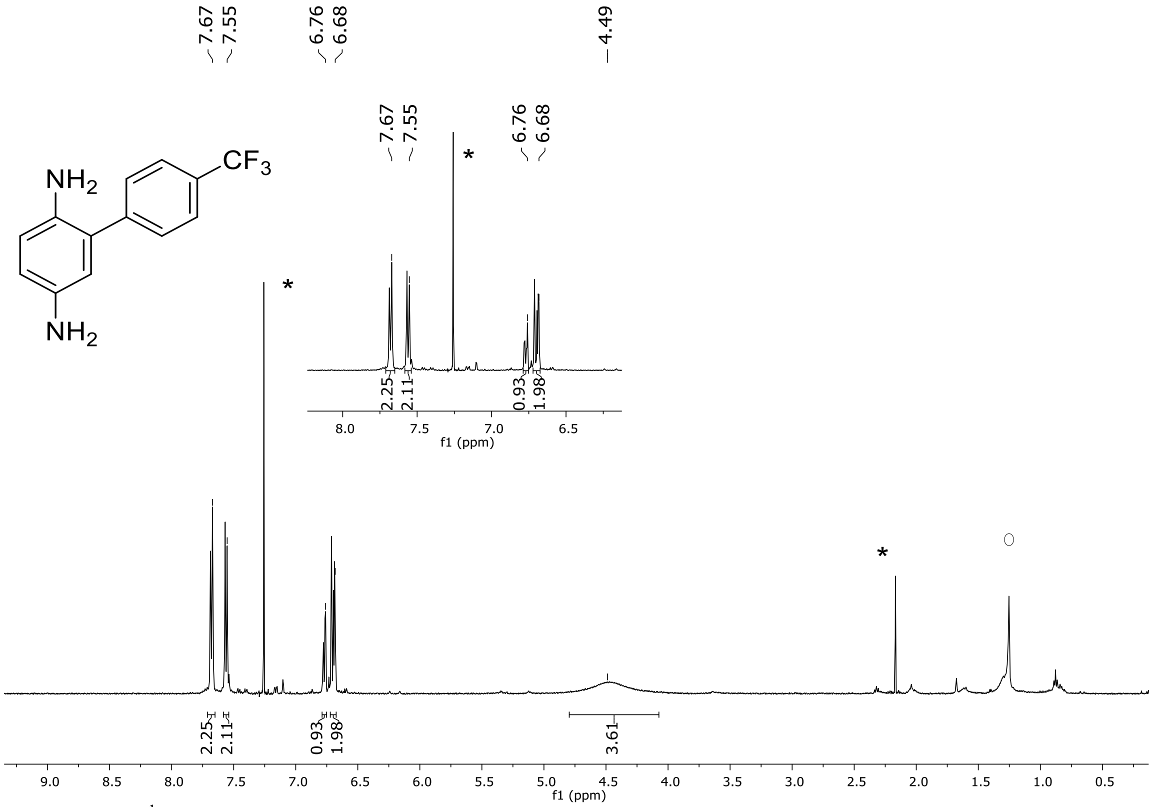


**Figure S74.** $^{1}H$ NMR (500.13 MHz, $CDCl_3$) of 4′-(trifluoromethyl)[1,1′-biphenyl]-2,5-diamine (**5k**) at 298 K. (*) Signal corresponding to the solvent (chloroform and acetone). (○) Residual high MW paraffin.

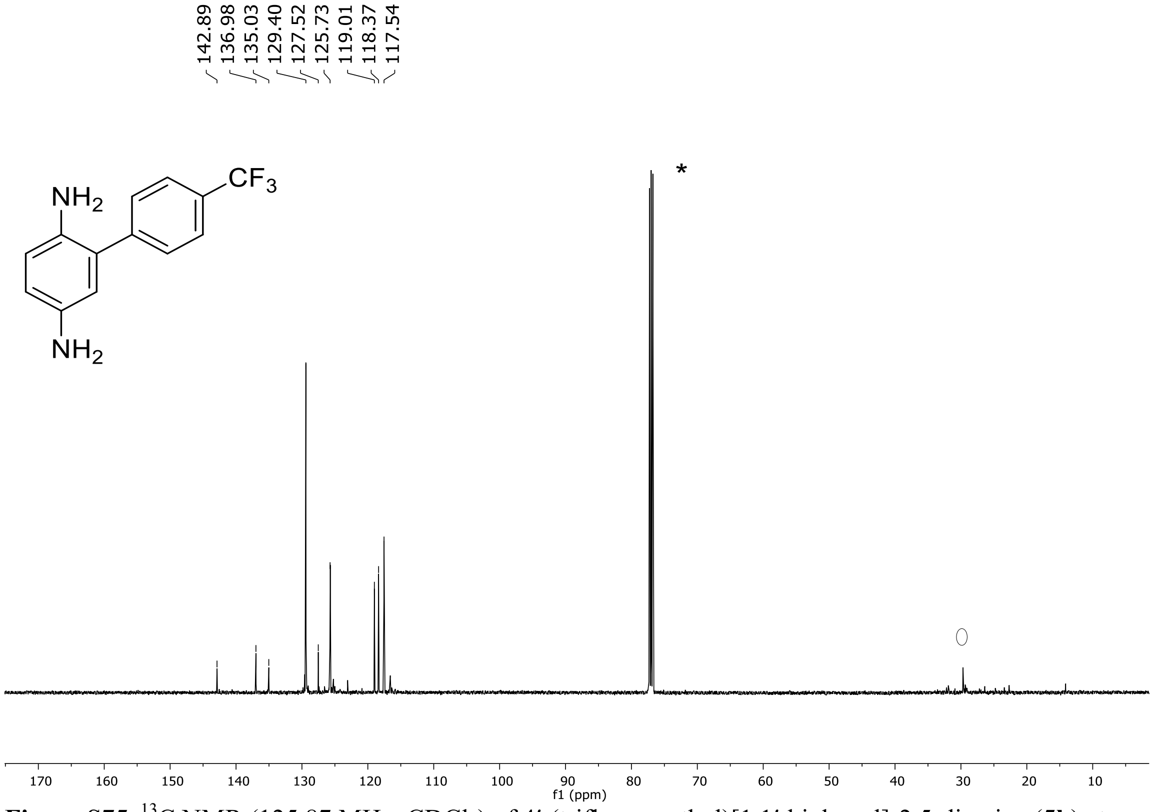


**Figure S75.** $^{13}C$ NMR (125.87 MHz, $CDCl_3$) of 4′-(trifluoromethyl)[1,1′-biphenyl]-2,5-diamine (**5k**) at 298 K. (*) Signal corresponding to the solvent (chloroform). (○) Residual high MW paraffin.

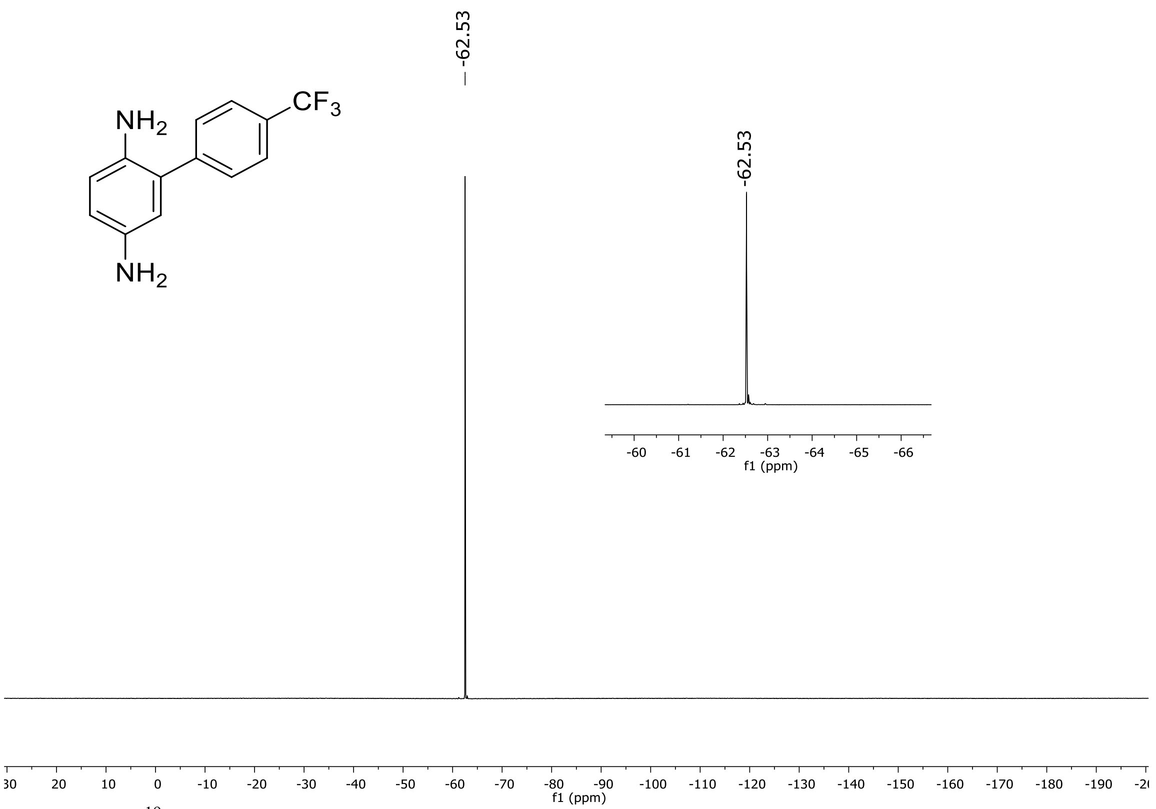


**Figure S76.** $^{19}$F NMR (470.168 MHz, $CDCl_3$) of 4′-(trifluoromethyl)[1,1′-biphenyl]-2,5-diamine (**5k**) at 298 K.

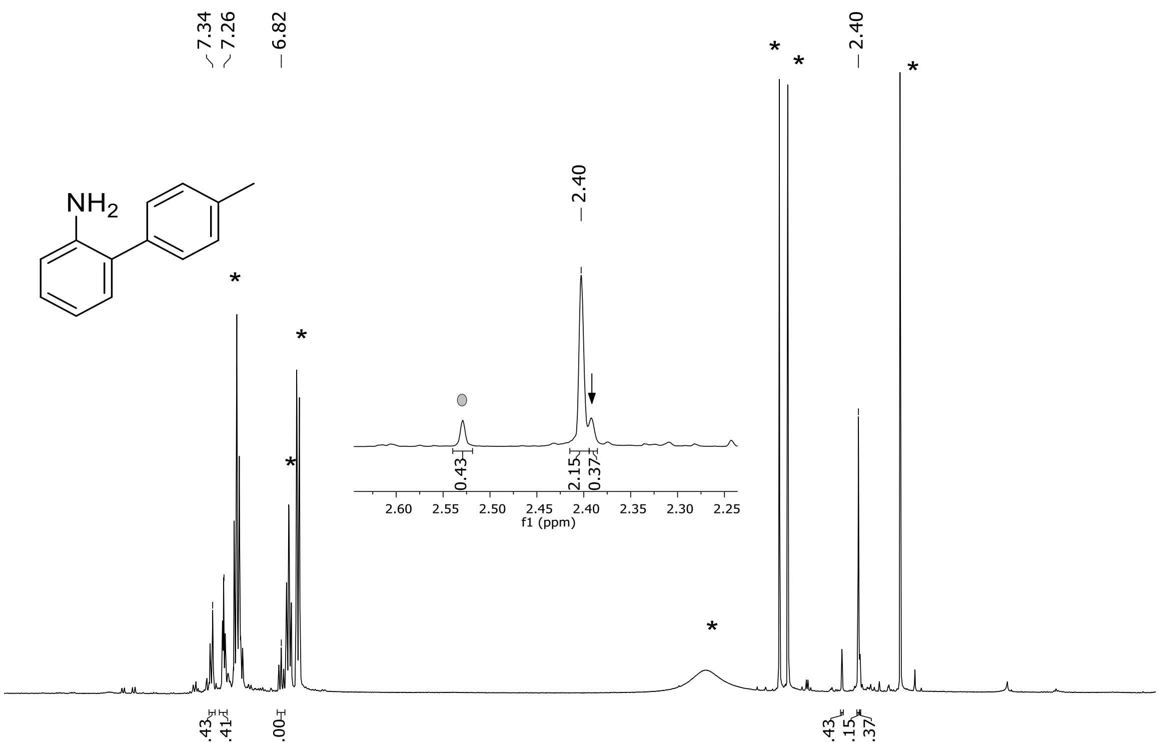


**Figure S77.** $^{1}$H NMR (500.13 MHz, DMA/$CDCl_3$) of an aliquot of the crude mixture of 4'-methyl-[1,1'-biphenyl]-2-amine. (↓) Signal corresponding to the aryl iodide. (○) Signal corresponding to minor unidentified compound. (*) Signals corresponding to the solvent (DMA and aniline).

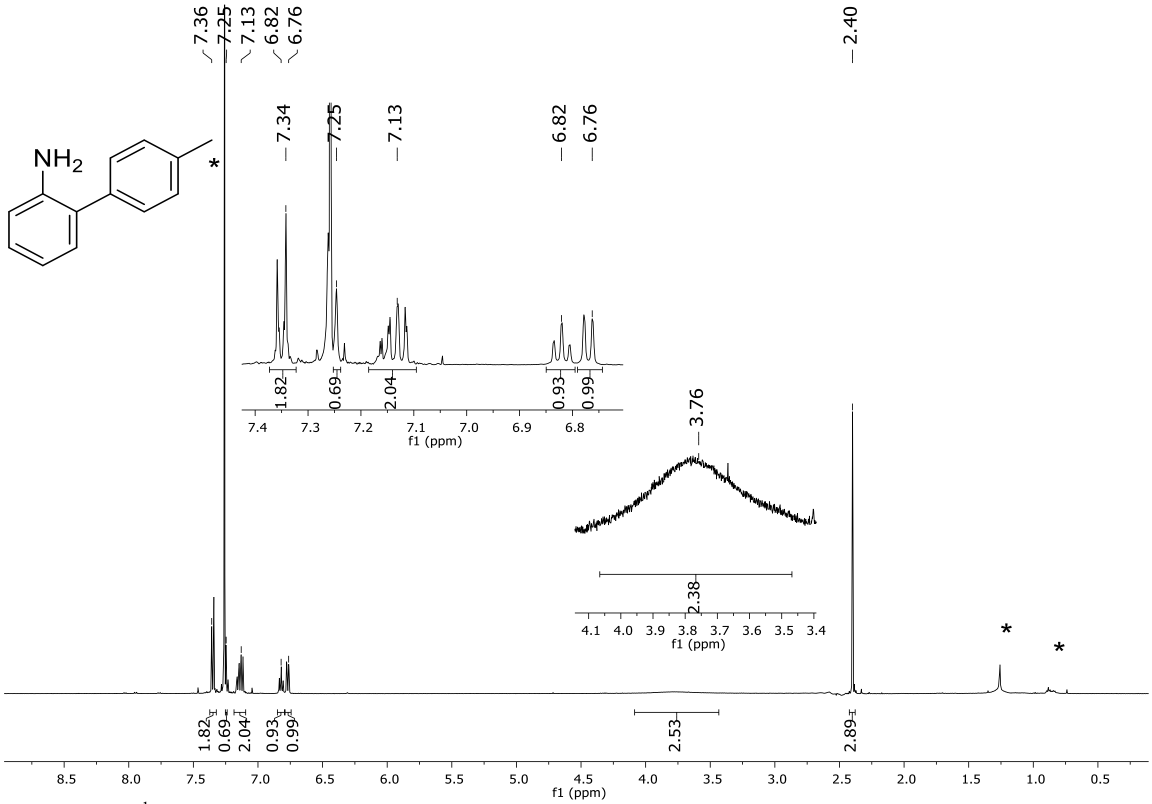


**Figure S78.** $^{1}H$ NMR (500.13 MHz, $CDCl_3$) of 4'-methyl-[1,1'-biphenyl]-2-amine (**5ab**) at 298 K. (*) Signals corresponding to the solvent (chloroform and hexane).

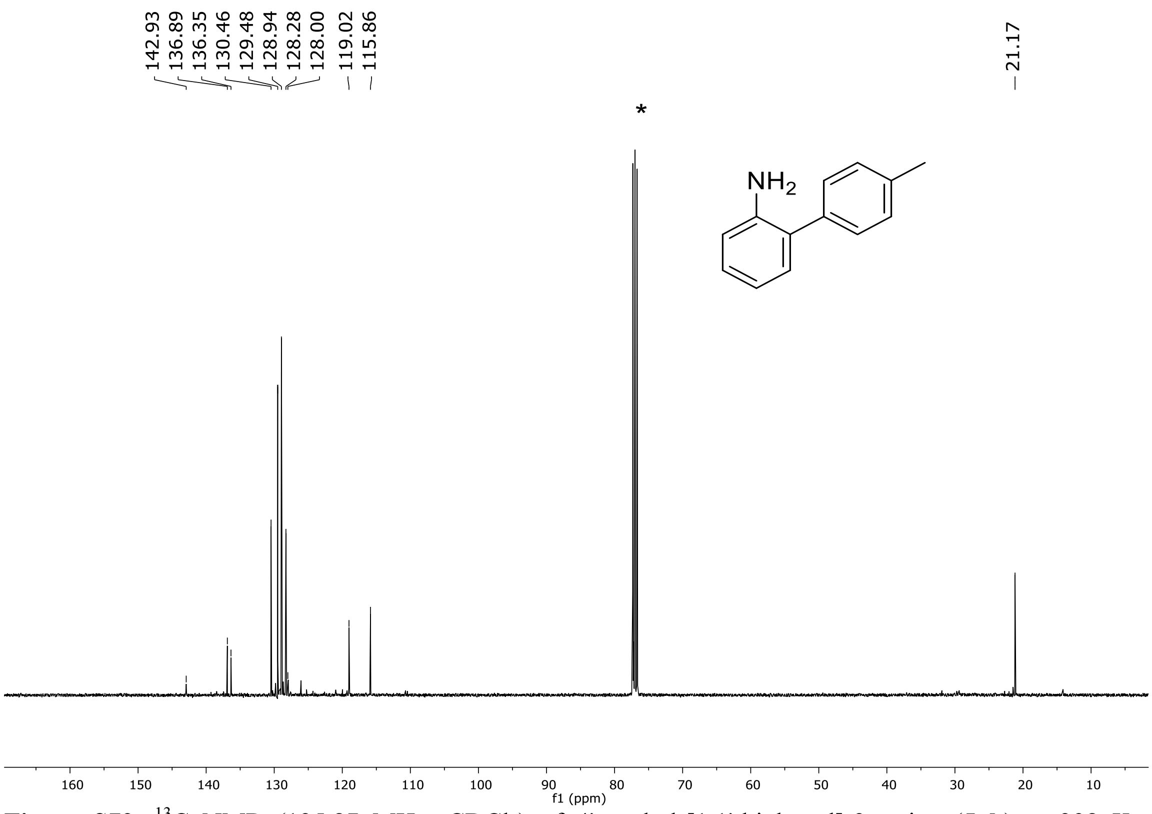


**Figure S79.** $^{13}C$ NMR (125.87 MHz, $CDCl_3$) of 4'-methyl-[1,1'-biphenyl]-2-amine (**5ab**) at 298 K. (*) Signal corresponding to the solvent (chloroform).

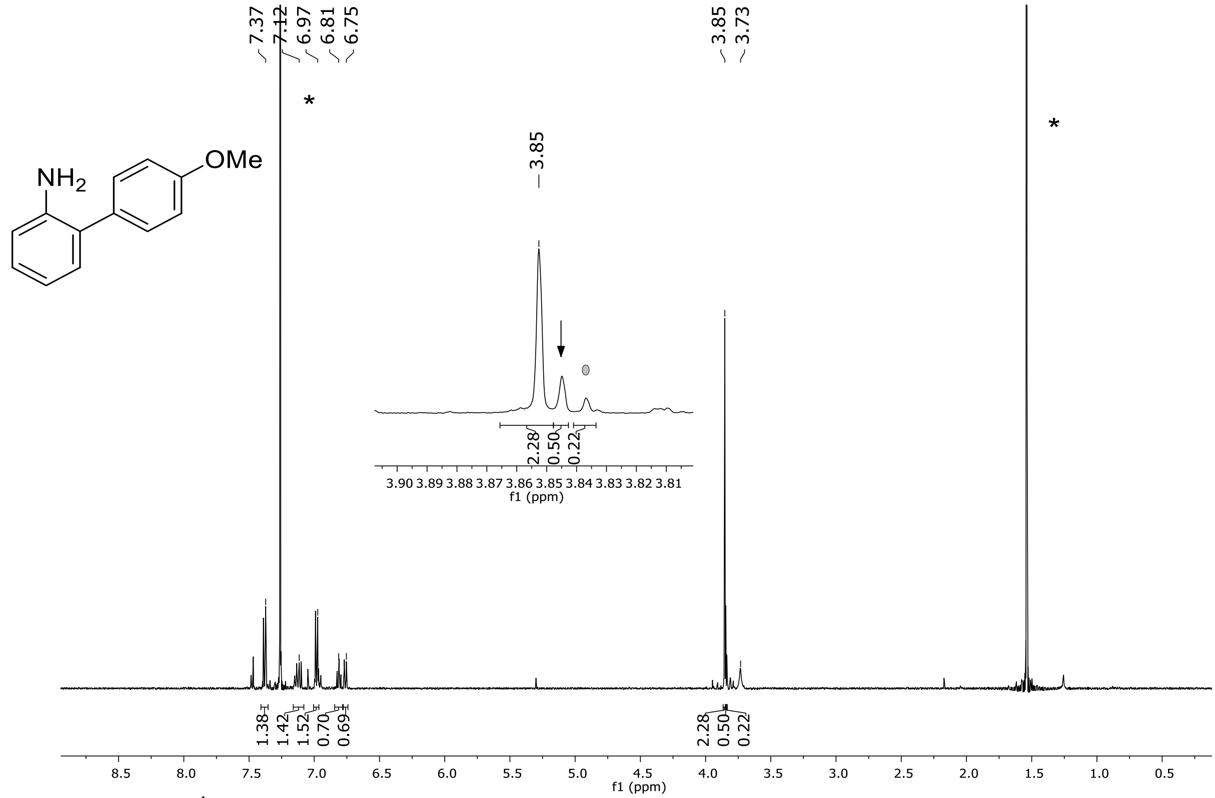


**Figure S80.** $^{1}$H NMR (500.13 MHz, $CDCl_3$) of the crude mixture of 4'-methoxy-[1,1'-biphenyl]-2-amine. (*) Signal corresponding to the solvent (chloroform and $H_2O$). (○) Signal corresponding to p-bromoanisole. (↓) Signal corresponding to minor unidentified product.

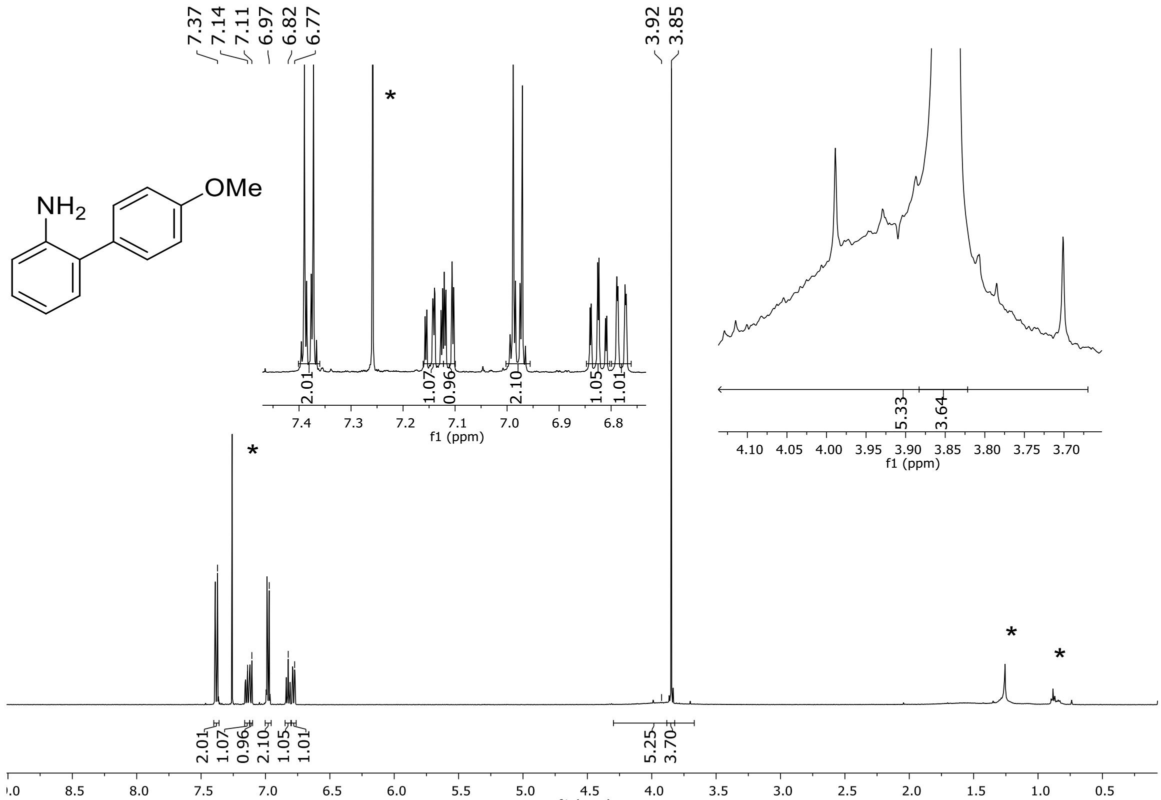


**Figure S81.** $^{1}$H NMR (500.13 MHz, $CDCl_3$) of 4'-methoxy-[1,1'-biphenyl]-2-amine (**5ac**) at 298 K. (*) Signal corresponding to the solvent (chloroform and hexane).

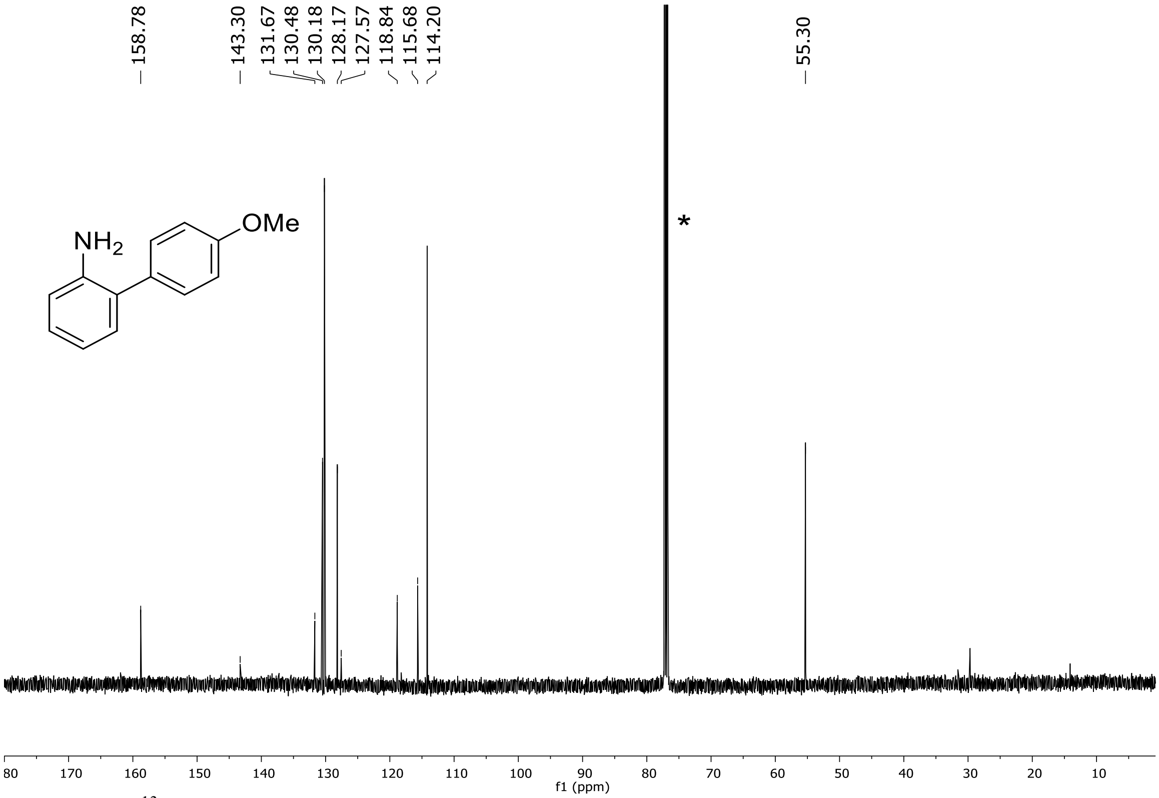


**Figure S82.**$^{13}$C NMR (125.87 MHz, $CDCl_3$) of 4'-methoxy-[1,1'-biphenyl]-2-amine (**5ac**) at 298 K. (*) Signal corresponding to the solvent (chloroform).

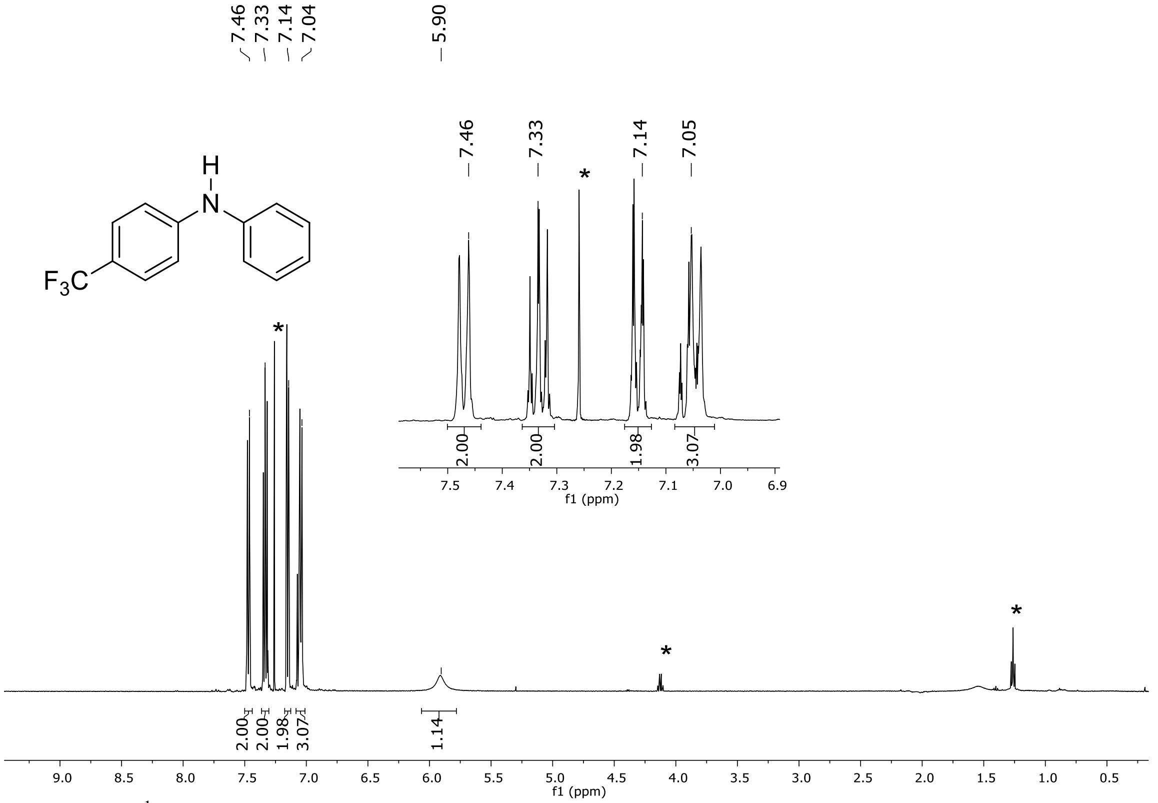


**Figure S83.** $^1H$ NMR (500.13 MHz, $CDCl_3$) of N-phenyl-4-(trifluoromethyl)benzenamine (**6a**) at 298 K. (*) Signal corresponding to the solvent (chloroform and ethyl acetate).

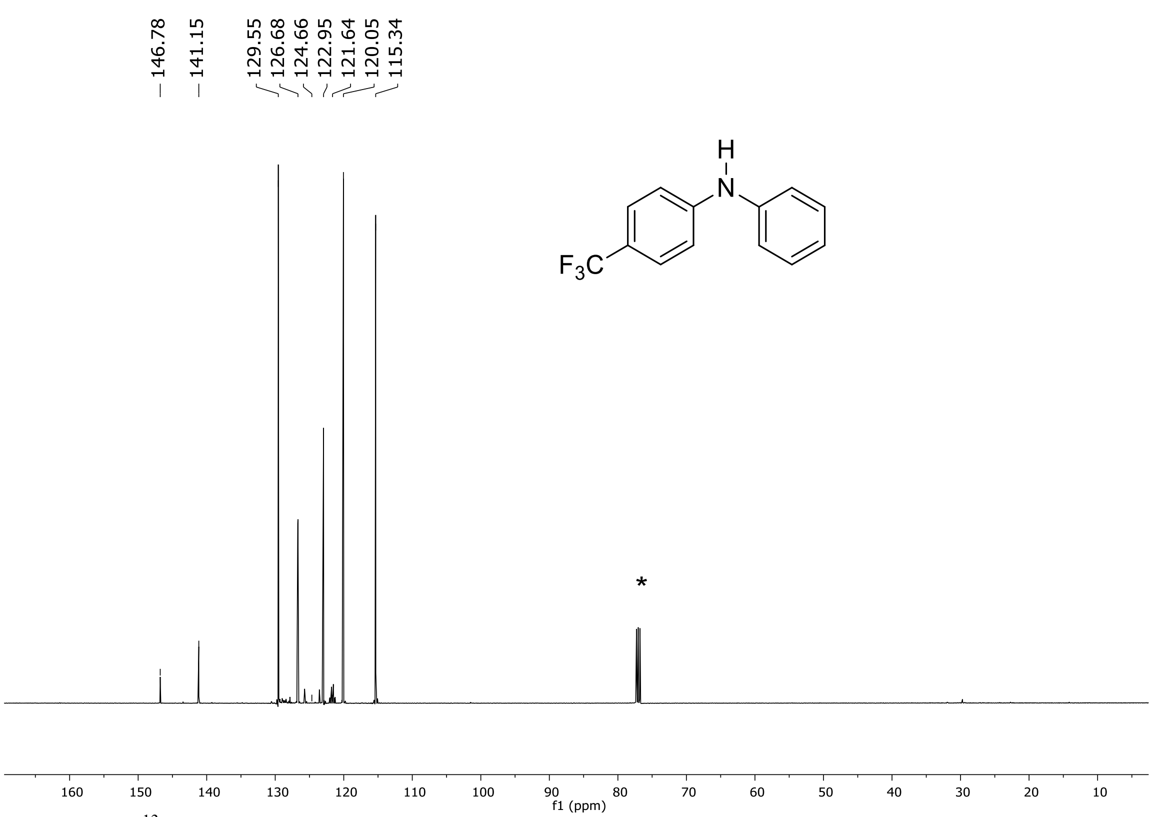


**Figure S84.** $^{13}C$ NMR (125.87 MHz, $CDCl_3$) of N-phenyl-4-(trifluoromethyl)benzenamine (**6a**) at 298 K. (*) Signal corresponding to the solvent (chloroform).

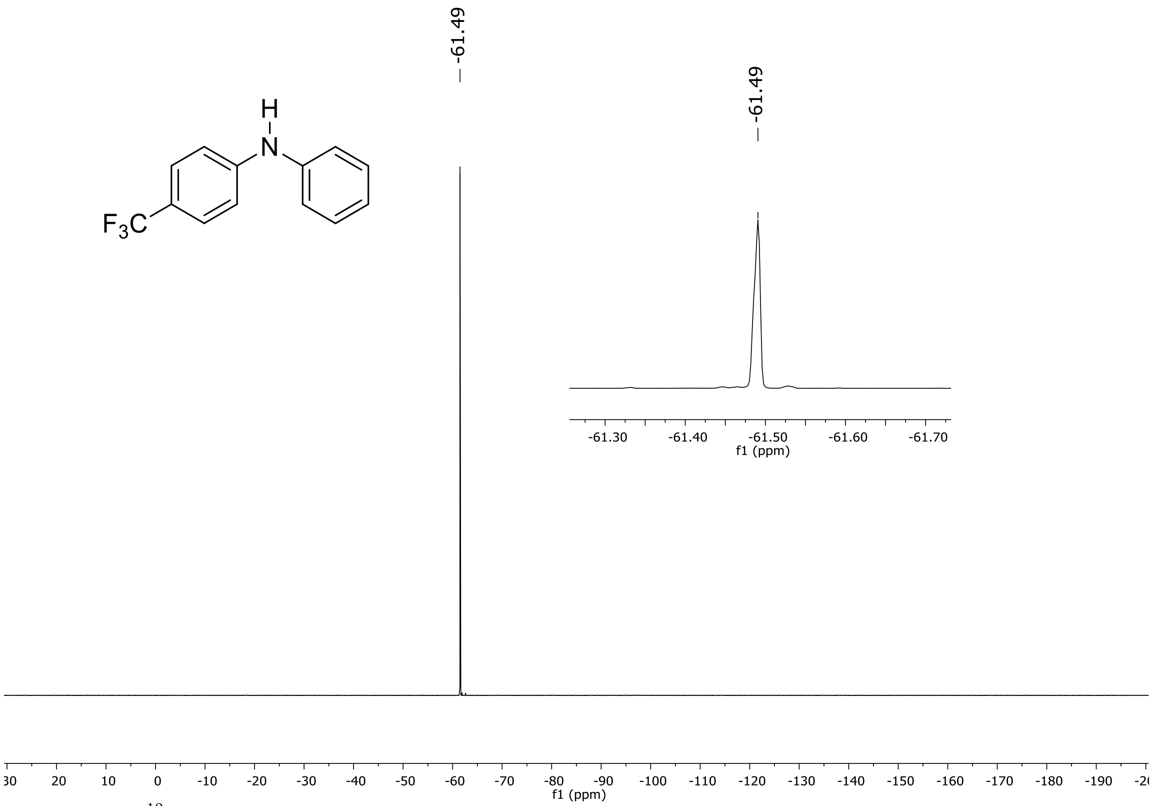


**Figure S85.** $^{19}F$ NMR (470.168 MHz, $CDCl_3$) of N-phenyl-4-(trifluoromethyl)benzenamine (**6a**) at 298 K.

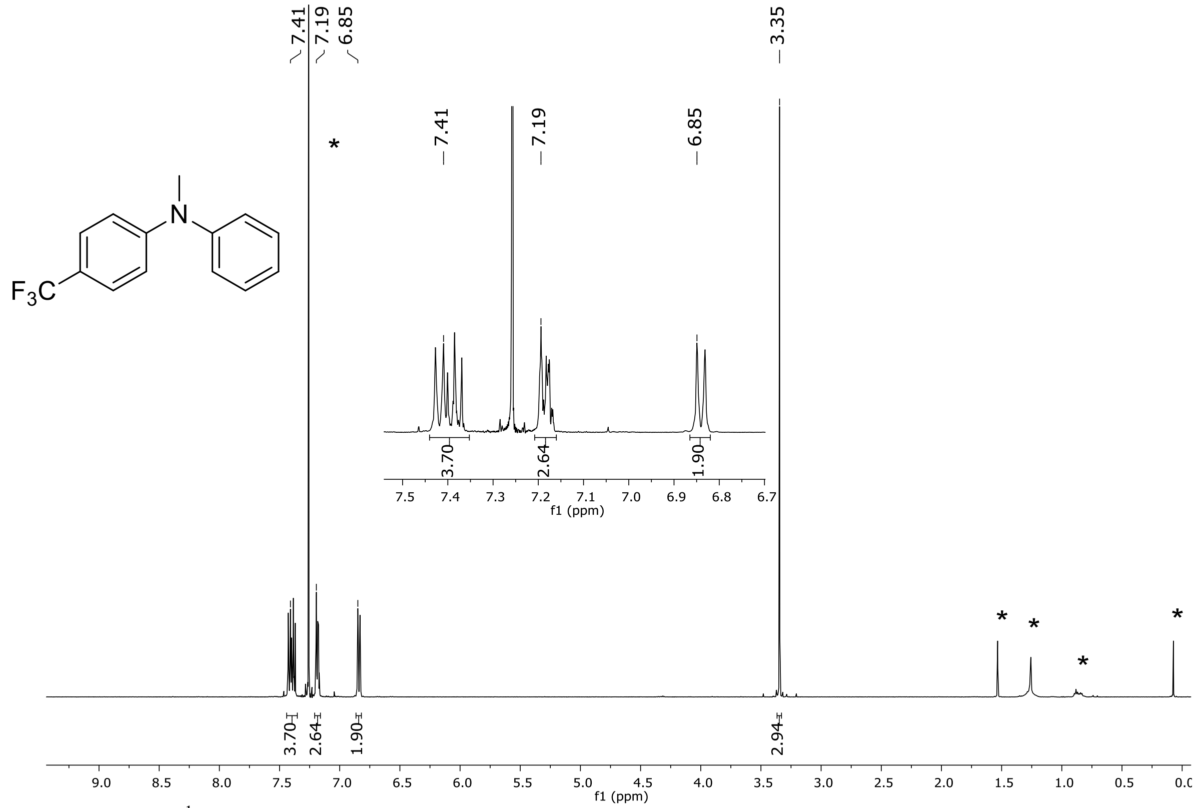


**Figure S86.** $^1$H NMR (500.13 MHz, $CDCl_3$) of N-methyl-N-phenyl-4-(trifluoromethyl)benzenamine (**6b**) at 298 K. (*) Signal corresponding to the solvent (chloroform, hexane, $H_2O$ and grease).

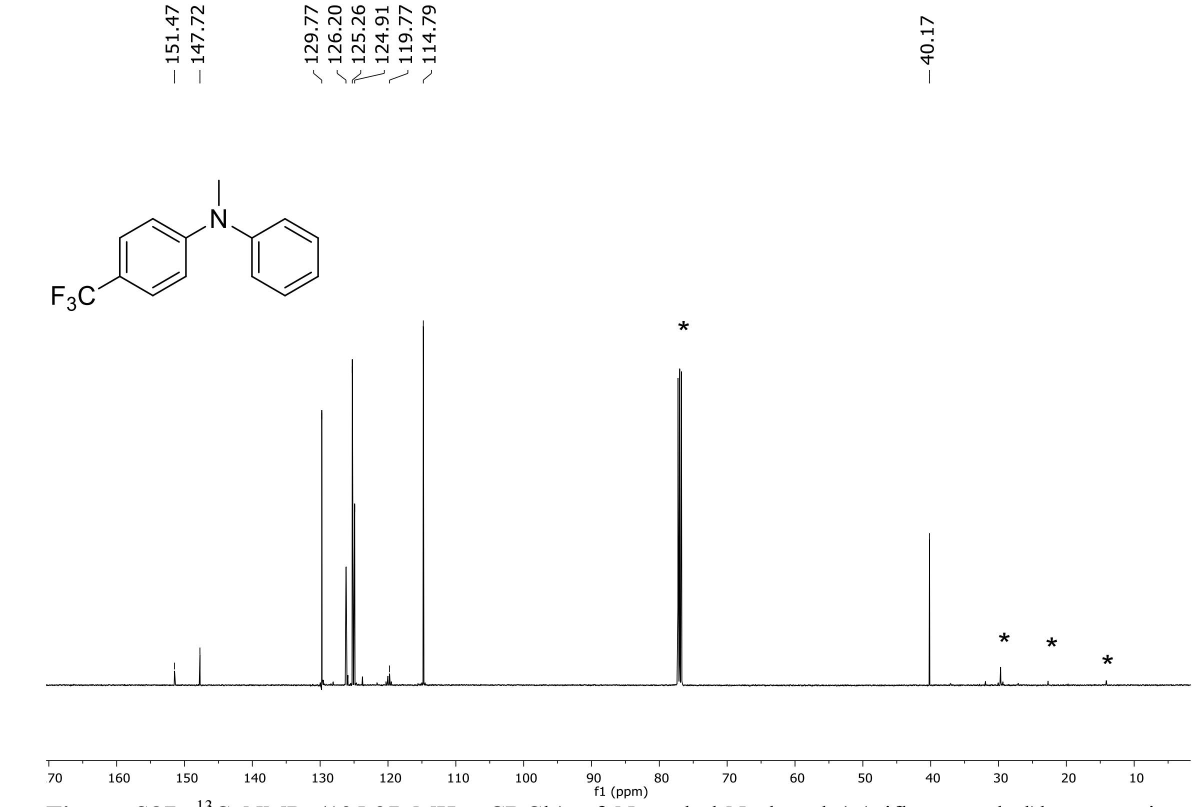


**Figure S87.** $^{13}$C NMR (125.87 MHz, $CDCl_3$) of N-methyl-N-phenyl-4-(trifluoromethyl)benzenamine (**6b**) at 298 K. (*) Signal corresponding to the solvent (chloroform, hexane and grease).

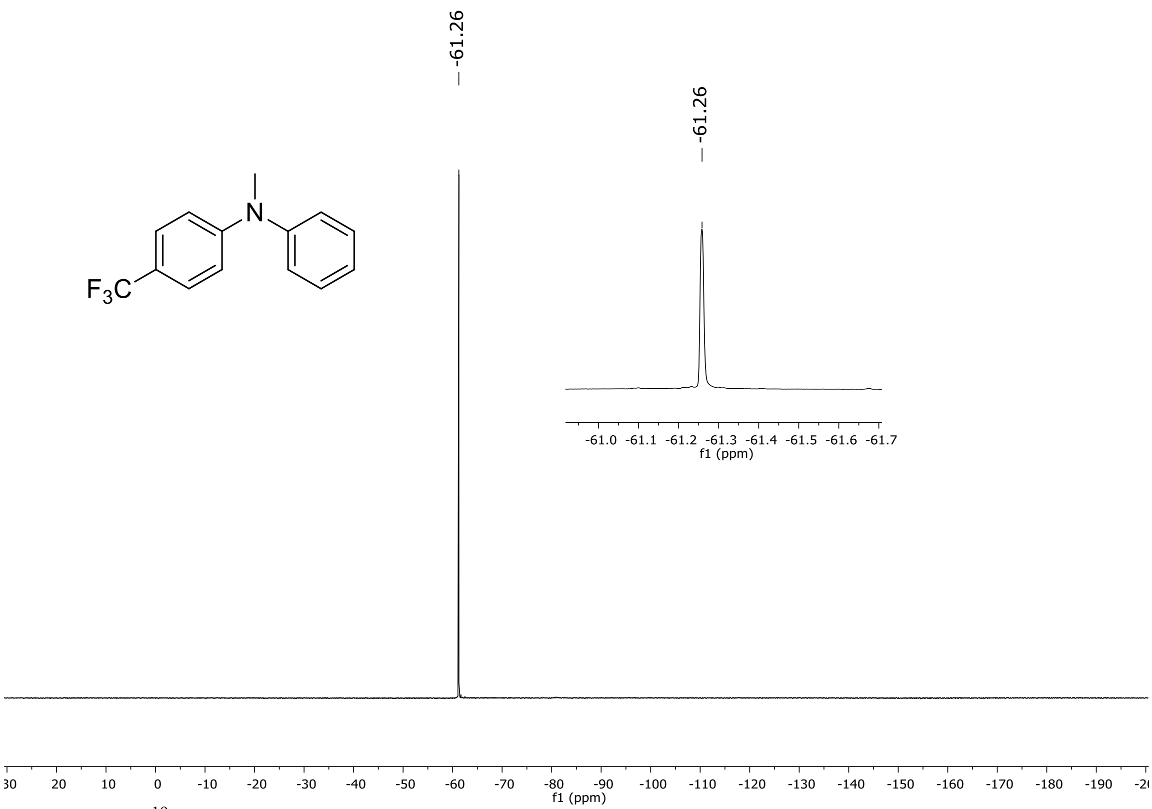


**Figure S88.** $^{19}$F NMR (470.168 MHz, $CDCl_3$) of N-methyl-N-phenyl-4-(trifluoromethyl)benzenamine (**6b**) at 298 K.

## *References*


(1) Salamanca, V.; Toledo, A.; Albéniz, A. C. [2,2′-Bipyridin]-6(1*H*)-one, a Truly Cooperating Ligand in the Palladium-Mediated C−H Activation Step: Experimental Evidence in the Direct C-3 Arylation of Pyridine, *J. Am. Chem. Soc.* **2018**, *140*, 17851-17856. DOI: 10.1021/jacs.8b10680.

(2) Espinet, P.; Albéniz, A. C.; Usón, R.; Forniés, J.; Nalda, J. A.; Lozano, M. J. Synthesis of $(NBu_4)_2[Pd(\mu\text{-}Br)_2(C_6X_5)_2Br_2]$ (X = F, Cl), New and More Versatile Precursors of Pentahalophenyl Derviatives of Palladium(II). *Inorg. Chim. Acta*, **1989**, *156*, 251-256. DOI: 10.1016/S0020-1693(00)83508-2.

(3) Villalba, F.; Albéniz, A. C. Non-Chelate-Assisted Palladium-Catalyzed Aerobic Oxidative Heck Reaction of Fluorobenzenes and Other Arenes: When Does the C–H Activation Need Help?. *Adv. Synth. Catal.* **2021**, *363*, 1-11. DOI: 10.1002/adsc.202100677.

(4) Ishii, Y.; Hasegawa, S.; Kimura, S.; Itoh, K. Novel Trinuclear Palladium(0) Complexes: Tris(tribenzylideneacetylacetone)tripalladium(solvent) Complexes and Their Reactions. *J. Organomet. Chem.* **1974**, *73*, 411-418. DOI:10.1016/S0022-328X(00)83841-3.

(5) Yamashita, M.; Cuevas Vicario, J.; Hartwig, J. F. Trans Influence on the Rate of Reductive Elimination. Reductive Elimination of Amines from Isomeric Arylpalladium Amides with Unsymmetrical Coordination Spheres. *J. Am. Chem. Soc.* **2003**, *125*, 16347-16360. DOI: 10.1021/ja037425g.

(6) Tomon, T.; Koizumi, T.; Tanaka, K. Stabilization and Desestabilization of the Ru-CO bond During the 2,2′-Bipyridin-6-onato (bpyO)-Localized Redox Reaction of $[Ru(terpy)(bpyO)(CO)](PF_6)$. *Eur. J. Inorg. Chem.* **2005**, 285-293. DOI: 10.1002/ejic.200400522.

(7) Salamanca, V.; Albéniz, A. C. Faster palladium-catalyzed arylation of simple arenes in the presence of a methylketone: beneficial effect of an *a priori* interfering solvent in C–H activation. *Org. Chem. Front.* **2021**, *8*, 1941-1951. DOI: 10.1039/d1qo00236h.

(8) Chen, H.; Wang, L.; Han, J. Aryl radical-induced desulfonylative ipso-substitution of diaryliodonium salts: an efficient route to sterically hindered biarylamines. *Chem. Commun.*, **2020**, *56*, 5697-5700. DOI: 10.1039/d0cc01766c.

(9) Benting, J.; Desbordes, P.; Gary, S.; Greul, J.; Tsuchiya, T.; Wachendorff-Neumann, U. N-Aryl Pyrazole(thio)carboxamides. US 9206137 B2, 2015.

(10) Parrish, C. A.; Adams, N. D.; Auger, K. D.; Burgess, J. L.; Carson, J. D.; Chaudhari, A. M.; Copeland, R. A.; Diamond, M. A.; Donatelli, C. A.; Duffy, K. J.; Faucette, L. F.; Finer, J. T.; Huffman, W. F.; Hugger, E. D.; Jackson, J. R.; Knight, S. D.; Luo, L.; Moore, M. L.; Newlander, K. A.; Ridgers, L. H.; Sakowicz, R.; Shaw, A. N.; Sung, C. M.; Sutton, D.; Wood, K. W.; Zhang, S. –Y-; Zimmerman, M. N.; Dhanak, D. Novel ATP-Competitive Kinesin Spindle Protein Inhibitors. *J. Med. Chem.* **2007**, *50*, 4939-4952. DOI: 10.1021/jm070435y.

(11) Dai, W.; Yang, B.; Xu, S.; Wang, Z. Nickel-Catalyzed Cross-Coupling of Aryl 2□Pyridyl Ethers with Organozinc Reagents: Removal of the Directing Group via Cleavage of the Carbon−Oxygen Bonds. *J. Org. Chem.* **2021**, *86*, 2235-2243. DOI: 10.1021/acs.joc.0c02389.

(12) Zuo, Z.; Liu, J.; Nan, J.; Fan, L.; Sun, W.; Wang, Y.; Luan, X. Highly Stereoselective Synthesis of Imine-Containing Dibenzo-[b,d]azepines by a Palladium(II)-Catalyzed [5+2] OxidativeAnnulation of o-Arylanilines with Alkynes. *Angew. Chem. Int. Ed.* **2015**, *54*, 15385-15389. DOI: 10.1002/anie.201508850.

(13) Gillespie, J. E.; Morrill, C.; Phipps, R. J. Regioselective Radical Arene Amination for the Concise Synthesis of ortho-Phenylenediamines. *J. Am. Chem. Soc.* **2021**, *143*, 9355-9360. DOI: 10.1021/jacs.1c05531.

(14) Maity, A.; Frey, B. L.; Hoskinson, N. D.; Powers, D. C. Electrocatalytic C−N Coupling via Anodically Generated Hypervalent Iodine Intermediates. *J. Am. Chem. Soc.* **2020**, *142*, 4990-4995. DOI: 10.1021/jacs.9b13918.

(15) (a) Ohshita, K.; Ishiyama, H.; Oyanagi, K.; Nakatab, H.; Kobayashi, J. Synthesis of hybrid molecules of caffeine and eudistomin D and its effects on adenosine receptors. *Bioorg. Med. Chem.* **2007**, 15, 3235-3240. DOI: 10.1016/j.bmc.2007.02.043. (b) Surry, D. S.; Buchwald, S. L. Biaryl Phosphane Ligands in Palladium-Catalyzed Amination. *Angew. Chem. Int. Ed.* **2008**, *47*, 6338-6361. DOI: 10.1002/anie.200800497.

(16) (a) Sharma, C.; Srivastava, A. K.; Sharma, K. S.; Joshi, R. K. Half-sandwich (η5-Cp*)Rh(III) complexes of pyrazolated organo-sulfur/selenium/tellurium ligands: efficient catalysts for base/solvent free C–N coupling of chloroarenes under aerobic conditions. *Org. Biomol. Chem.*, **2020**,*18*, 3599-3606. DOI: 10.1039/d0ob00538j. (b) Srivastava, A. K.; Sharma, C.; Joshi, R. K. Cp*Co(III) and Cu(OAc)2 bimetallic catalysis for Buchwald-type C–N cross coupling of aryl chlorides and amines under base, inert gas & solvent-free conditions. *Green Chem.*, **2020**, *22*, 8248-8253. DOI: 10.1039/d0gc02819c.

(17) (a) Li, J.; Huang, C.; Wen, D.; Zheng, Q.; Tu, B.; Tu, T. Nickel-Catalyzed Amination of Aryl Chlorides with Amides. *Org. Lett.* **2021**, *23*, 687-691. DOI: 10.1021/acs.orglett.0c03836. (b) Tröndle, S.; Freytag, M.; Jones, P. G.; Tamm, M. Allyl Palladium Complexes of Cycloheptatrienyl-Cyclopentadienyl Phosphane Ligands in Buchwald-Hartwig Amination Reactions. *Eur. J. Inorg. Chem.* **2019**, 2569-2576. DOI: 10.1002/ejic.201900225.

(18) Fuchita Y.; Tsuchiya, H.; Miyafuji, A. Cyclopalladation of secondary and primary benzylamine. *Inorg. Chim. Acta.* **1995**, *233*, 91-96. DOI: 10.1016/0020-1693(94)04440-7.

(19) a) Burés, J., Variable Time Normalization Analysis: General Graphical Elucidation of Reaction Orders from Concentration Profiles, *Angew. Chem. Int. Ed.* **2016**, *55*, 16084-16087. DOI: 10.1002/anie.201609757. b) Burés, J., A Simple Graphical Method to Determine the Order in Catalyst, *Angew. Chem. Int. Ed.* **2016**, *55*, 2028-2031. DOI: 10.1002/anie.201508983.

(20) CrysAlisPro Software system, version 1.171.33.51, **2009**, Oxford Diffraction Ltd, Oxford, UK.

(21) Sheldrick, G. M. Crystal structure refinement with SHELXL. *Acta Cryst.* **2015**, *C71*, 3-8. DOI: 10.1107/S2053229614024218.

(22) Dolomanov, O. V.; Bourhis, L. J.; Gildea, R. J.; Howard J. A. K.; Puschmann, H. OLEX2: a complete structure solution, refinement and analysis program. *J. Appl. Crystallogr.* **2009**, *42*, 339-341. DOI: 10.1107/S0021889808042726.
