# Supplementary material for: Palladium-Catalyzed Ortho C–H Arylation of Unprotected Anilines: Chemo- and Regioselectivity Enabled by the Cooperating Ligand [2,2′-Bipyridin]-6(1H)-one
Source: ACS Catal. 2022 Nov 11;12(23):14527–32. doi: 10.1021/acscatal.2c05206 (PMC9724229; doi:10.1021/acscatal.2c05206)
Supplement: Supplementary file 2 — cs2c05206_si_002.pdf [file cs2c05206_si_002.pdf]

Supporting information

Palladium-Catalyzed *Ortho* C–H Arylation of Unprotected Anilines: Chemo- and Regioselectivity Enabled by the Cooperating Ligand [2,2′-bipyridin]-6(1*H*)-one

Cintya Pinilla,[a] Vanesa Salamanca,[a] Agustí Lledós[b]* and Ana C Albéniz[a]*

[a] *IU CINQUIMA/Química Inorgánica, Universidad de Valladolid, 47071 Valladolid, Spain. E-mail: albeniz@uva.es*

[b] *Departament de Química, Universitat Autònoma de Barcelona, Barcelona 08193, Spain. Email: agusti.lledos@uab.cat*

PART 2

4. Computational details
   4.1 Computational methods
   4.2 Benchmarking of the chemical and computational models
   4.3 Gibbs energy profiles.
   4.4 Microkinetic modeling
   4.5 Probing the intermediacy of Pd(IV) species.
   4.6 3D-view of selected structures in the energy profiles.
   4.7 Absolute energies of all the calculated species (atomic units).
   4.8 Cartesian coordinates and electronic energies (M06/BS1/SMD) of all the calculated species.

References

4. Computational Details

4.1- Computational methods.

Geometry optimizations and thermochemical corrections: Density Functional Theory (DFT) calculations

Exploration of the potential energy surface, with location of minima and transition states has been carried out using the Gaussian16 software[23] at DFT level of theory with the M06 functional.[24] Basis set 1 (BS1) was employed in the optimizations, consisting of the 6-31+G(d) basis set for C, O, N, F and H,[25] and LANL2TZ(f) basis set for Pd and Cs[26] with the corresponding Hay-Wadt effective core potential (ECP).[27] All structure optimizations were carried out in solvent phase with no symmetry restrictions. Solvent effects have been considered in all the optimizations through a cluster/continuum solvation model,[28] that includes the continuum model SMD[29] for the experimental solvent, *N, N*-dimethylacetamide (DMA, ε = 37.781 at 25 ºC), and a few (4) explicit DMA molecules. For each structure several conformational isomers, which differ in the positions of the cesium and carbonate ions, as well as of DMA solvent molecules, were computed. The lowest energy isomers are collected in the article.

Vibrational frequency calculations were performed for all the optimized geometries to characterize the stationary points as either minima (without imaginary frequencies) or transition states (with one imaginary frequency). Connectivity of the transition state structures was confirmed by relaxing the transition state geometry towards both the reactant and the product. Thermal and entropic corrections were calculated at 403.15 K (the experimental temperature) from the unscaled harmonic vibrational frequencies at this level of theory.

Single point DLPNO-CCSD(T) energy calculations

To obtain accurate electronic energies, single point calculations were performed on all the M06 optimized structures with the domain based local pair natural orbital coupled-cluster ((DLPNO-CCSD(T)) method[30] as implemented in ORCA package.[31] The def2-TZVP basis set[32] and the RIJCOSX approximation[33] along with def2-ECP for Pd and Cs[34] were used in all single point calculations.

Gibbs energies in solution

All the energies reported in the article are Gibbs energies in solution at 403.15 K. They were calculated by combining the electronic energy computed by means of single point

DLPNO-CCSD(T) electronic energy calculations ($E_{el/gas}^{DLPNO-CCSD(T)/SP}$) with the thermal and entropic corrections evaluated at the geometry optimization level of theory (M06) ($(G\text{-}E)^{M06/opt}$) and Gibbs energies of solvation (ΔG_{solv}), computed as the energy difference between the single point energy calculated at M06 level of theory with and without the SMD continuum model ($E_{el/solv}^{M06} - E_{el/gas}^{M06}$). A correction of 2.56 kcal mol^{-1} ($\Delta G^{0\rightarrow *}$) was applied to all Gibbs energy values to change the standard state from the gas phase (1 atm) to solution (1M) at 430.15 K.[35] In this way, the Gibbs energy in DMA solution for each species is computed as:

$$G_{sol} = E_{el/gas}^{DLPNO-CCSD(T)/SP} + (G - E_{el})^{M06/opt} + (E_{el/solv}^{M06} - E_{el/gas}^{M06}) + \Delta G^{0\rightarrow *}$$

3D chemical structures were generated using CYLview.[36]

4.2- Benchmarking of the chemical and computational models

Initially, we performed single point calculations using the functional employed in the optimizations (M06), adding long-range dispersion effects (M06-D3) and an extended basis set (BS2), considering the simple model commonly employed in this kind of studies (the palladium ion and its ligands: complex **8**). DLPNO-CCSD(T) calculations were used as a benchmark. Table S7 collects the Gibbs energy barriers obtained for the two steps of the reaction: C-H bond activation and C-C reductive elimination, at different levels of calculation. Optimized geometries, obtained with M06/BS1 were used in the single point calculations.

Table S7. Gibbs energy barriers, using different computational methods, for the C-H bond activation and C-C reductive elimination steps using a simple model, not taking into account neither carbonate salt nor explicit solvent molecules.[a]

	**M06/BS1**	**M06-D3/BS2**	**DLPNO-CCSD(T)/BS2**
	**CH Bond Activation step**		
ortho	27.1	26.3	28.8
meta	32.4	30.9	32.2
para	27.9	27.0	29.3
	**C-C Reductive Elimination step**		
ortho	27.6	27.5	36.2
meta	26.9	26.5	34.7
para	26.3	26.2	33.6

a) Energies, in kcal mol^{-1}, are given taking complex **8** as reference (0.0 kcal mol^{-1}).

These results reveal that, although M06 barriers for the CH bond activation step are only a little lower than those for DLPNO-CCSD(T) (about 2 kcal mol^{-1}), the energies of the reductive elimination step show a marked deviation (about 8 kcal mol^{-1}). From these results we decided to use DLPNO-CCSD(T) as the method at choice to estimate accurate energies.

However, as it can be seen in Figure S89, even with this method the simple model (no carbonate salts or explicit solvent molecules) does not reproduce the experimental results, since it predicts a turnover limiting reductive elimination instead of the observed

C-H cleavage and it disfavors the *ortho* regioisomer, which is the one observed experimentally.

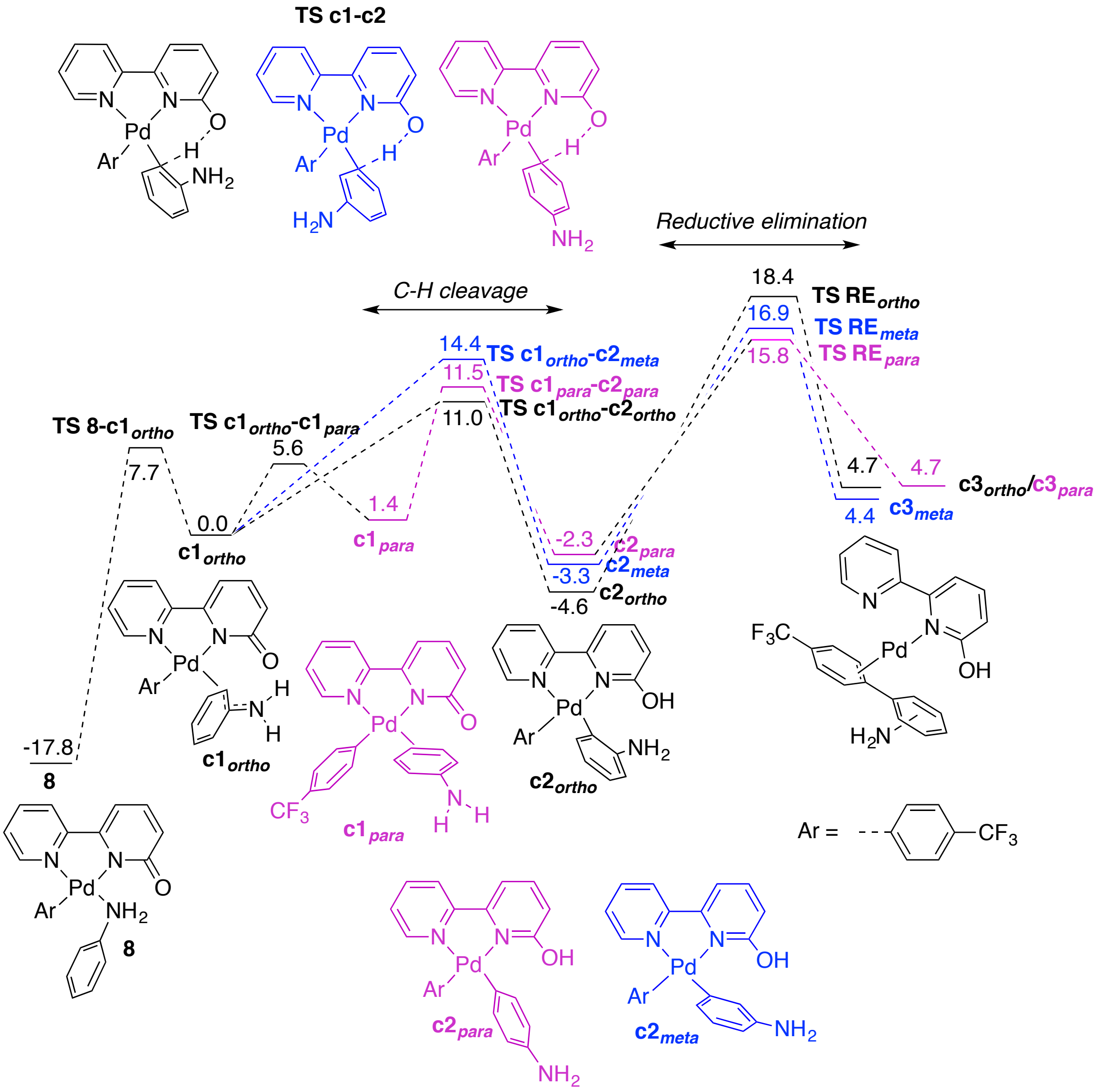

Figure S89. Gibbs energy profile for the arylation reaction of aniline (C-H cleavage and reductive elimination steps) using a simple model, not taking into account carbonate salts or solvent molecules. Data for the three regioisomers are shown. Energies in kcal mol^{-1}. No minimum has been found for a **c1**$_{meta}$ intermediate.

Another important feature of the reaction is the prevalence of the C-C coupling over the Buchwald-Hartwig C-N coupling. To benchmark the chemical model to be included in the calculations we scrutinized how this model affects the relative barriers of the C-H cleavage and C-N coupling steps. To do that, we introduced complexity in the model sequentially. Four models were checked. Model 1 is the simplest model: deprotonated

complex **8** (**8NH),** adding a cesium cation to make a neutral system. In model 2 we also added a [Cs^+,HCO_3^-] ion pair, complemented with two explicit DMA molecules in model 3 to solvate the cesium cations, and four explicit DMA molecules in model 4. 3D structures of all the transition states and their relative DLPNO-CCSD(T) Gibbs energies are depicted in Figure S90. The C-H bond cleavage vs. C-N coupling competition is notably influenced by the model chosen. The transition state for the C-N coupling moves from being 5.2 kcal mol^{-1} lower than the C-H cleavage with model 1 to 2.0 kcal mol^{-1} above it with model 4. Indeed, model 4, consisting of complex **8**, a carbonate anion and two cesium cations and four DMA molecules is the simplest one able to reproduce the experimental results and it has been adopted in most of the calculations collected in the paper.

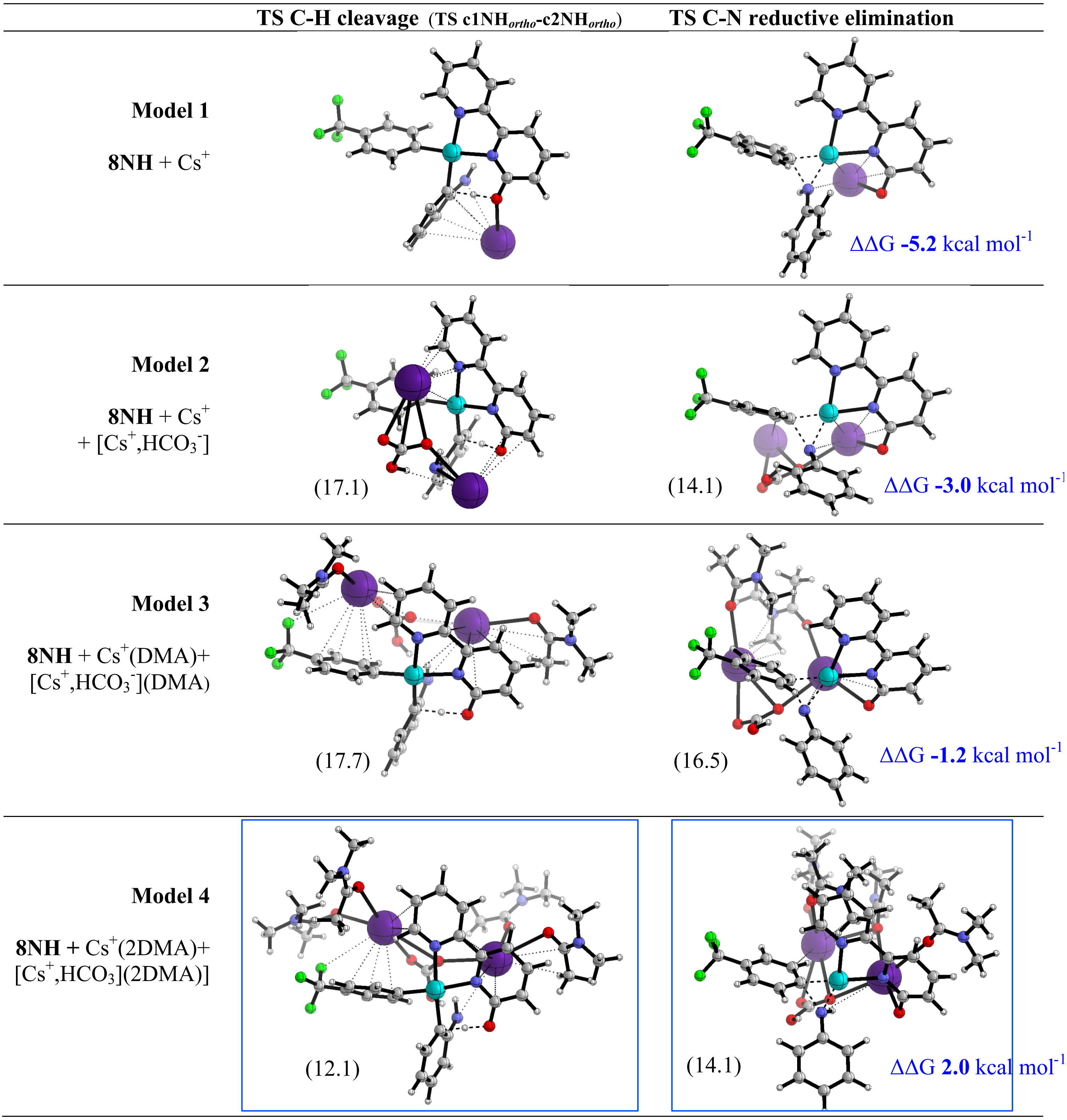

Figure S90. Optimized structures for the transition states of the competing C-H bond cleavage (**TS c1NH**$_{ortho}$-**c2NH**$_{ortho}$) and the C-N reductive elimination in an amido palladium complex **8NH** using different chemical models. Relative Gibbs energies of both transition states (ΔΔG) are also shown in blue (kcal mol^{-1}). A negative value implies a lower TS for C-N reductive elimination than for the *ortho* C-H cleavage. Only model 4 agrees with the experimental evidences. In parenthesis Gibbs activation barriers (kcal mol^{-1}, taking **c1**$_{ortho}$ as reference).

4.3- Gibbs energy profiles.

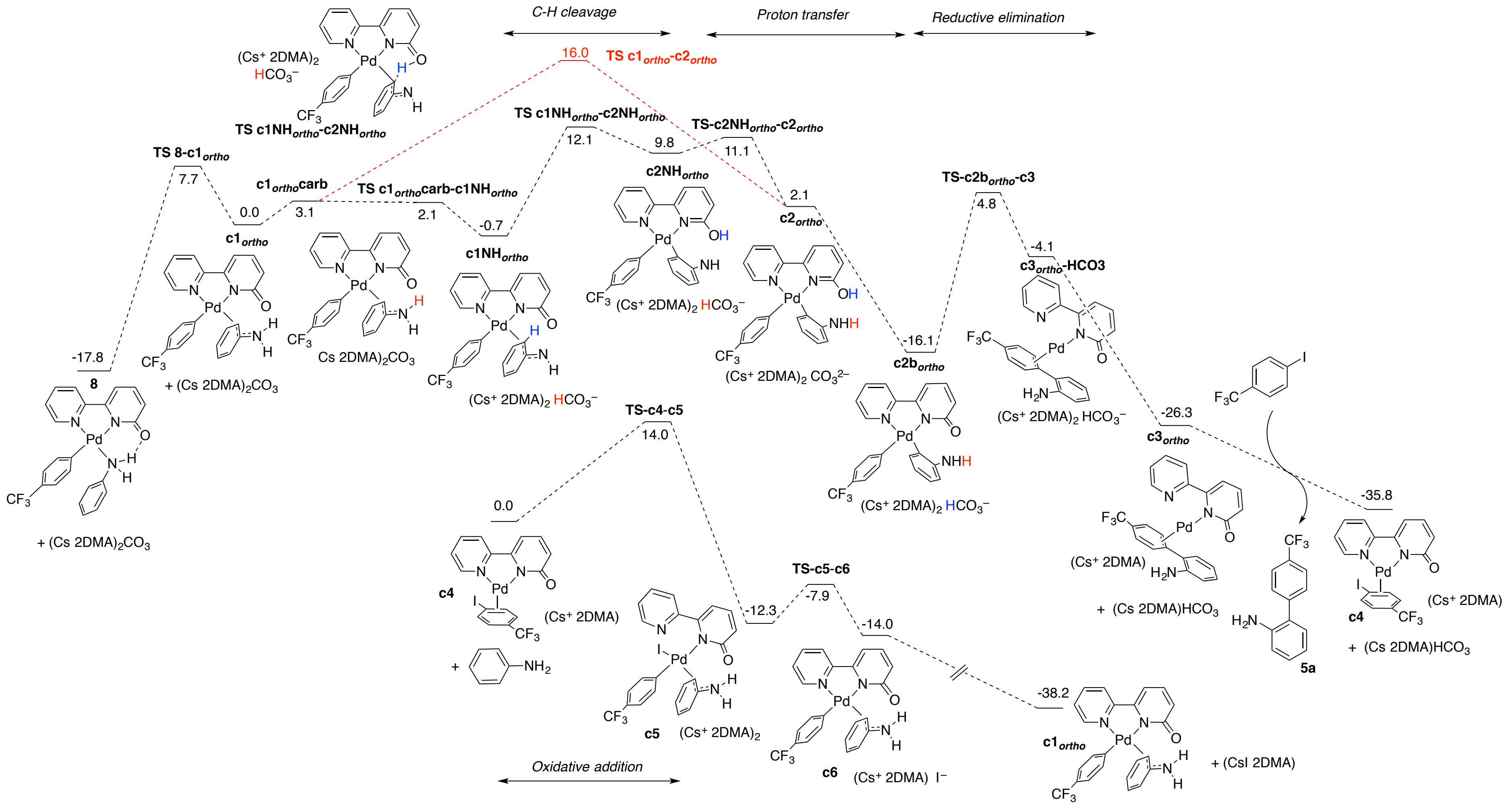

Figure S91. Detailed Gibbs energy profile for the *ortho* arylation of aniline. Energies in kcal mol^{-1}.

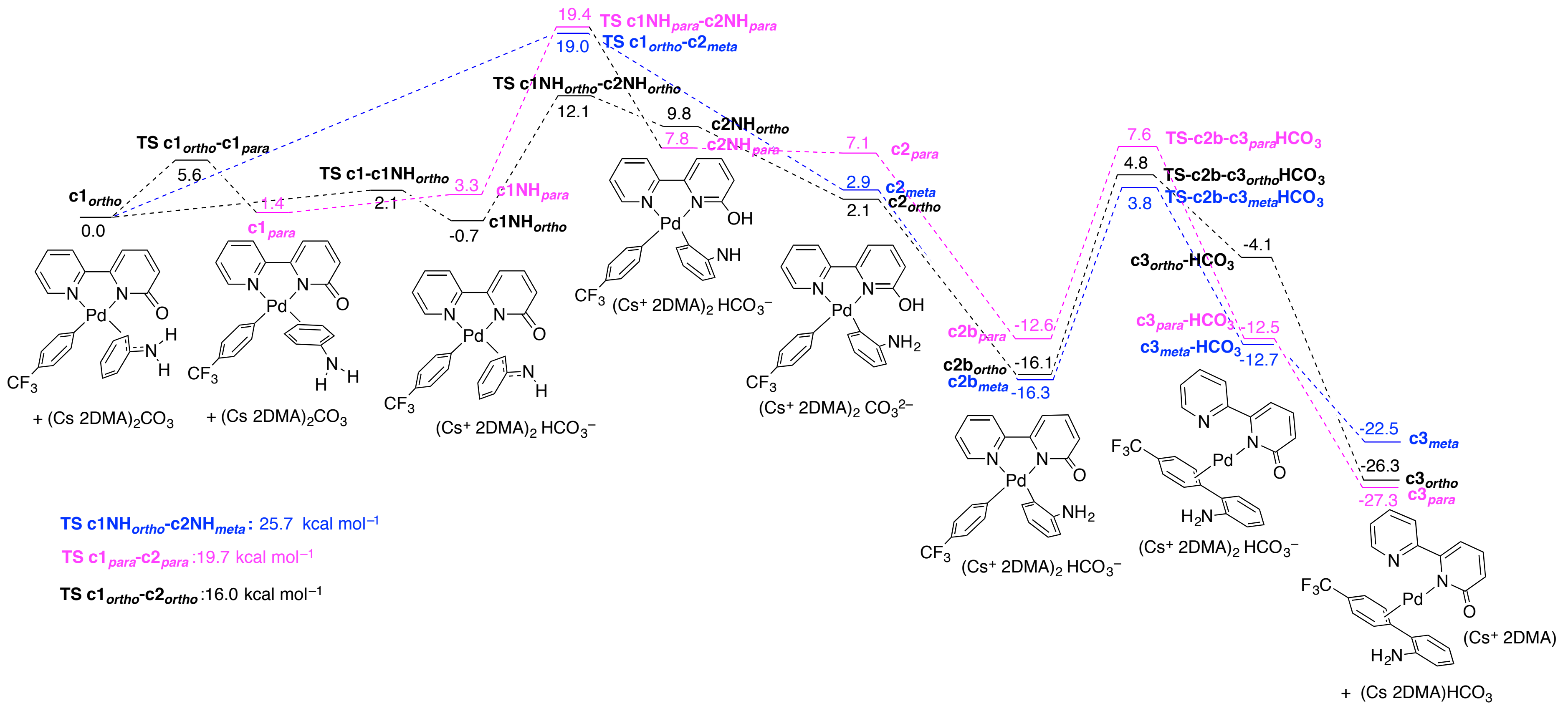

Figure S92. Gibbs energy profiles for the preferred C–H activation pathway (amido-type or anilino-type intermediates) and reductive elimination steps for aniline in the *ortho* (black), the meta (blue) and the para (magenta) positions. For simplicity, only the *ortho* isomer has been drawn for all the intermediates. Energies in kcal mol^{-1}. The energies of the C-H cleavage transition states not depicted in the profile are also shown. No minimum has been found for a **c1**$_{meta}$ intermediate.

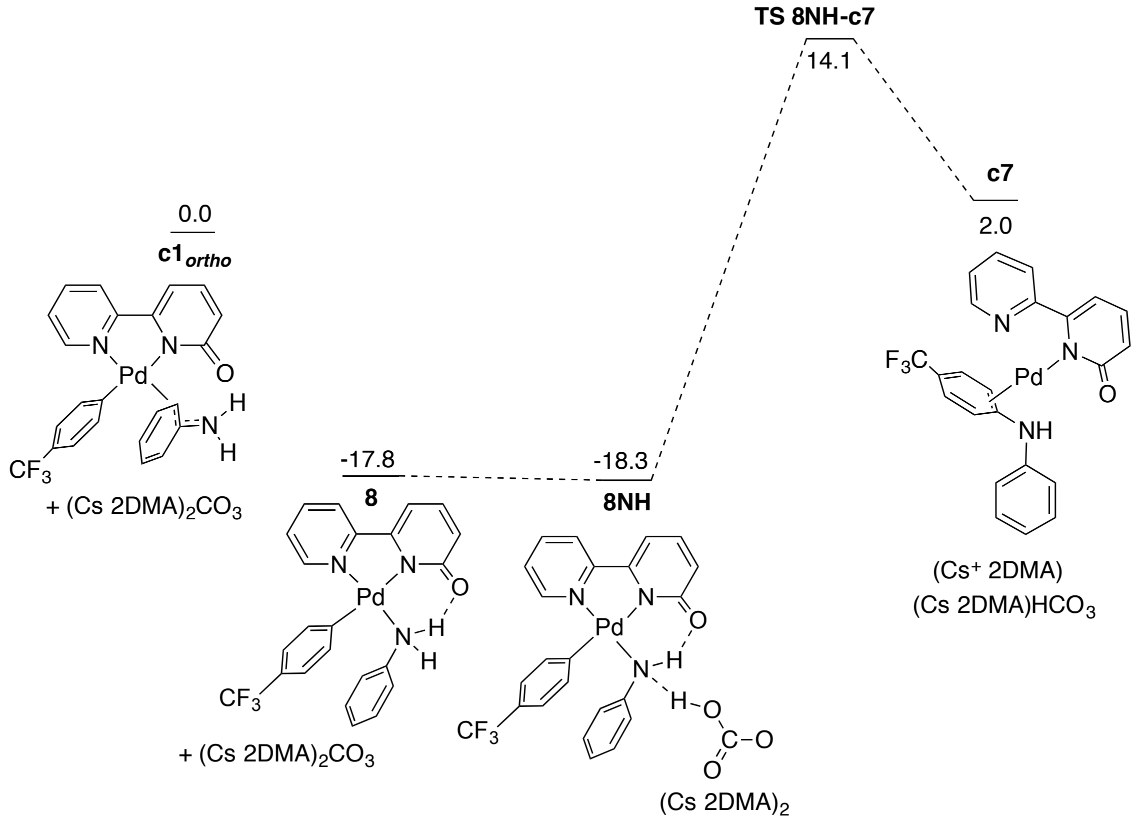

Figure S93. Gibbs energy profile for the formation of the Buchwald-Hartwig amination product (C-N coupling) from complex **8**. Energies (referenced to **c1***ortho*, also shown for clarity) in kcal mol^{-1}.

4.4- Microkinetic modeling.

A simple kinetic simulation of the reaction profile in Figure 1 (main text) corresponding to the reaction in Eq. S2 was carried out using the COPASI software.[37]

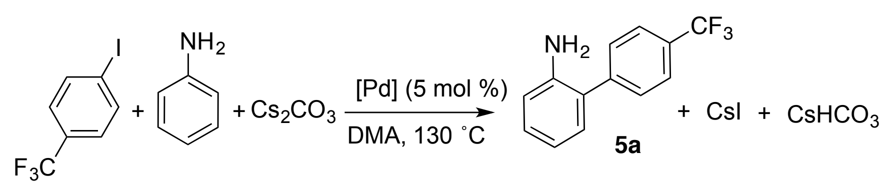

(S2)

The kinetic model is given below (for names of the intermediates, see Figure 1 and Scheme 3 in the main text). Experimental concentrations were used. Equilibrium and rate constants for the kinetic model were calculated from the calculated energy

differences between intermediates (ΔG) or intermediates and transition states ($\Delta G^{\ddagger}$) according to the following equations:

Equilibrium constants: $K = e^{-\Delta G/RT}$ Rate constants: $k = (k_B T/h)e^{-\Delta G\ddagger/RT}$

This model reproduces the experimental conversions collected (Figure S94).

Kinetic model and rate constants.

a) Equilibrium **8-c1*ortho***:

8 ⇌ **c1*ortho***

$k_1 = 0.13\ s^{-1}$, $k_{-1} = 5.7x10^{8}\ s^{-1}$ $K_1 = 2.3x10^{-10}$, $\Delta G = 17.7$ kcal mol^{-1}

b) Deprotonation:

c1*ortho* + Cs_2CO_3 ⇌ **c1NH*ortho*** + $CsHCO_3$

$k_2 = 6.1x10^{11}\ M^{-1}\ s^{-1}$, $k_{-2} = 2.5x10^{11}\ M^{-1}\ s^{-1}$; $K_2 = 2.4$, $\Delta G = -0.7$ kcal mol^{-1}

c) C-H cleavage:

c1NH*ortho* ⇌ **c2NH*ortho***

$k_3 = 9.7x10^{5}\ s^{-1}$, $k_{-3} = 4.8x10^{11}\ s^{-1}$; $K_3 = 2x10^{-6}$, $\Delta G = 10.5$ kcal mol^{-1}

d) Fast proton transfer reactions:

c2NH*ortho* + $CsHCO_3$ ⇌ **c2*ortho*** + Cs_2CO_3

$k_4 = 8.4x10^{12}\ M^{-1}\ s^{-1}$, $k_{-4} = 5.6x10^{8}\ M^{-1}\ s^{-1}$ $K_4 = 1.5x10^{4}$, $\Delta G = -7.7$ kcal mol^{-1}

c2*ortho* + Cs_2CO_3 ⇌ **c2b*ortho*** + $CsHCO_3$

$k_5 = 8.4x10^{12}\ M^{-1}\ s^{-1}$, $k_{-5} = 1.1x10^{3}\ M^{-1}\ s^{-1}$ $K_5 = 7.6x10^{9}$, $\Delta G = -18.2$ kcal mol^{-1}

e) Reductive elimination:

c2b*ortho* ⇌ **c3*ortho***

$k_6 = 40\ s^{-1}$, $k_{-6} = 1.2x10^{-4}\ s^{-1}$; $K_6 = 3.3x10^{5}$, $\Delta G = -10.2$ kcal mol^{-1}

f) Arylhalide coordination:

c3*ortho* + ArI ⇌ **c4** + ArAnil

$k_7 = 8.4x10^{12}\ M^{-1}\ s^{-1}$, $k_{-7} = 6x10^{7}\ M^{-1}\ s^{-1}$; $K_7 = 1.4x10^{5}$, $\Delta G = -9.5$ kcal mol^{-1}

g) Oxidative addition:

c4 + Anil → **c1*ortho*** + CsI $k_8 = 2.2x10^{5}\ M^{-1}\ s^{-1}$

Initial concentrations:

$[\mathbf{8}]_0 = 0.006$ M

$[ArI]_0 = 0.12$ M

$[Anil]_0 = 1.2$ M

$[Cs_2CO_3]_0 = 0.24$ M

$[\mathbf{c1}_{\mathbf{otho}}]_0 = [\mathbf{c1NH}_{\mathbf{otho}}]_0 = [\mathbf{c2NH}_{\mathbf{otho}}]_0 = [\mathbf{c2}_{\mathbf{otho}}]_0 = [\mathbf{c2}_{\mathbf{otho}}\mathbf{b}]_0 = [\mathbf{c3}_{\mathbf{otho}}]_0 = [\mathbf{c4}]_0 = 0$

Figure S94 shows the evolution of the concentration of the product vs. time and reproduces the conversions observed experimentally (see Table S5). As expected from the very small equilibrium constant (**8-c1***ortho*), complex **8** is the most abundant species throughout the reaction and only a small fraction of palladium is in the actual catalytic cycle (almost 0 in the graph in Figure S94).

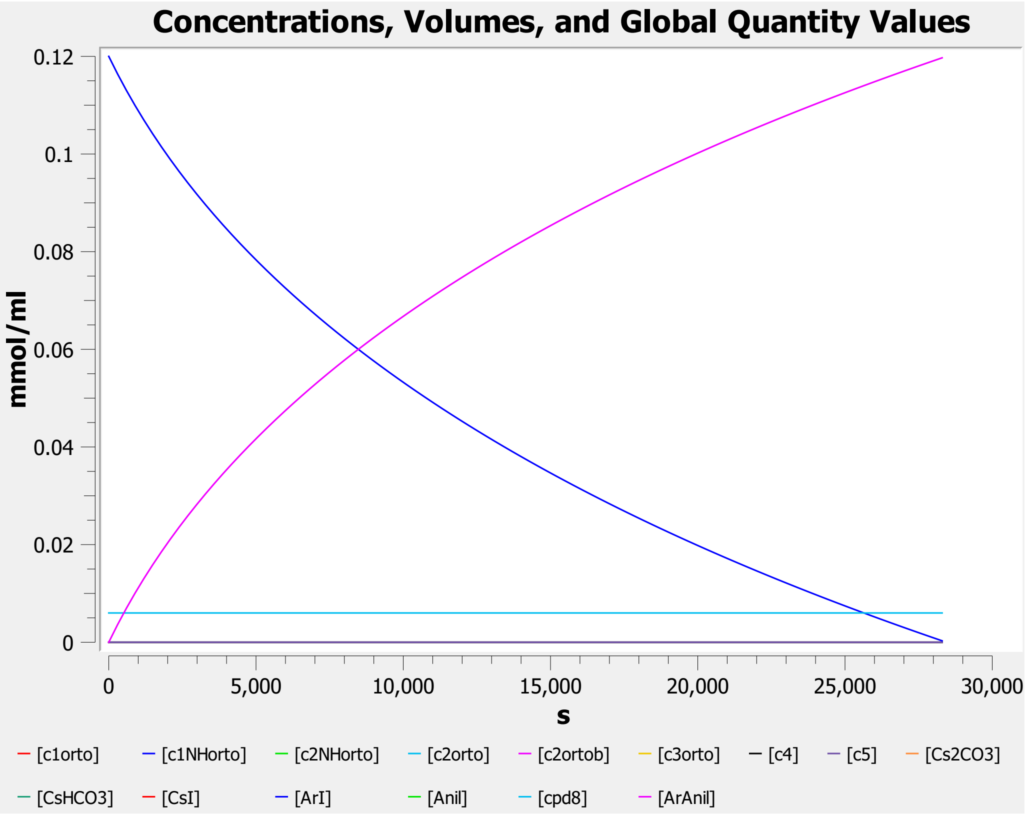

Figure S94. Evolution of concentration (M) over time (s) for the reaction in Eq. S3 Experimental product formation: 0.072 M of product after 3 h (10800 s). The concentrations of aniline and carbonates have been omitted for clarity.

4.5- Probing the intermediacy of Pd(IV) species.

The most stable plausible Pd(IV) intermediate that could be formed in the reaction was calculated. It is [Pd(bipy-6-O)(*o*-$NH_2C_6H_4$)(*p*-$CF_3C_6H_4$)I($PhNH_2$)] with a deprotonated ligand and a coordinated aniline, i.e. the combination of the most donor ligands. The Gibbs energy barrier for the oxidative addition of *p*-$CF_3C_6H_4I$ on the preceding Pd(II) complex is 46.7 kcal mol^{-1} (Figure S95) much higher that those found for the Pd(0)/Pd(II) mechanism in Figure S91. This makes a route where the C-H activation occurs first followed by an oxidative addition of the aryl halide to give a Pd(IV) species less probable in the arylation of anilines.

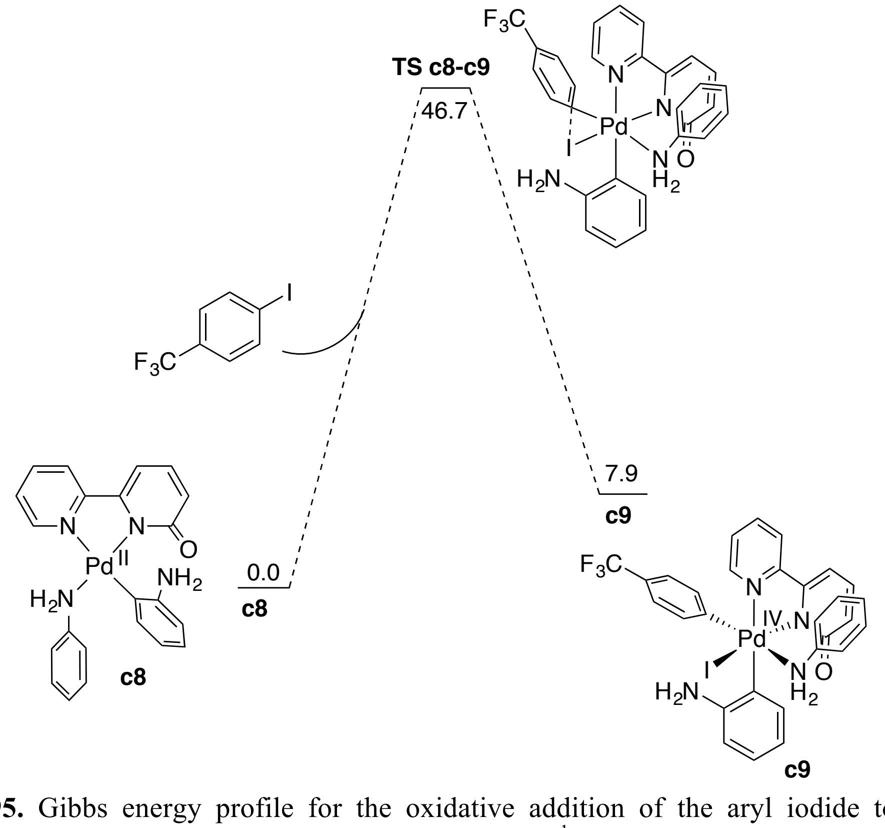

Figure S95. Gibbs energy profile for the oxidative addition of the aryl iodide to a Pd(II) complex to give a Pd(IV) derivative. Energies in kcal mol^{-1}.

4.6- 3D-view of selected structures in the energy profiles.

For structure names, see profiles in Figures S91 and S93.

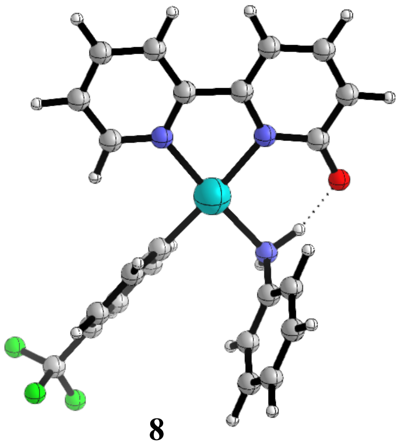

8

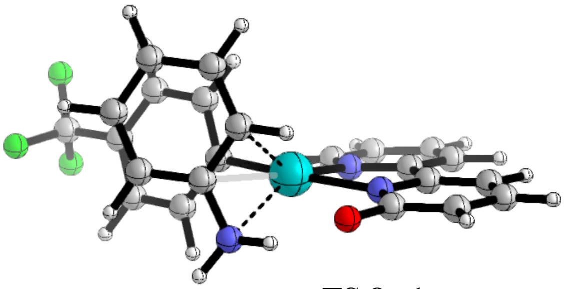

TS 8-c1*ortho*

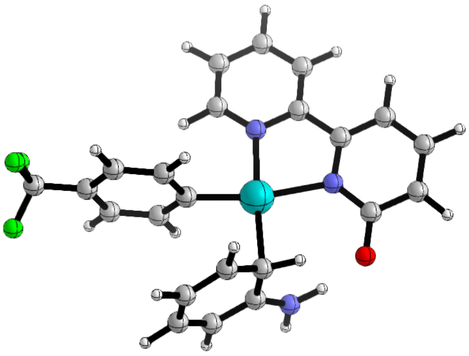

c1*ortho*

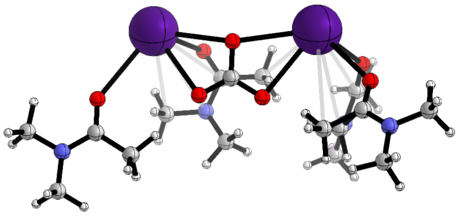

(Cs 2DMA)$_2$CO$_3$

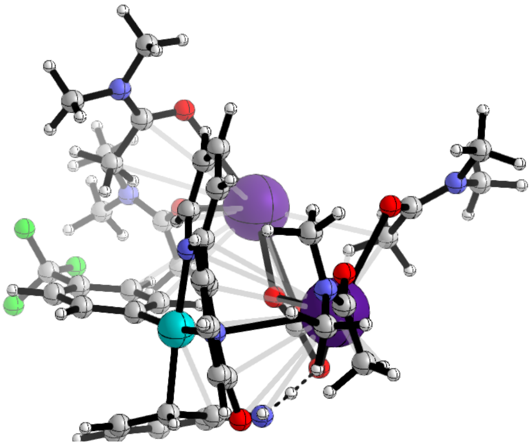

TS c1*ortho*- c1NH*ortho*

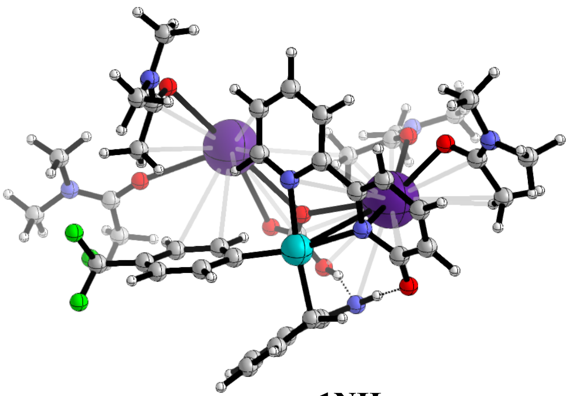

c1NH*ortho*

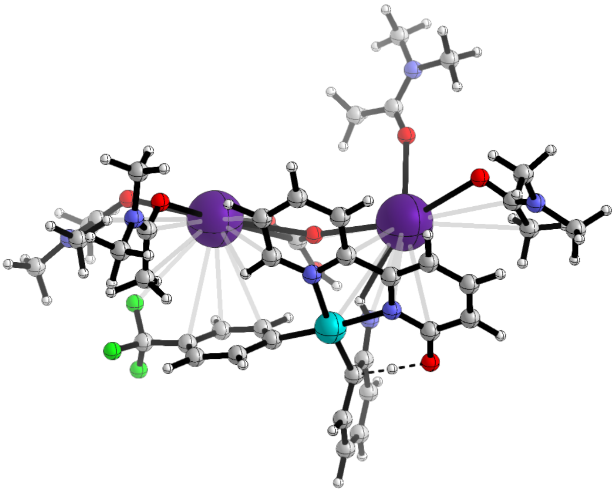

TS c1NH$_{ortho}$- c2NH$_{ortho}$

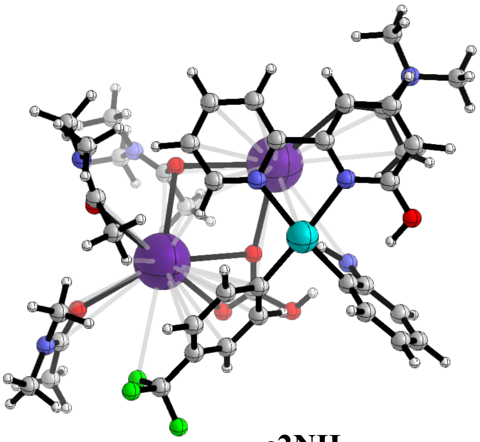

c2NH$_{ortho}$

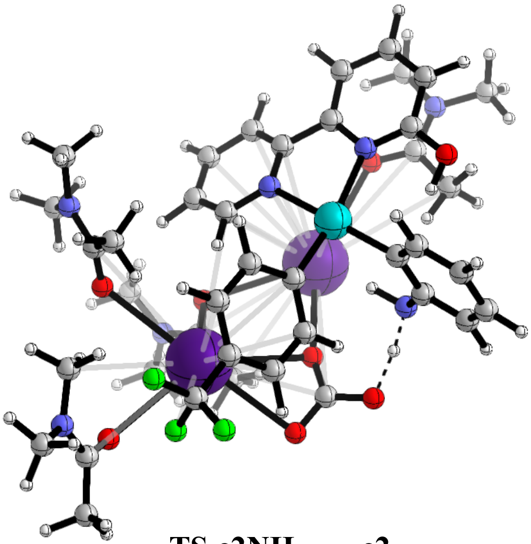

TS c2NH$_{ortho}$- c2$_{ortho}$

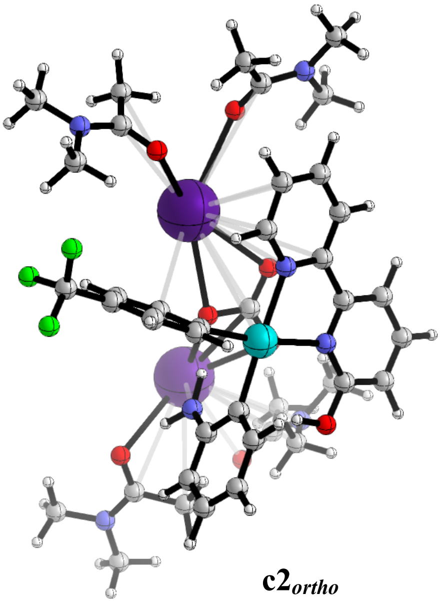

c2$_{ortho}$

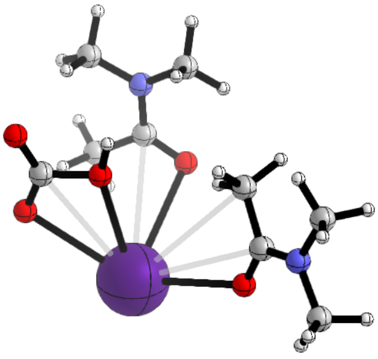

(Cs 2DMA)HCO$_3$

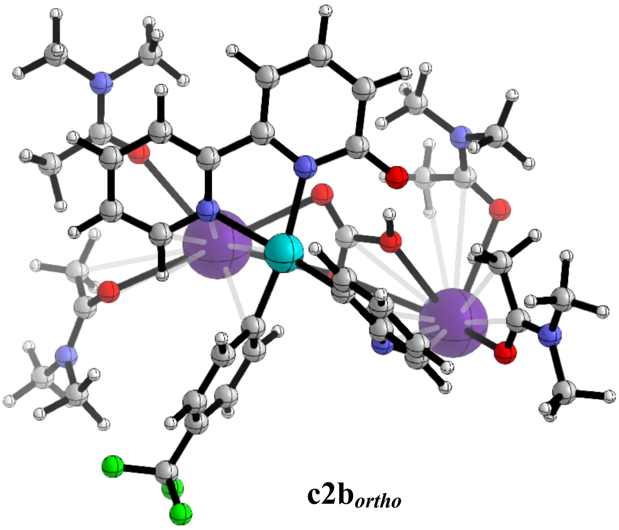

c2b$_{ortho}$

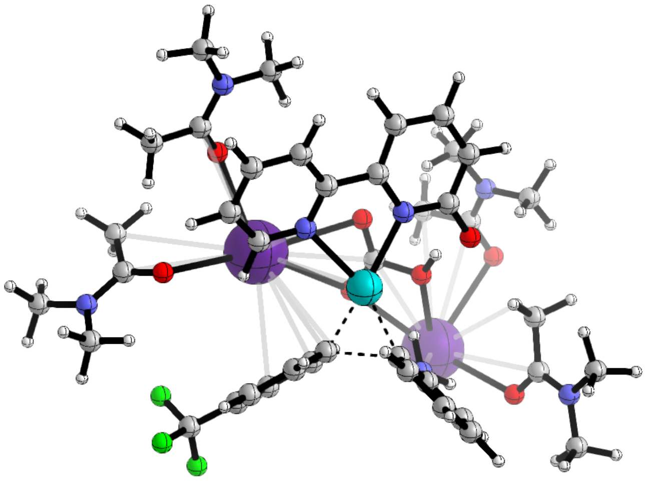

TS c2b$_{ortho}$- c3$_{ortho}$

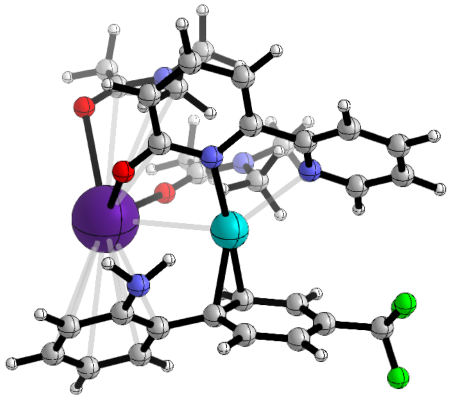

c3$_{ortho}$

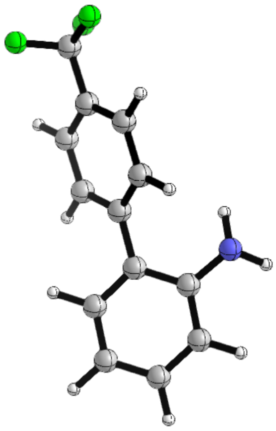

5a

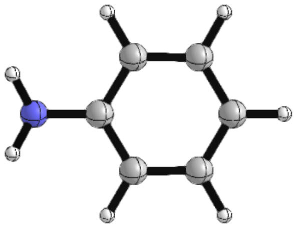

Aniline

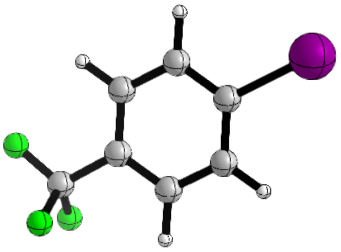

ArI

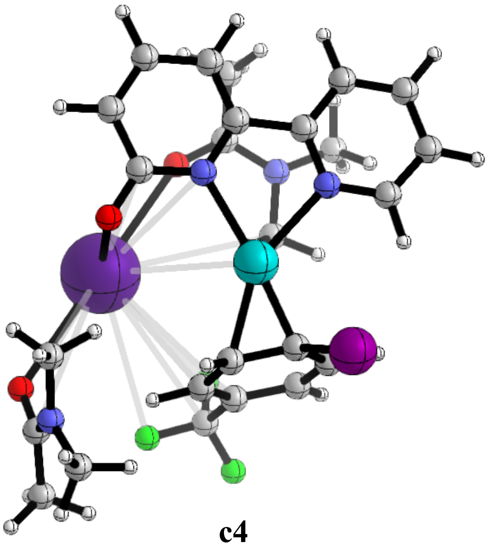

c4

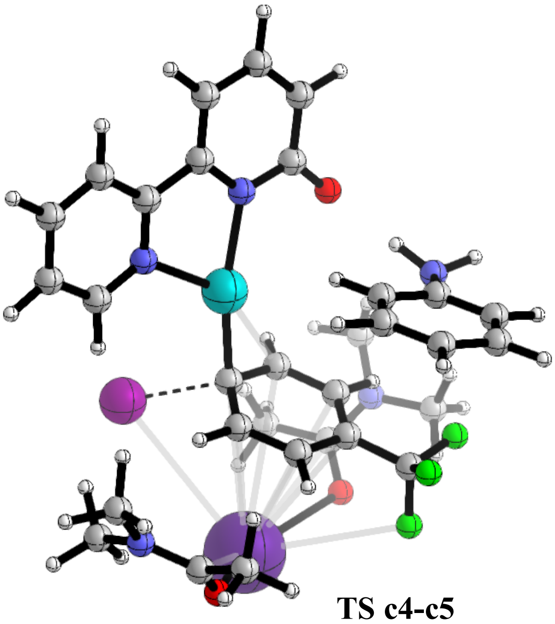

TS c4-c5

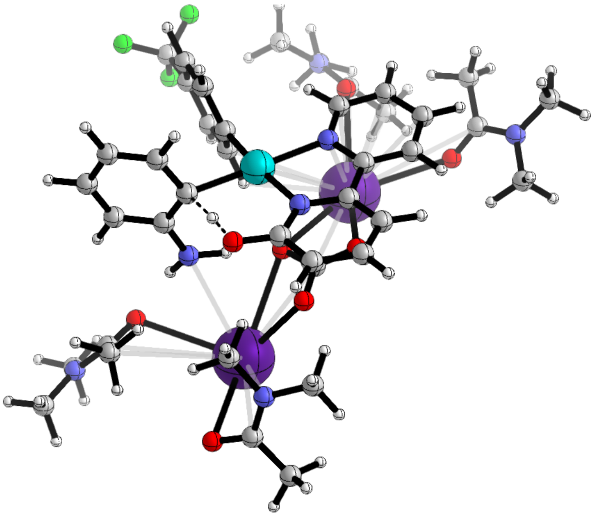

TS c1$_{ortho}$- c2$_{ortho}$

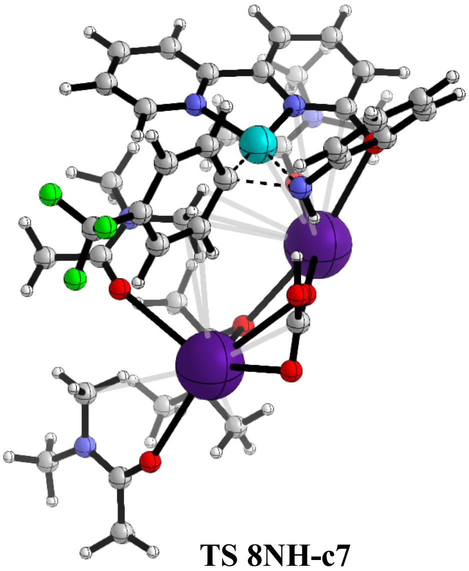

TS 8NH-c7

4.7- Absolute energies of all the calculated species (atomic units).

Table S8. Absolute energies of the calculated species for the arylation reaction of aniline with the simplest model (model 1). Gibbs energy profile in Figure S89.

	$E_{el/gas}^{M06/BS1}$	$E_{el/solv}^{M06/BS1}$	ΔG_{solv}	$T, S\ correction$	$E_{el/gas}^{DLPNO/BS2}$	G_{solv}
**Complex 8**	-1552,200436	-1552,231262	-0,030826	0,271684	-1551,555113	-1551,314255
**TS 8-c1$_{ortho}$**	-1552,165190	-1552,195222	-0,030032	0,273469	-1551,517025	-1551,273588
**c1$_{ortho}$**	-1552,178030	-1552,207005	-0,028975	0,268314	-1551,525243	-1551,285904
**TS c1$_{ortho}$-c1$_{para}$**	-1552,158733	-1552,194123	-0,035389	0,269614	-1551,511135	-1551,276910
**c1$_{para}$**	-1552,169422	-1552,204515	-0,035093	0,271589	-1551,520096	-1551,283601
**TS c1$_{ortho}$-c2$_{ortho}$**	-1552,151963	-1552,183855	-0,031892	0,267513	-1551,504049	-1551,268428
**TS c1$_{para}$-c2$_{para}$**	-1552,146394	-1552,180921	-0,034527	0,265869	-1551,498858	-1551,267516
**TS c1$_{meta}$-c2$_{meta}$**	-1552,139419	-1552,173649	-0,034230	0,265625	-1551,494368	-1551,262973
**c2$_{ortho}$**	-1552,170545	-1552,205259	-0,034714	0,269295	-1551,527837	-1551,293256
**c2$_{meta}$**	-1552,166547	-1552,202326	-0,035779	0,268034	-1551,523473	-1551,291218
**c2$_{para}$**	-1552,165474	-1552,200969	-0,035495	0,268061	-1551,522171	-1551,289604
**TS RE$_{ortho}$**	-1552,155248	-1552,187243	-0,031994	0,271725	-1551,496255	-1551,256525
**TS RE$_{meta}$**	-1552,153005	-1552,186616	-0,033611	0,269983	-1551,495267	-1551,258895
**TS RE$_{para}$**	-1552,152095	-1552,185972	-0,033878	0,268254	-1551,495104	-1551,260728
**c3$_{ortho}$**	-1552,202738	-1552,231738	-0,029000	0,274288	-1551,523653	-1551,278364
**c3$_{meta}$**	-1552,198669	-1552,230725	-0,032056	0,272091	-1551,518879	-1551,278844
**c3$_{para}$**	-1552,196585	-1552,229465	-0,033878	0,271347	-1551,515859	-1551,278389

Table S9. Absolute energies of the calculated species for the arylation reaction of aniline with the model including cesium and carbonate ions and four DMA solvent molecules (model 4). Gibbs energy profiles in Figure 1 (main text) and Figures S91 and S92.

	$E_{el/gas}^{M06/BS1}$	$E_{el/solv}^{M06/BS1}$	ΔG_{solv}	$T,S\ correction$	$E_{el/gas}^{DLPNO/BS2}$	G_{solv}
**Complex 8**	-1552,200436	-1552,231262	-0,030826	0,271684	-1551,555113	-1551,314255
**(Cs 2DMA)$_2$CO$_3$**	-1454,048505	-1454,109324	-0,060819	0,406632	-1452,933631	-1452,587817
	***ortho*-arylation**					
**TS 8-c1$_{ortho}$**	-1552,165190	-1552,195222	-0,030032	0,273469	-1551,517025	-1551,273588
**c1$_{ortho}$**	-1552,178030	-1552,207005	-0,028975	0,268314	-1551,525243	-1551,285904
**c1$_{ortho}$ + (Cs 2DMA)$_2$CO$_3$**	-3006,226535	-3006,316329	-0,089794	0,674946	-3004,458874	-3003,873722
**c1$_{ortho}$carb**	-3006,266936	-3006,356297	-0,089361	0,716283	-3004,491572	-3003,864650
**TS c1$_{ortho}$carb-c1NH$_{ortho}$**	-3006,260836	-3006,353727	-0,092891	0,711677	-3004,485152	-3003,866366
**c1NH$_{ortho}$**	-3006,275460	-3006,367942	-0,092482	0,714267	-3004,492502	-3003,870717
**TS c1NH$_{ortho}$-c2NH$_{ortho}$**	-3006,216659	-3006,328898	-0,112239	0,700029	-3004,438156	-3003,850366
**c2NH$_{ortho}$**	-3006,234749	-3006,352997	-0,118248	0,719252	-3004,454958	-3003,853954
**TS c2NH$_{ortho}$-c2$_{ortho}$**	-3006,220633	-3006,342968	-0,122336	0,719709	-3004,449348	-3003,851975
**c2$_{ortho}$**	-3006,248227	-3006,343711	-0,095484	0,712069	-3004,482809	-3003,866224
**c2b$_{ortho}$**	-3006,301235	-3006,394157	-0,092921	0,719943	-3004,522340	-3003,895319
**TS c2b$_{ortho}$-c3**	-3006,293730	-3006,380249	-0,086519	0,725281	-3004,500692	-3003,861930
**c3$_{ortho}$-HCO$_3$**	-3006,342503	-3006,424446	-0,081943	0,727538	-3004,521743	-3003,876148
**c3$_{ortho}$**	-2146,820487	-2146,873172	-0,052685	0,482563	-2145,691100	-2145,261222
**(Cs 2DMA)HCO$_3$**	-859,475613	-859,521978	-0,046365	0,197750	-858,801756	-858,650372
**c3$_{ortho}$ + (Cs 2DMA)HCO$_3$**	-3006,296100	-3006,395150	-0,099050	0,680313	-3004,492856	-3003,911594
**c4**	-1871,386000	-1871,434271	-0,048270	0,384248	-2156,360022	-2156,024044
**5a**	-855,180914	-855,196337	-0,015423	0,139465	-854,352524	-854,228483
**c4 + 5a + (Cs 2DMA)HCO$_3$**	-3586,042527	-3586,152586	-0,110059	0,721463	-3869,514302	-3868,902898
**TS c1$_{ortho}$-c2$_{ortho}$**	-3006,22958	-3006,324934	-0,095355	0,710714	-3004,459537	-3003,844178
	***para*-arylation**					
**TS c1$_{ortho}$-c1$_{para}$**	-1552,158733	-1552,194123	-0,035389	0,269614	-1551,511135	-1551,276910
**c1$_{para}$**	-1552,169422	-1552,204515	-0,035093	0,271589	-1551,520096	-1551,283601
**c1NH$_{para}$**	-3006,248344	-3006,358828	-0,110483	0,713263	-3004,467108	-3003,864328
**TS c1NH$_{para}$-c2NH$_{para}$**	-3006,217920	-3006,329528	-0,111609	0,71183	-3004,438887	-3003,838671
**c2NH$_{para}$**	-3006,226741	-3006,332553	-0,105812	0,70481	-3004,456246	-3003,857252

**c2$_{para}$**	-3006,245264	-3006,340387	-0,095123	0,71513	-3004,478272	-3003,858267
**c2b$_{para}$**	-3006,299383	-3006,391990	-0,092607	0,7212	-3004,518385	-3003,889794
**TS c2b-c3$_{para}$HCO$_3$**	-3006,275542	-3006,374886	-0,099344	0,72568	-3004,483811	-3003,857479
**c3$_{para}$-HCO$_3$**	-3006,317940	-3006,422864	-0,104925	0,72319	-3004,507849	-3003,889588
**c3$_{para}$**	-3006,275065	-3006,389300	-0,114235	0,6754	-3004,474299	-3003,913133
**TS c1$_{para}$-c2$_{para}$**	-3006,254902	-3006,332627	-0,077725	0,71513	-3004,475648	-3003,838248
	***meta*-arylation**					
**TS c1$_{ortho}$-c2$_{meta}$**	-3006,217362	-3006,321557	-0,104195	0,715504	-3004,450750	-3003,839440
**c2$_{meta}$**	-3006,236008	-3006,348143	-0,112136	0,718942	-3004,471760	-3003,864954
**c2b$_{meta}$**	-3006,267522	-3006,384124	-0,116602	0,719227	-3004,498266	-3003,895641
**TS c2b-c3$_{meta}$HCO$_3$**	-3006,255839	-3006,368037	-0,112198	0,719773	-3004,471204	-3003,863630
**c3$_{meta}$-HCO3**	-3006,323663	-3006,424780	-0,101117	0,725726	-3004,514421	-3003,889812
**c3$_{meta}$**	-3006,272584	-3006,387657	-0,115074	0,679582	-3004,470070	-3003,905562
**TS c1NH$_{meta}$-c2NH$_{meta}$**	-3006,206335	-3006,318972	-0,112637	0,711320	-3004,427403	-3003,828721

Table S10. Absolute energies of the calculated species for the oxidative addition. Gibbs energy profile in Figure 1 (bottom) and Figure S91 (bottom).

	$E_{el/gas}^{M06/BS1}$	$E_{el/solv}^{M06/BS1}$	ΔG_{solv}	$T,S\ correction$	$E_{el/gas}^{DLPNO/BS2}$	G_{solv}
**c4**	-1871,386000	-1871,434271	-0,048270	0,384248	-2156,360022	-2156,024044
**aniline**	-287,370789	-287,383680	-0,012891	0,074301	-287,062514	-287,001104
**c4 + aniline**	-2158,756789	-2158,817950	-0,061161	0,458549	-2443,422536	-2443,025148
**TS c4-c5**	-2158,744359	-2158,817258	-0,072899	0,483822	-2443,409618	-2442,998695
**c5**	-2158,792654	-2158,863127	-0,070473	0,490047	-2443,460307	-2443,040733
**TS c5-c6**	-2158,774033	-2158,856926	-0,082893	0,489854	-2443,440603	-2443,033642
**c6**	-2158,766756	-2158,860494	-0,093738	0,486521	-2443,436145	-2443,043361
**(CsI 2DMA)**	-606,574145	-606,636680	-0,062534	0,168433	-891,905983	-891,800084
**c1$_{ortho}$**	-1552,178030	-1552,207005	-0,028975	0,268314	-1551,525243	-1551,285904
**c1$_{ortho}$ + (CsI 2DMA)**	-2158,752175	-2158,843685	-0,091510	0,436747	-2443,431226	-2443,085989

Table S11. Absolute energies of the calculated species for the Buchwald-Hartwig amination (C-N coupling). Gibbs energy profile in Figure S93.

	$E_{el/gas}^{M06/BS1}$	$E_{el/solv}^{M06/BS1}$	ΔG_{solv}	$T, S\ correction$	$E_{el/gas}^{DLPNO/BS2}$	G_{solv}
**8NH**	-3006,286862	-3006,384403	-0,097540	0,708343	-3004,509738	-3003,898935
**TS 8NH-c7**	-3006,244700	-3006,353832	-0,109132	0,720247	-3004,458344	-3003,847229
**c7**	-3006,299370	-3006,407542	-0,108172	0,722302	-3004,480579	-3003,866449

Table S12. Absolute energies of the calculated species for the oxidative addition of the aryl iodide to a Pd(II) complex to give a Pd(IV) derivative. Gibbs energy profile in Figure S95

	$E_{el/gas}^{M06/BS1}$	$E_{el/solv}^{M06/BS1}$	ΔG_{solv}	$T, S\ correction$	$E_{el/gas}^{DLPNO/BS2}$	G_{solv}
**c8**	-1270,554107	-1270,604833	-0,050726	0,293791	-1270,118545	-1269,875480
**TS c8-c9**	-1850,259884	-1850,306978	-0,047094	0,357367	-2135,083461	-2134,773188
**c9**	-1850,306161	-1850,352260	-0,046099	0,364207	-2135,153025	-2134,834918

4.7- Cartesian coordinates and electronic energies (M06/BS1/SMD) of all the calculated species.

Arylation reaction of aniline with the model including cesium and carbonate ions and four DMA solvent molecules (model 4). Gibbs energy profiles in Figure 1 (main text) and S91 and S92.

\# **Complex 8**

| Charge 0
| Electronic Energy (a.u.) 1552.2312622

C	-5.060845	1.500983	-0.680123
H	-5.556059	2.422930	-0.979472
C	-5.761788	0.417637	-0.229049
H	-6.848413	0.456230	-0.158105
C	-3.626260	1.464976	-0.783823
C	-5.084561	-0.757566	0.144306
H	-5.642820	-1.618429	0.498440
C	-3.706833	-0.770542	0.043677
C	-2.908220	-1.959634	0.406083
C	-3.481562	-3.151383	0.849737
H	-4.559671	-3.238469	0.944339
C	-2.674625	-4.231542	1.169509
H	-3.121705	-5.160847	1.515665
C	-1.296492	-4.110913	1.040088
H	-0.624091	-4.930486	1.278206
C	-0.781023	-2.906190	0.594351
H	0.290261	-2.760977	0.476775
C	1.081961	-0.436255	-0.327151
C	1.825688	-0.620279	-1.501268
C	3.199081	-0.832745	-1.448939
C	3.852286	-0.857962	-0.215836
C	3.129551	-0.684688	0.962086
C	1.753345	-0.479315	0.901158
N	-2.997344	0.301693	-0.397052
N	-1.559567	-1.857551	0.286400
O	-2.974157	2.450940	-1.209448
Pd	-0.873584	-0.016471	-0.390532
C	5.320563	-1.113900	-0.202870
F	5.869308	-0.954446	1.007167
F	5.612454	-2.366353	-0.600818
F	5.979372	-0.298344	-1.044452
H	1.200640	-0.337057	1.832406
H	3.635918	-0.707019	1.925511
H	3.769953	-0.974446	-2.367286
H	1.334838	-0.582895	-2.475462
H	-1.255603	2.261393	-1.272438
N	-0.299842	1.892161	-1.069631
H	0.244036	1.809218	-1.930340
C	0.379333	2.685537	-0.095309
C	-0.351556	3.230827	0.960914

C	1.756602	2.885801	-0.173325
C	0.300937	3.973785	1.938011
H	-1.429074	3.067543	1.000849
C	2.401342	3.627297	0.812131
H	2.319130	2.446622	-0.998278
C	1.679094	4.173241	1.870087
H	-0.273059	4.401007	2.758998
H	3.477896	3.778521	0.749039
H	2.186905	4.752446	2.639434

***

(Cs 2DMA)$_2$CO$_3$

| Charge 0
| Electronic Energy (a.u.) -1454.10932354

Cs	-3.231987	-0.871574	-1.882012
O	-1.539543	1.007534	-0.212062
C	-0.407088	0.552502	-0.611957
O	0.652281	0.670814	0.147068
O	-0.299745	-0.045348	-1.751202
Cs	2.658885	-0.670843	-1.757437
O	4.623589	1.566333	-0.783343
C	4.387618	2.642085	-0.226238
C	3.007973	2.976368	0.278919
H	2.607189	3.864400	-0.229343
H	2.320913	2.133413	0.103539
H	3.021214	3.201617	1.354193
N	5.363297	3.578445	-0.049159
C	6.724331	3.294612	-0.451771
H	7.393667	3.312552	0.421184
H	6.763920	2.307818	-0.916826
H	7.076891	4.050493	-1.167824
C	5.190541	4.843356	0.632873
H	4.136212	5.103340	0.744299
H	5.655328	4.820581	1.630830
H	5.676462	5.639697	0.052188
O	3.981435	-2.374051	0.522338
C	3.639331	-2.557031	1.693455
C	3.670586	-3.939026	2.297992
H	2.657833	-4.306393	2.511921
H	4.136090	-4.613663	1.573813
H	4.240622	-3.972237	3.234682
N	3.227990	-1.533082	2.485347
C	3.205164	-0.177944	1.956417
H	3.971599	-0.064356	1.182111
H	2.223293	0.079313	1.518102
H	3.429455	0.519282	2.775043
C	2.734003	-1.675135	3.838375
H	2.553025	-2.720803	4.097080
H	3.443566	-1.250670	4.564865
H	1.782579	-1.133035	3.940864
O	-5.223709	0.870847	-0.140232
C	-5.268751	1.707214	0.765284
C	-4.016517	2.193441	1.448448
H	-4.001135	1.874721	2.501062
H	-3.135695	1.782468	0.930375
H	-3.959404	3.290481	1.446522

N	-6.456524	2.222323	1.199525
C	-7.689847	1.872986	0.527604
H	-8.150390	2.766800	0.080548
H	-7.476923	1.145925	-0.258415
H	-8.406344	1.439991	1.240032
C	-6.589977	3.237575	2.223112
H	-5.723936	3.255824	2.888262
H	-6.720889	4.239156	1.783399
H	-7.476798	3.017987	2.833028
O	-1.885135	-3.071342	0.080216
C	-1.224254	-2.387051	0.868944
C	0.279421	-2.408810	0.878200
H	0.683800	-2.603767	1.882224
H	0.629201	-3.187596	0.191351
H	0.642262	-1.414274	0.557738
N	-1.809102	-1.566427	1.784108
C	-3.240750	-1.364426	1.734054
H	-3.482578	-0.459487	1.155006
H	-3.714300	-2.232037	1.265100
H	-3.639046	-1.249084	2.750989
C	-1.038112	-0.593384	2.549030
H	-0.382529	-1.097052	3.275515
H	-0.448766	0.055990	1.878844
H	-1.740790	0.024457	3.119899

****

***Ortho*-arylation**

TS 8-c1*ortho*
| Charge 0
| Electronic Energy (a.u.) -1552.19522172

C	5.243924	-1.187067	-0.233928
H	5.868122	-2.068181	-0.371479
C	5.774784	0.034812	0.069548
H	6.851513	0.155641	0.184921
C	3.825205	-1.360703	-0.405518
C	4.930726	1.148492	0.222886
H	5.354141	2.123709	0.439316
C	3.569099	0.956671	0.080730
C	2.621404	2.082752	0.190115
C	3.034895	3.392931	0.438730
H	4.085565	3.612264	0.601276
C	2.109545	4.422077	0.473240
H	2.435817	5.441837	0.664522
C	0.769235	4.132269	0.251081
H	0.005504	4.904972	0.253011
C	0.408610	2.815666	0.028127
H	-0.629915	2.544883	-0.139628
C	-1.102086	0.123824	-0.148148
C	-1.823245	0.124686	-1.346508
C	-3.191136	0.367675	-1.334543
C	-3.842165	0.619542	-0.124594
C	-3.127936	0.632855	1.069652
C	-1.755093	0.390022	1.055419
N	3.023699	-0.256617	-0.198153
N	1.302720	1.813255	0.008094
O	3.335791	-2.470177	-0.725166

Pd	0.851060	-0.212946	-0.191843
C	-5.313601	0.858274	-0.158194
F	-5.824108	1.153506	1.042854
F	-5.638938	1.869955	-0.981973
F	-5.981037	-0.216596	-0.614254
H	-1.198952	0.412860	1.993967
H	-3.635935	0.836387	2.010558
H	-3.758254	0.360092	-2.266039
H	-1.321766	-0.083644	-2.292911
H	1.679159	-2.587588	-1.512503
N	0.660509	-2.471649	-1.593066
H	0.238985	-2.825318	-2.445878
C	-0.041003	-2.727234	-0.419216
C	0.610286	-2.629167	0.831849
C	-1.415180	-3.029476	-0.443686
C	-0.125757	-2.781259	2.012599
H	1.698129	-2.558515	0.854310
C	-2.118649	-3.190882	0.737652
H	-1.916194	-3.123754	-1.407929
C	-1.486349	-3.051307	1.979262
H	0.395849	-2.715012	2.967073
H	-3.181875	-3.426191	0.694492
H	-2.049818	-3.176916	2.901919

***

c1$_{ortho}$
| Charge   0
| Electronic Energy (a.u.)   -1552.20700532

C	5.319388	-0.750645	0.499516
H	6.025901	-1.567853	0.633245
C	5.696095	0.557535	0.609143
H	6.727233	0.820399	0.843926
C	3.962434	-1.111516	0.182802
C	4.747136	1.573213	0.403597
H	5.039101	2.615721	0.479640
C	3.443596	1.203409	0.127709
C	2.410525	2.228471	-0.126369
C	2.723515	3.575725	-0.321165
H	3.757096	3.906552	-0.292157
C	1.717204	4.493441	-0.572303
H	1.961819	5.542140	-0.726432
C	0.401067	4.052924	-0.632624
H	-0.422866	4.732895	-0.831451
C	0.150680	2.705786	-0.439201
H	-0.864113	2.317539	-0.481964
C	-1.144264	-0.034099	0.151265
C	-1.884706	-0.129263	-1.034229
C	-3.247811	0.145094	-1.038402
C	-3.884319	0.529993	0.142595
C	-3.156223	0.647140	1.323898
C	-1.792620	0.364274	1.324549
N	3.038156	-0.093593	0.057131
N	1.122780	1.813702	-0.194197
O	3.636897	-2.314550	0.027126
Pd	0.831409	-0.262701	0.102123
C	-5.343742	0.827627	0.090584
F	-5.846747	1.185320	1.277854

F	-5.619900	1.825811	-0.768900
F	-6.057790	-0.229808	-0.335097
H	-1.235929	0.452694	2.258807
H	-3.649737	0.955497	2.243992
H	-3.819222	0.067885	-1.964357
H	-1.399343	-0.414048	-1.968675
C	-1.819238	-3.501448	-0.308373
C	-0.427269	-2.614465	1.425432
C	0.652164	-2.463133	0.506313
C	0.480945	-2.953893	-0.835404
H	-2.783535	-3.895768	-0.629155
H	1.675939	-2.458079	0.891140
C	-1.646481	-3.115366	1.037314
C	-0.801626	-3.409597	-1.231546
H	-0.945663	-3.747345	-2.257968
H	-2.469057	-3.203246	1.744019
H	-0.257919	-2.317981	2.460917
N	1.528407	-3.006968	-1.675485
H	1.423509	-3.397616	-2.602737
H	2.452654	-2.759866	-1.302925

***

c1$_{ortho}$carb

| Charge 0
| Electronic Energy (a.u.) -3006.3562968

C	4.995647	-3.253612	0.782590
H	5.741093	-3.940468	0.382205
C	5.085050	-2.754598	2.053884
H	5.906576	-3.051838	2.706992
C	3.928473	-2.856249	-0.101971
C	4.115117	-1.846491	2.517060
H	4.175190	-1.415589	3.513416
C	3.063912	-1.548605	1.673141
C	2.025066	-0.573469	2.058502
C	2.290395	0.494113	2.917062
H	3.273663	0.591090	3.371505
C	1.312438	1.454813	3.134547
H	1.515491	2.305459	3.782749
C	0.085737	1.329221	2.491019
H	-0.701821	2.072797	2.603074
C	-0.118154	0.233927	1.665088
H	-1.057802	0.084246	1.138088
C	-1.257203	-2.257909	0.040574
C	-2.082234	-1.407332	-0.703398
C	-3.468105	-1.483521	-0.559683
C	-4.030487	-2.400424	0.326420
C	-3.210693	-3.229760	1.093554
C	-1.829096	-3.151407	0.955638
N	2.922177	-2.072705	0.426487
N	0.823266	-0.691678	1.452128
O	3.906393	-3.201298	-1.308767
Pd	0.723580	-2.170520	-0.068475
C	-5.501183	-2.503533	0.527082
F	-5.861043	-2.210370	1.794384
F	-6.201295	-1.685007	-0.269899
F	-5.956463	-3.749261	0.302646
H	-1.198185	-3.814898	1.549667

H	-3.655080	-3.936517	1.795695
H	-4.108090	-0.800815	-1.121667
H	-1.653035	-0.682262	-1.406702
H	2.701744	-2.208326	-2.455743
N	1.876805	-2.007496	-3.031900
C	0.787403	-2.724227	-2.671828
C	0.852965	-3.761300	-1.682438
C	-0.457836	-2.496543	-3.325519
C	-0.238984	-4.673531	-1.570657
H	1.848442	-4.085629	-1.368288
C	-1.491885	-3.382805	-3.159137
H	-0.555435	-1.606259	-3.948171
C	-1.387401	-4.504561	-2.301342
H	-0.138962	-5.515330	-0.884343
H	-2.424176	-3.214874	-3.700051
H	-2.221984	-5.197014	-2.202776
H	1.663649	-1.005296	-3.275943
O	1.050126	0.556043	-3.432420
O	0.283622	0.793710	-1.332693
C	0.051505	0.703917	-2.608165
O	-1.151621	0.756781	-3.048902
Cs	-2.304393	2.333021	-0.671188
Cs	3.284585	0.908265	-1.251660
O	5.747432	0.686078	0.703887
C	6.638169	-0.157988	0.586531
C	6.963527	-0.766984	-0.755250
H	6.701871	-1.833543	-0.786306
H	6.386804	-0.245986	-1.525756
H	8.029760	-0.678335	-0.999585
N	7.378390	-0.581751	1.647397
C	7.211167	0.037677	2.944289
H	8.132414	0.560885	3.241863
H	6.389743	0.755720	2.895961
H	6.989741	-0.722831	3.706976
C	8.458256	-1.542487	1.553126
H	8.300004	-2.248135	0.732924
H	9.432721	-1.048832	1.414018
H	8.501826	-2.120218	2.485327
O	-3.203169	2.586570	2.422231
C	-3.230995	1.532582	3.064650
C	-3.801092	0.272243	2.465821
H	-4.599893	-0.146824	3.090580
H	-4.215737	0.506029	1.477113
H	-3.027646	-0.502861	2.360031
N	-2.746156	1.455582	4.335928
C	-2.162674	2.621967	4.964885
H	-1.088192	2.466186	5.149145
H	-2.295979	3.488992	4.314747
H	-2.649405	2.811062	5.931767
C	-2.590360	0.228393	5.088409
H	-3.201756	-0.579598	4.682264
H	-1.537018	-0.095624	5.093065
H	-2.897260	0.396410	6.129446
O	-5.275053	1.419354	-0.584244
C	-6.384748	1.096582	-1.018395
C	-6.582996	0.801595	-2.481890
H	-7.354792	1.437784	-2.933023
H	-5.634501	0.976030	-2.998755

```
H          -6.882584       -0.243573       -2.637750
N          -7.469475        0.997971       -0.203690
C          -7.275437        1.094463        1.229530
H          -7.120925        0.096100        1.669814
H          -6.401723        1.715540        1.443320
H          -8.164232        1.543930        1.688736
C          -8.725526        0.400749       -0.611752
H          -8.915160        0.552798       -1.676826
H          -8.740428       -0.680264       -0.399529
H          -9.544865        0.870554       -0.053996
O           2.795555        3.868473       -0.197869
C           2.164190        4.573672       -0.989408
C           1.021864        4.005718       -1.793247
H           1.256758        3.998692       -2.867031
H           0.837246        2.969249       -1.473247
H           0.105347        4.597567       -1.665182
N           2.472758        5.887324       -1.181232
C           3.510284        6.513465       -0.389831
H           3.091342        7.337997        0.206121
H           3.949995        5.770933        0.279006
H           4.293248        6.926511       -1.041866
C           1.759677        6.785866       -2.064338
H           1.161106        6.241643       -2.797552
H           1.098606        7.459611       -1.497099
H           2.483059        7.404689       -2.612629
```

***

TS c1$_{ortho}$carb-c1NH$_{ortho}$

| Charge   0
| Electronic Energy (a.u.)   -3006.35372698

```
C           5.050573       -3.226640        0.504031
H           5.803129       -3.843655        0.013416
C           5.157783       -2.870498        1.820940
H           6.001094       -3.216921        2.419851
C           3.949454       -2.766298       -0.306805
C           4.177770       -2.045177        2.403019
H           4.253971       -1.726277        3.439444
C           3.098309       -1.685303        1.621713
C           2.045546       -0.790477        2.141517
C           2.294634        0.152107        3.140919
H           3.278294        0.205537        3.601528
C           1.299187        1.049193        3.499275
H           1.489941        1.804673        4.259251
C           0.067616        0.985422        2.855383
H          -0.732422        1.690202        3.073750
C          -0.122568        0.006330        1.892535
H          -1.074583       -0.101927        1.374048
C          -1.252091       -2.302116       -0.038675
C          -2.040690       -1.350972       -0.696833
C          -3.431102       -1.404756       -0.614112
C          -4.041878       -2.404764        0.139920
C          -3.265178       -3.337625        0.830052
C          -1.878277       -3.283961        0.741795
N           2.935735       -2.076913        0.329589
N           0.838766       -0.853548        1.536316
O           3.904665       -2.976317       -1.542763
```

Pd	0.731911	-2.191359	-0.119412
C	-5.520213	-2.483848	0.287836
F	-5.908525	-2.319903	1.569372
F	-6.172013	-1.559131	-0.430916
F	-6.005436	-3.681457	-0.085958
H	-1.285255	-4.033367	1.268765
H	-3.749239	-4.108011	1.432059
H	-4.035576	-0.645669	-1.112793
H	-1.544393	-0.557664	-1.264347
H	2.591752	-1.954719	-2.616410
N	1.749562	-1.726434	-3.155547
C	0.732529	-2.528094	-2.831076
C	0.844479	-3.590526	-1.854836
C	-0.532249	-2.365187	-3.485742
C	-0.200231	-4.568244	-1.783271
H	1.861583	-3.906763	-1.604531
C	-1.517303	-3.303467	-3.341527
H	-0.650802	-1.502764	-4.142738
C	-1.357290	-4.439416	-2.502940
H	-0.053028	-5.427415	-1.126698
H	-2.450976	-3.180817	-3.892489
H	-2.155282	-5.176018	-2.423300
H	1.429085	-0.621025	-3.374881
O	0.902865	0.597102	-3.638163
O	0.238940	0.736666	-1.494940
C	-0.003660	0.962994	-2.743322
O	-1.063874	1.535779	-3.130845
Cs	-2.124570	2.383989	-0.352196
Cs	3.239535	0.889023	-1.279419
O	5.612165	0.744047	0.788992
C	6.566219	-0.026406	0.662157
C	7.018498	-0.490094	-0.700704
H	6.780616	-1.552025	-0.856609
H	6.498881	0.098540	-1.463374
H	8.099350	-0.367388	-0.844411
N	7.264811	-0.499425	1.729927
C	6.973018	-0.015097	3.061830
H	7.832395	0.540023	3.466843
H	6.104829	0.646101	3.019698
H	6.759452	-0.855794	3.737339
C	8.408429	-1.382602	1.617490
H	8.301834	-2.074126	0.776067
H	9.350439	-0.824396	1.502116
H	8.480733	-1.983299	2.532563
O	-3.186428	2.367235	2.732159
C	-3.301261	1.249958	3.244822
C	-3.893790	0.099117	2.473598
H	-4.749580	-0.342817	2.999767
H	-4.232722	0.468082	1.497457
H	-3.156583	-0.702836	2.320829
N	-2.898678	1.001871	4.522990
C	-2.306229	2.056010	5.319477
H	-1.248703	1.834182	5.533134
H	-2.374281	3.001511	4.777460
H	-2.835428	2.144655	6.278350
C	-2.833115	-0.313649	5.124601
H	-3.457940	-1.035165	4.594902
H	-1.796048	-0.686758	5.135299

H	-3.183920	-0.258278	6.163761
O	-5.121136	1.561119	-0.531009
C	-6.209990	1.293951	-1.048570
C	-6.324392	1.084704	-2.535507
H	-7.049817	1.766862	-2.996345
H	-5.341398	1.259136	-2.983329
H	-6.640889	0.058867	-2.767596
N	-7.344766	1.181815	-0.307526
C	-7.240410	1.196440	1.138043
H	-7.152441	0.171249	1.532585
H	-6.359905	1.769452	1.439778
H	-8.139830	1.656913	1.564638
C	-8.590204	0.651014	-0.825154
H	-8.704041	0.861826	-1.890935
H	-8.653348	-0.437858	-0.670278
H	-9.428621	1.121461	-0.297049
O	2.748004	3.839857	-0.175329
C	2.124669	4.570268	-0.950642
C	1.019764	4.014823	-1.811844
H	1.305173	4.013496	-2.873585
H	0.821465	2.978586	-1.509523
H	0.094885	4.600605	-1.725681
N	2.408126	5.896659	-1.071264
C	3.395258	6.511152	-0.209237
H	2.933848	7.311082	0.387901
H	3.809448	5.754139	0.459086
H	4.205639	6.953422	-0.805368
C	1.703564	6.813263	-1.943582
H	1.197915	6.287542	-2.756476
H	0.960874	7.406725	-1.387134
H	2.425669	7.508861	-2.392164

***

c1NH$_{ortho}$

| Charge 0
| Electronic Energy (a.u.) -3006.36794213

C	5.069295	-3.250590	-0.553547
H	5.874123	-3.547689	-1.225787
C	5.077934	-3.588139	0.772583
H	5.892784	-4.184467	1.185573
C	4.010527	-2.442990	-1.111471
C	4.038093	-3.149173	1.613108
H	4.043290	-3.373652	2.676645
C	3.002522	-2.443028	1.034695
C	1.907905	-1.882141	1.853081
C	2.062914	-1.586278	3.209119
H	2.995510	-1.825994	3.714321
C	1.041804	-0.940191	3.890039
H	1.162691	-0.681439	4.940389
C	-0.122522	-0.604313	3.206012
H	-0.933274	-0.059960	3.686051
C	-0.224828	-0.959502	1.869721
H	-1.124031	-0.743120	1.290862
C	-1.218359	-2.066060	-1.028183
C	-1.967087	-0.886050	-1.149933
C	-3.361287	-0.910690	-1.146107
C	-4.029462	-2.125246	-1.008155

C	-3.302867	-3.308216	-0.855074
C	-1.913109	-3.276039	-0.868645
N	2.932063	-2.162532	-0.293486
N	0.761698	-1.574429	1.207322
O	4.057955	-2.001032	-2.282738
Pd	0.777865	-2.015165	-0.899024
C	-5.513699	-2.204363	-0.947916
F	-5.950233	-2.595739	0.268271
F	-6.115564	-1.035872	-1.211417
F	-6.011441	-3.103494	-1.815344
H	-1.366881	-4.215873	-0.770350
H	-3.829245	-4.255583	-0.729403
H	-3.918559	0.025424	-1.216963
H	-1.443486	0.069889	-1.239167
H	2.703843	-0.628665	-3.119230
N	1.889744	-0.184737	-3.564258
C	0.934272	-1.081812	-3.606834
C	1.030082	-2.432710	-3.023268
C	-0.319201	-0.767576	-4.274449
C	0.082759	-3.429759	-3.467175
H	2.062194	-2.781582	-2.902227
C	-1.211172	-1.744306	-4.594564
H	-0.471193	0.264628	-4.591991
C	-1.005591	-3.112832	-4.221688
H	0.255762	-4.464489	-3.162838
H	-2.106493	-1.483809	-5.161782
H	-1.728521	-3.870863	-4.520080
H	1.016169	1.478899	-3.240676
O	0.396746	2.252037	-3.258992
O	0.135416	1.545716	-1.141146
C	-0.319770	2.257261	-2.082187
O	-1.330570	2.980187	-2.067283
Cs	-2.040453	2.336191	0.947541
Cs	3.129238	1.118315	-0.652436
O	5.293807	0.173450	1.421015
C	6.323478	-0.428405	1.106904
C	6.942988	-0.254464	-0.257955
H	6.840036	-1.166679	-0.861356
H	6.431072	0.563158	-0.775243
H	8.011949	-0.013888	-0.198765
N	6.963117	-1.267039	1.965894
C	6.506653	-1.396096	3.332540
H	7.309710	-1.113853	4.029141
H	5.647864	-0.739148	3.485949
H	6.214447	-2.434646	3.547756
C	8.169431	-1.999467	1.638104
H	8.215089	-2.243498	0.572709
H	9.076736	-1.440557	1.915047
H	8.170685	-2.944929	2.195307
O	-3.323257	0.897694	3.582501
C	-3.491460	-0.323274	3.505178
C	-4.003663	-0.955847	2.237418
H	-4.907013	-1.553142	2.414467
H	-4.247393	-0.158136	1.524525
H	-3.252264	-1.625359	1.792672
N	-3.217467	-1.152341	4.551803
C	-2.692361	-0.614190	5.789242
H	-1.660885	-0.962684	5.956488

H	-2.701688	0.476712	5.742358
H	-3.306086	-0.950431	6.636323
C	-3.217191	-2.598637	4.479645
H	-3.777555	-2.963222	3.617194
H	-2.187286	-2.985050	4.418261
H	-3.680137	-3.011130	5.385791
O	-5.001411	1.734617	0.230591
C	-6.079784	1.757734	-0.371952
C	-6.155061	2.244926	-1.794934
H	-6.847979	3.088531	-1.906054
H	-5.155058	2.566700	-2.100856
H	-6.491594	1.448472	-2.471658
N	-7.236728	1.357794	0.219129
C	-7.181023	0.734158	1.526036
H	-7.148876	-0.364023	1.436032
H	-6.288952	1.072422	2.059230
H	-8.075448	1.007295	2.099679
C	-8.479313	1.157297	-0.499339
H	-8.549379	1.811619	-1.371030
H	-8.580840	0.111936	-0.831316
H	-9.320777	1.389084	0.165213
O	3.274662	4.117288	0.322861
C	2.522531	5.013505	-0.068846
C	1.092550	4.715127	-0.437836
H	0.874292	4.986375	-1.478708
H	0.913415	3.640829	-0.319632
H	0.385945	5.264346	0.199278
N	2.940298	6.305183	-0.181739
C	4.328867	6.638896	0.057068
H	4.397709	7.496088	0.740370
H	4.834903	5.779039	0.500009
H	4.831698	6.908664	-0.884149
C	2.145743	7.374598	-0.750363
H	1.075645	7.175301	-0.659737
H	2.361190	8.306634	-0.212214
H	2.387271	7.531509	-1.813211

***

TS c1NH$_{ortho}$-c2NH$_{ortho}$

| Charge 0
| Electronic Energy (a.u.) -3006.32889842

C	4.975943	-3.091261	0.678286
H	5.784480	-3.599656	0.156657
C	5.162899	-2.474547	1.890777
H	6.143689	-2.486425	2.364926
C	3.683185	-3.092006	0.081618
C	4.090865	-1.834630	2.531514
H	4.239184	-1.353046	3.493698
C	2.852271	-1.859224	1.914698
C	1.659250	-1.203550	2.497740
C	1.720845	-0.420287	3.651647
H	2.662688	-0.275984	4.173604
C	0.571146	0.194118	4.125363
H	0.613565	0.818621	5.015446
C	-0.627598	0.004500	3.448249
H	-1.549526	0.484765	3.769659

C	-0.619669	-0.800277	2.317881
H	-1.537148	-1.001620	1.759909
C	-1.263915	-2.304170	-0.684834
C	-1.666584	-1.491380	-1.760833
C	-3.008249	-1.238589	-2.016599
C	-3.994609	-1.817747	-1.213107
C	-3.628421	-2.681577	-0.184726
C	-2.277462	-2.917369	0.070456
N	2.658883	-2.473321	0.720419
N	0.488226	-1.379150	1.843205
O	3.480186	-3.674468	-1.042295
Pd	0.643932	-2.436694	-0.076861
C	-5.415740	-1.456805	-1.463325
F	-6.251495	-1.945429	-0.534901
F	-5.590203	-0.117246	-1.472426
F	-5.860344	-1.888019	-2.656014
H	-2.016741	-3.570249	0.907473
H	-4.392292	-3.151879	0.433549
H	-3.291818	-0.567040	-2.830329
H	-0.917608	-1.014044	-2.393119
H	1.436953	-0.978501	-1.988631
N	1.577787	-1.258989	-2.965696
C	1.242967	-2.540354	-3.048660
C	1.005308	-3.403658	-1.894588
C	1.136270	-3.159746	-4.346472
C	0.476217	-4.708593	-2.124830
H	2.250445	-3.577356	-1.413897
C	0.677952	-4.441049	-4.500362
H	1.369302	-2.544684	-5.218757
C	0.324281	-5.243371	-3.384009
H	0.240520	-5.327094	-1.253179
H	0.575985	-4.853193	-5.506045
H	-0.035885	-6.261400	-3.527830
H	0.601542	0.234757	-3.492613
O	-0.048934	0.951796	-3.705402
O	0.279758	1.270189	-1.503394
C	-0.379955	1.589644	-2.530003
O	-1.310034	2.413795	-2.618631
Cs	-2.558056	1.640032	0.101297
Cs	3.235569	0.867448	-0.647948
O	5.841580	1.213365	1.119395
C	6.730967	0.392921	0.880643
C	7.052522	-0.015824	-0.535284
H	6.689201	-1.034189	-0.737249
H	6.552499	0.674473	-1.221871
H	8.128602	0.000253	-0.745782
N	7.454371	-0.200632	1.868612
C	7.268583	0.202550	3.246588
H	8.186460	0.663319	3.640557
H	6.451088	0.924382	3.301344
H	7.029270	-0.670045	3.871443
C	8.529316	-1.143918	1.628810
H	8.334375	-1.760351	0.745313
H	9.499379	-0.637878	1.504229
H	8.605477	-1.817401	2.492271
O	-3.740773	1.222157	3.014319
C	-4.131864	0.168640	3.531114
C	-4.467981	-1.025093	2.675006

```
H          -5.511201       -1.346621        2.794190
H          -4.306331       -0.749444        1.627176
H          -3.826584       -1.883761        2.918126
N          -4.249997        0.029448        4.876813
C          -3.846862        1.107265        5.757322
H          -2.972103        0.807217        6.353791
H          -3.593423        1.986263        5.161520
H          -4.664366        1.356147        6.447492
C          -4.610343       -1.200877        5.552093
H          -5.086791       -1.911385        4.873876
H          -3.723866       -1.677413        5.997580
H          -5.318556       -0.976519        6.360685
O          -5.326899        2.997043       -0.410570
C          -6.432077        2.616068       -0.801932
C          -6.959141        3.027881       -2.151997
H          -7.895405        3.594802       -2.073209
H          -6.207002        3.657410       -2.635482
H          -7.153632        2.153362       -2.787282
N          -7.229831        1.811978       -0.046140
C          -6.712716        1.217172        1.168321
H          -6.655961        0.124699        1.050564
H          -5.715098        1.610150        1.381624
H          -7.374988        1.436564        2.017930
C          -8.465637        1.223416       -0.518915
H          -8.956164        1.853236       -1.264530
H          -8.289307        0.225817       -0.953062
H          -9.154182        1.109888        0.328181
O           3.581531        3.956140       -1.090794
C           3.258598        5.109142       -1.384466
C           1.960022        5.388398       -2.097746
H           2.127592        5.843660       -3.082366
H           1.435364        4.439077       -2.237215
H           1.316775        6.067110       -1.523358
N           4.049699        6.176244       -1.086538
C           5.283381        5.986829       -0.353188
H           5.250286        6.539966        0.597027
H           5.423988        4.923898       -0.147312
H           6.136217        6.361031       -0.937329
C           3.722091        7.559129       -1.363153
H           2.880351        7.647202       -2.052450
H           3.474680        8.096171       -0.434728
H           4.588681        8.055903       -1.821043
***
```

c2NH$_{ortho}$

```
| Charge                            0
| Electronic Energy (a.u.)   -3006.35299684

C          -6.245140        1.037091        1.867187
H          -7.138476        1.648425        1.764976
C          -6.235409       -0.155945        2.554448
H          -7.143444       -0.522528        3.030129
C          -5.047493        1.479622        1.281766
C          -5.051376       -0.891766        2.643766
H          -5.025065       -1.826532        3.196700
C          -3.912960       -0.397025        2.029851
C          -2.630635       -1.127707        2.071325
C          -2.567608       -2.500537        2.317152
```

H	-3.478276	-3.071921	2.478358
C	-1.334373	-3.137540	2.306072
H	-1.271236	-4.211032	2.474334
C	-0.187349	-2.388536	2.066490
H	0.797045	-2.848155	2.019705
C	-0.328979	-1.026982	1.838268
H	0.536054	-0.388362	1.653949
C	0.024399	2.244224	0.683478
C	0.622105	2.664568	-0.519032
C	1.951045	3.061977	-0.574359
C	2.726588	3.076310	0.588353
C	2.162834	2.681195	1.798479
C	0.829276	2.266585	1.835632
N	-3.907727	0.785024	1.361776
N	-1.515425	-0.409740	1.822111
O	-5.074502	2.625518	0.619775
Pd	-1.862889	1.565725	0.860063
C	4.148211	3.502817	0.479345
F	4.779608	3.533704	1.662391
F	4.861596	2.676223	-0.317141
F	4.273559	4.724964	-0.063460
H	0.416766	1.950750	2.797037
H	2.755367	2.695655	2.713244
H	2.390874	3.352307	-1.530143
H	0.047509	2.662804	-1.443216
H	-1.906275	1.317239	-1.692244
N	-2.354949	1.994290	-2.318514
C	-2.589322	3.108701	-1.595616
C	-2.356071	3.244197	-0.174587
C	-3.116939	4.273455	-2.238533
C	-2.523207	4.480381	0.455859
H	-4.153846	2.865373	0.318960
C	-3.314527	5.473580	-1.575705
H	-3.340206	4.194631	-3.306329
C	-3.010422	5.608191	-0.216887
H	-2.306203	4.558694	1.526219
H	-3.708969	6.327427	-2.130806
H	-3.156509	6.552856	0.306276
H	-0.795040	1.974169	-3.358984
O	0.115454	2.054397	-3.757416
O	0.252073	0.141767	-2.598141
C	0.870932	1.016284	-3.269620
O	2.085167	1.049435	-3.537512
Cs	2.655788	-0.441425	-0.702929
Cs	-1.756016	-1.782315	-1.582761
O	-4.666528	-2.021443	-0.543183
C	-5.671163	-1.343204	-0.775737
C	-5.573497	-0.064178	-1.570462
H	-6.144564	0.763873	-1.136004
H	-4.521121	0.240699	-1.635157
H	-5.948219	-0.220376	-2.591857
N	-6.904594	-1.726447	-0.348283
C	-7.060205	-2.939929	0.425958
H	-7.620225	-3.694353	-0.146506
H	-6.075779	-3.339332	0.678625
H	-7.619478	-2.727813	1.348603
C	-8.142125	-1.028404	-0.624699
H	-8.010238	-0.248419	-1.376875

```
H          -8.885980       -1.741606       -1.006723
H          -8.549079       -0.572602        0.291499
O           3.338160       -2.214932        1.863961
C           2.985672       -1.963184        3.020953
C           2.850718       -0.538670        3.494717
H           3.592515       -0.299624        4.269102
H           3.014567        0.122719        2.637351
H           1.856351       -0.337013        3.915301
N           2.694708       -2.944283        3.916430
C           2.692685       -4.332622        3.502011
H           1.670548       -4.741626        3.539066
H           3.074656       -4.407368        2.481779
H           3.324495       -4.929116        4.173570
C           2.143621       -2.698283        5.234362
H           2.476644       -1.739468        5.638655
H           1.041590       -2.715953        5.216501
H           2.486758       -3.486680        5.915374
O           5.771458       -0.437263       -0.874943
C           6.650347        0.335161       -0.488146
C           7.624665        0.950591       -1.457871
H           8.664228        0.694622       -1.216784
H           7.392941        0.578939       -2.459889
H           7.544262        2.045948       -1.457404
N           6.791889        0.667881        0.826275
C           5.768404        0.264400        1.769151
H           5.041924        1.079170        1.929446
H           5.238786       -0.617057        1.397419
H           6.230483        0.023734        2.735528
C           7.674139        1.723547        1.284534
H           8.603159        1.748663        0.709089
H           7.190315        2.710813        1.222012
H           7.936322        1.535832        2.332568
O           1.067795       -3.250099       -1.390372
C           1.651671       -3.599083       -2.424479
C           1.703980       -2.683466       -3.621336
H           1.256366       -3.151240       -4.508466
H           1.159287       -1.759408       -3.379550
H           2.739694       -2.419404       -3.877282
N           2.269034       -4.805917       -2.519236
C           2.245902       -5.726017       -1.401076
H           3.267873       -5.917576       -1.041553
H           1.652721       -5.296130       -0.591665
H           1.805126       -6.685308       -1.708700
C           2.976719       -5.293788       -3.683950
H           3.032350       -4.538862       -4.470668
H           4.002366       -5.576640       -3.404561
H           2.477020       -6.186222       -4.089702
```
***

TS c2NH$_{ortho}$-c2$_{ortho}$
| Charge   0
| Electronic Energy (a.u.)   -3006.3429684

```
C           6.175029       -1.056319        2.090567
H           7.062966       -1.682870        2.058829
C           6.152484        0.171102        2.715923
H           7.044583        0.548760        3.212441
```

C	4.997919	-1.507611	1.475122
C	4.978954	0.928914	2.713960
H	4.944328	1.891911	3.215831
C	3.860982	0.421319	2.073701
C	2.587881	1.165228	2.008901
C	2.522203	2.548225	2.182379
H	3.426441	3.119189	2.378351
C	1.299221	3.193714	2.051757
H	1.238162	4.275638	2.157155
C	0.163216	2.444099	1.766028
H	-0.807819	2.905248	1.600548
C	0.305047	1.070556	1.624435
H	-0.549943	0.427545	1.412393
C	-0.045602	-2.227328	0.732348
C	-0.681790	-2.638806	-0.451985
C	-2.017598	-3.017565	-0.462082
C	-2.758045	-3.020138	0.722514
C	-2.153152	-2.629842	1.914593
C	-0.813013	-2.235881	1.910333
N	3.869898	-0.792736	1.466372
N	1.482117	0.443774	1.727830
O	5.027322	-2.687239	0.869701
Pd	1.853385	-1.565646	0.896531
C	-4.185685	-3.435428	0.655937
F	-4.799983	-3.402059	1.848260
F	-4.901429	-2.641984	-0.170292
F	-4.332034	-4.682131	0.177274
H	-0.367874	-1.918305	2.856351
H	-2.718240	-2.630734	2.846593
H	-2.488647	-3.281855	-1.410353
H	-0.154166	-2.642323	-1.405434
H	1.870602	-1.295717	-1.590289
N	2.167389	-2.017576	-2.249640
C	2.371609	-3.188136	-1.549791
C	2.300784	-3.270110	-0.125737
C	2.637091	-4.378535	-2.262967
C	2.474330	-4.511406	0.496424
H	4.128089	-2.898128	0.506155
C	2.816594	-5.596005	-1.617365
H	2.692244	-4.321268	-3.352748
C	2.743753	-5.678355	-0.227636
H	2.407734	-4.570713	1.586874
H	3.016628	-6.490965	-2.208295
H	2.884541	-6.628872	0.286412
H	1.238869	-2.071698	-2.996051
O	0.056325	-2.176850	-3.628128
O	-0.168748	-0.138333	-2.726520
C	-0.720681	-1.169971	-3.269892
O	-1.971654	-1.255223	-3.453291
Cs	-2.674398	0.396629	-0.909459
Cs	1.826922	1.783767	-1.746706
O	4.669391	1.869568	-0.520840
C	5.654567	1.154123	-0.723360
C	5.524559	-0.139686	-1.490053
H	5.992958	-0.992736	-0.984829
H	4.459600	-0.366528	-1.628815
H	5.991642	-0.049739	-2.480790
N	6.891812	1.506159	-0.280266

C	7.079895	2.762898	0.413946
H	7.655989	3.465591	-0.206897
H	6.106186	3.201060	0.643466
H	7.636849	2.595985	1.346720
C	8.115329	0.776559	-0.534536
H	7.928128	-0.170006	-1.044092
H	8.794522	1.376085	-1.158581
H	8.628571	0.563276	0.414672
O	-3.277725	2.274155	1.624956
C	-2.920015	2.080637	2.791081
C	-2.774241	0.681832	3.334494
H	-3.511245	0.477879	4.123587
H	-2.936467	-0.023906	2.512494
H	-1.777105	0.506557	3.760536
N	-2.635270	3.107073	3.637835
C	-2.647604	4.471099	3.149559
H	-1.621152	4.862371	3.059120
H	-3.130735	4.498959	2.170745
H	-3.198997	5.113786	3.848264
C	-2.057188	2.927604	4.955221
H	-2.384506	1.991498	5.414574
H	-0.955314	2.940073	4.916496
H	-2.384849	3.750822	5.602057
O	-5.810623	0.436365	-0.925217
C	-6.690243	-0.298721	-0.473503
C	-7.698781	-0.950068	-1.383675
H	-8.727239	-0.658852	-1.134899
H	-7.483685	-0.641174	-2.410420
H	-7.641001	-2.045081	-1.322532
N	-6.804630	-0.555373	0.860739
C	-5.755732	-0.117008	1.758836
H	-5.067840	-0.948308	1.987973
H	-5.189870	0.701857	1.306365
H	-6.196446	0.230392	2.702943
C	-7.694105	-1.564640	1.401071
H	-8.629043	-1.619857	0.837493
H	-7.221058	-2.559297	1.404690
H	-7.943885	-1.300538	2.435933
O	-0.973689	3.109704	-1.162510
C	-1.484903	3.614235	-2.170193
C	-1.564304	2.833244	-3.458471
H	-1.032168	3.344188	-4.272188
H	-1.113559	1.840549	-3.296544
H	-2.606053	2.701699	-3.783238
N	-1.991656	4.875314	-2.153391
C	-1.941169	5.663272	-0.939505
H	-2.958457	5.909607	-0.601386
H	-1.427002	5.095467	-0.160018
H	-1.403378	6.605079	-1.121297
C	-2.618205	5.539470	-3.276386
H	-2.652061	4.896635	-4.157947
H	-3.647864	5.827488	-3.017129
H	-2.064291	6.454717	-3.533299

***

c2*ortho*

| Charge 0
| Electronic Energy (a.u.) -3006.34371125

C	-2.516351	-3.266425	3.275393
H	-3.535339	-3.431178	3.617235
C	-1.634388	-4.299981	3.032188
H	-1.949374	-5.332195	3.176928
C	-2.063631	-1.958713	3.065680
C	-0.338004	-4.022004	2.604274
H	0.353940	-4.834831	2.406392
C	0.045034	-2.697149	2.442255
C	1.428020	-2.309868	2.091381
C	2.448008	-3.248005	1.910834
H	2.236108	-4.312600	1.958334
C	3.741225	-2.815228	1.668602
H	4.540636	-3.542752	1.524490
C	4.006720	-1.448281	1.621235
H	5.006025	-1.060815	1.435974
C	2.949399	-0.575037	1.817594
H	3.097590	0.505600	1.786519
C	0.901897	2.014055	1.718727
C	1.064551	2.284109	0.349148
C	1.800673	3.380875	-0.094769
C	2.384388	4.242178	0.833588
C	2.241641	3.992113	2.199688
C	1.505573	2.894574	2.632057
N	-0.812761	-1.681838	2.694347
N	1.696141	-0.990072	2.041968
O	-2.904759	-0.956435	3.296225
Pd	-0.024836	0.319379	2.242715
C	3.153538	5.446625	0.418044
F	2.684650	6.568220	0.994233
F	4.451769	5.372027	0.777264
F	3.131060	5.654453	-0.906531
H	1.398910	2.729489	3.705955
H	2.698868	4.668243	2.923852
H	1.908503	3.573314	-1.162338
H	0.589314	1.612829	-0.372227
H	-1.760575	0.713833	0.027370
N	-2.486934	1.403275	0.319487
C	-2.508006	1.982380	1.558866
C	-1.597165	1.596853	2.583417
C	-3.475365	2.973781	1.840397
C	-1.735695	2.205470	3.838201
H	-2.452971	-0.104305	3.066109
C	-3.576382	3.552865	3.098248
H	-4.149062	3.283877	1.037386
C	-2.714600	3.162963	4.121499
H	-1.044337	1.921654	4.637176
H	-4.338399	4.312372	3.277974
H	-2.789612	3.602228	5.116123
H	-3.055318	1.840455	-0.403360
O	0.213996	-2.422367	-0.372037
O	-0.638211	-0.393290	-0.813559
C	-0.800452	-1.640520	-0.427051
O	-1.983011	-2.050275	-0.134255
Cs	-3.381803	-0.610900	-2.415841
Cs	2.329278	-0.705029	-1.643070
O	5.038843	0.799343	-0.939569
C	5.627196	1.159855	-1.964459

C	5.921242	0.177768	-3.071042
H	7.001790	0.051786	-3.223413
H	5.494627	-0.792403	-2.791842
H	5.489216	0.502204	-4.026970
N	6.034003	2.444998	-2.144866
C	5.670487	3.461322	-1.179338
H	5.011778	4.207447	-1.646003
H	5.151936	2.993181	-0.340304
H	6.567371	3.978829	-0.810696
C	6.666278	2.948112	-3.346587
H	7.128635	2.146345	-3.926020
H	5.942244	3.476627	-3.986060
H	7.454437	3.659696	-3.067123
O	4.623998	-2.834793	-1.782771
C	5.532005	-3.272089	-1.069818
C	6.655586	-2.377056	-0.614025
H	7.543220	-2.514228	-1.247920
H	6.314009	-1.340345	-0.709643
H	6.956061	-2.561087	0.424583
N	5.557811	-4.568165	-0.658478
C	4.419401	-5.430260	-0.900529
H	4.126079	-5.922310	0.038298
H	3.582726	-4.834407	-1.273198
H	4.661216	-6.211041	-1.636067
C	6.632926	-5.161808	0.111614
H	7.581882	-4.648875	-0.063711
H	6.414028	-5.154415	1.191503
H	6.757124	-6.205810	-0.202722
O	-6.429157	-1.820995	-1.555175
C	-5.842258	-2.882126	-1.307027
C	-5.913662	-4.033259	-2.280347
H	-6.188795	-4.979249	-1.798472
H	-6.661993	-3.788793	-3.039676
H	-4.947354	-4.183891	-2.781758
N	-5.094512	-3.051897	-0.189764
C	-4.977222	-1.981158	0.786105
H	-3.969709	-1.536297	0.727237
H	-5.752738	-1.233657	0.603234
H	-5.115862	-2.398510	1.794091
C	-4.213349	-4.177876	0.072129
H	-4.260779	-4.919211	-0.728826
H	-3.184253	-3.786839	0.150493
H	-4.502360	-4.670546	1.013688
O	-4.388686	2.483364	-1.803334
C	-5.590200	2.316934	-1.545315
C	-6.015418	1.379038	-0.446446
H	-6.449134	0.454923	-0.859151
H	-5.125779	1.104545	0.131825
H	-6.751724	1.829559	0.229960
N	-6.561946	2.952389	-2.244745
C	-6.216214	3.927302	-3.259271
H	-6.720602	4.880325	-3.046963
H	-5.135650	4.081612	-3.261995
H	-6.536804	3.581783	-4.252432
C	-7.983666	2.781872	-2.020582
H	-8.198978	1.851436	-1.491133
H	-8.400351	3.624211	-1.448401
H	-8.495682	2.742160	-2.990988

***

$c2b_{ortho}$

| Charge 0
| Electronic Energy (a.u.) -3006.39415659

C	1.827657	-4.204718	-2.069974
H	2.814821	-4.659154	-1.987678
C	0.718072	-4.943352	-2.385878
H	0.802750	-6.014527	-2.570566
C	1.723409	-2.801350	-1.769710
C	-0.539341	-4.321460	-2.447177
H	-1.438296	-4.902421	-2.634590
C	-0.593538	-2.951997	-2.238434
C	-1.889542	-2.236474	-2.252020
C	-3.039316	-2.768299	-2.840431
H	-3.000595	-3.726241	-3.353863
C	-4.225513	-2.048838	-2.798380
H	-5.123252	-2.446926	-3.269157
C	-4.247498	-0.814910	-2.157846
H	-5.154144	-0.219310	-2.087142
C	-3.065958	-0.339588	-1.610532
H	-3.039689	0.629831	-1.111313
C	-0.594515	1.797134	-1.383269
C	-0.742853	2.283789	-0.071718
C	-1.410032	3.476598	0.197939
C	-1.929132	4.231422	-0.853189
C	-1.774694	3.787197	-2.167510
C	-1.111945	2.591322	-2.422227
N	0.499628	-2.200631	-1.965862
N	-1.912801	-1.018715	-1.663073
O	2.706328	-2.145367	-1.339840
Pd	0.083996	-0.065427	-1.694700
C	-2.638352	5.519023	-0.622572
F	-2.064942	6.539040	-1.286868
F	-3.916294	5.481636	-1.054224
F	-2.679734	5.872275	0.670033
H	-1.005343	2.266018	-3.459644
H	-2.173118	4.383465	-2.990099
H	-1.514962	3.824835	1.225237
H	-0.315408	1.722414	0.764049
H	2.146609	1.091337	0.100968
N	2.536947	1.908629	-0.372004
C	2.654117	1.682315	-1.749187
C	1.745935	0.842811	-2.432102
C	3.679947	2.328505	-2.457685
C	1.946259	0.666276	-3.806426
H	2.381141	-1.360916	0.193878
C	3.849597	2.135297	-3.826838
H	4.351139	2.996064	-1.911240
C	2.985623	1.286452	-4.510705
H	1.264771	0.015615	-4.362665
H	4.654815	2.652524	-4.350888
H	3.102802	1.114529	-5.581043
H	3.447040	2.125673	0.035926
O	0.459243	-1.890263	1.390132
O	0.882475	0.084068	2.400619
C	1.138169	-0.868213	1.627592

O	2.358824	-0.753752	0.979389
Cs	3.856932	1.052057	3.077821
Cs	-2.255740	-0.496473	1.966755
O	-4.737421	1.000166	0.691865
C	-5.503645	1.373676	1.586673
C	-6.012964	0.393296	2.613972
H	-7.106964	0.303196	2.585720
H	-5.578761	-0.587436	2.390944
H	-5.730392	0.689419	3.632919
N	-5.917834	2.663898	1.685868
C	-5.376178	3.664874	0.789493
H	-4.721307	4.359088	1.337053
H	-4.802050	3.172076	0.002244
H	-6.190780	4.247132	0.337618
C	-6.731827	3.186312	2.763648
H	-7.276310	2.392468	3.279033
H	-6.115804	3.728104	3.497942
H	-7.467536	3.890042	2.352544
O	-4.313713	-2.634638	1.055434
C	-5.196061	-2.994622	0.271027
C	-6.377141	-2.099128	-0.006170
H	-7.195259	-2.328284	0.692121
H	-6.049614	-1.067137	0.168027
H	-6.771068	-2.179344	-1.024639
N	-5.141722	-4.200445	-0.356649
C	-3.969427	-5.037872	-0.212762
H	-3.640791	-5.382521	-1.204325
H	-3.165007	-4.468681	0.258732
H	-4.189885	-5.923999	0.400978
C	-6.186143	-4.763546	-1.186471
H	-7.137916	-4.246989	-1.046006
H	-5.912072	-4.731412	-2.252866
H	-6.335722	-5.816059	-0.909056
O	4.858654	-1.930517	2.970329
C	3.910598	-2.704877	2.810321
C	2.751838	-2.731310	3.776184
H	2.739530	-3.672765	4.343074
H	2.864330	-1.904963	4.485875
H	1.788430	-2.642071	3.257890
N	3.864307	-3.606023	1.793696
C	4.866772	-3.566556	0.746936
H	4.433019	-3.140578	-0.172006
H	5.705982	-2.952799	1.082623
H	5.225066	-4.584216	0.535771
C	2.671932	-4.379717	1.490306
H	2.256805	-4.841004	2.393753
H	1.896885	-3.752393	1.021485
H	2.949494	-5.190614	0.806706
O	5.515830	1.896718	0.498055
C	5.872863	1.134512	-0.411987
C	5.333995	-0.266811	-0.506820
H	6.136053	-1.007321	-0.384210
H	4.586855	-0.418013	0.278594
H	4.846510	-0.460208	-1.472205
N	6.771493	1.517486	-1.355745
C	7.233289	2.889138	-1.410278
H	6.881974	3.373697	-2.334506
H	6.845894	3.434937	-0.547572

H	8.331193	2.922128	-1.401710
C	7.125540	0.702005	-2.500598
H	7.121275	-0.362532	-2.253347
H	6.435585	0.873318	-3.343350
H	8.138733	0.968281	-2.824674

***

TS c2b$_{ortho}$-c3

| Charge | 0 |
| Electronic Energy (a.u.) | -3006.38024852 |

C	1.955601	4.281829	1.677441
H	2.957849	4.685718	1.534979
C	0.878004	5.095891	1.914221
H	1.002912	6.178101	1.961339
C	1.796976	2.857168	1.578395
C	-0.394623	4.530152	2.078033
H	-1.269685	5.159023	2.219290
C	-0.499599	3.146616	2.030949
C	-1.823700	2.494102	2.164870
C	-2.916795	3.131221	2.760593
H	-2.809754	4.127167	3.184760
C	-4.132907	2.467037	2.840258
H	-4.989288	2.947357	3.311640
C	-4.237004	1.179415	2.323232
H	-5.169572	0.620493	2.355657
C	-3.103908	0.608134	1.763048
H	-3.138572	-0.408429	1.364707
C	-0.385761	-1.832598	1.459529
C	-0.273713	-2.305513	0.129879
C	-1.127512	-3.283480	-0.363492
C	-2.117160	-3.836747	0.449692
C	-2.231329	-3.411866	1.775065
C	-1.371704	-2.440504	2.268080
N	0.554499	2.326324	1.829652
N	-1.928583	1.240868	1.680873
O	2.768433	2.111642	1.271844
Pd	0.094942	0.104585	1.715270
C	-3.044955	-4.832775	-0.145351
F	-2.393751	-5.881975	-0.682229
F	-3.920341	-5.328834	0.739131
F	-3.769793	-4.305500	-1.156501
H	-1.485870	-2.127200	3.306712
H	-2.999886	-3.834263	2.420935
H	-1.038323	-3.606580	-1.402802
H	0.461195	-1.868438	-0.545259
H	2.390941	-0.934754	0.070727
N	2.724542	-1.808717	0.485608
C	2.491481	-1.930104	1.849436
C	1.308200	-1.428205	2.450771
C	3.437794	-2.610650	2.635597
C	1.170876	-1.615029	3.840557
H	2.488766	1.350753	-0.296011
C	3.267330	-2.774513	4.002948
H	4.326582	-3.008113	2.139874
C	2.127883	-2.258301	4.619690
H	0.265288	-1.252169	4.330390
H	4.024294	-3.302386	4.583798

H	1.977343	-2.366758	5.693477
H	3.702382	-1.964947	0.243320
O	0.582002	1.740380	-1.579102
O	1.082766	-0.289883	-2.429350
C	1.290777	0.722039	-1.723769
O	2.494879	0.682045	-1.028486
Cs	4.048565	-1.367242	-2.857497
Cs	-2.088920	0.117824	-1.678229
O	-4.830170	-0.974003	-0.396135
C	-5.668011	-1.272958	-1.253542
C	-5.853961	-0.407692	-2.476615
H	-6.899178	-0.099743	-2.609151
H	-5.240965	0.492708	-2.354823
H	-5.544953	-0.933235	-3.390737
N	-6.451414	-2.378050	-1.142808
C	-6.386389	-3.195836	0.051037
H	-6.274545	-4.252477	-0.223215
H	-5.530275	-2.886328	0.654609
H	-7.307823	-3.086332	0.643006
C	-7.501802	-2.735976	-2.074001
H	-7.346092	-2.277012	-3.052604
H	-7.505525	-3.825293	-2.210347
H	-8.491526	-2.436916	-1.695073
O	-4.008787	2.505760	-1.252083
C	-4.854290	3.062474	-0.544852
C	-6.127782	2.344589	-0.177556
H	-6.919568	2.588100	-0.900940
H	-5.922225	1.270023	-0.234739
H	-6.502325	2.582600	0.823619
N	-4.681722	4.332965	-0.091479
C	-3.431983	5.024947	-0.326928
H	-3.048287	5.428778	0.622069
H	-2.700586	4.330946	-0.747239
H	-3.573760	5.864531	-1.023597
C	-5.664492	5.097228	0.648460
H	-6.666748	4.675912	0.545139
H	-5.406908	5.155489	1.717765
H	-5.692476	6.121265	0.252162
O	5.072955	1.618351	-2.899491
C	4.123436	2.408490	-2.922251
C	3.057506	2.318438	-3.986045
H	3.069461	3.208177	-4.630604
H	3.252577	1.439712	-4.609321
H	2.053619	2.241613	-3.548325
N	3.996011	3.425590	-2.030409
C	4.905906	3.509467	-0.903779
H	4.431326	3.098230	0.002465
H	5.811495	2.942879	-1.133682
H	5.172390	4.560325	-0.724841
C	2.779049	4.214083	-1.920225
H	2.454130	4.581403	-2.900763
H	1.960914	3.627922	-1.471115
H	2.987461	5.089496	-1.294287
O	5.764403	-1.841911	-0.184973
C	5.931434	-1.000332	0.711221
C	5.470569	0.418783	0.523653
H	6.236414	1.143436	0.826735
H	5.239366	0.580697	-0.535729

H	4.557628	0.637335	1.098025
N	6.520201	-1.326227	1.889830
C	7.062754	-2.652005	2.100363
H	6.533333	-3.159937	2.920578
H	6.958019	-3.237273	1.184732
H	8.125371	-2.582319	2.372888
C	6.620407	-0.446965	3.036103
H	6.051180	0.473523	2.890682
H	6.213706	-0.957965	3.920325
H	7.669667	-0.189667	3.242783

***

c3$_{ortho}$-HCO$_3$

| Charge 0
| Electronic Energy (a.u.) -3006.42444585

C	1.411913	4.457774	-1.259017
H	2.356117	4.687661	-1.752415
C	0.291849	5.225686	-1.455269
H	0.323554	6.084059	-2.127127
C	1.382345	3.324755	-0.381699
C	-0.898920	4.901353	-0.787737
H	-1.807130	5.483168	-0.931064
C	-0.894990	3.792765	0.047055
C	-2.143147	3.422699	0.764700
C	-2.907268	4.403647	1.403979
H	-2.582187	5.442423	1.390600
C	-4.062283	4.025226	2.078412
H	-4.663009	4.768735	2.600289
C	-4.430152	2.683773	2.082313
H	-5.325338	2.342122	2.598463
C	-3.619472	1.778715	1.402979
H	-3.887469	0.717891	1.360879
C	-0.096472	-1.654014	1.864926
C	0.296336	-2.172007	0.617557
C	-0.427630	-3.175014	-0.007073
C	-1.579292	-3.689949	0.586990
C	-1.994751	-3.192050	1.817699
C	-1.253311	-2.199651	2.447590
N	0.196431	3.023827	0.250354
N	-2.501627	2.129960	0.762339
O	2.411268	2.624030	-0.164068
Pd	0.319061	1.257480	1.522929
C	-2.374892	-4.696984	-0.168256
F	-1.605855	-5.676742	-0.669343
F	-3.326013	-5.279705	0.569022
F	-2.991995	-4.136578	-1.232187
H	-1.575656	-1.856656	3.429117
H	-2.887829	-3.584708	2.299589
H	-0.103437	-3.529394	-0.985920
H	1.146321	-1.770024	0.079109
H	2.748736	-0.942623	0.835475
N	2.870209	-1.319715	1.780182
C	2.095894	-0.595055	2.700971
C	0.653762	-0.588111	2.588970
C	2.707349	0.047341	3.773174
C	-0.052758	0.260555	3.502801
H	2.372226	0.988427	-0.723169

C	1.971760	0.776029	4.717862
H	3.794948	-0.012398	3.849525
C	0.606486	0.920785	4.564836
H	-1.142167	0.208401	3.534036
H	2.491100	1.256346	5.547070
H	0.020787	1.504013	5.274502
H	3.860983	-1.280423	2.016751
O	0.697140	0.605682	-2.204112
O	1.516819	-1.498968	-2.250117
C	1.489908	-0.302559	-1.885786
O	2.503148	0.037703	-0.987180
Cs	4.429982	-2.480132	-1.231716
Cs	-2.107720	-0.360972	-1.293008
O	-4.621717	-1.398783	0.572716
C	-5.240633	-2.251605	-0.072800
C	-5.513588	-2.059747	-1.543960
H	-6.573949	-2.208820	-1.785817
H	-5.229904	-1.035456	-1.813274
H	-4.934989	-2.766061	-2.154668
N	-5.712138	-3.389518	0.501540
C	-5.662122	-3.560245	1.938409
H	-5.105349	-4.470975	2.199846
H	-5.174067	-2.692229	2.386994
H	-6.680739	-3.653158	2.343283
C	-6.422039	-4.424926	-0.222154
H	-6.098737	-4.477706	-1.265182
H	-6.203284	-5.393654	0.243828
H	-7.511563	-4.269692	-0.193110
O	-4.662894	1.325956	-1.839033
C	-5.509634	1.990449	-1.233003
C	-6.572814	1.298446	-0.420258
H	-7.431877	1.052496	-1.060796
H	-6.138319	0.362110	-0.052211
H	-6.940346	1.878011	0.432179
N	-5.519920	3.348951	-1.280120
C	-4.454662	4.058889	-1.958222
H	-4.092680	4.873854	-1.314607
H	-3.632281	3.371956	-2.172330
H	-4.810754	4.497631	-2.902394
C	-6.553394	4.193846	-0.716084
H	-7.487420	3.646794	-0.569507
H	-6.238687	4.632458	0.244163
H	-6.758297	5.016201	-1.413972
O	5.602446	0.037492	-2.795439
C	4.649699	0.423943	-3.482762
C	3.930214	-0.538189	-4.395234
H	3.748067	-0.129039	-5.395553
H	4.548709	-1.436824	-4.496294
H	2.960586	-0.830793	-3.960249
N	4.216069	1.710946	-3.462461
C	4.809578	2.649973	-2.528953
H	4.161845	2.769582	-1.644275
H	5.792896	2.285134	-2.223443
H	4.922311	3.625985	-3.021732
C	2.942650	2.144845	-4.008016
H	2.651567	1.546016	-4.875167
H	2.147166	2.066573	-3.249421
H	3.034200	3.188685	-4.336707

O	5.917562	-1.106350	1.237633
C	5.799399	0.085739	1.554100
C	5.306651	1.097277	0.558787
H	5.881632	2.031415	0.587329
H	5.383949	0.664822	-0.446426
H	4.250671	1.354040	0.742116
N	6.070198	0.519035	2.812969
C	6.638777	-0.386201	3.790847
H	5.962704	-0.503029	4.650511
H	6.802722	-1.360592	3.326460
H	7.596033	0.009136	4.159212
C	5.885567	1.886766	3.259587
H	5.090327	2.386454	2.699032
H	5.588184	1.874156	4.316321
H	6.812541	2.473069	3.171397

***

c3$_{\mathit{ortho}}$

|	Charge	0
|	Electronic Energy (a.u.)	-2146.87317207

C	0.990835	-0.435847	4.358328
H	1.753682	0.089156	4.932498
C	0.414565	-1.591455	4.817706
H	0.716416	-2.012582	5.777445
C	0.633317	0.117841	3.077065
C	-0.558735	-2.244434	4.042784
H	-0.998723	-3.184956	4.365316
C	-0.916351	-1.664468	2.834320
C	-1.934251	-2.315217	1.972081
C	-2.992206	-3.048565	2.518748
H	-3.097056	-3.135916	3.598194
C	-3.923263	-3.632065	1.670359
H	-4.758221	-4.198628	2.078971
C	-3.780802	-3.466773	0.296595
H	-4.489006	-3.897925	-0.407813
C	-2.705774	-2.715715	-0.161318
H	-2.568025	-2.546456	-1.231906
C	-1.767139	2.152736	-0.538500
C	-3.107000	2.264173	-0.041010
C	-4.123036	1.465543	-0.510197
C	-3.858280	0.479398	-1.488243
C	-2.582407	0.318721	-1.985507
C	-1.519214	1.136157	-1.518856
N	-0.366611	-0.522677	2.365678
N	-1.800679	-2.153337	0.643862
O	1.198844	1.131246	2.604834
Pd	-0.987283	0.279114	0.378856
C	-4.982327	-0.382632	-1.936762
F	-5.934030	0.317386	-2.583793
F	-4.595904	-1.366210	-2.761352
F	-5.609025	-0.970645	-0.898491
H	-0.590045	1.156733	-2.095838
H	-2.379496	-0.422850	-2.756986
H	-5.138137	1.593851	-0.134532
H	-3.327407	3.043946	0.687766
C	-0.264557	3.896082	-1.497103
C	-0.503359	3.813619	0.910126

C	0.920288	5.483494	-0.141945
C	0.605710	4.978800	-1.402574
H	-0.528794	3.503324	-2.479842
C	-0.830437	3.295578	-0.366453
C	0.372448	4.904557	0.996648
H	0.627320	5.287893	1.986066
H	1.022260	5.427758	-2.302868
Cs	2.437310	1.712752	-0.150416
O	4.654012	-0.273607	0.784388
C	4.643236	-1.396571	0.275873
C	5.811772	-1.885262	-0.540224
H	5.529816	-2.071539	-1.584666
H	6.587306	-1.114486	-0.520995
H	6.226214	-2.819388	-0.139562
N	3.593888	-2.251423	0.429585
C	2.509132	-1.913244	1.328988
H	2.647553	-0.896908	1.707048
H	1.541295	-1.974893	0.809368
H	2.485685	-2.611666	2.180514
C	3.571493	-3.609239	-0.075514
H	4.166231	-3.712906	-0.987703
H	3.943971	-4.325839	0.673887
H	2.535865	-3.879686	-0.319965
O	3.006102	-0.385050	-2.396318
C	2.959810	-1.501603	-2.916969
C	4.052262	-1.966171	-3.847060
H	3.658712	-2.177100	-4.850390
H	4.799656	-1.170888	-3.921037
H	4.542616	-2.881806	-3.490848
N	1.933233	-2.366867	-2.687852
C	0.777006	-1.929582	-1.931778
H	0.445685	-2.733167	-1.259415
H	1.029011	-1.046084	-1.335082
H	-0.059024	-1.678440	-2.605043
C	1.767903	-3.626060	-3.387039
H	2.730879	-4.064551	-3.661059
H	1.254003	-4.335484	-2.726158
H	1.160510	-3.507784	-4.298007
N	-1.043486	3.259076	2.066967
H	-0.451786	3.430357	2.876326
H	-1.172052	2.246931	1.969386
H	1.596805	6.331670	-0.041161

***

(Cs 2DMA)HCO_3

| Charge 0
| Electronic Energy (a.u.) -859.521978176

H	-0.915646	1.335057	1.758261
O	-2.705726	0.673773	2.858260
O	-2.412474	-1.260010	1.708741
C	-2.150033	-0.094536	2.057620
O	-1.007519	0.436603	1.405024
Cs	-0.091674	-2.017744	-0.288597
O	-0.961216	0.643137	-1.974557
C	-2.054832	0.883814	-1.456037
C	-3.121930	-0.177453	-1.387142
H	-4.111892	0.184270	-1.688085

H	-2.834638	-0.995855	-2.056253
H	-3.193092	-0.570134	-0.359394
N	-2.357548	2.107091	-0.936146
C	-1.319384	3.112829	-0.865741
H	-0.849003	3.132162	0.131447
H	-0.552997	2.899049	-1.614057
H	-1.752403	4.103039	-1.057761
C	-3.490721	2.343144	-0.060043
H	-4.364154	1.761668	-0.367266
H	-3.251071	2.088059	0.986151
H	-3.762848	3.404616	-0.112639
O	2.724911	-0.644624	-0.457138
C	2.986453	0.508983	-0.108821
C	1.919179	1.574141	-0.091182
H	2.208829	2.449638	-0.687086
H	0.995245	1.157068	-0.507689
H	1.721730	1.922555	0.931586
N	4.239337	0.874409	0.280590
C	5.327100	-0.078440	0.214966
H	5.775786	-0.217792	1.208759
H	4.946286	-1.034927	-0.148231
H	6.110561	0.284248	-0.466582
C	4.630159	2.213066	0.669784
H	3.766408	2.827450	0.931799
H	5.283295	2.157192	1.551240
H	5.189402	2.713732	-0.135569

***

c4

| Charge 0
| Electronic Energy (a.u.) -1871.4342706

C	-1.479456	2.828636	-3.092111
H	-0.857060	3.448836	-3.736518
C	-2.809535	3.093733	-2.906477
H	-3.278250	3.942577	-3.405685
C	-0.848739	1.700061	-2.453006
C	-3.579707	2.262625	-2.076488
H	-4.641650	2.451064	-1.949829
C	-2.943900	1.197111	-1.454585
C	-3.704693	0.280340	-0.564001
C	-5.030279	0.520984	-0.183278
H	-5.552737	1.413503	-0.517328
C	-5.684425	-0.387247	0.635543
H	-6.715854	-0.207170	0.933083
C	-5.007500	-1.521775	1.072467
H	-5.482004	-2.259782	1.714467
C	-3.691644	-1.686356	0.665430
H	-3.111195	-2.556272	0.979266
N	-1.631205	0.921759	-1.622112
N	-3.059712	-0.817396	-0.129015
O	0.362552	1.436191	-2.627476
Pd	-0.813755	-0.888037	-0.564218
C	1.946067	-1.015357	1.865323
C	2.105898	-1.067092	0.498935
C	1.185578	-1.778781	-0.317309
C	0.093347	-2.464288	0.324881
C	-0.016050	-2.433779	1.754129

C	0.873150	-1.701822	2.495461
H	2.963862	-0.587449	0.022805
H	1.497716	-2.049265	-1.327319
H	-0.818739	-2.985002	2.243282
H	0.761146	-1.644457	3.578536
C	2.855002	-0.227797	2.735773
F	2.209555	0.804498	3.325495
F	3.896756	0.301188	2.080021
F	3.357767	-0.960297	3.745479
I	-0.695857	-4.283817	-0.641300
Cs	1.614126	2.557625	-0.039814
O	-1.261837	3.357593	0.812979
C	-2.351887	3.083020	1.317105
C	-3.557226	3.959539	1.091779
H	-3.864993	4.459165	2.020243
H	-3.288004	4.723294	0.356069
H	-4.420012	3.393189	0.717540
N	-2.503175	1.976512	2.095590
C	-1.325693	1.164353	2.346630
H	-0.870523	0.836965	1.397840
H	-0.571992	1.708844	2.935444
H	-1.628071	0.269863	2.903419
C	-3.668016	1.694379	2.911489
H	-4.530025	2.285455	2.594261
H	-3.935069	0.632180	2.827094
H	-3.468857	1.914898	3.971519
O	4.627369	1.859796	-0.712539
C	5.051451	0.715333	-0.887095
C	6.148831	0.144788	-0.027046
H	7.029249	-0.131497	-0.621565
H	6.441064	0.902655	0.705213
H	5.813188	-0.754644	0.506009
N	4.549394	-0.089043	-1.867087
C	3.436378	0.397840	-2.663008
H	3.457258	-0.088055	-3.645262
H	2.458911	0.193946	-2.198047
H	3.527592	1.479205	-2.801004
C	4.876886	-1.496587	-1.994891
H	5.913090	-1.692905	-1.706718
H	4.217241	-2.130712	-1.379956
H	4.759408	-1.796983	-3.042362

***

5a

| Charge 0

| Electronic Energy (a.u.) -855.196337383

C	-2.109969	-0.201641	-0.048729
C	-2.937689	0.915394	0.215068
C	-4.330659	0.749202	0.168269
C	-4.901599	-0.479764	-0.125238
C	-4.093772	-1.588084	-0.373609
C	-2.714416	-1.433978	-0.327420
C	-0.631892	-0.116962	-0.030381
C	0.056485	0.910724	-0.688659
C	1.445481	0.952648	-0.698237
C	2.170068	-0.039081	-0.046063
C	1.505262	-1.072512	0.614019

C	0.119929	-1.108099	0.619759
H	-4.962270	1.613654	0.377460
H	-5.986703	-0.572477	-0.153800
H	-4.531612	-2.557082	-0.605655
H	-2.069768	-2.288504	-0.536030
H	-0.502560	1.682477	-1.215415
H	1.960537	1.755228	-1.221368
H	2.075503	-1.845156	1.129241
H	-0.394836	-1.908982	1.149215
N	-2.418849	2.173522	0.488587
H	-3.066607	2.787862	0.972209
H	-1.503768	2.187532	0.927473
C	3.661988	-0.024833	-0.014208
F	4.129401	0.101818	1.239586
F	4.180294	-1.169662	-0.489391
F	4.187940	0.973701	-0.731405

***

TS c1$_{ortho}$-c2$_{ortho}$

| Charge | 0 |
| Electronic Energy (a.u.) | -3006.32493446 |

C	-2.293079	-3.596859	3.239147
H	-3.302107	-3.817965	3.581901
C	-1.362920	-4.585752	3.017443
H	-1.629769	-5.629632	3.181094
C	-1.933670	-2.237874	3.028363
C	-0.069860	-4.262372	2.586233
H	0.661757	-5.045179	2.408792
C	0.235091	-2.923957	2.395566
C	1.577697	-2.449671	1.998923
C	2.639404	-3.314806	1.725099
H	2.484360	-4.390589	1.726102
C	3.892814	-2.796974	1.441303
H	4.723540	-3.468784	1.222578
C	4.078648	-1.416185	1.443806
H	5.040432	-0.959891	1.222087
C	2.984935	-0.613246	1.722204
H	3.068939	0.474077	1.724561
C	0.777374	1.823150	1.812790
C	0.914611	2.187636	0.462893
C	1.545609	3.372630	0.089699
C	2.048625	4.226142	1.071491
C	1.927202	3.884182	2.419308
C	1.295764	2.699046	2.781180
N	-0.666866	-1.950965	2.643391
N	1.769469	-1.110603	1.986573
O	-2.766597	-1.284622	3.237503
Pd	-0.011311	0.032140	2.253862
C	2.712352	5.514649	0.733445
F	2.142047	6.557752	1.362823
F	4.008910	5.533201	1.106093
F	2.684825	5.792549	-0.578392
H	1.204669	2.460199	3.842695
H	2.319979	4.555182	3.184904
H	1.635578	3.638099	-0.963751
H	0.497716	1.522147	-0.299115

H	-1.821674	0.457490	0.043942
N	-2.514175	1.195058	0.343511
C	-2.441768	1.772010	1.567346
C	-1.812478	1.107792	2.668483
C	-3.027667	3.047791	1.772132
C	-1.812927	1.778407	3.917926
H	-2.238365	-0.129745	2.878138
C	-2.986890	3.654896	3.010911
H	-3.492527	3.553326	0.922703
C	-2.381667	3.023513	4.111566
H	-1.357101	1.264211	4.768838
H	-3.438566	4.639985	3.133928
H	-2.374882	3.503120	5.089358
H	-2.907902	1.775347	-0.398121
O	0.248618	-2.538291	-0.505213
O	-0.654757	-0.500013	-0.755639
C	-0.787908	-1.790304	-0.508500
O	-1.968510	-2.254365	-0.307074
Cs	-3.485148	-0.668863	-2.400529
Cs	2.273756	-0.680752	-1.721220
O	4.854285	0.975621	-0.900654
C	5.414782	1.471719	-1.883799
C	5.745628	0.630458	-3.091734
H	6.827778	0.594284	-3.276330
H	5.391101	-0.391085	-2.910464
H	5.270362	1.025288	-3.999691
N	5.757198	2.787471	-1.927646
C	5.350565	3.677550	-0.859816
H	4.644067	4.428088	-1.242321
H	4.872101	3.096615	-0.068442
H	6.222271	4.206198	-0.448867
C	6.346343	3.446869	-3.074702
H	6.852263	2.737620	-3.733238
H	5.585569	3.991080	-3.655983
H	7.091530	4.174357	-2.726625
O	4.754457	-2.550461	-2.057388
C	5.686416	-2.979980	-1.370997
C	6.777867	-2.060285	-0.887083
H	7.657918	-2.123432	-1.542721
H	6.389711	-1.036239	-0.920631
H	7.105941	-2.284777	0.135029
N	5.766740	-4.289885	-1.014554
C	4.660193	-5.184596	-1.285345
H	4.409242	-5.743020	-0.371866
H	3.790731	-4.604255	-1.604164
H	4.916798	-5.909122	-2.071762
C	6.876770	-4.875289	-0.288610
H	7.803144	-4.320298	-0.456554
H	6.676994	-4.922781	0.793918
H	7.033683	-5.899302	-0.650533
O	-6.459536	-1.887081	-1.486244
C	-5.917891	-2.959005	-1.191615
C	-6.109618	-4.174769	-2.066224
H	-6.424352	-5.058617	-1.498135
H	-6.875860	-3.938139	-2.810105
H	-5.180973	-4.432127	-2.594142
N	-5.117308	-3.090847	-0.104470
C	-4.880327	-1.961996	0.780865

H	-3.819913	-1.666696	0.712574
H	-5.542790	-1.138894	0.498028
H	-5.095088	-2.255754	1.818709
C	-4.297683	-4.249964	0.203249
H	-4.441273	-5.049787	-0.526852
H	-3.242314	-3.929185	0.194103
H	-4.559550	-4.643061	1.197979
O	-4.113876	2.559805	-1.822911
C	-5.319364	2.619451	-1.539481
C	-5.884756	1.809263	-0.401421
H	-6.643787	1.098588	-0.753916
H	-5.067571	1.248968	0.065655
H	-6.350854	2.445811	0.361310
N	-6.174992	3.409475	-2.234559
C	-5.706174	4.155759	-3.384947
H	-6.012123	5.206786	-3.297371
H	-4.617696	4.096375	-3.437847
H	-6.137565	3.747064	-4.310947
C	-7.610522	3.439627	-2.037547
H	-7.893031	3.060860	-1.053410
H	-7.962411	4.476373	-2.115679
H	-8.125560	2.845058	-2.807530

***Para*-arylation**

\# **TS c1$_{ortho}$-c1$_{para}$**
| Charge 0
| Electronic Energy (a.u.) -1552.19412254

C	5.405064	-1.244159	0.113960
H	6.040833	-2.127942	0.120886
C	5.915746	0.015190	0.232054
H	6.989054	0.172370	0.339481
C	3.984874	-1.473315	-0.018369
C	5.047690	1.121457	0.216108
H	5.447036	2.124744	0.322368
C	3.691345	0.887174	0.080921
C	2.738852	2.015777	0.061507
C	3.157119	3.348714	0.049769
H	4.215334	3.588397	0.024942
C	2.226323	4.373253	0.058448
H	2.556207	5.409669	0.048741
C	0.874020	4.055048	0.078068
H	0.102225	4.820180	0.090581
C	0.513950	2.719757	0.070723
H	-0.533352	2.430578	0.074261
C	-0.992730	0.069602	-0.056111
C	-1.662843	0.397160	-1.240342
C	-3.007887	0.746589	-1.209912
C	-3.692298	0.774362	0.006802
C	-3.027693	0.475960	1.193431
C	-1.678198	0.129612	1.159012
N	3.157498	-0.359988	-0.027069
N	1.414320	1.722132	0.056042
O	3.531681	-2.630055	-0.118928
Pd	0.955051	-0.328956	-0.079820
C	-5.130755	1.165147	-0.002991
F	-5.710124	1.050275	1.198240

F	-5.300744	2.440458	-0.398425
F	-5.847917	0.416263	-0.859243
H	-1.168647	-0.109319	2.093913
H	-3.557814	0.509387	2.143724
H	-3.531180	0.996845	-2.133480
H	-1.137575	0.378601	-2.196094
H	-3.332739	-2.986108	1.820211
N	-2.350740	-3.228232	1.908814
C	-1.593897	-3.013937	0.768258
C	-2.186457	-3.049453	-0.504017
C	-0.209740	-2.810871	0.848566
C	-1.436300	-2.837865	-1.655634
H	-3.261075	-3.225196	-0.576324
C	0.559795	-2.619150	-0.312146
H	0.277752	-2.830314	1.825245
C	-0.065354	-2.614283	-1.575141
H	-1.934601	-2.851018	-2.624405
H	1.655924	-2.684422	-0.244246
H	0.533476	-2.484083	-2.475754
H	-1.948890	-2.850892	2.761640

***

c1*para*

| Charge 0
| Electronic Energy (a.u.) -1552.20451465

C	-5.322172	-1.347720	0.397218
H	-5.918034	-2.247116	0.544266
C	-5.893914	-0.111422	0.312797
H	-6.974421	0.006039	0.395075
C	-3.892641	-1.527391	0.286713
C	-5.084627	1.022109	0.113019
H	-5.536628	2.005875	0.039929
C	-3.717692	0.837262	0.017375
C	-2.814392	1.983105	-0.213925
C	-3.277482	3.291318	-0.374324
H	-4.339718	3.507002	-0.315786
C	-2.383880	4.321294	-0.613989
H	-2.746653	5.339482	-0.736978
C	-1.027005	4.033673	-0.699945
H	-0.288009	4.805972	-0.895282
C	-0.623450	2.721832	-0.526452
H	0.428243	2.451542	-0.579636
C	0.997294	0.149836	0.108498
C	1.563833	0.708771	1.262277
C	2.909166	1.053220	1.295111
C	3.704959	0.852452	0.164659
C	3.148162	0.328177	-0.998559
C	1.797567	-0.016853	-1.024253
N	-3.126521	-0.383624	0.110551
N	-1.485842	1.722445	-0.282890
O	-3.368040	-2.655024	0.345897
Pd	-0.947631	-0.285659	0.093945
C	5.142196	1.239679	0.237798
F	5.819617	0.949589	-0.879503
F	5.296109	2.558852	0.456837
F	5.776792	0.623994	1.251601
H	1.381403	-0.440104	-1.939783

H	3.764063	0.186244	-1.884913
H	3.345937	1.476070	2.200729
H	0.953104	0.865236	2.153225
C	1.934301	-2.886955	0.704090
C	0.647481	-3.139911	-1.355202
C	-0.499363	-2.785715	-0.667430
C	-0.454412	-2.411233	0.700595
H	2.896155	-2.939273	1.215400
H	-1.477650	-2.837016	-1.143649
H	-1.389477	-2.453110	1.262926
C	1.883444	-3.187846	-0.682039
C	0.798159	-2.508981	1.370115
H	0.849535	-2.283569	2.435297
H	0.610530	-3.401729	-2.412683
N	3.027402	-3.477340	-1.352106
H	2.965290	-3.886855	-2.276811
H	3.860516	-3.708675	-0.824124

***

c1NH*para*

| Charge 0
| Electronic Energy (a.u.) -3006.3588275

C	-8.928441	0.139330	-2.881554
H	-9.228844	0.135953	-3.928361
C	-9.836617	0.016443	-1.870306
H	-10.899478	-0.090530	-2.088520
C	-7.512712	0.262977	-2.616282
C	-9.395864	0.022923	-0.535015
H	-10.107837	-0.099976	0.274846
C	-8.039758	0.164048	-0.295487
C	-7.529634	0.177293	1.093736
C	-8.372389	0.289358	2.203615
H	-9.444852	0.395617	2.070539
C	-7.838178	0.285950	3.481782
H	-8.492842	0.374051	4.346407
C	-6.461389	0.179264	3.638676
H	-5.994261	0.176994	4.620009
C	-5.679176	0.089247	2.499833
H	-4.595347	0.019303	2.573790
C	-3.300766	-0.036874	0.419051
C	-3.036274	-1.311360	0.953642
C	-2.032149	-1.503495	1.898287
C	-1.258602	-0.418750	2.318845
C	-1.462206	0.842470	1.758676
C	-2.469942	1.019787	0.812479
N	-7.115296	0.283441	-1.284241
N	-6.190996	0.084886	1.260972
O	-6.687720	0.347601	-3.544628
Pd	-5.023106	0.228996	-0.561104
C	-0.160539	-0.659210	3.295504
F	0.324381	0.461888	3.831078
F	-0.554314	-1.446741	4.315331
F	0.884776	-1.305423	2.727524
H	-2.605201	2.014594	0.379474
H	-0.811792	1.673406	2.025806
H	-1.855801	-2.494855	2.320328
H	-3.657079	-2.164031	0.669014

H	0.013554	3.063988	-2.431715
N	-0.894398	3.545105	-2.395567
C	-1.833039	2.629280	-2.423012
C	-1.576355	1.200633	-2.531878
C	-3.230794	3.008766	-2.375478
C	-2.576175	0.282933	-2.512631
H	-0.531563	0.901840	-2.633308
C	-4.225406	2.080373	-2.427189
H	-3.453140	4.077247	-2.358454
C	-3.970719	0.655091	-2.404220
H	-2.329677	-0.779650	-2.599023
H	-5.266841	2.397843	-2.500300
H	-4.690342	0.063163	-2.981291
H	-0.224836	3.533787	-0.547076
O	0.131549	3.255000	0.325104
O	1.379001	2.044164	-1.108445
C	1.070398	2.276899	0.089799
O	1.521923	1.717275	1.111401
Cs	0.688213	-1.050521	-0.559622
Cs	4.404870	1.252197	-0.102114
O	1.257837	-3.825411	0.766244
C	0.338050	-4.296023	1.441008
C	0.511557	-4.548211	2.915980
H	0.317023	-5.596172	3.176699
H	1.542414	-4.300266	3.183693
H	-0.164659	-3.925603	3.516429
N	-0.868521	-4.604516	0.890334
C	-1.084679	-4.434709	-0.531798
H	-1.761134	-3.586661	-0.728709
H	-0.128475	-4.260105	-1.032806
H	-1.545156	-5.340949	-0.948094
C	-1.992881	-5.169424	1.609091
H	-1.861556	-5.093299	2.690340
H	-2.910211	-4.626600	1.339485
H	-2.134622	-6.227870	1.343901
O	3.599120	-1.352745	-1.677972
C	4.215354	-2.362637	-2.040425
C	3.639027	-3.740822	-1.842299
H	4.271152	-4.363477	-1.195395
H	2.656440	-3.653380	-1.366356
H	3.532619	-4.268663	-2.799231
N	5.421914	-2.263868	-2.649413
C	5.941764	-0.945855	-2.972704
H	6.613331	-1.031515	-3.834627
H	5.119660	-0.270598	-3.231860
H	6.501645	-0.515957	-2.128514
C	6.237382	-3.391242	-3.056860
H	5.944317	-4.306078	-2.535929
H	6.168318	-3.563601	-4.141258
H	7.286246	-3.184290	-2.806443
O	5.238472	3.817930	1.542812
C	4.598095	4.834154	1.258802
C	3.109209	4.769013	1.033570
H	2.865985	4.896147	-0.031049
H	2.744229	3.786956	1.354569
H	2.566500	5.543759	1.588826
N	5.198635	6.047893	1.120645
C	6.607790	6.199987	1.415353

H	6.750185	6.943883	2.212783
H	7.012737	5.239660	1.740489
H	7.154203	6.542708	0.525157
C	4.502720	7.276548	0.796841
H	3.568276	7.082338	0.265206
H	4.283216	7.866565	1.700096
H	5.139428	7.884267	0.140786
O	6.993569	-0.519577	0.185993
C	7.048674	-1.749370	0.278043
C	5.991527	-2.514553	1.032156
H	6.415589	-3.002612	1.920074
H	5.216290	-1.812153	1.355464
H	5.527817	-3.299307	0.421513
N	8.053339	-2.470209	-0.291998
C	9.163386	-1.786587	-0.925283
H	9.319343	-2.175076	-1.941672
H	8.949971	-0.716922	-0.973460
H	10.088973	-1.944571	-0.351604
C	8.251766	-3.891726	-0.088716
H	7.309714	-4.407492	0.113349
H	8.685426	-4.326582	-0.998543
H	8.945839	-4.083245	0.744397

***

TS c1NH$_{para}$-c2NH$_{para}$

| Charge 0
| Electronic Energy (a.u.) -3006.3295284

C	-8.150742	0.440890	-3.460674
H	-8.176086	0.619054	-4.533079
C	-9.271958	0.093820	-2.747717
H	-10.230365	-0.012298	-3.253525
C	-6.909179	0.566322	-2.786448
C	-9.185938	-0.135835	-1.368177
H	-10.066505	-0.437276	-0.809243
C	-7.954170	0.011395	-0.754469
C	-7.756021	-0.224764	0.692424
C	-8.822026	-0.414690	1.571866
H	-9.846937	-0.375919	1.213438
C	-8.569367	-0.637817	2.917682
H	-9.395452	-0.783399	3.610563
C	-7.254262	-0.664026	3.363868
H	-7.008223	-0.831878	4.409128
C	-6.242894	-0.457882	2.437450
H	-5.195821	-0.462198	2.738358
C	-3.321544	0.214641	0.781215
C	-3.087784	-1.017256	1.425110
C	-2.031183	-1.194888	2.314078
C	-1.165106	-0.133714	2.583660
C	-1.344881	1.088732	1.935766
C	-2.407979	1.248143	1.048935
N	-6.845852	0.365641	-1.451388
N	-6.478288	-0.246558	1.138039
O	-5.839559	0.867259	-3.444660
Pd	-4.985257	0.377658	-0.343749
C	-0.010846	-0.356959	3.497976
F	0.533096	0.778039	3.943107

F	-0.357434	-1.080436	4.580554
F	0.983656	-1.056114	2.904695
H	-2.510407	2.208922	0.539771
H	-0.623855	1.892760	2.079951
H	-1.883561	-2.159554	2.802804
H	-3.760771	-1.860797	1.249480
H	0.339458	3.161210	-2.853892
N	-0.471539	3.754417	-2.648419
C	-1.504884	2.952076	-2.438310
C	-1.476301	1.514335	-2.569679
C	-2.776377	3.496656	-2.030563
C	-2.557077	0.730953	-2.245217
H	-0.548190	1.062828	-2.933977
C	-3.846066	2.691771	-1.734115
H	-2.857384	4.585012	-1.971605
C	-3.814094	1.262701	-1.828369
H	-2.453439	-0.357326	-2.330364
H	-4.788889	3.167284	-1.440726
H	-4.898067	0.963183	-2.717293
H	0.189808	3.734783	-0.862687
O	0.433870	3.487596	0.064192
O	1.720728	2.116338	-1.169057
C	1.304770	2.431668	-0.024752
O	1.596711	1.884374	1.065047
Cs	0.452710	-0.790414	-0.499742
Cs	4.452457	0.983770	-0.020809
O	1.021738	-3.551716	0.870083
C	0.129274	-3.989461	1.601006
C	0.384416	-4.243580	3.064340
H	0.150083	-5.277198	3.348197
H	1.442794	-4.050712	3.260654
H	-0.213120	-3.578836	3.702778
N	-1.116397	-4.261615	1.123170
C	-1.404187	-4.094931	-0.286777
H	-2.013136	-3.193015	-0.466049
H	-0.469627	-4.016525	-0.849116
H	-1.968066	-4.963258	-0.652767
C	-2.220170	-4.769520	1.912547
H	-2.025110	-4.684109	2.983597
H	-3.128025	-4.192213	1.685640
H	-2.420176	-5.824764	1.673267
O	3.240494	-1.419861	-1.733937
C	3.755200	-2.464794	-2.148730
C	3.075076	-3.794671	-1.951456
H	3.686864	-4.489489	-1.361308
H	2.136124	-3.631757	-1.411739
H	2.865470	-4.280789	-2.913608
N	4.936710	-2.453016	-2.815753
C	5.548212	-1.182111	-3.163590
H	6.022406	-1.271105	-4.148995
H	4.781574	-0.402902	-3.209491
H	6.310891	-0.889662	-2.426110
C	5.611774	-3.635247	-3.314327
H	5.305872	-4.532902	-2.770500
H	5.415727	-3.786722	-4.386774
H	6.694183	-3.512417	-3.176886

O	5.488908	3.432457	1.710973
C	5.009259	4.526089	1.399113
C	3.547883	4.658148	1.056012
H	3.410346	4.816498	-0.023307
H	3.031192	3.732469	1.335042
H	3.069555	5.498279	1.574239
N	5.771190	5.652862	1.335738
C	7.158505	5.616471	1.747502
H	7.329216	6.328085	2.568544
H	7.406282	4.608290	2.085343
H	7.817642	5.892468	0.912031
C	5.269713	6.964868	0.981229
H	4.367616	6.899307	0.368297
H	5.050299	7.569130	1.875193
H	6.033828	7.491515	0.394592
O	6.864218	-1.041654	-0.125573
C	6.860773	-2.274872	-0.074548
C	5.801202	-3.011132	0.705370
H	6.238858	-3.557795	1.551471
H	5.085918	-2.279357	1.095139
H	5.260789	-3.741053	0.089229
N	7.801438	-3.022976	-0.714544
C	8.902322	-2.373128	-1.397135
H	8.966534	-2.723531	-2.437406
H	8.748607	-1.292270	-1.385973
H	9.854241	-2.607860	-0.897468
C	7.933701	-4.460617	-0.592750
H	7.003535	-4.927646	-0.261744
H	8.196602	-4.884796	-1.571384
H	8.732768	-4.724343	0.117301

***

c2NH*para*

| Charge 0
| Electronic Energy (a.u.) -3006.3325533

C	-4.826210	-4.757597	0.311528
H	-4.032409	-5.482349	0.148780
C	-6.031994	-5.089413	0.889045
H	-6.222378	-6.111446	1.209968
C	-4.631921	-3.426494	-0.084942
C	-7.016752	-4.112941	1.056354
H	-7.971384	-4.380778	1.497744
C	-6.749142	-2.815034	0.648771
C	-7.744751	-1.725091	0.759447
C	-9.020320	-1.926140	1.286457
H	-9.324237	-2.901816	1.654508
C	-9.912782	-0.864584	1.339201
H	-10.910302	-1.012284	1.747511
C	-9.516574	0.379019	0.864814
H	-10.183481	1.236996	0.883863
C	-8.231146	0.508758	0.359395
H	-7.868123	1.464183	-0.019074
C	-5.251258	1.646408	-0.710781
C	-5.162162	2.572220	0.344299
C	-5.298432	3.937553	0.123873
C	-5.520828	4.415720	-1.168657

C	-5.601623	3.522775	-2.233614
C	-5.460602	2.156310	-2.001402
N	-5.553747	-2.479684	0.100348
N	-7.367220	-0.510011	0.306640
O	-3.484528	-3.106155	-0.666996
Pd	-5.280793	-0.317458	-0.347766
C	-5.675795	5.883799	-1.359963
F	-5.826694	6.233068	-2.643510
F	-6.744173	6.366955	-0.696769
F	-4.616588	6.567991	-0.889637
H	-5.517428	1.477040	-2.854034
H	-5.769590	3.890766	-3.244623
H	-5.231510	4.639180	0.956646
H	-4.990033	2.222886	1.364375
H	1.381980	0.444164	-0.793021
N	0.882567	0.120047	-1.627365
C	-2.355393	0.183871	0.194819
C	-3.289589	-0.183520	-0.797444
C	-0.995537	0.290480	-0.055480
C	-2.749477	-0.372438	-2.088295
H	-3.434205	-2.114425	-0.783601
C	-0.427111	0.052806	-1.350878
H	-0.315299	0.565872	0.759422
C	-1.389170	-0.284873	-2.356257
H	-3.419481	-0.627732	-2.916668
H	-1.021206	-0.464110	-3.370629
H	2.284406	0.811657	-2.555148
O	3.118331	1.209176	-2.929253
O	3.698018	0.870110	-0.786834
C	4.033499	1.313974	-1.917519
O	5.126645	1.840537	-2.225313
Cs	6.636078	0.235163	0.024842
Cs	2.968376	-2.129255	-0.387323
O	1.105373	-2.366036	2.165619
C	-0.099014	-2.566911	1.979116
C	-0.572243	-3.420406	0.832219
H	-0.926174	-2.778428	0.011331
H	0.270251	-4.018689	0.470542
H	-1.396411	-4.091669	1.100457
N	-1.055954	-2.019935	2.773051
C	-0.671142	-1.213800	3.913356
H	-1.194468	-1.561347	4.814363
H	0.407822	-1.305393	4.063953
H	-0.926371	-0.154897	3.751395
C	-2.478370	-2.235461	2.572055
H	-2.741730	-2.173186	1.510809
H	-2.810173	-3.206022	2.972331
H	-3.029711	-1.444312	3.093509
O	4.460927	-0.809292	2.244205
C	3.592472	-0.232843	2.909510
C	2.545519	0.615650	2.236836
H	2.398988	1.585061	2.728600
H	2.859791	0.784163	1.198855
H	1.585071	0.077453	2.245713
N	3.530684	-0.358588	4.263948
C	4.548237	-1.107380	4.970482
H	5.216795	-1.577318	4.246657
H	5.131275	-0.442296	5.625462

H	4.084116	-1.882572	5.596159
C	2.592526	0.340777	5.117843
H	1.708286	0.667353	4.565825
H	2.259548	-0.335721	5.916605
H	3.060841	1.218521	5.590407
O	7.317975	2.907033	1.534036
C	6.897206	4.055089	1.704840
C	7.794914	5.138200	2.247115
H	7.384363	5.598319	3.154641
H	8.763174	4.688935	2.485219
H	7.951333	5.937598	1.511129
N	5.610555	4.375894	1.417713
C	4.753154	3.327989	0.885657
H	5.015535	3.052560	-0.146913
H	4.819983	2.426634	1.507905
H	3.715536	3.680169	0.899138
C	5.040504	5.705182	1.489944
H	5.766167	6.431524	1.861607
H	4.706172	6.033788	0.495020
H	4.170273	5.713024	2.161581
O	5.647626	-1.949076	-2.052726
C	5.484971	-2.028171	-3.276219
C	5.666609	-0.826064	-4.162290
H	4.833720	-0.699792	-4.863944
H	5.723201	0.069624	-3.531060
H	6.587625	-0.920584	-4.754657
N	5.152194	-3.203749	-3.875391
C	4.871265	-4.376708	-3.072805
H	3.797006	-4.617168	-3.094680
H	5.423363	-5.241251	-3.464635
H	5.179964	-4.192853	-2.041273
C	4.872579	-3.340810	-5.291549
H	5.443850	-2.622275	-5.884349
H	5.165321	-4.347503	-5.613894
H	3.800965	-3.210185	-5.508082
H	-2.703237	0.389343	1.213397

***

c2*para*

| Charge 0

| Electronic Energy (a.u.) -3006.3403867

C	1.050413	6.035464	1.727863
H	0.284653	6.255420	2.467129
C	1.764639	7.020153	1.081921
H	1.570864	8.068579	1.298455
C	1.332784	4.699636	1.408186
C	2.752212	6.671078	0.158155
H	3.331663	7.447132	-0.331946
C	2.990405	5.329027	-0.092344
C	4.062900	4.872953	-1.003373
C	4.811980	5.755437	-1.780828
H	4.606397	6.821889	-1.766149
C	5.827000	5.261376	-2.588127
H	6.416057	5.942196	-3.198815
C	6.076715	3.895444	-2.604345
H	6.863581	3.465517	-3.218396
C	5.284824	3.074013	-1.815298

H	5.433421	1.994890	-1.798934
C	3.659315	0.488321	-0.399233
C	4.786918	0.068165	0.328419
C	5.398052	-1.155093	0.074321
C	4.887815	-1.994621	-0.916811
C	3.775551	-1.600434	-1.657225
C	3.177743	-0.368826	-1.398913
N	2.267277	4.355414	0.520311
N	4.304225	3.544317	-1.037602
O	0.632035	3.750337	2.016961
Pd	2.876535	2.283628	0.025672
C	5.500011	-3.339997	-1.087768
F	5.065336	-3.981596	-2.177622
F	6.840594	-3.282454	-1.171146
F	5.237138	-4.145575	-0.034196
H	2.296506	-0.088550	-1.978444
H	3.355241	-2.262742	-2.413242
H	6.267143	-1.466612	0.655907
H	5.196265	0.702100	1.118016
H	-2.122047	-1.948462	1.712648
N	-1.513252	-1.512946	2.439360
C	1.526532	0.596737	2.185205
C	1.440579	1.113847	0.881351
C	0.558323	-0.261792	2.705126
C	0.331587	0.699926	0.120307
H	0.913763	2.863431	1.673642
C	-0.535321	-0.671233	1.925025
C	-0.635573	-0.172559	0.612883
H	0.223892	1.049937	-0.911384
H	-1.463364	-0.509817	-0.029317
H	-1.122958	-2.202504	3.078916
O	-3.046434	-1.369929	-1.251085
O	-3.244277	-2.678850	0.561711
C	-3.111166	-2.551960	-0.735687
O	-3.027397	-3.604544	-1.463542
Cs	-0.983067	-4.775478	0.495597
Cs	-4.332233	0.205503	1.024849
O	-3.320229	2.622246	-0.801744
C	-3.386817	3.732284	-0.266938
C	-4.620272	4.162845	0.483263
H	-5.032817	5.100935	0.090413
H	-5.380443	3.377363	0.392252
H	-4.400469	4.322700	1.547890
N	-2.351339	4.613362	-0.339441
C	-1.119307	4.161149	-0.960326
H	-0.534662	3.524833	-0.277964
H	-1.345543	3.578363	-1.857852
H	-0.514400	5.033029	-1.237637
C	-2.273223	5.813278	0.470853
H	-3.262696	6.251852	0.625191
H	-1.822002	5.611059	1.455519
H	-1.655317	6.557430	-0.045597
O	-7.056392	1.656699	0.259343
C	-7.391539	1.424708	-0.907505
C	-6.386687	1.503210	-2.028355
H	-6.732088	2.143217	-2.849736
H	-5.448025	1.909719	-1.632784
H	-6.185017	0.507622	-2.447641

N	-8.666418	1.082067	-1.231655
C	-9.695493	1.053183	-0.213510
H	-10.090656	0.034305	-0.092799
H	-9.275664	1.392256	0.735372
H	-10.527445	1.711949	-0.500979
C	-9.129751	0.781095	-2.570405
H	-8.299806	0.573705	-3.248720
H	-9.769737	-0.111133	-2.539094
H	-9.724301	1.611961	-2.979660
O	1.416848	-4.314030	-1.726306
C	0.576632	-4.388164	-2.632067
C	0.267344	-5.717299	-3.278820
H	0.425941	-5.702095	-4.364601
H	0.924698	-6.472672	-2.838295
H	-0.777719	-6.009295	-3.103115
N	-0.123907	-3.313526	-3.065436
C	-0.021034	-2.041410	-2.372374
H	-1.025412	-1.738622	-2.022671
H	0.667197	-2.140795	-1.526958
H	0.369167	-1.269248	-3.055570
C	-1.242005	-3.374526	-3.992744
H	-1.188762	-4.261639	-4.629264
H	-2.173321	-3.371248	-3.397833
H	-1.206627	-2.492053	-4.647716
O	0.716244	-3.617154	2.905329
C	1.829493	-3.134738	2.668175
C	2.279583	-2.850194	1.259155
H	2.996998	-3.607200	0.913898
H	1.415829	-2.875498	0.587981
H	2.759636	-1.867283	1.165699
N	2.719064	-2.856906	3.658608
C	2.321590	-2.965383	5.046326
H	2.452820	-1.997781	5.552924
H	1.271261	-3.260567	5.096591
H	2.933355	-3.713187	5.570561
C	4.042829	-2.314432	3.424728
H	4.466293	-2.678704	2.483379
H	4.039317	-1.212381	3.411894
H	4.704240	-2.643568	4.235477
H	2.373412	0.869551	2.822189
H	0.648748	-0.635615	3.728442

***

c2b*para*
| Charge 0
| Electronic Energy (a.u.) -3006.39199009

C	-0.841457	5.023378	1.802671
H	-1.856924	4.955657	2.192858
C	-0.186970	6.222960	1.699903
H	-0.673447	7.143858	2.022077
C	-0.228623	3.808959	1.335342
C	1.102159	6.275149	1.143890
H	1.595902	7.229503	0.985826
C	1.695411	5.078349	0.777444
C	3.011057	5.042408	0.098263
C	3.844076	6.157682	-0.012792
H	3.577744	7.094772	0.468774

C	5.024947	6.061318	-0.733767
H	5.679677	6.925712	-0.825226
C	5.358142	4.852095	-1.334299
H	6.268435	4.734892	-1.916718
C	4.500425	3.777152	-1.158589
H	4.726543	2.800002	-1.584871
C	3.418223	0.650861	-0.350508
C	4.688852	0.273843	0.110957
C	5.402490	-0.771333	-0.473181
C	4.854783	-1.463391	-1.549499
C	3.606564	-1.089315	-2.053845
C	2.900983	-0.050168	-1.459244
N	1.086506	3.878814	0.936906
N	3.362430	3.861780	-0.460040
O	-0.859518	2.722463	1.269947
Pd	2.314694	2.140088	0.414627
C	5.530110	-2.645882	-2.146012
F	4.767036	-3.755178	-2.049885
F	5.775918	-2.488802	-3.459985
F	6.699521	-2.933327	-1.560902
H	1.916403	0.213057	-1.861925
H	3.182486	-1.628271	-2.904210
H	6.376204	-1.058277	-0.078098
H	5.133325	0.789085	0.965766
C	0.521274	-0.147600	1.291360
C	2.290465	0.325240	2.808319
C	0.734506	-1.470372	3.322602
C	0.082274	-1.181938	2.116480
H	-0.056009	0.063287	0.389774
H	3.180909	0.884319	3.109096
C	1.636571	0.639613	1.607709
H	-0.583840	2.094429	-0.541919
C	1.847320	-0.693194	3.659718
H	2.373407	-0.893978	4.596428
H	-0.792278	-1.776222	1.831468
N	0.321417	-2.545655	4.122382
H	0.558813	-2.434115	5.104132
H	-0.667461	-2.761096	4.027129
Cs	-0.065337	-2.591598	-1.548147
O	-0.420527	0.318886	-2.988258
C	-0.858396	0.748785	-1.903039
O	-0.307057	1.938786	-1.474958
O	-1.719283	0.222671	-1.149962
Cs	-3.147993	0.662814	1.524256
O	-4.850434	3.256796	1.107836
C	-5.279527	3.693298	0.037804
C	-6.748741	3.976853	-0.148279
H	-7.184738	3.319004	-0.912795
H	-7.255552	3.790141	0.802662
H	-6.937596	5.013188	-0.455066
N	-4.464563	3.935915	-1.024749
C	-3.043901	3.668943	-0.922799
H	-2.764132	3.490965	0.118159
H	-2.763548	2.782977	-1.513286
H	-2.472287	4.529181	-1.301344
C	-4.889847	4.544268	-2.269244
H	-5.959143	4.404919	-2.447421
H	-4.666731	5.622507	-2.282358

H	-4.348233	4.077594	-3.102874
O	-5.207021	0.170180	-0.790271
C	-4.715475	-0.054497	-1.898582
C	-4.708387	0.996725	-2.974979
H	-5.224067	0.660002	-3.883812
H	-5.215471	1.883592	-2.582377
H	-3.679573	1.261572	-3.256956
N	-4.189914	-1.268868	-2.220349
C	-4.001007	-2.219788	-1.145091
H	-3.857513	-3.219904	-1.568319
H	-3.111308	-1.961208	-0.543774
H	-4.877113	-2.224192	-0.487530
C	-3.374599	-1.455472	-3.410187
H	-3.883565	-1.063103	-4.297912
H	-2.393015	-0.962909	-3.309302
H	-3.227856	-2.530573	-3.569873
O	1.306384	-4.436455	0.551854
C	1.797669	-4.640428	1.666304
C	1.191732	-5.661438	2.594783
H	0.724086	-5.167919	3.458117
H	0.423445	-6.209303	2.040977
H	1.932915	-6.375742	2.973581
N	2.892138	-3.963229	2.102052
C	3.556043	-3.013086	1.234405
H	4.607165	-3.305601	1.085654
H	3.051540	-2.988127	0.265292
H	3.535567	-2.007296	1.682041
C	3.505836	-4.119424	3.403428
H	2.871962	-4.689783	4.085309
H	4.481163	-4.622576	3.320467
H	3.672257	-3.126639	3.846970
O	-2.060231	-5.015353	-0.815202
C	-2.708527	-4.875232	0.224096
C	-2.075458	-4.268721	1.453465
H	-2.186642	-4.909022	2.338227
H	-1.006575	-4.134208	1.249560
H	-2.530477	-3.295588	1.692244
N	-4.017723	-5.241431	0.310399
C	-4.683595	-5.842361	-0.825648
H	-5.495692	-5.191655	-1.184608
H	-3.961267	-5.996146	-1.629835
H	-5.121956	-6.808441	-0.537714
C	-4.872741	-5.032995	1.459251
H	-4.349497	-4.524337	2.270704
H	-5.740953	-4.420637	1.172208
H	-5.249024	-5.995063	1.836931

***

TS c2b-c3$_{para}$HCO$_3$

| Charge 0

| Electronic Energy (a.u.) -3006.37488628

C	0.372114	5.121406	1.421236
H	-0.667578	5.305155	1.692048
C	1.279085	6.143918	1.300106
H	0.972578	7.174408	1.482530
C	0.754601	3.764859	1.140644
C	2.600909	5.868515	0.916203

H	3.312736	6.674017	0.757551
C	2.947627	4.540345	0.714141
C	4.308157	4.161588	0.260052
C	5.399440	5.031389	0.333205
H	5.290600	6.015511	0.782276
C	6.631853	4.621367	-0.154136
H	7.489716	5.289179	-0.101378
C	6.755885	3.349202	-0.703573
H	7.701149	2.989280	-1.102317
C	5.633378	2.534055	-0.719507
H	5.680744	1.520165	-1.121017
C	3.273731	-0.466226	-0.049582
C	4.486449	-1.092816	0.321802
C	5.048662	-2.112280	-0.433151
C	4.423261	-2.544584	-1.604224
C	3.238143	-1.925346	-2.016761
C	2.679320	-0.908759	-1.259974
N	2.076805	3.520872	0.861617
N	4.443156	2.921548	-0.250819
O	-0.083899	2.821255	1.136388
Pd	2.854126	1.439173	0.462026
C	4.927457	-3.715620	-2.361923
F	4.211764	-4.833725	-2.101957
F	4.849749	-3.540053	-3.693264
F	6.203626	-4.012361	-2.079025
H	1.749834	-0.447029	-1.604128
H	2.752537	-2.245876	-2.940133
H	5.979574	-2.576825	-0.109678
H	4.988534	-0.787971	1.241552
C	0.670949	-0.559496	1.097093
C	2.483181	-0.603420	2.648977
C	0.373842	-1.645711	3.249916
C	-0.123014	-1.257580	1.995625
H	0.216532	-0.247820	0.157227
H	3.514626	-0.379047	2.928308
C	2.003428	-0.215231	1.384190
H	0.000213	2.040354	-0.588213
C	1.691350	-1.289350	3.567353
H	2.098172	-1.568395	4.541682
H	-1.153394	-1.511352	1.729653
N	-0.398168	-2.418636	4.115683
H	-0.136775	-2.337859	5.093953
H	-1.400550	-2.300893	3.997979
Cs	-0.935481	-2.621143	-1.644338
O	-0.467003	0.314476	-3.039281
C	-0.685368	0.806166	-1.914252
O	0.174805	1.812656	-1.532822
O	-1.573092	0.474359	-1.084550
Cs	-2.797635	1.412988	1.534593
O	-3.588257	4.411035	1.049049
C	-3.940912	4.973248	0.010315
C	-5.236362	5.743310	-0.048383
H	-5.940274	5.279523	-0.753431
H	-5.684353	5.734123	0.949187
H	-5.089355	6.784171	-0.362882
N	-3.199549	4.926854	-1.130228
C	-1.954484	4.186098	-1.159326
H	-1.674640	3.869008	-0.151700

H	-2.046485	3.293247	-1.796201
H	-1.150468	4.816104	-1.568311
C	-3.507668	5.660828	-2.341295
H	-4.574834	5.882349	-2.425352
H	-2.945334	6.606395	-2.389544
H	-3.223077	5.054893	-3.211695
O	-4.993493	1.580876	-0.720175
C	-4.645784	1.222524	-1.847468
C	-4.435792	2.221995	-2.953284
H	-5.091524	2.026774	-3.811973
H	-4.652488	3.217678	-2.553449
H	-3.399200	2.195141	-3.318626
N	-4.466565	-0.089727	-2.163667
C	-4.472610	-1.048426	-1.078215
H	-4.636532	-2.052661	-1.485197
H	-3.506216	-1.034110	-0.544897
H	-5.272267	-0.810182	-0.368402
C	-3.791900	-0.497766	-3.386166
H	-4.207511	0.023981	-4.255302
H	-2.706553	-0.306692	-3.332140
H	-3.964340	-1.569963	-3.540851
O	-0.096050	-4.696879	0.508086
C	0.333670	-4.980099	1.630255
C	-0.537019	-5.706151	2.623557
H	-0.784802	-5.054314	3.473158
H	-1.462102	-5.994689	2.115533
H	-0.054198	-6.608039	3.019558
N	1.592519	-4.648395	2.021802
C	2.503047	-4.015224	1.089995
H	3.365437	-4.670287	0.891790
H	1.981182	-3.813101	0.150636
H	2.875722	-3.068901	1.509990
C	2.171252	-4.963992	3.310671
H	1.409328	-5.256449	4.035995
H	2.908835	-5.776465	3.223667
H	2.687817	-4.075955	3.702305
O	-3.568382	-4.306933	-0.635260
C	-4.075464	-3.872316	0.400877
C	-3.211437	-3.403632	1.547060
H	-3.465205	-3.906440	2.489615
H	-2.168635	-3.631991	1.296889
H	-3.322045	-2.321074	1.711747
N	-5.425390	-3.779001	0.557579
C	-6.313061	-4.222396	-0.496040
H	-6.890757	-3.374463	-0.894764
H	-5.725521	-4.669978	-1.300135
H	-7.022549	-4.964961	-0.104102
C	-6.098870	-3.212564	1.706355
H	-5.393358	-2.804639	2.432489
H	-6.766073	-2.399560	1.382321
H	-6.714553	-3.973920	2.207690

***

c3$_{para}$-HCO$_3$

| Charge 0
| Electronic Energy (a.u.) -3006.42286433

C	1.899146	4.670952	1.228069

H	0.964817	5.181379	1.462821
C	3.102828	5.328004	1.189546
H	3.153116	6.396720	1.400439
C	1.824456	3.266363	0.950265
C	4.273180	4.622530	0.864591
H	5.238375	5.120072	0.801065
C	4.159118	3.271294	0.579980
C	5.367640	2.499884	0.192163
C	6.552008	2.628025	0.921020
H	6.591518	3.291078	1.783457
C	7.659287	1.876602	0.544216
H	8.589440	1.947852	1.105579
C	7.553625	1.029833	-0.552092
H	8.389782	0.416992	-0.881501
C	6.338159	0.983303	-1.229218
H	6.220289	0.338890	-2.103314
C	2.431127	-1.665348	0.754128
C	3.705508	-2.004002	1.310049
C	4.798474	-2.284721	0.513123
C	4.701436	-2.163448	-0.885139
C	3.523943	-1.713090	-1.461307
C	2.386313	-1.457971	-0.661357
N	2.985868	2.602825	0.624979
N	5.265734	1.690902	-0.874251
O	0.733581	2.631604	1.000820
Pd	2.725961	0.457037	0.327607
C	5.859207	-2.573068	-1.722992
F	5.972344	-3.913998	-1.809752
F	5.780272	-2.117131	-2.982364
F	7.033643	-2.143810	-1.224969
H	1.422439	-1.353319	-1.164611
H	3.443624	-1.608318	-2.542066
H	5.739889	-2.589132	0.969207
H	3.796521	-2.106734	2.391198
C	-0.020277	-1.242296	1.216952
C	1.165805	-2.785219	2.609790
C	-1.209121	-2.445904	2.961838
C	-1.193829	-1.525363	1.901230
H	-0.063594	-0.489657	0.425113
H	2.075013	-3.318896	2.887250
C	1.190894	-1.874338	1.543875
H	0.425180	1.853380	-0.624293
C	0.000903	-3.059900	3.313155
H	0.015113	-3.778516	4.134729
H	-2.124953	-1.027754	1.614856
N	-2.393805	-2.791259	3.594308
H	-2.284326	-3.166055	4.531494
H	-3.122263	-2.085405	3.556625
Cs	-1.779224	-2.114222	-1.863451
O	-1.311913	0.956146	-2.936864
C	-0.957989	1.137658	-1.751959
O	0.278842	1.714088	-1.591425
O	-1.583924	0.829672	-0.703403
Cs	-2.123005	2.300009	1.945805
O	-1.941787	5.332044	1.190574
C	-2.379389	5.871974	0.172340
C	-3.472416	6.907604	0.257214
H	-4.370739	6.582733	-0.285448

H	-3.729344	7.047275	1.310996
H	-3.161538	7.872922	-0.162097
N	-1.921736	5.562249	-1.071693
C	-0.881841	4.567829	-1.242690
H	-0.674832	4.060876	-0.296278
H	-1.190372	3.825658	-1.992137
H	0.050948	5.035214	-1.598206
C	-2.263938	6.299800	-2.271148
H	-3.253393	6.759020	-2.199841
H	-1.523546	7.087190	-2.483408
H	-2.275705	5.609644	-3.125011
O	-4.444759	2.967878	-0.017896
C	-4.433886	2.372472	-1.098530
C	-4.115612	3.087318	-2.383259
H	-4.914408	2.976772	-3.128422
H	-3.977555	4.149735	-2.157086
H	-3.193203	2.676752	-2.822669
N	-4.719864	1.045567	-1.203676
C	-4.778533	0.243485	-0.001611
H	-5.577083	-0.505979	-0.084341
H	-3.814205	-0.270113	0.157041
H	-4.986849	0.890704	0.854747
C	-4.544615	0.325102	-2.456620
H	-5.221356	0.706259	-3.232927
H	-3.503345	0.399547	-2.809987
H	-4.797953	-0.729730	-2.288375
O	-1.308064	-4.686252	-0.173848
C	-0.729770	-5.322407	0.712004
C	-1.508860	-5.964272	1.830976
H	-1.213742	-5.569662	2.812099
H	-2.571398	-5.756402	1.672784
H	-1.360996	-7.051791	1.853749
N	0.621295	-5.479789	0.719175
C	1.424974	-4.938005	-0.356362
H	1.837623	-5.745759	-0.979961
H	0.809846	-4.284681	-0.980651
H	2.264736	-4.360687	0.058723
C	1.349900	-6.294607	1.669185
H	0.778202	-6.459214	2.585365
H	1.611851	-7.272381	1.236242
H	2.284088	-5.784082	1.940701
O	-4.726986	-3.124379	-1.513683
C	-5.257456	-3.103150	-0.399033
C	-4.453950	-3.375907	0.847514
H	-4.884312	-4.185332	1.451122
H	-3.436496	-3.665170	0.557056
H	-4.410926	-2.481236	1.485660
N	-6.580469	-2.819151	-0.242088
C	-7.373813	-2.429002	-1.390040
H	-7.593628	-1.349343	-1.362827
H	-6.826741	-2.660585	-2.306083
H	-8.327620	-2.972855	-1.385827
C	-7.235146	-2.629851	1.036129
H	-6.680075	-3.102221	1.848785
H	-7.352170	-1.557860	1.264124
H	-8.235752	-3.080135	1.001341

***

$c3_{para}$
| Charge 0
| Electronic Energy (a.u.) -2146.86732164

C	-0.738355	-2.542310	-3.142266
H	-1.663103	-2.463534	-3.713342
C	0.104060	-3.614378	-3.282509
H	-0.143256	-4.421024	-3.973544
C	-0.429637	-1.465120	-2.239861
C	1.287570	-3.675545	-2.526498
H	1.968814	-4.520988	-2.594700
C	1.550636	-2.631655	-1.655745
C	2.775502	-2.690245	-0.813682
C	4.024648	-2.868728	-1.412453
H	4.100886	-2.939682	-2.496164
C	5.154535	-2.934305	-0.606250
H	6.141923	-3.056452	-1.048815
C	5.000767	-2.836280	0.771243
H	5.855232	-2.879265	1.442855
C	3.715339	-2.670463	1.277434
H	3.558505	-2.593532	2.355459
C	1.932507	2.248906	0.127579
C	3.073390	2.453497	-0.711946
C	4.315845	1.954502	-0.382125
C	4.483909	1.184252	0.784504
C	3.394286	0.895924	1.585574
C	2.116501	1.408747	1.268322
N	0.744659	-1.556428	-1.514128
N	2.621894	-2.593645	0.516299
O	-1.181755	-0.469691	-2.101668
Pd	1.333569	0.232998	-0.440280
C	5.854011	0.724318	1.132169
F	6.671213	1.756718	1.422230
F	5.880334	-0.096708	2.190387
F	6.446523	0.072984	0.113584
H	1.334887	1.323440	2.024066
H	3.514295	0.303581	2.490954
H	5.174885	2.170715	-1.017014
H	2.976840	3.094241	-1.587682
C	-0.060475	3.480885	1.055618
C	0.322048	3.550695	-1.306560
C	-1.565717	4.747973	-0.363786
C	-1.178905	4.290003	0.903991
H	0.219614	3.166694	2.060938
H	0.882884	3.251730	-2.192599
C	0.719983	3.089102	-0.041165
C	-0.791243	4.360964	-1.469232
H	-1.082557	4.696111	-2.465744
H	-1.763050	4.583522	1.777371
N	-2.661465	5.582105	-0.518920
H	-3.079648	5.582025	-1.443549
H	-3.358627	5.524503	0.216215
Cs	-2.996726	1.486118	-0.753328
O	-5.047445	-0.836931	-1.083460
C	-5.215280	-1.623323	-0.149416
C	-6.409464	-1.492508	0.761741
H	-6.109878	-1.352179	1.808321
H	-6.983661	-0.618360	0.441648

H	-7.058450	-2.376333	0.712986
N	-4.337842	-2.634417	0.103763
C	-3.214475	-2.846799	-0.787472
H	-2.966488	-1.919715	-1.314635
H	-2.339967	-3.168549	-0.202656
H	-3.437697	-3.634666	-1.525041
C	-4.558443	-3.694381	1.064549
H	-5.347755	-3.438956	1.774847
H	-4.835370	-4.632477	0.557925
H	-3.634294	-3.877362	1.631198
O	-3.625127	0.202542	2.038223
C	-3.325557	-0.759523	2.751264
C	-4.231636	-1.216761	3.865963
H	-3.719955	-1.201016	4.837027
H	-5.088745	-0.538196	3.910355
H	-4.597185	-2.239966	3.703495
N	-2.163356	-1.435060	2.560100
C	-1.241457	-0.919463	1.561531
H	-0.318447	-1.509749	1.588947
H	-1.657421	-0.985109	0.545728
H	-0.987535	0.133419	1.760577
C	-1.692676	-2.530444	3.385481
H	-2.505260	-2.957269	3.977890
H	-1.287151	-3.326149	2.746540
H	-0.896079	-2.201243	4.069748

***

TS c1$_{para}$-c2$_{para}$

Charge	0
Electronic Energy (a.u.)	-3006.33262707

C	3.770106	-6.528731	0.671991
H	3.011709	-7.308099	0.657993
C	5.076313	-6.786492	1.006379
H	5.382554	-7.798427	1.267915
C	3.379567	-5.203424	0.332516
C	6.023307	-5.751561	1.019160
H	7.053258	-5.956408	1.294556
C	5.602149	-4.478856	0.678924
C	6.506169	-3.308096	0.672347
C	7.863930	-3.399945	0.976330
H	8.310090	-4.358785	1.225861
C	8.650276	-2.257178	0.957714
H	9.710436	-2.322466	1.193429
C	8.068436	-1.037360	0.635868
H	8.646432	-0.117521	0.609941
C	6.713822	-1.013124	0.341200
H	6.208989	-0.082722	0.082340
C	3.625728	-0.237980	-0.550607
C	3.716402	0.281053	-1.853635
C	3.768013	1.651325	-2.086826
C	3.715811	2.544154	-1.014221
C	3.577800	2.058793	0.285381
C	3.528446	0.683049	0.505815
N	4.315383	-4.222616	0.336316
N	5.951173	-2.112206	0.357672
O	2.165092	-4.926238	0.026273
Pd	3.864979	-2.191726	-0.159247

C	3.751604	4.004672	-1.304308
F	3.914484	4.751955	-0.203789
F	4.745688	4.328301	-2.148352
F	2.616433	4.430851	-1.896790
H	3.433679	0.326258	1.533876
H	3.513138	2.747833	1.127621
H	3.862069	2.031770	-3.104841
H	3.771988	-0.394688	-2.708767
C	0.876553	-1.698355	0.050825
C	1.587708	-2.470660	-2.109709
C	-0.444077	-1.163510	-1.904488
C	-0.234050	-1.100416	-0.501061
H	1.019804	-1.624597	1.133343
H	2.286780	-3.019127	-2.747222
C	1.836311	-2.419745	-0.711705
H	2.041148	-3.676084	-0.276983
C	0.489837	-1.877731	-2.696899
H	0.320655	-1.947854	-3.773381
H	-0.972388	-0.547466	0.096206
N	-1.531009	-0.573291	-2.453236
H	-1.562938	-0.532914	-3.466240
H	-2.069377	0.181614	-1.944013
Cs	0.011955	2.382409	-0.658786
O	-2.363326	0.830491	0.639746
C	-3.227282	1.397325	-0.137194
O	-4.344843	1.826283	0.306823
O	-2.942065	1.514040	-1.410485
Cs	-5.708657	0.170895	-1.939477
O	-7.758951	-0.891194	0.210377
C	-8.157985	-1.968378	0.662147
C	-7.452842	-3.255614	0.320578
H	-8.130476	-3.978662	-0.151675
H	-6.631591	-3.024764	-0.367339
H	-7.031473	-3.733409	1.215635
N	-9.237378	-2.044032	1.488093
C	-9.999266	-0.853049	1.799876
H	-10.048573	-0.706960	2.888128
H	-9.519985	0.012883	1.338976
H	-11.027258	-0.945794	1.419405
C	-9.796706	-3.272932	2.011487
H	-9.092346	-4.104016	1.936357
H	-10.044317	-3.135035	3.072901
H	-10.721669	-3.544504	1.479401
O	-4.430166	-2.596770	-1.137788
C	-3.805527	-2.541879	-0.071780
C	-4.266739	-1.667020	1.060867
H	-3.616125	-0.769622	1.118160
H	-5.293963	-1.342358	0.855615
H	-4.247736	-2.182697	2.029817
N	-2.653647	-3.245340	0.118866
C	-2.179987	-4.150760	-0.906753
H	-2.301522	-5.199306	-0.594448
H	-2.750750	-3.981989	-1.822440
H	-1.112993	-3.970450	-1.103484
C	-1.967024	-3.319087	1.393925
H	-1.932621	-2.343866	1.891755
H	-2.437009	-4.048638	2.071993
H	-0.932044	-3.635825	1.217765

O	0.568879	5.273646	0.406347
C	1.449857	5.679220	1.168867
C	1.809973	4.908452	2.412021
H	1.510874	5.457980	3.315741
H	1.277539	3.949060	2.394935
H	2.890239	4.726515	2.485736
N	2.119263	6.843695	0.940491
C	1.967045	7.523249	-0.328908
H	2.873729	7.399537	-0.942697
H	1.113810	7.102278	-0.864211
H	1.803706	8.596921	-0.167133
C	3.229889	7.302203	1.749046
H	3.113635	7.007442	2.795058
H	4.192111	6.917210	1.373989
H	3.265267	8.398125	1.713430
O	0.101593	1.969062	2.421897
C	-0.975836	1.617660	2.915020
C	-1.155950	0.213611	3.426593
H	-1.917321	-0.284591	2.812721
H	-0.202733	-0.315721	3.328728
H	-1.472407	0.180103	4.477016
N	-2.020584	2.475670	3.073982
C	-2.028384	3.711988	2.317520
H	-2.419439	4.531247	2.937488
H	-1.017967	3.968822	1.986671
H	-2.685711	3.592185	1.439784
C	-3.364124	2.017830	3.391650
H	-3.343482	1.156370	4.065146
H	-3.894735	2.828978	3.911500
H	-3.890265	1.766016	2.451657

***Meta*-arylation**

\# **TS c1$_{ortho}$-c2$_{meta}$**
| Charge 0
| Electronic Energy (a.u.) -3006.32155677 |

C	0.577830	5.339197	-1.055700
H	-0.046020	5.910890	-1.739540
C	0.934576	5.820064	0.181531
H	0.584976	6.799920	0.504360
C	1.034040	4.057609	-1.464981
C	1.755358	5.064848	1.033273
H	2.042976	5.454026	2.005632
C	2.190875	3.830770	0.586423
C	3.101218	2.956342	1.356443
C	3.613942	3.307673	2.603886
H	3.336519	4.252352	3.063766
C	4.494675	2.452193	3.251257
H	4.905876	2.726826	4.220730
C	4.849854	1.254343	2.642760
H	5.545548	0.559300	3.107740
C	4.293942	0.954173	1.407372
H	4.536147	0.027049	0.887792
C	3.271010	-0.440625	-1.338698
C	2.577507	-1.527144	-0.790544
C	3.136927	-2.803588	-0.782536

C	4.397268	-3.012274	-1.336233
C	5.095975	-1.943584	-1.902130
C	4.533322	-0.670851	-1.906207
N	1.819938	3.348313	-0.624399
N	3.444063	1.776155	0.779844
O	0.721960	3.559172	-2.610160
Pd	2.537000	1.411016	-1.115923
C	5.038807	-4.355532	-1.345389
F	6.222469	-4.350615	-0.701932
F	4.287133	-5.299605	-0.766158
F	5.306700	-4.780947	-2.594513
H	5.095287	0.152180	-2.352576
H	6.081198	-2.110807	-2.340154
H	2.569751	-3.618827	-0.333878
H	1.586290	-1.427971	-0.338854
H	-0.845210	-1.908246	-2.331222
N	-0.627680	-1.904266	-3.355603
C	0.544282	0.121662	-2.802121
C	1.551785	1.074399	-3.010304
C	0.350952	-0.974245	-3.660650
C	2.358351	0.951670	-4.163463
H	1.200789	2.356004	-2.721942
C	1.170746	-1.061621	-4.796983
C	2.151768	-0.100746	-5.048103
H	3.147464	1.680708	-4.357804
H	1.039156	-1.900381	-5.483293
H	2.768831	-0.196528	-5.942347
H	-0.438890	-2.833498	-3.718910
O	0.123772	-2.563786	0.962817
O	-1.061627	-1.509483	-0.625911
C	-0.590971	-2.628190	-0.105380
O	-0.859311	-3.733623	-0.686771
Cs	-3.696067	-2.836396	-1.495555
Cs	-0.025956	0.441638	1.491640
O	-1.914221	2.903576	0.832654
C	-2.172081	3.161615	-0.349834
C	-2.077810	2.093445	-1.409425
H	-1.215422	2.288587	-2.064301
H	-1.940495	1.117437	-0.928135
H	-2.973480	2.040867	-2.041894
N	-2.539653	4.409796	-0.744883
C	-2.668251	5.475984	0.225166
H	-3.701578	5.853480	0.244789
H	-2.398959	5.101827	1.215050
H	-2.005882	6.312356	-0.041196
C	-2.851371	4.796523	-2.104532
H	-2.582340	4.018251	-2.820861
H	-3.923942	5.023678	-2.208997
H	-2.291006	5.705791	-2.365334
O	-5.090527	-0.044322	-1.108368
C	-4.956152	0.800503	-0.219397
C	-4.315664	0.453829	1.099969
H	-4.987494	0.661080	1.944334
H	-4.070517	-0.614306	1.099615
H	-3.399847	1.042746	1.256425
N	-5.357968	2.091418	-0.386875
C	-6.042165	2.481842	-1.601898
H	-5.549602	3.357469	-2.048703

H	-6.017448	1.651274	-2.310225
H	-7.089059	2.746825	-1.391587
C	-5.337385	3.087001	0.667693
H	-4.466810	2.964041	1.319173
H	-5.269788	4.081316	0.207339
H	-6.254023	3.054709	1.277088
O	-4.661233	-3.009047	1.480475
C	-5.377249	-2.873309	2.475784
C	-4.939100	-3.400364	3.817946
H	-5.622214	-4.173606	4.191412
H	-3.943680	-3.837234	3.702628
H	-4.891712	-2.606255	4.572882
N	-6.578134	-2.233966	2.412444
C	-7.080531	-1.775995	1.132833
H	-7.566870	-0.799117	1.259247
H	-6.257195	-1.673039	0.421642
H	-7.824742	-2.478928	0.726912
C	-7.539472	-2.175906	3.494929
H	-7.079930	-2.409301	4.457458
H	-7.961352	-1.163644	3.556158
H	-8.368573	-2.880044	3.321997
O	1.758697	-0.229453	3.976963
C	2.722722	-0.995900	3.901784
C	2.807506	-2.047154	2.825212
H	2.979477	-3.045326	3.250190
H	1.875132	-2.073769	2.242808
H	3.646385	-1.840978	2.143658
N	3.764919	-0.921960	4.778175
C	3.722954	0.014606	5.879992
H	4.638500	0.624090	5.894033
H	2.853013	0.665049	5.767567
H	3.654712	-0.517899	6.840967
C	4.898471	-1.823324	4.782383
H	5.074838	-2.254993	3.794118
H	5.798756	-1.263357	5.068793
H	4.763574	-2.641026	5.507307
H	-0.067822	0.142106	-1.897913

***

c2*meta*

| Charge 0
| Electronic Energy (a.u.) -3006.34814332

C	0.211604	4.932496	-0.932231
H	-0.533062	5.359894	-1.599773
C	0.599435	5.539692	0.240134
H	0.160231	6.490243	0.537194
C	0.801256	3.704945	-1.266809
C	1.561713	4.931214	1.049786
H	1.875790	5.414759	1.969312
C	2.108250	3.721363	0.651359
C	3.130598	3.018410	1.459563
C	3.545384	3.483526	2.708030
H	3.114633	4.382676	3.138888
C	4.517502	2.784216	3.409990
H	4.846955	3.140815	4.383909
C	5.063000	1.635183	2.851567
H	5.835957	1.063072	3.359308

C	4.596869	1.225344	1.610380
H	4.990428	0.329887	1.127211
C	3.850193	-0.489126	-1.179058
C	3.314226	-1.749506	-0.869306
C	4.121603	-2.886424	-0.875122
C	5.469234	-2.788097	-1.213723
C	6.012929	-1.548632	-1.553267
C	5.207590	-0.414899	-1.537415
N	1.726210	3.115592	-0.505794
N	3.652882	1.888860	0.935203
O	0.409972	3.129705	-2.397844
Pd	2.796632	1.201521	-0.959265
C	6.359530	-3.980464	-1.247797
F	7.450894	-3.816684	-0.475824
F	5.754041	-5.099376	-0.831218
F	6.825405	-4.227909	-2.486957
H	5.653942	0.547792	-1.796365
H	7.066380	-1.473851	-1.826997
H	3.695130	-3.854351	-0.612780
H	2.256526	-1.864503	-0.589981
H	-1.467538	-1.257024	-2.517957
N	-1.311703	-0.985293	-3.509107
C	0.680006	-0.017176	-2.574241
C	1.941436	0.573118	-2.683865
C	-0.048353	-0.450441	-3.700608
C	2.495064	0.738200	-3.966312
H	0.831658	2.241506	-2.489532
C	0.524353	-0.278803	-4.970420
C	1.780650	0.311075	-5.087859
H	3.482508	1.186810	-4.094509
H	-0.019848	-0.609030	-5.857188
H	2.215490	0.435715	-6.081206
H	-1.583041	-1.686515	-4.191054
O	0.301466	-2.239174	0.308817
O	-1.472946	-1.276307	-0.673179
C	-0.714524	-2.340119	-0.478876
O	-1.011517	-3.423354	-1.082611
Cs	-3.995889	-2.692632	-1.597815
Cs	-0.147611	0.577833	1.366302
O	-2.347410	2.673948	0.555456
C	-2.668383	2.800584	-0.633254
C	-2.580011	1.646456	-1.597318
H	-1.957679	1.901880	-2.465219
H	-2.155162	0.762281	-1.100456
H	-3.575863	1.374268	-1.978095
N	-3.122674	3.985713	-1.128767
C	-3.195706	5.158683	-0.281453
H	-4.229510	5.530644	-0.228649
H	-2.849878	4.902597	0.722552
H	-2.566974	5.966266	-0.685840
C	-3.393690	4.218195	-2.534663
H	-3.783485	3.322238	-3.025552
H	-4.152938	5.004998	-2.623804
H	-2.491383	4.550600	-3.072789
O	-5.544466	-0.029591	-0.872179
C	-5.299503	0.665374	0.119292
C	-4.526207	0.125607	1.293493
H	-5.090806	0.206843	2.232218

H	-4.288127	-0.928294	1.115132
H	-3.593473	0.695513	1.417449
N	-5.682159	1.968020	0.206254
C	-6.372225	2.600416	-0.899080
H	-5.872556	3.544426	-1.160194
H	-6.354877	1.930619	-1.762117
H	-7.417725	2.821831	-0.639693
C	-5.481398	2.764526	1.406467
H	-4.412801	2.909328	1.616495
H	-5.938602	3.746923	1.249870
H	-5.963814	2.309039	2.281593
O	-4.837383	-3.380358	1.325625
C	-5.482346	-3.372300	2.376762
C	-4.961981	-4.070672	3.606371
H	-5.637329	-4.872247	3.932037
H	-3.988751	-4.506052	3.363461
H	-4.839004	-3.377918	4.448561
N	-6.677495	-2.727409	2.480605
C	-7.274421	-2.128402	1.303573
H	-7.748322	-1.176201	1.577195
H	-6.508114	-1.937819	0.548050
H	-8.047750	-2.785783	0.875585
C	-7.556582	-2.819686	3.628802
H	-7.009042	-3.085154	4.535473
H	-8.035547	-1.846386	3.796747
H	-8.349743	-3.565690	3.464378
O	1.684165	-0.104075	3.829061
C	2.684492	-0.811625	3.682635
C	2.805566	-1.770987	2.528061
H	3.073027	-2.782642	2.861616
H	1.860582	-1.823532	1.970994
H	3.593207	-1.443872	1.833411
N	3.739315	-0.753357	4.544249
C	3.660635	0.073112	5.729545
H	4.555291	0.706562	5.810010
H	2.772786	0.706044	5.666372
H	3.598050	-0.547378	6.636376
C	4.902830	-1.612843	4.449663
H	5.171566	-1.813886	3.407827
H	5.754041	-1.106791	4.921341
H	4.749574	-2.572835	4.966680
H	0.213933	-0.209083	-1.602557

***

c2b$_{meta}$

| Charge 0
| Electronic Energy (a.u.) -3006.38412377

C	0.084895	3.437898	2.383020
H	-0.873902	3.262157	2.871597
C	0.752961	4.623373	2.520686
H	0.337431	5.425275	3.131731
C	0.614499	2.374661	1.564418
C	1.974145	4.816879	1.851017
H	2.500086	5.763990	1.928041
C	2.487888	3.758200	1.121918
C	3.747171	3.907272	0.355121
C	4.345149	5.146773	0.115395

H	3.901847	6.057634	0.507478
C	5.499847	5.214386	-0.649163
H	5.968762	6.176867	-0.844147
C	6.040114	4.043680	-1.169851
H	6.938806	4.050890	-1.781192
C	5.396273	2.848185	-0.892589
H	5.778500	1.900439	-1.272526
C	4.808084	-0.280606	-0.069551
C	5.938468	-0.432252	0.754046
C	7.069552	-1.112149	0.318161
C	7.097300	-1.669403	-0.961787
C	5.985569	-1.552654	-1.791927
C	4.856466	-0.871426	-1.342218
N	1.874482	2.549077	1.030176
N	4.283142	2.778803	-0.153483
O	-0.046954	1.331855	1.347782
Pd	3.298750	0.924314	0.462057
C	8.323072	-2.396531	-1.390400
F	8.269352	-2.810009	-2.662984
F	9.427707	-1.635811	-1.272999
F	8.552881	-3.488636	-0.636538
H	3.996964	-0.794536	-2.011233
H	5.998628	-1.991210	-2.788651
H	7.938836	-1.210624	0.970133
H	5.943362	-0.002308	1.757909
C	1.762091	-1.646257	0.319315
C	2.725005	-1.225251	2.478373
C	1.385154	-3.219113	2.114448
C	1.170441	-2.832470	0.783063
H	1.568752	-1.362181	-0.716347
H	3.332425	-0.622524	3.158142
H	0.945910	-4.148908	2.485233
C	2.512920	-0.812242	1.149956
H	-0.145714	0.656356	-0.327920
C	2.161561	-2.414322	2.945648
H	2.329897	-2.723149	3.979364
N	0.423501	-3.622923	-0.092655
H	-0.216526	-4.254082	0.383981
H	-0.071391	-3.070252	-0.804306
Cs	-4.180849	-2.448353	-1.234734
O	-0.805292	-1.714070	-2.061949
C	-0.960820	-0.869450	-1.149728
O	-0.162160	0.243750	-1.228469
O	-1.767467	-0.924388	-0.188101
Cs	-3.151811	1.479005	1.023573
O	-1.915949	3.735014	-0.756866
C	-1.925340	4.966592	-0.702680
C	-0.638977	5.750344	-0.673773
H	-0.555748	6.361821	0.234068
H	0.192797	5.039385	-0.700555
H	-0.556175	6.425259	-1.535766
N	-3.092875	5.670092	-0.682587
C	-4.350076	4.953284	-0.602507
H	-5.110475	5.486637	-1.187728
H	-4.225390	3.944022	-1.007130
H	-4.704596	4.882453	0.438819
C	-3.183524	7.097747	-0.453050
H	-2.251834	7.605417	-0.711245

H	-3.980906	7.516110	-1.080474
H	-3.425444	7.318203	0.598524
O	-5.266390	0.527169	-1.373717
C	-5.310499	1.519173	-2.111543
C	-6.603604	2.264574	-2.318724
H	-6.887665	2.301446	-3.377885
H	-7.387441	1.745698	-1.759953
H	-6.538980	3.298772	-1.955644
N	-4.204451	1.984029	-2.749877
C	-2.943942	1.272198	-2.648665
H	-2.148716	1.967878	-2.354189
H	-3.019142	0.485551	-1.894793
H	-2.680321	0.814668	-3.614426
C	-4.170865	3.120752	-3.647144
H	-5.108919	3.679227	-3.634239
H	-3.359879	3.798919	-3.343109
H	-3.972757	2.791361	-4.678198
O	-3.311709	-5.428979	-0.629680
C	-2.177314	-5.740314	-1.002406
C	-1.688627	-5.372013	-2.380936
H	-1.340525	-6.248786	-2.941164
H	-2.518776	-4.915476	-2.929678
H	-0.859811	-4.652211	-2.336671
N	-1.311930	-6.412979	-0.191686
C	-1.740720	-6.862735	1.117043
H	-1.081988	-6.451666	1.897266
H	-2.767180	-6.535417	1.295689
H	-1.692559	-7.960227	1.175207
C	0.006316	-6.877290	-0.579022
H	0.364338	-6.379427	-1.482129
H	0.718395	-6.658645	0.229985
H	0.002828	-7.965439	-0.747257
O	-4.394103	-1.566934	1.821774
C	-3.755839	-1.637734	2.877629
C	-4.042407	-0.694413	4.020822
H	-3.171355	-0.069744	4.260175
H	-4.874789	-0.045632	3.730456
H	-4.322176	-1.232648	4.935235
N	-2.780960	-2.563964	3.048174
C	-2.461858	-3.421816	1.916688
H	-1.721937	-4.162967	2.241846
H	-3.353732	-3.959193	1.569953
H	-2.049123	-2.830347	1.083993
C	-1.880714	-2.620480	4.182605
H	-2.261823	-2.037661	5.023913
H	-1.767582	-3.660918	4.514959
H	-0.883980	-2.236973	3.913913

***

TS c2b-c3$_{meta}$HCO$_3$

| Charge | 0 |
| Electronic Energy (a.u.) | -3006.36803685 |

C	0.134464	3.577554	2.274945
H	-0.835105	3.447376	2.756565
C	0.835000	4.749034	2.387378
H	0.430320	5.581220	2.964689
C	0.655396	2.471009	1.512002

C	2.076946	4.883648	1.744949
H	2.639464	5.809989	1.819322
C	2.567637	3.786197	1.053740
C	3.867748	3.864389	0.339259
C	4.518228	5.073935	0.077936
H	4.079846	6.017862	0.390402
C	5.724499	5.065711	-0.606991
H	6.237770	6.002097	-0.817693
C	6.261979	3.853069	-1.026574
H	7.202909	3.802362	-1.568717
C	5.554538	2.695363	-0.739127
H	5.925039	1.715174	-1.047238
C	4.523576	-0.729065	0.110527
C	5.682996	-0.867643	0.909657
C	6.868672	-1.359873	0.389916
C	6.943864	-1.746286	-0.952039
C	5.815325	-1.632603	-1.763009
C	4.628098	-1.140498	-1.236747
N	1.915335	2.603423	0.972522
N	4.393264	2.700073	-0.079398
O	-0.014973	1.419456	1.350173
Pd	3.285182	0.815418	0.484032
C	8.233012	-2.275811	-1.465548
F	8.196735	-2.565574	-2.772743
F	9.245653	-1.404470	-1.286895
F	8.611587	-3.401587	-0.828536
H	3.755742	-1.075028	-1.887998
H	5.862948	-1.926700	-2.810375
H	7.751035	-1.443087	1.026129
H	5.650737	-0.580179	1.961051
C	1.959011	-1.899243	0.198368
C	2.993237	-1.532485	2.343462
C	1.378806	-3.320773	2.062877
C	1.214873	-2.970109	0.713284
H	1.808377	-1.638450	-0.851216
H	3.695724	-1.002472	2.987990
H	0.820701	-4.166274	2.471128
C	2.852527	-1.162797	0.989218
H	-0.136184	0.601405	-0.285788
C	2.256370	-2.593062	2.862925
H	2.370890	-2.866560	3.913194
N	0.384695	-3.703013	-0.129008
H	-0.306424	-4.264710	0.361451
H	-0.061524	-3.133376	-0.858216
Cs	-4.344547	-2.296165	-1.023555
O	-0.917133	-1.773916	-1.983355
C	-1.006506	-0.928155	-1.059802
O	-0.166911	0.148620	-1.166773
O	-1.780471	-0.954445	-0.071581
Cs	-3.112150	1.542418	1.086893
O	-1.893975	3.681395	-0.875209
C	-1.826846	4.912040	-0.862345
C	-0.496925	5.617846	-0.918809
H	-0.338447	6.256973	-0.040694
H	0.291139	4.859641	-0.954669
H	-0.415048	6.254893	-1.809111
N	-2.948196	5.685872	-0.813074
C	-4.239587	5.048679	-0.651308

H	-4.990606	5.589331	-1.242090
H	-4.181540	4.012882	-0.999244
H	-4.559912	5.057366	0.403109
C	-2.941697	7.122604	-0.628593
H	-2.002833	7.565889	-0.966935
H	-3.755190	7.566516	-1.216679
H	-3.098514	7.391256	0.427924
O	-5.309180	0.686845	-1.209761
C	-5.327791	1.630172	-2.010368
C	-6.590575	2.422175	-2.230363
H	-6.905331	2.401853	-3.281256
H	-7.379197	1.978759	-1.616267
H	-6.469583	3.473946	-1.940405
N	-4.221776	1.994346	-2.709108
C	-2.998779	1.218409	-2.614576
H	-2.151808	1.887993	-2.425520
H	-3.067209	0.503651	-1.791762
H	-2.820288	0.665445	-3.549428
C	-4.162887	3.073023	-3.674350
H	-5.073191	3.675589	-3.667323
H	-3.312091	3.727403	-3.435371
H	-4.014383	2.673790	-4.688920
O	-3.478126	-5.288396	-0.589124
C	-2.414936	-5.532867	-1.168097
C	-2.138153	-4.948571	-2.529639
H	-1.582106	-5.613581	-3.198697
H	-3.099385	-4.712002	-2.999222
H	-1.573972	-4.005624	-2.420885
N	-1.441167	-6.291417	-0.593645
C	-1.670087	-6.904790	0.698745
H	-0.894501	-6.587699	1.412381
H	-2.652495	-6.607565	1.071497
H	-1.628328	-8.000772	0.615027
C	-0.205860	-6.685185	-1.245580
H	0.132043	-5.934741	-1.963657
H	0.577590	-6.780853	-0.483444
H	-0.312563	-7.655976	-1.753804
O	-4.312894	-1.491851	2.052024
C	-3.578100	-1.611476	3.038384
C	-3.682356	-0.654977	4.201240
H	-2.746716	-0.101499	4.358650
H	-4.486253	0.057038	3.989721
H	-3.917761	-1.175707	5.138313
N	-2.660156	-2.606680	3.121198
C	-2.500910	-3.480001	1.967783
H	-1.826088	-4.300250	2.240896
H	-3.464106	-3.916873	1.678370
H	-2.082216	-2.926308	1.110855
C	-1.659425	-2.709369	4.165491
H	-1.966652	-2.173254	5.066035
H	-1.517919	-3.764000	4.434292
H	-0.689606	-2.307792	3.831260

***

c3$_{meta}$-HCO$_3$

| Charge 0

| Electronic Energy (a.u.) -3006.42478027 |

C	1.524490	-2.288073	-3.114270
H	0.554579	-2.660453	-3.447004
C	2.692251	-2.886427	-3.517849
H	2.663877	-3.760551	-4.169375
C	1.538496	-1.139741	-2.248824
C	3.926090	-2.361415	-3.098973
H	4.871855	-2.801226	-3.408049
C	3.904333	-1.223727	-2.308960
C	5.181485	-0.621433	-1.842628
C	6.123176	-1.412000	-1.180306
H	5.932743	-2.473638	-1.029513
C	7.278085	-0.812438	-0.691575
H	8.016702	-1.399838	-0.148291
C	7.466431	0.548640	-0.900066
H	8.352278	1.062543	-0.532767
C	6.487052	1.247124	-1.599625
H	6.609132	2.314708	-1.794863
C	2.928105	2.861775	0.699181
C	4.308765	3.229036	0.699464
C	5.227448	2.600557	1.510826
C	4.824325	1.540034	2.344599
C	3.503283	1.129098	2.354652
C	2.538027	1.785724	1.556235
N	2.767405	-0.624869	-1.893641
N	5.363928	0.690631	-2.056010
O	0.473234	-0.621554	-1.819651
Pd	2.801195	0.993487	-0.458979
C	5.858416	0.872753	3.176992
F	5.369289	-0.120331	3.930042
F	6.844483	0.340924	2.426088
F	6.467750	1.734867	4.013300
H	1.484579	1.561867	1.724898
H	3.182044	0.319361	3.007808
H	6.269459	2.921587	1.507632
H	4.636384	4.066012	0.084188
C	0.590638	3.711237	0.400952
C	2.292113	4.471605	-1.127842
C	-0.005367	5.190200	-1.409171
C	-0.392558	4.413728	-0.307072
H	0.266527	3.108193	1.245709
H	3.319052	4.466657	-1.490499
H	-0.759332	5.754460	-1.960605
C	1.924265	3.701569	-0.008732
H	-0.165241	0.325689	-0.377325
C	1.328804	5.211647	-1.803670
H	1.619399	5.804827	-2.670783
N	-1.714063	4.346236	0.110811
H	-2.381405	4.611482	-0.607468
H	-1.971462	3.452169	0.552472
Cs	-5.375039	-0.513316	0.029153
O	-2.441380	1.658524	1.129251
C	-1.881613	0.841043	0.359721
O	-0.513652	0.913873	0.334675
O	-2.419344	-0.021560	-0.371662
Cs	-1.566561	-2.824814	-0.970281
O	0.838327	-2.642584	1.281114
C	1.656468	-3.449012	0.835145
C	3.075105	-3.045116	0.532513

H	3.285535	-3.086412	-0.545762
H	3.222789	-2.014290	0.872505
H	3.798998	-3.699965	1.035495
N	1.321119	-4.749889	0.584467
C	0.068159	-5.285313	1.080749
H	0.254959	-6.036677	1.863492
H	-0.529597	-4.474670	1.508323
H	-0.494818	-5.771681	0.270451
C	2.259668	-5.735112	0.082758
H	2.969048	-5.287978	-0.620006
H	2.819830	-6.221504	0.896602
H	1.700894	-6.509372	-0.456966
O	-3.970042	-3.076542	1.272869
C	-3.321167	-3.628569	2.170691
C	-3.622548	-5.048440	2.573862
H	-3.852146	-5.130438	3.643629
H	-4.487393	-5.389415	1.998178
H	-2.776621	-5.717222	2.364759
N	-2.301267	-2.996375	2.808422
C	-2.035327	-1.591765	2.552259
H	-0.965732	-1.453198	2.346497
H	-2.608458	-1.254046	1.685176
H	-2.313134	-0.985304	3.428108
C	-1.543649	-3.555317	3.911247
H	-1.630910	-4.643307	3.955827
H	-0.483124	-3.304006	3.775706
H	-1.879220	-3.133386	4.870934
O	-6.173913	2.459102	0.709827
C	-5.431796	2.918596	1.582643
C	-5.081501	2.113713	2.806332
H	-5.169800	2.687345	3.736539
H	-5.757930	1.254034	2.862157
H	-4.047753	1.754314	2.703862
N	-4.882360	4.160515	1.480905
C	-5.300813	5.048110	0.417268
H	-4.426692	5.474502	-0.093817
H	-5.908311	4.490009	-0.298698
H	-5.895692	5.882661	0.820421
C	-4.055743	4.764554	2.505918
H	-3.480612	4.010315	3.049200
H	-3.336530	5.439031	2.025471
H	-4.655295	5.346268	3.224415
O	-4.420475	-0.120490	-2.933703
C	-3.314170	0.387242	-3.134593
C	-2.209539	-0.387346	-3.805180
H	-1.320172	-0.455976	-3.159077
H	-2.581733	-1.392130	-4.033216
H	-1.898189	0.088271	-4.745473
N	-3.036680	1.680836	-2.805435
C	-3.991126	2.410207	-1.991284
H	-3.945572	3.477281	-2.247956
H	-4.999493	2.041917	-2.199101
H	-3.779150	2.287490	-0.914951
C	-1.680282	2.199125	-2.815838
H	-1.161994	1.933766	-3.743564
H	-1.721759	3.294170	-2.763454
H	-1.084318	1.827186	-1.965380

***

$c3_{meta}$
| Charge 0
| Electronic Energy (a.u.) -2146.86567892 |

C	0.802277	-2.754311	-3.548460
H	-0.007550	-3.217514	-4.111647
C	2.051013	-3.316406	-3.495875
H	2.256313	-4.247335	-4.025981
C	0.499669	-1.526564	-2.853667
C	3.075650	-2.682083	-2.768735
H	4.087458	-3.080270	-2.733205
C	2.762576	-1.498298	-2.125257
C	3.819934	-0.766027	-1.377785
C	4.549997	-1.410347	-0.376967
H	4.333019	-2.449102	-0.130743
C	5.529687	-0.696958	0.304348
H	6.104397	-1.170635	1.098669
C	5.758275	0.628780	-0.043982
H	6.516764	1.225383	0.458261
C	4.988669	1.181908	-1.063779
H	5.145444	2.219276	-1.367085
C	1.056019	2.162940	0.858070
C	2.420432	2.305061	1.260336
C	2.994803	1.452491	2.178850
C	2.246170	0.388701	2.721723
C	0.935328	0.192442	2.330336
C	0.322406	1.075175	1.416331
N	1.530414	-0.942314	-2.130777
N	4.037656	0.515132	-1.719013
O	-0.634768	-1.003493	-2.880536
Pd	1.091932	0.492354	-0.564938
C	2.917332	-0.596435	3.608815
F	2.050478	-1.331733	4.319371
F	3.668115	-1.474383	2.909124
F	3.753275	-0.013564	4.483634
H	-0.758853	1.009949	1.306865
H	0.341280	-0.632489	2.719361
H	4.029187	1.601393	2.487393
H	3.000517	3.143256	0.874697
C	-0.974576	3.497246	0.255686
C	1.085574	3.929828	-0.925097
C	-0.932244	5.136727	-1.514672
C	-1.648428	4.442132	-0.529236
H	-1.532484	2.982350	1.040631
H	2.137493	3.712376	-1.110514
H	-1.445467	5.878310	-2.127642
C	0.380926	3.218112	0.059746
C	0.422145	4.878748	-1.695279
H	0.968855	5.421646	-2.465955
N	-2.987261	4.721883	-0.290209
H	-3.488454	5.116073	-1.079758
H	-3.510632	3.969546	0.147450
Cs	-2.627529	0.437643	-1.133844
O	-1.399008	-1.701847	1.201697
C	-0.969681	-2.656125	0.545265
C	0.479273	-3.070976	0.612007
H	0.989360	-2.879478	-0.343814

H	0.979682	-2.483317	1.389248
H	0.589147	-4.138166	0.843395
N	-1.783210	-3.383524	-0.269250
C	-3.209042	-3.130757	-0.289984
H	-3.759366	-4.029192	0.028649
H	-3.443123	-2.309245	0.393505
H	-3.541620	-2.872375	-1.307311
C	-1.354558	-4.504472	-1.080387
H	-0.277439	-4.483658	-1.262854
H	-1.619316	-5.463502	-0.608977
H	-1.859227	-4.453151	-2.054762
O	-5.348090	0.636746	0.502861
C	-5.394993	-0.404447	1.164491
C	-6.437379	-1.455091	0.876628
H	-7.006592	-1.729830	1.773133
H	-7.124969	-1.051792	0.127969
H	-5.985772	-2.372377	0.475158
N	-4.510038	-0.655964	2.168415
C	-3.546732	0.351604	2.568250
H	-2.524860	-0.017380	2.397992
H	-3.717190	1.267293	1.995664
H	-3.664526	0.574514	3.638702
C	-4.445223	-1.876871	2.945801
H	-5.100850	-2.650930	2.541517
H	-3.413510	-2.257369	2.928171
H	-4.728473	-1.685915	3.991767

***

TS c1NH$_{meta}$-c2NH$_{meta}$

| Charge 0
| Electronic Energy (a.u.) -3006.31897223

C	4.663997	-3.117723	3.666596
H	4.353560	-3.517957	4.629029
C	5.633879	-3.721535	2.903318
H	6.116496	-4.631690	3.257194
C	4.041132	-1.926177	3.198551
C	6.012061	-3.174455	1.667326
H	6.784742	-3.651291	1.070853
C	5.378682	-2.017701	1.248443
C	5.671654	-1.351062	-0.036590
C	6.559383	-1.878510	-0.972662
H	7.095232	-2.800552	-0.764035
C	6.750907	-1.226851	-2.181592
H	7.435336	-1.638039	-2.920689
C	6.058253	-0.048640	-2.432515
H	6.174760	0.496443	-3.365605
C	5.207830	0.436669	-1.450549
H	4.659805	1.367804	-1.591482
C	3.167612	2.204039	0.459104
C	1.902307	2.560800	-0.024558
C	1.681058	3.771208	-0.678846
C	2.732755	4.668073	-0.838813
C	3.998257	4.354581	-0.337593
C	4.208740	3.137015	0.299598
N	4.417845	-1.424052	1.996100
N	5.015349	-0.190866	-0.283660
O	3.137211	-1.326705	3.876886

Pd	3.642138	0.412672	1.248580
C	2.552974	5.972923	-1.532385
F	3.366709	6.091852	-2.599633
F	1.304153	6.164541	-1.974312
F	2.849603	7.014336	-0.732696
H	5.211567	2.905627	0.665803
H	4.818870	5.064525	-0.452026
H	0.681455	3.983313	-1.056493
H	1.046484	1.902522	0.129041
H	-1.431826	0.543572	1.553996
N	-1.461797	1.364028	2.169893
C	-0.212334	1.521190	2.650941
C	0.885164	0.646979	2.377689
C	0.126579	2.640617	3.474068
C	2.204331	0.896418	2.785512
H	0.674782	-0.253166	1.784638
C	1.418314	2.882637	3.922709
H	-0.679435	3.328849	3.741895
C	2.473371	2.037950	3.575933
H	2.764828	-0.212784	3.254101
H	1.603908	3.758542	4.548234
H	3.488069	2.244397	3.920181
H	-1.391359	2.283649	0.582830
O	-1.363868	2.676383	-0.336009
O	-1.256836	0.470492	-0.750123
C	-1.137287	1.634893	-1.222937
O	-0.835929	1.971587	-2.379818
Cs	1.459367	-0.388945	-2.020422
Cs	-4.287118	1.268026	0.385608
O	-7.058862	1.024183	1.794982
C	-7.910780	0.209229	2.153197
C	-7.824627	-0.465828	3.497444
H	-7.752622	-1.557563	3.405823
H	-6.931648	-0.096311	4.009580
H	-8.705175	-0.245532	4.115149
N	-8.960331	-0.130000	1.352268
C	-9.153736	0.591703	0.109279
H	-9.799267	1.472513	0.251548
H	-8.185669	0.922583	-0.276838
H	-9.627762	-0.071958	-0.624189
C	-10.078115	-0.935835	1.805362
H	-9.750199	-1.732015	2.480298
H	-10.840641	-0.329703	2.319186
H	-10.550145	-1.409537	0.936182
O	-6.016369	-0.777648	-1.231847
C	-6.788188	-1.647353	-0.812742
C	-6.504764	-2.363674	0.483265
H	-6.193382	-3.400361	0.295174
H	-5.687712	-1.841843	0.994231
H	-7.375067	-2.393695	1.149184
N	-7.904742	-2.000783	-1.501879
C	-8.196081	-1.374637	-2.774983
H	-9.250416	-1.066281	-2.804095
H	-7.556380	-0.499716	-2.906756
H	-8.021761	-2.077678	-3.603073
C	-8.820325	-3.059594	-1.133322
H	-8.553802	-3.516439	-0.178616
H	-9.843742	-2.662232	-1.057346

H	-8.820182	-3.845396	-1.903124
O	-0.873274	-2.002422	-3.355692
C	-1.870039	-1.878096	-2.640389
C	-3.029854	-1.025127	-3.075849
H	-3.015466	-0.097371	-2.487627
H	-2.889495	-0.773347	-4.131407
H	-4.006906	-1.503266	-2.943874
N	-1.970833	-2.517951	-1.438688
C	-0.809135	-3.185820	-0.895049
H	-1.102874	-4.141079	-0.438550
H	-0.080647	-3.373504	-1.688323
H	-0.344143	-2.564310	-0.110375
C	-2.985359	-2.236789	-0.446039
H	-3.891710	-1.812568	-0.889082
H	-3.259568	-3.170714	0.064659
H	-2.592114	-1.533609	0.307269
O	3.273984	-2.873593	-1.518632
C	3.281067	-3.752529	-0.652107
C	2.376906	-3.652925	0.551154
H	2.917843	-3.772743	1.497975
H	1.904398	-2.663682	0.542078
H	1.591850	-4.420992	0.520080
N	4.090058	-4.842822	-0.742867
C	4.971549	-5.001327	-1.880137
H	4.658880	-5.858496	-2.495141
H	4.948049	-4.094388	-2.487895
H	5.998852	-5.188103	-1.534994
C	4.111754	-5.951227	0.187310
H	3.424962	-5.797956	1.021561
H	3.827368	-6.881186	-0.327091
H	5.126220	-6.086352	0.591832

Oxidative addition. Gibbs energy profile in Figure 1 (bottom) and Figure S91 (bottom).

```
# aniline | Charge                         0              |
Electronic Energy (a.u.)     -287.383679536
 C          1.877336      -0.000009       0.007259
C          1.167919      -1.199978       0.003441
C         -0.221849      -1.206178      -0.004851
C         -0.938702       0.000029      -0.010370
C         -0.221839       1.206199      -0.004827
C          1.167953       1.199950       0.003434
H          2.965982      -0.000009       0.015479
H          1.703198      -2.149313       0.010243
H         -0.771128      -2.148696      -0.007542
H         -0.771082       2.148738      -0.007463
H          1.703229       2.149288       0.010231
N         -2.325427      -0.000032      -0.078743
H         -2.768530      -0.838329       0.283125
H         -2.768582       0.838462       0.282606

***
# TS c4-c5 | Charge                         0              |
Electronic Energy (a.u.)    -2158.81725833
 C         -5.558825       2.266493      -1.155986
H         -5.760077       3.299938      -1.434293
C         -6.570969       1.390327      -0.866603
H         -7.609391       1.719281      -0.910264
C         -4.182026       1.845908      -1.121918
C         -6.280868       0.060217      -0.513486
H         -7.083314      -0.639057      -0.298408
C         -4.949457      -0.314563      -0.461135
C         -4.532234      -1.687536      -0.088345
C         -5.427807      -2.673577       0.331260
H         -6.490458      -2.459394       0.406503
C         -4.953572      -3.933021       0.667250
H         -5.646448      -4.704143       0.997914
C         -3.590596      -4.195433       0.580859
H         -3.177405      -5.168131       0.835172
C         -2.756033      -3.171796       0.159724
H         -1.678527      -3.318916       0.066289
N         -3.942009       0.541296      -0.747489
N         -3.211232      -1.958015      -0.163723
O         -3.233764       2.607916      -1.421103
Pd        -1.925818      -0.237650      -0.593805
H         -2.038778       3.599731      -0.336644
N         -1.418321       4.125983       0.289140
H         -1.482860       5.129610       0.153941
C         -1.445447       3.718779       1.611722
C         -1.841707       2.412048       1.945830
C         -1.025433       4.570326       2.647195
C         -1.794123       1.972303       3.263406
H         -2.175189       1.740509       1.148189
C         -0.990486       4.122083       3.962521
H         -0.717270       5.588199       2.402086
C         -1.370643       2.819848       4.286789
H         -2.097956       0.949099       3.490270
H         -0.660561       4.803718       4.746764
H         -1.338877       2.473152       5.318438
```

C	1.354657	1.443918	1.013354
C	0.862479	1.711408	-0.250784
C	0.177600	0.711210	-0.962843
C	-0.022152	-0.546619	-0.344161
C	0.551342	-0.846001	0.910789
C	1.207714	0.162811	1.587497
H	1.010175	2.686043	-0.709310
H	-0.064394	0.875174	-2.014569
H	0.420261	-1.829524	1.367169
H	1.593406	-0.018839	2.590930
C	2.113243	2.478307	1.770005
F	1.803114	2.475839	3.076267
F	1.899505	3.718288	1.314456
F	3.447378	2.273568	1.708108
I	0.469988	-2.505032	-2.096683
Cs	3.978999	-0.826683	-0.501889
O	3.876949	1.937103	-2.028047
C	2.934631	2.082037	-2.810477
C	2.564791	0.989118	-3.782634
H	2.599368	1.339741	-4.821918
H	3.283231	0.170518	-3.671381
H	1.555422	0.597158	-3.598797
N	2.195272	3.225448	-2.839066
C	2.523061	4.322439	-1.951096
H	1.620044	4.657900	-1.418854
H	3.271921	3.992244	-1.227141
H	2.917462	5.176657	-2.520798
C	1.023093	3.446467	-3.659462
H	0.862987	2.632619	-4.369124
H	0.126862	3.538057	-3.024140
H	1.132590	4.381925	-4.226311
O	3.496883	-2.253975	2.257533
C	2.580406	-2.699397	2.951439
C	2.260665	-2.096267	4.295449
H	2.406919	-2.818289	5.109326
H	2.931410	-1.247593	4.458105
H	1.220332	-1.747444	4.347790
N	1.809612	-3.746763	2.545976
C	1.997676	-4.330642	1.234703
H	1.065398	-4.264509	0.652806
H	2.789660	-3.796251	0.704557
H	2.273152	-5.391454	1.321542
C	0.669906	-4.263077	3.276599
H	0.725160	-4.021073	4.339940
H	-0.275315	-3.866020	2.871373
H	0.647342	-5.356429	3.180797***

c5 | Charge 0 | Electronic Energy (a.u.) -2158.86312678

C	5.784271	2.182722	0.993361
H	6.130451	3.190068	1.220417
C	6.577677	1.084352	1.198502
H	7.589333	1.202844	1.587641
C	4.447928	2.045450	0.481627
C	6.086984	-0.199873	0.907457
H	6.698735	-1.087356	1.051420
C	4.802238	-0.302338	0.400359
C	4.224493	-1.636017	0.096559

C	4.488748	-2.735640	0.917940
H	5.136992	-2.628364	1.785314
C	3.881380	-3.951717	0.632108
H	4.062579	-4.817496	1.266769
C	3.034098	-4.042634	-0.467291
H	2.537856	-4.975252	-0.728943
C	2.839779	-2.899273	-1.235134
H	2.191781	-2.927316	-2.116402
N	4.014846	0.772718	0.174008
N	3.409835	-1.723971	-0.966402
O	3.686590	3.033101	0.324019
Pd	1.910212	0.592608	-0.435103
H	2.622658	3.624115	-1.046007
N	2.002368	3.785538	-1.850889
H	1.263677	4.464702	-1.700857
C	1.660029	2.666821	-2.550344
C	2.503128	1.517219	-2.516388
C	0.511958	2.631016	-3.366855
C	2.205285	0.410237	-3.344265
H	3.512465	1.630172	-2.120635
C	0.239200	1.522424	-4.146754
H	-0.149202	3.498750	-3.382153
C	1.076963	0.396798	-4.143495
H	2.893738	-0.434401	-3.339270
H	-0.652434	1.526171	-4.774258
H	0.843766	-0.466010	-4.764513
C	-2.709361	0.005998	-1.563166
C	-2.279332	1.217736	-1.016169
C	-0.942144	1.395207	-0.683653
C	-0.007335	0.374481	-0.904366
C	-0.454099	-0.830933	-1.455682
C	-1.795321	-1.019872	-1.785581
H	-2.997759	2.019144	-0.840624
H	-0.622216	2.349960	-0.258937
H	0.250597	-1.646934	-1.634673
H	-2.121834	-1.964064	-2.220136
C	-4.161326	-0.156961	-1.845084
F	-4.464644	-1.352087	-2.363506
F	-4.620736	0.775193	-2.699139
F	-4.903411	-0.017305	-0.725704
I	1.224221	-0.063496	2.130120
Cs	-2.813654	-0.678747	2.113801
O	-4.117518	2.194517	2.157079
C	-3.301084	3.037727	1.778596
C	-1.989276	3.215290	2.501919
H	-1.837350	4.249454	2.834818
H	-1.994392	2.563277	3.381279
H	-1.134349	2.939800	1.869021
N	-3.541541	3.833589	0.698813
C	-4.795722	3.719580	-0.017409
H	-4.612720	3.501107	-1.080878
H	-5.387760	2.913190	0.420583
H	-5.358317	4.662362	0.042877
C	-2.616340	4.800381	0.146352
H	-1.694197	4.857977	0.727900
H	-2.357074	4.526354	-0.888939
H	-3.077324	5.798440	0.126258
O	-3.348037	-3.273878	0.486857

C	-2.789904	-3.902638	-0.415444
C	-3.576922	-4.452376	-1.577994
H	-3.454788	-5.538973	-1.671138
H	-4.633106	-4.224080	-1.409859
H	-3.271544	-4.004069	-2.532537
N	-1.451531	-4.147802	-0.383867
C	-0.673848	-3.668294	0.741337
H	-0.292613	-2.648490	0.564951
H	-1.286462	-3.666065	1.648074
H	0.184141	-4.334409	0.896165
C	-0.685449	-4.746726	-1.457490
H	-1.316665	-4.991806	-2.313919
H	0.097546	-4.049902	-1.798039
H	-0.193900	-5.668026	-1.112926***

TS c5-c6 | Charge 0 |

Electronic Energy (a.u.) -2158.85692633

C	5.810726	-1.984088	-1.071951
H	6.155538	-2.984429	-1.328998
C	6.568429	-0.872690	-1.325409
H	7.548895	-0.971234	-1.791442
C	4.502495	-1.870153	-0.484403
C	6.081628	0.404701	-0.998770
H	6.663042	1.290606	-1.235397
C	4.835097	0.492013	-0.402712
C	4.232097	1.802066	-0.065503
C	4.958093	2.996109	-0.112474
H	6.016518	2.987720	-0.357929
C	4.325823	4.197155	0.170660
H	4.886415	5.129163	0.136391
C	2.974984	4.191700	0.499007
H	2.435484	5.108795	0.724102
C	2.323703	2.967926	0.546338
H	1.266774	2.907834	0.805819
N	4.084703	-0.602692	-0.130567
N	2.926669	1.807993	0.279705
O	3.765889	-2.866308	-0.294881
Pd	2.080228	-0.312782	0.601651
H	2.264170	-3.623988	0.636943
N	1.597179	-3.797935	1.393905
H	0.759607	-4.315195	1.152276
C	1.471697	-2.779679	2.291719
C	2.501825	-1.800855	2.383340
C	0.386404	-2.694917	3.185110
C	2.441385	-0.809031	3.388407
H	3.446997	-2.023034	1.891319
C	0.345479	-1.696018	4.142915
H	-0.406534	-3.442041	3.128115
C	1.363504	-0.737980	4.254819
H	3.278075	-0.116140	3.483580
H	-0.501361	-1.660327	4.828712
H	1.311557	0.034895	5.019156
C	-2.539098	0.323095	1.695659
C	-2.105076	-0.768114	0.940091
C	-0.756444	-0.927689	0.657716
C	0.190982	-0.009537	1.133662
C	-0.252910	1.051912	1.925785
C	-1.611181	1.226734	2.203633
H	-2.830796	-1.487961	0.559645

H	-0.439995	-1.788878	0.064726
H	0.461613	1.756793	2.355691
H	-1.936767	2.064715	2.819843
C	-3.995874	0.430038	1.985320
F	-4.353099	1.627875	2.459892
F	-4.391484	-0.479309	2.900222
F	-4.743819	0.190073	0.892089
I	1.162406	-0.240281	-2.471725
Cs	-3.109754	0.401867	-2.314500
O	-4.207255	-2.502464	-1.706540
C	-3.255124	-3.196391	-1.340709
C	-2.055114	-3.394406	-2.232606
H	-1.866253	-4.455298	-2.439669
H	-2.243712	-2.884139	-3.182511
H	-1.141911	-2.976744	-1.784978
N	-3.233719	-3.796054	-0.117549
C	-4.337430	-3.611898	0.803306
H	-3.971045	-3.189323	1.751099
H	-5.067538	-2.929646	0.361771
H	-4.820538	-4.574795	1.021659
C	-2.111960	-4.529743	0.429296
H	-1.364742	-4.757751	-0.333586
H	-1.630977	-3.946893	1.233077
H	-2.463629	-5.477010	0.860493
O	-3.547708	2.831664	-0.425295
C	-3.001867	3.681230	0.282210
C	-3.768953	4.423991	1.345275
H	-3.790780	5.502759	1.142607
H	-4.793955	4.044441	1.353262
H	-3.331792	4.284309	2.342255
N	-1.689432	3.994654	0.108052
C	-0.953483	3.262537	-0.906467
H	-0.779611	2.214838	-0.608177
H	-1.497943	3.271676	-1.858537
H	0.018424	3.743115	-1.062136
C	-0.935590	4.883403	0.970081
H	-1.599176	5.548675	1.527066
H	-0.320419	4.324503	1.694026
H	-0.270261	5.508986	0.361728***

c6 | Charge 0 |

Electronic Energy (a.u.) -2158.86049357

C	5.976967	-1.529846	-1.278750
H	6.419577	-2.474898	-1.588656
C	6.527797	-0.327412	-1.627558
H	7.439942	-0.292016	-2.223425
C	4.748780	-1.598426	-0.529069
C	5.902663	0.870815	-1.240265
H	6.292897	1.828951	-1.570098
C	4.758465	0.780780	-0.469322
C	3.999137	1.983234	-0.077971
C	4.527463	3.271199	-0.172362
H	5.552392	3.415113	-0.502696
C	3.750339	4.364505	0.175926
H	4.159588	5.370056	0.107186
C	2.450533	4.154165	0.619763
H	1.801455	4.978983	0.904195
C	1.990373	2.850479	0.711414
H	0.986349	2.635052	1.071173

N	4.227492	-0.403379	-0.074129
N	2.735418	1.790914	0.375821
O	4.179513	-2.689324	-0.294180
Pd	2.324647	-0.234555	0.941589
H	2.483343	-3.476246	0.163594
N	1.740765	-3.741964	0.815872
H	0.942610	-4.260191	0.469411
C	1.616229	-2.991702	1.932730
C	2.643736	-2.055554	2.269249
C	0.521538	-3.136415	2.813855
C	2.571611	-1.361635	3.508248
H	3.610025	-2.203031	1.785917
C	0.474427	-2.415618	3.989992
H	-0.262432	-3.853910	2.570235
C	1.492012	-1.513810	4.353247
H	3.408220	-0.721337	3.788427
H	-0.373781	-2.560146	4.659273
H	1.432779	-0.966561	5.291740
C	-2.372609	0.115803	1.849471
C	-1.844565	-0.774198	0.911097
C	-0.472338	-0.829109	0.705793
C	0.404315	-0.022238	1.447240
C	-0.137347	0.851447	2.393896
C	-1.518433	0.928098	2.592240
H	-2.508389	-1.433820	0.345411
H	-0.080354	-1.533128	-0.031911
H	0.516016	1.480285	3.003754
H	-1.920523	1.615909	3.336282
C	-3.842491	0.097114	2.094277
F	-4.282886	1.188844	2.729825
F	-4.202983	-0.956418	2.858915
F	-4.547530	-0.018683	0.957792
I	1.080050	-0.046098	-2.846462
Cs	-3.164498	0.543953	-2.327067
O	-4.174733	-2.425983	-2.055854
C	-3.211820	-3.078961	-1.646146
C	-1.936351	-3.170457	-2.445109
H	-1.709986	-4.204309	-2.735601
H	-2.051035	-2.573276	-3.355387
H	-1.073800	-2.792073	-1.877065
N	-3.237799	-3.732289	-0.451381
C	-4.372508	-3.579383	0.436543
H	-4.051460	-3.123672	1.384853
H	-5.118054	-2.938251	-0.037990
H	-4.819856	-4.557432	0.659780
C	-2.098038	-4.419105	0.121810
H	-1.436260	-4.819770	-0.650170
H	-1.520040	-3.745630	0.776856
H	-2.456810	-5.260847	0.727364
O	-3.777088	2.655356	-0.114984
C	-3.278179	3.445326	0.689797
C	-4.097411	4.056566	1.796916
H	-4.140899	5.149506	1.703549
H	-5.111864	3.653832	1.730904
H	-3.690731	3.824268	2.788975
N	-1.971507	3.810014	0.586714
C	-1.196651	3.236647	-0.497597
H	-0.950376	2.176867	-0.313509

```
H          -1.747925       3.311162       -1.442389
H          -0.261269       3.796065       -0.604909
C          -1.257875       4.630886        1.544358
H          -1.944681       5.107121        2.246852
H          -0.536751       4.032109        2.123518
H          -0.707104       5.424280        1.021461***
```

# (CsI 2DMA) | Charge 0 |

```
Electronic Energy (a.u.)     -606.636679741
I          -2.033357      -0.490641       -1.766142
Cs          0.647093       2.164818       -0.063016
O          -0.307871       0.110364        2.125784
C          -1.535613      -0.027077        2.135028
C          -2.441199       1.175181        2.239250
H          -3.215389       1.057089        3.006896
H          -1.827355       2.045446        2.491998
H          -2.946209       1.369342        1.282155
N          -2.123602      -1.248420        2.053710
C          -1.305920      -2.441046        1.979846
H          -1.289511      -2.843138        0.953799
H          -0.285398      -2.204571        2.290098
H          -1.719647      -3.211211        2.645562
C          -3.549739      -1.480254        1.952240
H          -4.095924      -0.560480        1.732226
H          -3.744224      -2.186124        1.132458
H          -3.946806      -1.916787        2.880881
O           3.219633       0.466038        0.427811
C           3.780289      -0.611993        0.219118
C           5.268947      -0.763892        0.400294
H           5.768696      -0.987452       -0.551503
H           5.662393       0.179825        0.788279
H           5.519766      -1.567982        1.103331
N           3.072068      -1.703071       -0.175175
C           1.649294      -1.531933       -0.414476
H           1.159802      -1.057283        0.446550
H           1.461718      -0.917936       -1.309352
H           1.197484      -2.516477       -0.575127
C           3.655757      -2.954415       -0.616226
H           4.698449      -3.038683       -0.303017
H           3.102062      -3.796063       -0.180527
H           3.613676      -3.046724       -
1.712269*************************************************
```

Arylation reaction of aniline with the simplest model (model 1). Gibbs energy profile in Figure S89.

```
# TS 8-c1ortho | Charge                          0              |
Electronic Energy (a.u.)    -1552.19522172
 C          5.243924       -1.187067       -0.233928
H           5.868122       -2.068181       -0.371479
C           5.774784        0.034812        0.069548
H           6.851513        0.155641        0.184921
C           3.825205       -1.360703       -0.405518
C           4.930726        1.148492        0.222886
H           5.354141        2.123709        0.439316
C           3.569099        0.956671        0.080730
C           2.621404        2.082752        0.190115
C           3.034895        3.392931        0.438730
H           4.085565        3.612264        0.601276
C           2.109545        4.422077        0.473240
H           2.435817        5.441837        0.664522
C           0.769235        4.132269        0.251081
H           0.005504        4.904972        0.253011
C           0.408610        2.815666        0.028127
H          -0.629915        2.544883       -0.139628
C          -1.102086        0.123824       -0.148148
C          -1.823245        0.124686       -1.346508
C          -3.191136        0.367675       -1.334543
C          -3.842165        0.619542       -0.124594
C          -3.127936        0.632855        1.069652
C          -1.755093        0.390022        1.055419
N           3.023699       -0.256617       -0.198153
N           1.302720        1.813255        0.008094
O           3.335791       -2.470177       -0.725166
Pd          0.851060       -0.212946       -0.191843
C          -5.313601        0.858274       -0.158194
F          -5.824108        1.153506        1.042854
F          -5.638938        1.869955       -0.981973
F          -5.981037       -0.216596       -0.614254
H          -1.198952        0.412860        1.993967
H          -3.635935        0.836387        2.010558
H          -3.758254        0.360092       -2.266039
H          -1.321766       -0.083644       -2.292911
H           1.679159       -2.587588       -1.512503
N           0.660509       -2.471649       -1.593066
H           0.238985       -2.825318       -2.445878
C          -0.041003       -2.727234       -0.419216
C           0.610286       -2.629167        0.831849
C          -1.415180       -3.029476       -0.443686
C          -0.125757       -2.781259        2.012599
H           1.698129       -2.558515        0.854310
C          -2.118649       -3.190882        0.737652
H          -1.916194       -3.123754       -1.407929
C          -1.486349       -3.051307        1.979262
H           0.395849       -2.715012        2.967073
H          -3.181875       -3.426191        0.694492
H          -2.049818       -3.176916        2.901919***
 # TS c1ortho-c1para | Charge                          0
| Electronic Energy (a.u.)    -1552.19412254
```

C	5.405064	-1.244159	0.113960
H	6.040833	-2.127942	0.120886
C	5.915746	0.015190	0.232054
H	6.989054	0.172370	0.339481
C	3.984874	-1.473315	-0.018369
C	5.047690	1.121457	0.216108
H	5.447036	2.124744	0.322368
C	3.691345	0.887174	0.080921
C	2.738852	2.015777	0.061507
C	3.157119	3.348714	0.049769
H	4.215334	3.588397	0.024942
C	2.226323	4.373253	0.058448
H	2.556207	5.409669	0.048741
C	0.874020	4.055048	0.078068
H	0.102225	4.820180	0.090581
C	0.513950	2.719757	0.070723
H	-0.533352	2.430578	0.074261
C	-0.992730	0.069602	-0.056111
C	-1.662843	0.397160	-1.240342
C	-3.007887	0.746589	-1.209912
C	-3.692298	0.774362	0.006802
C	-3.027693	0.475960	1.193431
C	-1.678198	0.129612	1.159012
N	3.157498	-0.359988	-0.027069
N	1.414320	1.722132	0.056042
O	3.531681	-2.630055	-0.118928
Pd	0.955051	-0.328956	-0.079820
C	-5.130755	1.165147	-0.002991
F	-5.710124	1.050275	1.198240
F	-5.300744	2.440458	-0.398425
F	-5.847917	0.416263	-0.859243
H	-1.168647	-0.109319	2.093913
H	-3.557814	0.509387	2.143724
H	-3.531180	0.996845	-2.133480
H	-1.137575	0.378601	-2.196094
H	-3.332739	-2.986108	1.820211
N	-2.350740	-3.228232	1.908814
C	-1.593897	-3.013937	0.768258
C	-2.186457	-3.049453	-0.504017
C	-0.209740	-2.810871	0.848566
C	-1.436300	-2.837865	-1.655634
H	-3.261075	-3.225196	-0.576324
C	0.559795	-2.619150	-0.312146
H	0.277752	-2.830314	1.825245
C	-0.065354	-2.614283	-1.575141
H	-1.934601	-2.851018	-2.624405
H	1.655924	-2.684422	-0.244246
H	0.533476	-2.484083	-2.475754
H	-1.948890	-2.850892	2.761640***

TS c1$_{ortho}$-c2$_{ortho}$ | Charge 0 | Electronic Energy (a.u.) -1552.18385496

C	-5.046227	-1.375910	-0.360747
H	-5.574392	-2.319107	-0.479169
C	-5.706688	-0.176301	-0.265375
H	-6.794496	-0.149815	-0.308178
C	-3.623650	-1.401870	-0.310522
C	-4.991562	1.022068	-0.115343
H	-5.518441	1.968788	-0.045739

C	-3.611800	0.952106	-0.064792
C	-2.746817	2.140285	0.086865
C	-3.251527	3.429472	0.246162
H	-4.323848	3.601777	0.269369
C	-2.377178	4.498385	0.379562
H	-2.765978	5.506523	0.505228
C	-1.008485	4.264236	0.350531
H	-0.288242	5.071634	0.451657
C	-0.568190	2.959338	0.191381
H	0.493692	2.720341	0.161111
C	1.155567	0.161624	-0.075478
C	1.948677	-0.104722	1.051609
C	3.307016	0.193639	1.061709
C	3.905727	0.755042	-0.066655
C	3.143506	1.011883	-1.203686
C	1.782570	0.716390	-1.201449
N	-2.956799	-0.231099	-0.156784
N	-1.407370	1.925287	0.062773
O	-2.967892	-2.497847	-0.409894
Pd	-0.830730	-0.120872	-0.107637
C	5.373739	1.007314	-0.052050
F	5.770731	1.817887	-1.041982
F	5.779519	1.566491	1.100348
F	6.083424	-0.130801	-0.178923
H	1.200397	0.932257	-2.100124
H	3.610225	1.442681	-2.088236
H	3.906720	-0.010977	1.949125
H	1.502025	-0.548722	1.943468
C	0.369554	-2.601137	-1.368746
C	0.001397	-2.817936	1.020138
C	1.690298	-4.140005	-0.094395
C	1.374975	-3.548109	-1.327698
H	0.105675	-2.142721	-2.324352
H	2.478045	-4.891450	-0.042929
C	-0.349295	-2.198426	-0.215106
H	-1.657628	-2.267326	-0.361250
C	1.014782	-3.794965	1.061336
H	1.267464	-4.264512	2.012929
H	1.908138	-3.841797	-2.230213
N	-0.622591	-2.443015	2.180917
H	-1.481976	-1.911077	2.084247
H	-0.624734	-3.108410	2.946675***

TS c1$_{para}$-c2$_{para}$ | Charge 0
| Electronic Energy (a.u.) -1552.18092075

C	5.053775	1.681846	-0.319738
H	5.516489	2.657173	-0.450667
C	5.794826	0.531561	-0.209215
H	6.881928	0.578812	-0.251575
C	3.633462	1.610998	-0.266992
C	5.162264	-0.710634	-0.047597
H	5.751031	-1.619591	0.028679
C	3.780784	-0.733868	0.002424
C	2.999009	-1.979286	0.149380
C	3.590628	-3.231162	0.308518
H	4.672045	-3.329425	0.339940
C	2.791264	-4.358533	0.429967
H	3.247921	-5.337986	0.554450
C	1.409933	-4.219335	0.389969

```
H          0.746627      -5.075222       0.481476
C          0.882022      -2.947242       0.231753
H         -0.193787      -2.782798       0.194161
C         -1.024436      -0.292143      -0.056953
C         -1.829835      -0.220326       1.090498
C         -3.149836      -0.658747       1.073585
C         -3.697695      -1.173948      -0.101450
C         -2.923828      -1.239681      -1.258391
C         -1.602152      -0.803452      -1.229508
N          3.046726       0.401662      -0.095183
N          1.648727      -1.857125       0.113681
O          2.905358       2.660047      -0.380973
Pd         0.931771       0.149394      -0.047938
C         -5.131701      -1.576579      -0.117476
F         -5.433359      -2.377040      -1.148956
F         -5.488604      -2.227298       1.002180
F         -5.954747      -0.513392      -0.205292
H         -1.009838      -0.874677      -2.144371
H         -3.350710      -1.635843      -2.178713
H         -3.756583      -0.602374       1.977655
H         -1.423933       0.179218       2.021185
C         -0.519125       2.581539      -1.179669
C         -0.020895       2.717455       1.164642
C         -1.930733       3.900767       0.274954
C         -1.609514       3.409413      -1.008771
H         -0.301884       2.211676      -2.184301
H          0.608911       2.469379       2.022731
C          0.324073       2.193561      -0.107529
H          1.643266       2.357955      -0.305334
C         -1.107134       3.545006       1.364426
H         -1.341449       3.941699       2.353275
H         -2.237203       3.696118      -1.853653
N         -3.033803       4.680073       0.464140
H         -3.099713       5.213246       1.323297
H         -3.449568       5.124184      -0.346116***
```

TS c1$_{ortho}$-c2$_{meta}$ | Charge 0

```
| Electronic Energy (a.u.)    -1552.17364914
C          5.088840       1.551864      -0.237798
H          5.588337       2.515006      -0.310676
C          5.786447       0.369455      -0.215508
H          6.873954       0.380418      -0.270413
C          3.666806       1.531549      -0.168883
C          5.111698      -0.858785      -0.129481
H          5.666912      -1.791775      -0.123215
C          3.731533      -0.834352      -0.060122
C          2.896824      -2.050361       0.009994
C          3.431848      -3.335810       0.056223
H          4.508276      -3.482641       0.051875
C          2.583707      -4.432390       0.108724
H          2.996619      -5.438127       0.144224
C          1.210063      -4.228366       0.114197
H          0.509448      -5.057958       0.154705
C          0.737360      -2.926032       0.068511
H         -0.330206      -2.712902       0.071118
C         -1.035003      -0.217194       0.025473
C         -1.791262      -0.271260       1.204902
C         -3.123397      -0.673165       1.184232
```

C	-3.725353	-1.026967	-0.023190
C	-2.995438	-0.966559	-1.209113
C	-1.663388	-0.564334	-1.179094
N	3.043006	0.332253	-0.066872
N	1.551551	-1.865055	0.016131
O	2.967991	2.602915	-0.204144
Pd	0.933112	0.167081	0.016170
C	-5.170201	-1.390938	-0.037748
F	-5.518571	-2.078448	-1.134020
F	-5.516388	-2.139525	1.022062
F	-5.965353	-0.304224	-0.000105
H	-1.105111	-0.531578	-2.117026
H	-3.464846	-1.237747	-2.153708
H	-3.695490	-0.715340	2.111167
H	-1.339073	-0.005631	2.161438
C	-0.496384	2.720543	-0.861989
C	0.122853	2.663465	1.474026
C	-1.818494	3.906311	0.761426
C	-1.596277	3.537952	-0.575413
H	-0.326873	2.425013	-1.900792
H	0.803222	2.346985	2.266725
H	-2.671079	4.545164	0.996726
C	0.366557	2.256584	0.145887
H	1.668504	2.299762	-0.107937
C	-0.963690	3.478086	1.772586
H	-1.152334	3.795696	2.797975
N	-2.485102	3.920407	-1.571953
H	-3.004728	4.770045	-1.375771
H	-2.089876	3.937324	-2.506694

***

c2$_{ortho}$ | Charge 0 |

Electronic Energy (a.u.) -1552.20525891

C	5.179169	0.654067	-0.173998
H	5.850079	1.506241	-0.239976
C	5.630149	-0.640862	-0.055915
H	6.697494	-0.848318	-0.024020
C	3.793412	0.867829	-0.211927
C	4.714404	-1.692936	0.017084
H	5.073634	-2.713186	0.102651
C	3.359539	-1.406903	-0.023196
C	2.327685	-2.464729	0.035128
C	2.646499	-3.818779	0.134567
H	3.680374	-4.147686	0.181411
C	1.627741	-4.760130	0.172776
H	1.867965	-5.818206	0.249764
C	0.307628	-4.334576	0.109965
H	-0.521363	-5.036843	0.135365
C	0.061567	-2.972965	0.014060
H	-0.955837	-2.586377	-0.038778
C	-1.290772	0.036306	-0.079307
C	-2.033795	0.179646	1.104226
C	-3.407148	-0.033516	1.119384
C	-4.072553	-0.380830	-0.057668
C	-3.361200	-0.506091	-1.247636
C	-1.984387	-0.294030	-1.252825
N	2.909354	-0.128286	-0.128958
N	1.039449	-2.061849	-0.022627

O	3.360006	2.114682	-0.342196
Pd	0.707492	0.086520	-0.108666
C	-5.542251	-0.613857	0.003871
F	-6.069326	-0.941888	-1.182260
F	-5.857266	-1.605204	0.858391
F	-6.208287	0.470246	0.441414
H	-1.444002	-0.405751	-2.194856
H	-3.878333	-0.771096	-2.168349
H	-3.968694	0.071211	2.048700
H	-1.533495	0.464370	2.031211
C	0.495603	2.882578	1.013974
C	0.401135	2.107164	-0.167009
H	2.370854	2.115876	-0.371450
C	0.312009	4.272493	0.957053
H	0.394985	4.853670	1.877778
C	-0.076412	4.158317	-1.420855
H	-0.295073	4.645813	-2.370391
C	0.028664	4.903147	-0.248813
H	-0.106878	5.984353	-0.269554
C	0.103102	2.774600	-1.361979
H	0.018251	2.196078	-2.285242
N	0.740542	2.269648	2.242342
H	1.204113	1.367310	2.161433
H	1.190969	2.864508	2.931531***

c2$_{para}$ | Charge 0 |

Electronic Energy (a.u.) -1552.20096873

C	-5.212762	0.553899	0.097876
H	-5.880851	1.410343	0.127941
C	-5.666947	-0.745387	0.105765
H	-6.734259	-0.952219	0.145871
C	-3.827102	0.766615	0.051517
C	-4.754412	-1.801951	0.063518
H	-5.115500	-2.825336	0.075354
C	-3.399626	-1.515963	0.013208
C	-2.370595	-2.577070	-0.032808
C	-2.693508	-3.932401	-0.094867
H	-3.729047	-4.258079	-0.121493
C	-1.677318	-4.876611	-0.128148
H	-1.920554	-5.935686	-0.177251
C	-0.355542	-4.452250	-0.098159
H	0.471575	-5.156803	-0.121897
C	-0.105817	-3.089144	-0.041084
H	0.913137	-2.703481	-0.018161
C	1.261311	-0.090679	0.024686
C	1.999253	-0.154778	-1.169069
C	3.369067	-0.389596	-1.156872
C	4.037437	-0.550123	0.058117
C	3.332214	-0.468774	1.255548
C	1.958807	-0.234904	1.233051
N	-2.945884	-0.234601	0.009789
N	-1.081398	-2.175320	-0.010303
O	-3.389754	2.018779	0.052009
Pd	-0.735410	-0.025868	0.006292
C	5.499779	-0.830366	0.027686
F	6.052352	-0.864274	1.246684
F	5.770068	-2.013807	-0.555121
F	6.176018	0.089705	-0.683542
H	1.425600	-0.175030	2.183722

H	3.851796	-0.587251	2.205127
H	3.925554	-0.448431	-2.093266
H	1.498052	-0.028391	-2.130409
C	-0.230505	2.727781	1.165826
C	-0.383762	2.712506	-1.217507
C	-0.033041	4.815245	-0.056618
C	-0.050708	4.108092	1.153884
H	-0.235289	2.218795	2.132733
H	-0.517859	2.191490	-2.168897
C	-0.413077	1.985900	-0.013916
H	-2.401204	2.018744	0.023421
C	-0.204967	4.093326	-1.245314
H	-0.196224	4.627565	-2.197393
H	0.082478	4.654396	2.089732
N	0.208837	6.185879	-0.082037
H	-0.159902	6.659396	-0.900948
H	-0.056502	6.673146	0.768059***

c2$_{meta}$ | Charge 0 |

Electronic Energy (a.u.) -1552.20232568

C	-5.212186	0.542894	-0.023484
H	-5.890678	1.390503	-0.067317
C	-5.648791	-0.754431	0.118611
H	-6.712345	-0.969531	0.196537
C	-3.830363	0.766673	-0.111218
C	-4.722079	-1.798309	0.162982
H	-5.067843	-2.820183	0.281328
C	-3.372403	-1.502437	0.062618
C	-2.333156	-2.553759	0.095229
C	-2.644578	-3.912439	0.140340
H	-3.677583	-4.247295	0.142504
C	-1.620199	-4.847616	0.174972
H	-1.853966	-5.909384	0.209470
C	-0.302125	-4.410865	0.163949
H	0.531211	-5.107968	0.190079
C	-0.064159	-3.045432	0.111945
H	0.951411	-2.650609	0.097917
C	1.276095	-0.036479	-0.052930
C	2.016837	-0.183202	-1.237375
C	3.389747	-0.397350	-1.205639
C	4.057352	-0.454095	0.018922
C	3.348557	-0.290612	1.205792
C	1.972148	-0.077766	1.163839
N	-2.934654	-0.221441	-0.067200
N	-1.047888	-2.140411	0.077029
O	-3.416019	2.020675	-0.241777
Pd	-0.723601	0.006429	-0.081599
C	5.523869	-0.714134	0.012013
F	6.074149	-0.643485	1.230510
F	5.812912	-1.935584	-0.475420
F	6.187532	0.157159	-0.768910
H	1.436175	0.048302	2.106428
H	3.867646	-0.328681	2.162224
H	3.948976	-0.520434	-2.134101
H	1.515794	-0.138567	-2.205993
C	-0.282496	2.821650	0.868264
C	-0.412541	2.617742	-1.528607
C	-0.161782	4.803699	-0.509075
C	-0.153815	4.216887	0.765430

H	-0.277208	2.382230	1.870194
H	-0.516801	2.015642	-2.433382
H	-0.066274	5.886456	-0.600969
C	-0.416636	2.005312	-0.262013
H	-2.431968	2.038351	-0.291882
C	-0.290786	4.004374	-1.637642
H	-0.299628	4.472067	-2.623100
N	0.035179	4.996057	1.900458
H	-0.253516	5.964199	1.801946
H	-0.328452	4.589698	2.756716

***

TS RE$_{ortho}$

Charge 0	Electronic Energy (a.u.) -1552.18724283		
C	5.142452	0.812908	-0.271678
H	5.764687	1.700723	-0.345429
C	5.666227	-0.463786	-0.256681
H	6.742441	-0.610489	-0.321401
C	3.750096	0.941219	-0.193451
C	4.820192	-1.571633	-0.163033
H	5.238376	-2.573791	-0.161743
C	3.451693	-1.357368	-0.081746
C	2.462017	-2.455964	0.020544
C	2.834707	-3.795826	0.126027
H	3.881971	-4.085053	0.143023
C	1.848365	-4.768663	0.217740
H	2.124722	-5.817493	0.302498
C	0.512380	-4.387352	0.201409
H	-0.289938	-5.117355	0.271803
C	0.220068	-3.033998	0.096039
H	-0.812073	-2.678677	0.079944
C	-1.298298	0.508372	-0.034374
C	-2.031357	0.303467	1.156648
C	-3.343878	-0.151517	1.133526
C	-3.971594	-0.433808	-0.077847
C	-3.274225	-0.233999	-1.273576
C	-1.972065	0.234553	-1.250168
N	2.938603	-0.107645	-0.096974
N	1.164381	-2.094893	0.008239
O	3.210826	2.160110	-0.217790
Pd	0.693323	0.121833	-0.044030
C	-5.388935	-0.881712	-0.109350
F	-5.637016	-1.716655	-1.132145
F	-5.750118	-1.520391	1.014217
F	-6.251489	0.144647	-0.251456
H	-1.457309	0.381753	-2.199776
H	-3.752442	-0.456903	-2.227055
H	-3.876543	-0.305376	2.071391
H	-1.570095	0.509236	2.119863
C	-0.129711	2.710057	-1.318737
C	0.043471	2.829296	1.089282
C	0.078588	4.854315	-0.257860
H	-0.232759	2.125546	-2.233743
H	0.147160	5.940082	-0.312335
C	-0.102180	2.034485	-0.082700
H	2.230402	2.053829	-0.192616
C	0.115018	4.225825	0.979426

H	0.226844	4.813857	1.892306
C	-0.032201	4.092744	-1.420872
H	-0.048362	4.570660	-2.399415
N	0.100847	2.243899	2.348409
H	0.498029	1.307098	2.351873
H	0.521244	2.829542	3.063829***

TS RE*meta* | Charge 0 |

Electronic Energy (a.u.)	-1552.1866159		
C	-5.147142	0.692120	-0.157208
H	-5.776145	1.575385	-0.228968
C	-5.663879	-0.582699	-0.050962
H	-6.741290	-0.734100	-0.036829
C	-3.753234	0.827179	-0.168946
C	-4.807498	-1.682693	0.039382
H	-5.219177	-2.683702	0.125234
C	-3.437780	-1.463037	0.020062
C	-2.441498	-2.557412	0.111867
C	-2.804100	-3.901363	0.206349
H	-3.848844	-4.200051	0.212253
C	-1.810557	-4.867523	0.292299
H	-2.079681	-5.919134	0.366617
C	-0.477395	-4.475896	0.281899
H	0.330557	-5.200080	0.347800
C	-0.195215	-3.120284	0.183120
H	0.834065	-2.757287	0.168864
C	1.310204	0.390526	-0.092393
C	2.024778	0.088480	-1.271847
C	3.346255	-0.326803	-1.227288
C	4.005415	-0.441361	-0.000472
C	3.328819	-0.133658	1.178364
C	2.004238	0.282212	1.129205
N	-2.930491	-0.214166	-0.083361
N	-1.146612	-2.187754	0.099999
O	-3.227743	2.047864	-0.269406
Pd	-0.679624	0.013555	-0.080975
C	5.426603	-0.880281	0.001155
F	5.929696	-1.015611	1.234411
F	5.588897	-2.063669	-0.620356
F	6.224924	-0.015071	-0.653000
H	1.497536	0.526982	2.063326
H	3.834751	-0.221900	2.138288
H	3.875415	-0.568520	-2.149972
H	1.533685	0.178514	-2.241187
C	-0.009645	2.729202	0.913482
C	0.023811	2.541358	-1.494250
C	-0.227525	4.714413	-0.446576
C	-0.160482	4.120417	0.823644
H	0.041222	2.281125	1.908809
H	0.099912	1.945791	-2.404260
H	-0.344540	5.795652	-0.526447
C	0.075477	1.919014	-0.231069
H	-2.246970	1.958478	-0.257755
C	-0.135717	3.922070	-1.585075
H	-0.192960	4.393149	-2.566856
N	-0.192101	4.902248	1.970392
H	-0.670421	5.791266	1.865854
H	-0.493258	4.414957	2.808177***

TS RE$_{para}$ | Charge 0 |

Electronic Energy (a.u.)	-1552.18597215		
C	-5.130435	0.753299	-0.031546
H	-5.736662	1.655253	-0.039374
C	-5.679911	-0.512526	-0.030959
H	-6.760909	-0.636429	-0.038713
C	-3.733619	0.852745	-0.021354
C	-4.852887	-1.638366	-0.020458
H	-5.290603	-2.632039	-0.019962
C	-3.477939	-1.452685	-0.010550
C	-2.508169	-2.573978	0.001482
C	-2.904970	-3.911371	0.008500
H	-3.957337	-4.181917	0.005299
C	-1.936874	-4.906611	0.020199
H	-2.232961	-5.953517	0.025977
C	-0.594198	-4.549242	0.024483
H	0.195215	-5.296469	0.033543
C	-0.277079	-3.197676	0.017072
H	0.761177	-2.861464	0.020216
C	1.310385	0.286240	0.016938
C	2.025240	0.085437	-1.185756
C	3.350848	-0.316852	-1.179693
C	4.018704	-0.524817	0.030796
C	3.341431	-0.324705	1.232668
C	2.012128	0.077645	1.222954
N	-2.939204	-0.213478	-0.011230
N	-1.203834	-2.236704	0.005973
O	-3.174318	2.062370	-0.021746
Pd	-0.680520	-0.045120	0.004569
C	5.443860	-0.943816	-0.010474
F	5.955821	-1.180176	1.204294
F	5.619149	-2.066616	-0.733772
F	6.230175	-0.015369	-0.589805
H	1.506795	0.234644	2.176695
H	3.851065	-0.488010	2.180912
H	3.877704	-0.477305	-2.121406
H	1.529619	0.248738	-2.143464
C	0.041625	2.600557	1.207991
C	0.057543	2.601781	-1.187423
C	-0.117322	4.705575	0.010204
C	-0.067191	3.984900	1.213432
H	0.078808	2.079161	2.166217
H	0.106843	2.081442	-2.145675
C	0.092101	1.861494	0.010150
H	-2.195776	1.943129	-0.013880
C	-0.050985	3.986333	-1.192899
H	-0.085559	4.530451	-2.138326
H	-0.114351	4.528004	2.158903
N	-0.162752	6.091883	0.010828
H	-0.551343	6.505702	-0.830671
H	-0.562750	6.504562	0.847520***

c3$_{ortho}$ | Charge 0 |

Electronic Energy (a.u.)	-1552.23173782		
C	2.184822	-4.225183	-0.057021
H	3.217652	-4.561596	-0.088549
C	1.126511	-5.090747	0.138512
H	1.311255	-6.155926	0.262858
C	1.889945	-2.869186	-0.231106

C	-0.181090	-4.602093	0.161000
H	-1.022941	-5.279339	0.277674
C	-0.385922	-3.239164	-0.001245
C	-1.737454	-2.633628	0.001189
C	-2.805768	-3.226329	0.675610
H	-2.667082	-4.151878	1.229911
C	-4.043390	-2.594564	0.660379
H	-4.887426	-3.033174	1.189035
C	-4.180391	-1.392767	-0.023510
H	-5.126617	-0.857262	-0.053324
C	-3.063596	-0.875398	-0.670351
H	-3.125827	0.067778	-1.217236
C	1.008786	1.821699	0.105963
C	0.289413	2.065104	1.319560
C	-1.049042	2.383855	1.315840
C	-1.759989	2.463843	0.099197
C	-1.118021	2.217394	-1.096021
C	0.258302	1.880316	-1.111124
N	0.648366	-2.387676	-0.185930
N	-1.871820	-1.472845	-0.663538
O	2.887292	-2.011994	-0.455585
Pd	0.305710	-0.208817	-0.535331
C	-3.213631	2.774538	0.156360
F	-3.789245	2.813041	-1.051840
F	-3.890603	1.860719	0.880439
F	-3.454998	3.958599	0.746515
H	0.784331	1.893897	-2.068036
H	-1.654427	2.291710	-2.040237
H	-1.562675	2.591956	2.254388
H	0.833976	2.049251	2.262876
C	3.149469	2.714494	-0.810467
C	3.278619	1.122138	1.006413
C	5.297484	2.078279	0.046132
H	2.544476	3.309335	-1.495509
H	6.384729	2.146238	0.035202
C	2.495113	1.876305	0.099219
H	2.475790	-1.115757	-0.547012
C	4.674021	1.237788	0.958983
H	5.267719	0.642759	1.654469
C	4.535362	2.823482	-0.850210
H	5.011821	3.489506	-1.567504
N	2.690056	0.284854	1.949138
H	1.792348	-0.105153	1.658678
H	3.312000	-0.443456	2.288727***

c3$_{meta}$ | Charge 0 |

Electronic Energy (a.u.) -1552.23072478

C	-4.394870	-2.783408	-0.223478
H	-5.476478	-2.753043	-0.324061
C	-3.679528	-3.963356	-0.237318
H	-4.197414	-4.914511	-0.343117
C	-3.682527	-1.588719	-0.079462
C	-2.290008	-3.931887	-0.109341
H	-1.710591	-4.851393	-0.096366
C	-1.655732	-2.706423	0.034919
C	-0.182112	-2.634652	0.184110
C	0.636498	-3.493701	-0.551392
H	0.201713	-4.186960	-1.268631
C	2.012588	-3.431976	-0.365577

H	2.673182	-4.086435	-0.931214
C	2.524772	-2.521089	0.548134
H	3.593919	-2.436298	0.728525
C	1.631070	-1.696237	1.226893
H	2.002931	-0.960992	1.943829
C	1.020024	1.771874	-0.378526
C	1.527621	0.920974	-1.372228
C	2.813468	0.406586	-1.282958
C	3.618866	0.745589	-0.198839
C	3.141872	1.610906	0.784037
C	1.855584	2.119609	0.692921
N	-2.352158	-1.544927	0.052075
N	0.309442	-1.739050	1.055175
O	-4.354632	-0.438577	-0.081383
Pd	-1.451142	0.429551	0.160927
C	4.982603	0.169884	-0.024523
F	5.934051	1.117093	-0.004577
F	5.090080	-0.485681	1.148904
F	5.309164	-0.699025	-0.986747
H	1.499170	2.807248	1.458049
H	3.779780	1.883727	1.624481
H	3.180693	-0.274649	-2.047786
H	0.893385	0.630432	-2.209025
C	-1.113908	2.564757	0.718453
C	-0.908785	2.690892	-1.715371
C	-2.831152	3.672215	-0.601301
C	-2.350471	3.266152	0.645076
H	-0.656734	2.448307	1.703838
H	-0.340378	2.513865	-2.627178
H	-3.769009	4.225549	-0.655153
C	-0.369518	2.290413	-0.463504
H	-3.678922	0.288062	0.004772
C	-2.108505	3.381317	-1.761567
H	-2.498225	3.711281	-2.724152
N	-2.998593	3.615617	1.814993
H	-3.986322	3.825589	1.715091
H	-2.818721	3.008228	2.607665***

c3$_{para}$ | Charge 0 |

Electronic Energy (a.u.)	-1552.22946506		
C	-4.207410	-3.058155	0.082360
H	-5.293554	-3.084008	0.106488
C	-3.433907	-4.193499	0.213780
H	-3.907627	-5.164955	0.340199
C	-3.550666	-1.834750	-0.081682
C	-2.042356	-4.090990	0.176602
H	-1.413685	-4.974241	0.256391
C	-1.465417	-2.841428	0.002479
C	0.008945	-2.694176	-0.060993
C	0.822169	-3.432376	0.800178
H	0.378513	-4.086508	1.548149
C	2.202192	-3.298654	0.698358
H	2.858799	-3.855796	1.364052
C	2.722813	-2.441846	-0.261580
H	3.795332	-2.303663	-0.378977
C	1.832174	-1.742387	-1.072666
H	2.209546	-1.054437	-1.832337
C	0.931516	1.818055	0.182887
C	1.763450	2.104623	-0.911199

C	3.081800	1.677497	-0.935059
C	3.602288	0.960247	0.140793
C	2.801590	0.685948	1.246918
C	1.482152	1.113935	1.266598
N	-2.218377	-1.723200	-0.124546
N	0.506445	-1.852219	-0.981130
O	-4.278824	-0.724651	-0.192275
Pd	-1.408624	0.283338	-0.244485
C	5.004920	0.468414	0.044397
F	5.379790	-0.252570	1.106663
F	5.177627	-0.314455	-1.040461
F	5.887116	1.473005	-0.085395
H	0.857732	0.867319	2.124550
H	3.200297	0.118529	2.085485
H	3.713187	1.902335	-1.794570
H	1.377425	2.682094	-1.749550
C	-1.112482	2.679712	1.398926
C	-1.222751	2.443898	-1.009775
C	-3.042137	3.532856	0.193533
C	-2.336392	3.317271	1.396269
H	-0.579430	2.565526	2.342695
H	-0.755545	2.244517	-1.974994
C	-0.487642	2.250622	0.194538
H	-3.635856	0.030213	-0.280840
C	-2.478762	3.074572	-0.997955
H	-3.007830	3.234332	-1.938165
H	-2.771515	3.664669	2.333921
N	-4.239619	4.229459	0.202572
H	-4.841533	4.059722	-0.596624
H	-4.753415	4.189582	1.076835***

Buchwald-Hartwig amination (C-N coupling). Gibbs energy profile in Figure S93.

```
# 8NH | Charge                          0              |
Electronic Energy (a.u.)    -3006.38440284
C           3.912150       5.225791      -3.087549
H           4.350111       5.165760      -4.082689
C           3.734965       6.418505      -2.446789
H           4.035404       7.352782      -2.921316
C           3.510729       3.979634      -2.480685
C           3.158176       6.442490      -1.165130
H           3.012785       7.387476      -0.652048
C           2.797793       5.237159      -0.588894
C           2.196221       5.199564       0.762024
C           1.916583       6.355151       1.497219
H           2.126227       7.335394       1.080129
C           1.369335       6.257008       2.766045
H           1.151723       7.157370       3.336921
C           1.107316       4.997451       3.292729
H           0.681560       4.865842       4.284415
C           1.396198       3.890121       2.513926
H           1.205628       2.883440       2.880941
C           1.697224       0.951372       1.325990
C           0.472175       0.305115       1.096105
C          -0.024572      -0.629623       1.995247
C           0.694716      -0.946116       3.149849
C           1.908879      -0.312386       3.403894
C           2.396281       0.630371       2.498884
N           2.972025       4.038278      -1.205557
N           1.919697       3.979395       1.281779
O           3.642235       2.895930      -3.089191
Pd          2.358865       2.328607       0.036095
C           0.139358      -1.986979       4.057554
F           0.811950      -2.096240       5.208862
F          -1.147687      -1.745436       4.372769
F           0.148776      -3.209249       3.485385
H           3.349557       1.117081       2.717566
H           2.473289      -0.551443       4.304129
H          -0.974674      -1.129066       1.793543
H          -0.098935       0.530042       0.193146
H           3.125852       1.422962      -2.070610
N           2.825801       0.818226      -1.295747
H           1.390388      -0.248706      -1.755181
C           3.860634      -0.068135      -1.031688
C           5.078689      -0.000913      -1.747661
C           3.725194      -1.134276      -0.112678
C           6.087984      -0.939545      -1.558917
H           5.210887       0.816830      -2.459014
C           4.741891      -2.060985       0.078962
H           2.800329      -1.214709       0.456564
C           5.936130      -1.978997      -0.640924
H           7.012992      -0.850165      -2.131029
H           4.597058      -2.866602       0.801348
H           6.731939      -2.707812      -0.488047
O          -1.335040      -1.589408      -1.664859
O           0.805415      -1.050568      -1.855123
C          -0.512835      -0.657067      -1.834719
O          -0.747943       0.567078      -1.972671
Cs          0.664891      -3.533195      -0.017632
```

Cs	-3.632498	0.460437	-0.872885
O	-1.825560	-4.417672	1.612087
C	-2.650121	-3.647537	2.107765
C	-3.167427	-2.465274	1.323758
H	-3.160040	-1.539194	1.915238
H	-2.534239	-2.337972	0.434760
H	-4.201826	-2.636250	0.992532
N	-3.138795	-3.823004	3.364561
C	-2.583871	-4.847596	4.223110
H	-3.362098	-5.563751	4.522657
H	-1.791874	-5.375496	3.688473
H	-2.167491	-4.391199	5.133501
C	-4.124794	-2.968051	3.991375
H	-4.728379	-2.438194	3.250495
H	-4.803824	-3.587815	4.591556
H	-3.652786	-2.232942	4.661158
O	-4.565017	2.285712	1.524500
C	-4.142709	3.442131	1.598319
C	-5.087842	4.615454	1.566407
H	-4.943955	5.220081	0.661270
H	-6.110570	4.228416	1.567230
H	-4.954188	5.276350	2.431794
N	-2.813242	3.717638	1.705767
C	-1.853336	2.640391	1.830242
H	-1.233196	2.790156	2.727020
H	-2.381832	1.687891	1.925281
H	-1.187053	2.607830	0.953203
C	-2.248962	5.049016	1.798894
H	-2.932387	5.805222	1.406443
H	-1.996325	5.304534	2.839697
H	-1.326091	5.090415	1.203073
O	-6.430231	-0.067738	-2.170251
C	-7.306538	-0.358853	-2.987225
C	-8.735392	-0.552916	-2.548926
H	-9.078676	-1.579653	-2.730202
H	-8.791867	-0.349984	-1.475850
H	-9.419912	0.123914	-3.075719
N	-7.032571	-0.524898	-4.310532
C	-5.698404	-0.277820	-4.815036
H	-5.703348	0.571830	-5.514016
H	-5.029934	-0.051868	-3.981669
H	-5.324252	-1.161139	-5.351040
C	-8.023110	-0.797496	-5.331609
H	-8.942618	-1.204229	-4.905720
H	-8.268466	0.113150	-5.899568
H	-7.620921	-1.538298	-6.035495
O	3.114587	-5.183811	-1.059775
C	4.238656	-5.214107	-1.565438
C	5.309808	-6.112094	-1.000072
H	6.160750	-5.531951	-0.618303
H	4.873884	-6.679037	-0.172652
H	5.696259	-6.816689	-1.747060
N	4.565858	-4.449204	-2.642628
C	3.597418	-3.534619	-3.208699
H	3.490651	-3.718577	-4.287350
H	2.629938	-3.678514	-2.723357
H	3.922724	-2.491623	-3.068155
C	5.860936	-4.419550	-3.287812

H	6.585459	-5.056056	-2.776540
H	5.775770	-4.756833	-4.331433
H	6.251684	-3.390879	-3.293468

```
```

***

TS 8NH-c7 | Charge 0 |

Electronic Energy (a.u.) -3006.3538319

C	-4.906753	-3.027264	-0.789665
H	-5.333156	-3.461347	-1.693691
C	-5.192605	-3.529010	0.451736
H	-5.868976	-4.378191	0.560471
C	-3.999911	-1.918126	-0.953192
C	-4.610920	-2.951340	1.594116
H	-4.831797	-3.345198	2.582403
C	-3.785336	-1.852026	1.409679
C	-3.135611	-1.185069	2.566433
C	-3.144648	-1.719143	3.859215
H	-3.647067	-2.660524	4.064958
C	-2.498993	-1.045773	4.885946
H	-2.503324	-1.456790	5.893650
C	-1.842018	0.149378	4.610187
H	-1.317306	0.705948	5.382845
C	-1.866648	0.618760	3.306415
H	-1.362060	1.546395	3.027338
C	-1.732581	2.551000	0.381661
C	-0.314591	2.638587	0.378113
C	0.343999	3.706554	0.957618
C	-0.375716	4.739762	1.571010
C	-1.774227	4.662165	1.605097
C	-2.444008	3.594583	1.032736
N	-3.510872	-1.325159	0.195106
N	-2.494279	-0.025689	2.315836
O	-3.657467	-1.503086	-2.085264
Pd	-2.549436	0.750069	0.288839
C	0.292122	5.874211	2.243051
F	-0.163519	7.071957	1.817454
F	0.090187	5.881550	3.581088
F	1.622143	5.881981	2.063047
H	-3.533061	3.555364	1.084221
H	-2.342187	5.446693	2.109126
H	1.433777	3.737537	0.939420
H	0.254283	1.859403	-0.129558
H	-1.772587	1.542063	-1.956932
N	-2.388489	2.089118	-1.341459
H	-1.051462	3.271332	-2.213650
C	-3.628573	2.477716	-1.890229
C	-4.475156	1.568412	-2.546565
C	-4.015051	3.825612	-1.828303
C	-5.659976	2.005037	-3.130304
H	-4.190378	0.512228	-2.563837
C	-5.214672	4.250034	-2.393254
H	-3.351491	4.540846	-1.341495
C	-6.043067	3.344227	-3.051244
H	-6.300267	1.287550	-3.644723
H	-5.493842	5.301987	-2.333688
H	-6.977192	3.678186	-3.501116
O	1.664292	2.833351	-3.418164
O	-0.291826	3.617466	-2.728309

C	0.547634	2.550642	-2.960493
O	0.097241	1.401898	-2.672355
Cs	3.072783	0.943759	-1.346281
Cs	-0.565838	-1.540974	-2.062889
O	2.620902	0.504832	1.719539
C	2.294989	-0.215946	2.666763
C	3.081109	-0.235059	3.950516
H	3.476601	-1.237462	4.165455
H	3.914523	0.467317	3.857786
H	2.464420	0.064509	4.808045
N	1.208708	-1.028510	2.584171
C	0.518631	-1.070100	1.306294
H	0.024755	-0.109989	1.089152
H	1.224267	-1.291607	0.493148
H	-0.250912	-1.851912	1.334205
C	0.849229	-1.994718	3.605917
H	1.054345	-1.604372	4.607463
H	-0.226737	-2.198734	3.547321
H	1.391328	-2.946408	3.484774
O	2.483468	-2.212623	-1.287558
C	3.394561	-2.720189	-0.618538
C	3.114456	-3.330645	0.730597
H	3.505239	-2.692741	1.538681
H	2.029883	-3.417941	0.850485
H	3.564662	-4.324081	0.843492
N	4.678645	-2.730764	-1.055575
C	5.003018	-2.270605	-2.393777
H	5.544160	-3.059414	-2.934482
H	4.082683	-2.035868	-2.934012
H	5.638018	-1.373479	-2.350018
C	5.781904	-3.331375	-0.328347
H	5.604891	-3.322324	0.750485
H	5.958075	-4.367474	-0.654505
H	6.691625	-2.748217	-0.523614
O	-1.180783	-4.177569	-0.487218
C	-1.705910	-4.850793	0.403180
C	-1.289007	-4.688101	1.842999
H	-1.058816	-5.646705	2.324261
H	-0.395634	-4.056211	1.868912
H	-2.082590	-4.202252	2.429805
N	-2.678157	-5.765985	0.142565
C	-3.050693	-6.062271	-1.224712
H	-4.140911	-5.993925	-1.344272
H	-2.568723	-5.345624	-1.893366
H	-2.737521	-7.081075	-1.499110
C	-3.290061	-6.606653	1.152177
H	-3.323821	-6.107642	2.124716
H	-4.323738	-6.822274	0.853878
H	-2.760322	-7.565719	1.261687
O	6.182303	0.680598	-1.141960
C	7.135167	0.125716	-0.584083
C	8.450629	-0.059389	-1.293030
H	8.719781	-1.121176	-1.374539
H	8.355881	0.358451	-2.299352
H	9.271830	0.452035	-0.774896
N	7.011572	-0.357140	0.677559
C	5.720108	-0.212491	1.325579
H	5.464480	0.841167	1.500686

H	4.927822	-0.655186	0.707520
H	5.747734	-0.735131	2.288043
C	8.076813	-0.958577	1.454249
H	8.986816	-1.066116	0.860604
H	8.309455	-0.341942	2.334187
H	7.776617	-1.956120	1.804717***

c7 | Charge 0 | Electronic Energy (a.u.) -3006.40754174

C	4.399439	2.809160	-1.731899
H	4.766325	2.917220	-2.752385
C	4.556068	3.804554	-0.803069
H	5.064936	4.732474	-1.070314
C	3.721199	1.580573	-1.405996
C	4.073157	3.619882	0.504406
H	4.214049	4.374337	1.275855
C	3.466433	2.408331	0.805165
C	3.009401	2.161408	2.198817
C	2.312339	3.147551	2.902162
H	2.098186	4.106415	2.431989
C	1.875072	2.877987	4.194370
H	1.318511	3.628904	4.753842
C	2.151789	1.636136	4.751189
H	1.823604	1.374509	5.754937
C	2.875000	0.725022	3.986667
H	3.132331	-0.253217	4.398085
C	2.146001	-2.754124	0.614811
C	0.872507	-2.118760	0.694370
C	0.340274	-1.773374	1.947871
C	1.035638	-2.054085	3.119395
C	2.307234	-2.643211	3.048380
C	2.871845	-2.956454	1.822319
N	3.283404	1.419915	-0.098063
N	3.294675	0.967721	2.744049
O	3.519788	0.683888	-2.256828
Pd	2.339611	-0.501470	0.360249
C	0.386125	-1.718588	4.410173
F	1.254386	-1.640898	5.432173
F	-0.265951	-0.539150	4.357101
F	-0.544361	-2.625401	4.779480
H	3.858730	-3.412882	1.787221
H	2.870586	-2.841256	3.958982
H	-0.643698	-1.302644	1.997515
H	0.252317	-2.101800	-0.204483
H	1.929705	-2.931851	-1.372679
N	2.486406	-3.340136	-0.613158
H	1.408977	-4.065950	-3.528057
C	3.802942	-3.654651	-1.003919
C	4.355286	-3.000001	-2.111556
C	4.542137	-4.654840	-0.362916
C	5.624978	-3.339181	-2.567307
H	3.782571	-2.204739	-2.593250
C	5.820385	-4.972436	-0.811280
H	4.099098	-5.202475	0.467890
C	6.367184	-4.321223	-1.915051
H	6.041394	-2.822281	-3.431252
H	6.385646	-5.752848	-0.303350
H	7.364085	-4.581296	-2.267184
O	-1.187883	-4.133056	-2.047108

O	0.648063	-4.578084	-3.209959
C	-0.144426	-3.674326	-2.524085
O	0.308305	-2.494051	-2.467504
Cs	-2.768962	-1.876981	-0.625269
Cs	0.429294	0.616847	-2.539525
O	-2.544952	-0.126717	1.984737
C	-2.469278	0.977145	2.537556
C	-3.446082	1.377056	3.611581
H	-3.957848	2.319954	3.375812
H	-4.187840	0.581195	3.723735
H	-2.935300	1.514526	4.574432
N	-1.510828	1.881569	2.215006
C	-0.541072	1.550314	1.183915
H	0.349086	1.056937	1.611562
H	-1.000559	0.870574	0.455509
H	-0.221273	2.457637	0.653836
C	-1.436807	3.201085	2.817318
H	-1.601310	3.162777	3.899938
H	-0.433795	3.607788	2.651689
H	-2.167575	3.896851	2.375011
O	-2.573872	1.067422	-1.731840
C	-3.565102	1.710995	-1.364770
C	-3.429841	2.851566	-0.390448
H	-3.877058	2.598562	0.582305
H	-2.362995	3.051917	-0.246069
H	-3.921062	3.763130	-0.753356
N	-4.810581	1.413916	-1.815889
C	-4.992362	0.414194	-2.852589
H	-5.616569	0.828469	-3.655958
H	-4.020290	0.133280	-3.265411
H	-5.485607	-0.483394	-2.450235
C	-6.013155	2.129194	-1.433145
H	-5.906980	2.601143	-0.452696
H	-6.271905	2.900580	-2.174247
H	-6.845540	1.414831	-1.374081
O	0.489189	3.561899	-1.455518
C	1.017342	4.655964	-1.239290
C	0.796403	5.377565	0.065180
H	0.384147	6.383130	-0.090137
H	0.089920	4.795992	0.667278
H	1.734615	5.489018	0.625850
N	1.815478	5.267865	-2.155055
C	1.986285	4.691375	-3.471739
H	3.047772	4.710736	-3.752141
H	1.635710	3.656426	-3.467935
H	1.419369	5.259088	-4.225769
C	2.398910	6.581629	-1.972896
H	2.577758	6.798531	-0.916294
H	3.367553	6.613569	-2.487843
H	1.761093	7.373322	-2.396056
O	-5.895747	-1.988102	-0.620152
C	-6.946801	-1.385636	-0.375784
C	-8.204226	-1.668505	-1.153673
H	-8.553242	-0.774840	-1.689325
H	-7.983894	-2.452834	-1.883468
H	-9.022685	-2.007886	-0.506133
N	-6.985800	-0.425806	0.581769
C	-5.753443	-0.146480	1.297070

H	-5.421465	-1.010442	1.888985
H	-4.949381	0.114709	0.595689
H	-5.920895	0.701067	1.970433
C	-8.176544	0.277522	1.016057
H	-9.019670	0.076900	0.351630
H	-8.458302	-0.023616	2.035526
H	-7.994184	1.360894	1.016584

Oxidative addition of the aryl iodide to a Pd(II) complex to give a Pd(IV) derivative. Gibbs energy profile in Figure S95

```
# c8 | Charge                         0            | Electronic
Energy (a.u.)    -1270.60483265
C          -2.856965        3.313219       -0.438296
H          -2.706848        4.371102       -0.646600
C          -4.071030        2.816370       -0.062914
H          -4.928296        3.479552        0.050331
C          -1.717146        2.448850       -0.639077
C          -4.227873        1.432699        0.145944
H          -5.207254        1.019218        0.364843
C          -3.115020        0.624841        0.034358
C          -3.219705       -0.844669        0.142897
C          -4.394502       -1.504432        0.508736
H          -5.276660       -0.942613        0.802379
C          -4.431978       -2.890258        0.501009
H          -5.344366       -3.411511        0.783424
C          -3.297422       -3.600333        0.124349
H          -3.286724       -4.686439        0.089922
C          -2.157298       -2.882646       -0.199680
H          -1.243982       -3.400056       -0.489579
N          -1.877176        1.121158       -0.263131
N          -2.110303       -1.546331       -0.183840
O          -0.654768        2.868371       -1.126283
Pd         -0.339695       -0.260278       -0.049150
C           2.031024        1.581955       -0.422129
C           1.141884        0.984672        0.493324
C           3.179592        2.218433        0.064525
H           3.874957        2.664405       -0.650494
C           2.525891        1.766931        2.341003
H           2.700664        1.843704        3.414113
C           3.431112        2.304094        1.433298
H           4.331213        2.808843        1.784386
C           1.387463        1.107212        1.858553
H           0.697237        0.654797        2.575358
N           1.765190        1.504376       -1.796232
H           0.792054        1.797698       -1.931376
H           2.376081        2.120420       -2.329001
H           0.950994       -2.146685        1.201589
H           0.708263       -2.625117       -0.348316
C           2.477541       -1.703722       -0.057810
C           3.388462       -1.535880        0.981155
C           2.910721       -1.709693       -1.381683
C           4.735812       -1.344273        0.690422
H           3.036874       -1.543475        2.013016
C           4.260288       -1.529841       -1.663791
H           2.183321       -1.839019       -2.183931
C           5.176260       -1.339029       -0.630837
H           5.444780       -1.203259        1.504989
H           4.596901       -1.534248       -2.699371
H           6.231181       -1.191186       -0.855628
N           1.078152       -1.864532        0.226202***

# TS c8-c9  | Charge                              0              |
| Electronic Energy (a.u.)     -1850.30697775      |
```

C	0.581144	1.424176	3.690986
H	0.953293	1.069769	4.651064
C	0.310081	2.741964	3.466424
H	0.453587	3.477469	4.258040
C	0.443266	0.427373	2.649004
C	-0.132677	3.162928	2.197474
H	-0.280985	4.218040	1.989654
C	-0.326706	2.203441	1.222208
C	-0.705212	2.588721	-0.152895
C	-1.144724	3.873183	-0.490490
H	-1.291809	4.625142	0.279759
C	-1.400829	4.183045	-1.816467
H	-1.737665	5.181765	-2.086940
C	-1.225859	3.204808	-2.791327
H	-1.409501	3.406143	-3.843498
C	-0.819717	1.943960	-2.383827
H	-0.682759	1.135434	-3.103607
N	-0.114162	0.877167	1.451421
N	-0.568323	1.641796	-1.104830
O	0.792540	-0.747666	2.811046
C	-0.524151	-3.344677	1.001412
C	-0.951811	-2.005526	0.930964
C	-1.230285	-4.220576	1.849367
H	-0.897614	-5.259627	1.894522
C	-2.721873	-2.477437	2.546262
H	-3.567151	-2.115870	3.131633
C	-2.306051	-3.802842	2.616358
H	-2.819668	-4.515316	3.260992
C	-2.031133	-1.598323	1.713730
H	-2.355292	-0.557391	1.685862
N	0.591906	-3.854059	0.334891
H	0.600895	-4.870183	0.310114
H	0.714436	-3.489414	-0.605336
Pd	-0.392366	-0.414312	-0.230162
H	-3.105022	-1.487430	-1.117116
H	-2.726787	-0.188651	-2.093916
C	-3.944167	0.272057	-0.527601
C	-4.067968	1.639421	-0.806501
C	-4.797633	-0.304931	0.421483
C	-5.008350	2.411429	-0.135452
H	-3.422401	2.088761	-1.562345
C	-5.735033	0.476514	1.088917
H	-4.720849	-1.372880	0.627655
C	-5.845643	1.839484	0.821409
H	-5.086235	3.473755	-0.367037
H	-6.390310	0.009555	1.823424
H	-6.580560	2.447320	1.346400
N	-2.938429	-0.483885	-1.143499
I	0.432361	-1.696372	-2.500160
C	1.807434	-0.535971	-0.801274
C	2.526467	-1.311229	0.099457
C	2.321548	0.662933	-1.303599
C	3.746727	-0.834197	0.571847
H	2.142286	-2.263030	0.459645
C	3.537955	1.124054	-0.823326
H	1.789356	1.234064	-2.058522
C	4.248392	0.380838	0.120716
H	4.299959	-1.424236	1.299755

H	3.941879	2.063804	-1.199739
C	5.540300	0.929392	0.623400
F	6.190855	0.077244	1.422421
F	5.358149	2.063518	1.322518
F	6.374823	1.244699	-0.381687***

c10 | Charge 0 |

Electronic Energy (a.u.) -1850.35226005

C	1.628851	-1.279045	3.632660
H	1.641951	-0.966966	4.675374
C	2.234733	-2.422540	3.209812
H	2.772535	-3.058625	3.912094
C	0.885793	-0.425999	2.730519
C	2.152342	-2.792768	1.854386
H	2.615563	-3.711865	1.511177
C	1.494453	-1.957924	0.976028
C	1.385906	-2.312126	-0.451790
C	1.795469	-3.547410	-0.964082
H	2.186806	-4.319420	-0.308578
C	1.697995	-3.787590	-2.324253
H	2.017830	-4.744710	-2.730861
C	1.187849	-2.797383	-3.158311
H	1.098526	-2.942778	-4.231526
C	0.779055	-1.606333	-2.582172
H	0.363690	-0.799009	-3.185653
N	0.934477	-0.775023	1.378235
N	0.870372	-1.375328	-1.267717
O	0.246812	0.550554	3.140266
C	-1.313889	3.000513	0.824911
C	-0.109047	2.270799	0.813263
C	-1.325354	4.228913	1.518211
H	-2.257811	4.796737	1.520788
C	0.972318	3.986002	2.164032
H	1.861901	4.338742	2.683728
C	-0.208569	4.721723	2.170817
H	-0.263590	5.680645	2.684968
C	0.999026	2.766189	1.492195
H	1.911245	2.177239	1.541550
N	-2.511381	2.591276	0.246035
H	-3.107836	3.369142	-0.020393
H	-2.409322	1.940211	-0.525125
Pd	0.301762	0.496384	-0.204794
H	2.700052	2.013096	-0.390317
H	2.542094	1.064427	-1.723745
C	3.608081	0.191188	-0.242369
C	4.084308	-0.822742	-1.075373
C	4.112496	0.313348	1.054435
C	5.042243	-1.717546	-0.609668
H	3.695221	-0.907227	-2.091495
C	5.070100	-0.584327	1.512417
H	3.755650	1.110705	1.706726
C	5.535393	-1.607713	0.687775
H	5.401682	-2.506576	-1.269129
H	5.453314	-0.483296	2.526987
H	6.282751	-2.309655	1.053489
N	2.570429	1.049531	-0.702708
I	-0.243921	1.968447	-2.432971
C	-1.571353	-0.254620	0.073367
C	-2.130843	-0.311494	1.345258

C	-2.246388	-0.776081	-1.030125
C	-3.392629	-0.878166	1.512453
H	-1.594063	0.096619	2.200724
C	-3.507955	-1.336904	-0.854096
H	-1.820543	-0.740694	-2.029868
C	-4.079951	-1.384969	0.415096
H	-3.834832	-0.919113	2.505949
H	-4.046058	-1.737460	-1.713457
C	-5.432633	-1.999682	0.554726
F	-5.888671	-1.972666	1.811473
F	-5.437244	-3.284956	0.161300
F	-6.343922	-1.374716	-0.210014***

References

(23) Gaussian 16, Revision C.01, Frisch, M. J.; Trucks, G. W.; Schlegel, H. B.; Scuseria, G. E.; Robb, M. A.; Cheeseman, J. R.; Scalmani, G.; Barone, V.; Petersson, G. A.; Nakatsuji, H.; Li, X.; Caricato, M.; Marenich, A. V.; Bloino, J.; Janesko, B. G.; Gomperts, R.; Mennucci, B.; Hratchian, H. P.; Ortiz, J. V.; Izmaylov, A. F.; Sonnenberg, J. L.; Williams-Young, D.; Ding, F.; Lipparini, F.; Egidi, F.; Goings, J.; Peng, B.; Petrone, A.; Henderson, T.; Ranasinghe, D.; Zakrzewski, V. G.; Gao, J.; Rega, N.; Zheng, G.; Liang, W.; Hada, M.; Ehara, M.; Toyota, K.; Fukuda, R.; Hasegawa, J.; Ishida, M.; Nakajima, T.; Honda, Y.; Kitao, O.; Nakai, H.; Vreven, T.; Throssell, K.; Montgomery, J. A., Jr.; Peralta, J. E.; Ogliaro, F.; Bearpark, M. J.; Heyd, J. J.; Brothers, E. N.; Kudin, K. N.; Staroverov, V. N.; Keith, T. A.; Kobayashi, R.; Normand, J.; Raghavachari, K.; Rendell, A. P.; Burant, J. C.; Iyengar, S. S.; Tomasi, J.; Cossi, M.; Millam, J. M.; Klene, M.; Adamo, C.; Cammi, R.; Ochterski, J. W.; Martin, R. L.; Morokuma, K.; Farkas, O.; Foresman, J. B.; Fox, D. J. Gaussian, Inc., Wallingford CT, 2016.

(24) (a) Zhao, Y.; Truhlar, D. G. J. A new local density functional for main-group thermochemistry, transition metal bonding, thermochemical kinetics, and noncovalent interactions. *Chem. Phys.* **2006**, *125*, 194101-194118. DOI:10.1063/1.2370993. (b) Zhao, Y.; Truhlar, D. G. The M06 suite of density functionals for main group thermochemistry, thermochemical kinetics, noncovalent interactions, excited states, and transition elements: two new functionals and systematic testing of four M06-class functionals and 12 other functionals. *Theor. Chem. Acc.* **2006**, *120*, 215-241. DOI: 10.1007/s00214-007-0310-x.

(25) (a) Francl, M. M; Petro, W. J.; Hehre, W. J.; Binkley, J. S.; Gordon, M. S.; DeFrees, D. J.; Pople, J. A. Self-consistent molecular orbital methods. XXIII. A polarization-type basis set for second-row elements. *J. Chem. Phys.* **1982**, *77*, 3654-3665. DOI: 10.1063/1.444267. (b) Clark, T.; Chandrasekhar, J.; Schleyer, P. V. R. J. Efficient diffuse function-augmented basis sets for anion calculations. III.The 3-21+G basis set for first-row elements. Li–F, *J. Comput. Chem.* **1983**, *4*, 294-301. DOI: 10.1002/jcc.540040303.

(26) (a) Ehlers, A. W.; Böhme, M.; Dapprich, S.; Gobbi, A.; Höllwarth, A.; Jonas, V.; Köhler, K. F.; Stegmann, R.; Veldkamp, A.; Frenking, G., A set of f-polarization functions for pseudo-potential basis sets of the transition metals Sc-Cu, Y-Ag and La-Au. *Chem. Phys. Lett.* **1993,** *208*, 111-114. DOI: 10.1016/0009-2614(93)80086-5. (b) Roy, L. E.; Hay, P. J.; Martin, R. L. J., Revised Basis Sets for the LANL Effective Core Potentials. *J. Chem. Theory Comput.* **2008**, *4,* 1029-1031. DOI:10.1021/ct8000409.

(27) Hay, P. J.; Wadt, W. R. Ab Initio Effective Core Potentials for Molecular Calculations. Potentials for the Transition Metal Atoms Sc to Hg. *J. Chem. Phys.* **1985**, *82*, 270–283.

(28) Norjmaa, G.; Ujaque, G.; Lledós, A. Beyond Continuum Solvent Models in Computational Homogeneous Catalysis. *Top. Catal.* **2022**, *65*, 118–140. DOI:10.1007/s11244-021-01520-2

(29) Marenich, A. V.; Cramer, C. J.; Truhlar, D. G. Universal Solvation Model Based on Solute Electron Density and on a Continuum Model of the Solvent Defined by the Bulk Dielectric Constant and Atomic Surface Tensions. *J. Phys. Chem. B* **2009**, *113*, 6378−6396.

(30) (a) Riplinger, C.; Neese, F. An Efficient and near Linear Scaling Pair Natural Orbital Based Local Coupled Cluster Method. *J. Chem. Phys.* **2013**, *138*, 034106. (b) Riplinger, C.; Sandhoefer, B.; Hansen, A.; Neese, F. Natural Triple Excitations in Local Coupled Cluster Calculations with Pair Natural Orbitals. *J. Chem. Phys.* **2013**, *139*, 134101. (c) Riplinger, C.; Pinski, P.; Becker, U.; Valeev, E. F.; Neese, F. Sparse Maps—A Systematic Infrastructure for Reduced-Scaling Electronic Structure Methods. II. Linear Scaling Domain Based Pair Natural Orbital Coupled Cluster Theory. *J. Chem. Phys.* **2016**, 144, 024109.

(31) (a) Neese, F. The ORCA program system. *WIREs Comput. Mol. Sci.* **2012**, *2*, 73−78. (b) Neese, F. Software update: the ORCA program system, version 4.0. *WIREs Comput. Mol. Sci.* **2018**, *8*, e1327.

(32) (a) Schäfer, A.; Horn, H.; Ahlrichs, R. Fully optimized contracted Gaussian basis sets for atoms Li to Kr. *J. Chem. Phys.* **1992**, *97*, 2571−2577. (b) Weigend, F.; Ahlrichs, R. Balanced basis sets of split valence, triple zeta valence and quadruple zeta valence quality for H to Rn: Design and assessment of accuracy. *Phys. Chem. Chem. Phys.* **2005**, *7*, 3297−3305. (c) Weigend, F. Accurate Coulomb-fitting basis sets for H to Rn. *Phys. Chem. Chem. Phys.* **2006**, *8*, 1057−1065.

(33) Neese, F.; Wennmohs, F.; Hansen, A.; Becker, U. Efficient, Approximate and Parallel Hartree-Fock and Hybrid DFT Calculations. A 'Chain-of-Spheres' Algorithm for the Hartree-Fock Exchange. *Chem. Phys.* **2009**, *356*, 98–109.

(34) (a) Andrae, D.; Häussermann, U.; Dolg, M.; Stoll, H.; Preuss, H. Energy-Adjusted ab Initio Pseudopotentials for the Second and Third Row Transition Elements. *Theoret. Chim. Acta* **1990**, *77*, 123–141. (b) Leininger, T.; Nicklass, A.; Kuechle, W.; Stoll, H.; Dolg, M.; Bergner, A. The accuracy of the pseudopotential approximation: non-frozen-core effects for spectroscopic constants of alkali fluorides XF (X = K, Rb, Cs). *Chem. Phys. Lett.* **1996**, *255*, 274-280.

(35) Bryantsev, V. S.; Diallo, M. S.; Goddard III, W. A. Calculation of Solvation Free Energies of Charged Solutes Using Mixed Cluster/Continuum Models. *J. Phys. Chem. B 2008*, *112*, 9709-9719.

(36) Legault, C. Y. CYLview20; Université de Sherbrooke, 2020; http://www.cylview.org

(37) COmplex PAthway Simulator (COPASI) is an easily available free software: Hoops, S.; Sahle, S.; Gauges, R.; Lee, C.; Pahle, J.; Simus, N.; Singhal, M; Xu, L.; Mendes, P.; Kummer, U. COPASI–A COmplex PAthway SImulator *Bioinformatics,* **2006**, *22*, 3067-3074.
